# Synthesis of malhamensilipin A exploiting iterative epoxidation/chlorination: experimental and computational analysis of epoxide-derived chloronium ions†
†Electronic supplementary information (ESI) available. CCDC 1470484 and 1470483. For ESI and crystallographic data in CIF or other electronic format see DOI: 10.1039/c6sc03012b



**DOI:** 10.1039/c6sc03012b

**Published:** 2016-08-02

**Authors:** J. Saska, W. Lewis, R. S. Paton, R. M. Denton

**Affiliations:** a School of Chemistry , University Park , Nottingham , NG7 2RD , UK . Email: ross.denton@nottingham.ac.uk; b Chemistry Research Laboratory , 12 Mansfield Road , Oxford OX1 3TA , UK . Email: robert.paton@chem.ox.uk

## Abstract

We report a 12-step catalytic enantioselective formal synthesis of malhamensilipin A (**3**) and diastereoisomeric analogues from (*E*)-2-undecenal. The convergent synthesis relied upon iterative epoxidation and phosphorus(v)-mediated deoxydichlorination reactions as well a titanium-mediated epoxide-opening to construct the C11–C16 stereohexad. The latter transformation occurred with very high levels of stereoretention regardless of the C13 configuration of the parent epoxide, implicating anchimeric assistance of either the γ- or δ-chlorine atoms, and the formation of chloretanium or chlorolanium ions, respectively. A computational analysis of the chloronium ion intermediates provided support for the involvement of chlorolanium ions, whereas the potential chloretanium ions were found to be less likely intermediates on the basis of their greater carbocationic character.

## Abstract 2

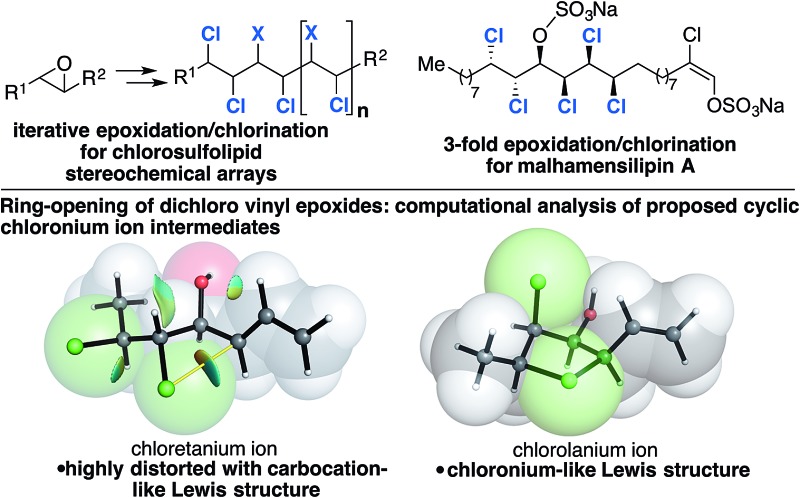
We report a 12-step catalytic enantioselective formal synthesis of malhamensilipin A (**3**) based upon an iterative epoxidation/chlorination strategy.

## Introduction

**Scheme 1:**
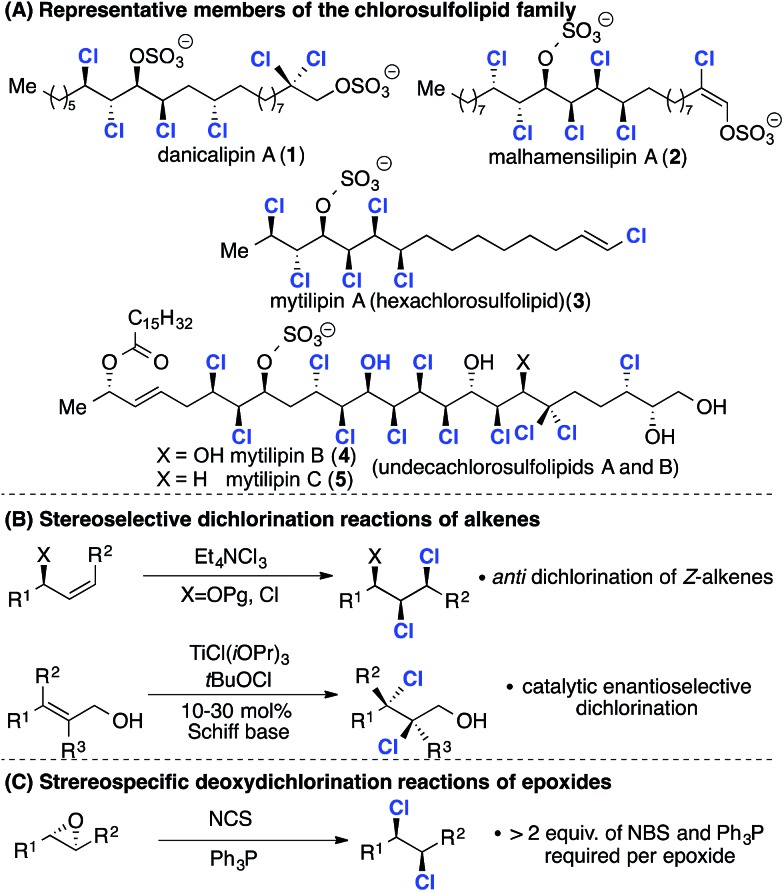
(A) Selected chlorosulfolipids. (B) and (C) Synthesis methods.

The chlorosulfolipids (Scheme 1A) are a family of bioactive polychlorinated natural products^1^ isolated from algal sources and toxic mussels.^2^ Since the original reports in the late 1960s,^3^ the family has grown and many of the gross structures have been refined to include relative and absolute stereochemistry.^4^ The stereochemical challenges posed by these unique lipids have stimulated the interest of synthesis chemists around the world and the first total synthesis of a chlorosulfolipid was reported by the Carreira group in 2009.^5^ Following this work syntheses of mytilipin A (**3**),^6^ danicalipin A (**1**),^7^ malhamensilipin A (**2**)^8^ and mytilipin B (**4**)^9^ were reported (Scheme 1A).

In terms of synthesis strategies two complementary dichlorination reactions have been applied. The first method (Scheme 1B, top), examined in detail by Vanderwal and co-workers, involves *anti* dichlorination of alkenes using tetraethylammonium trichloride.^10^ This dichlorination reaction is a feature of many of the groundbreaking syntheses reported by both the Vanderwal and Carreira groups.^2^ Very recently Burns and co-workers have developed catalytic enantioselective chlorination reactions of allylic alcohols and applied them in synthesis of (deschloro)mytilipin A and danicalipin A (**2**) (Scheme 1B, bottom).^11^ This work, along with other methods developed by the Snyder,^12^ Nicolaou and^13^ Denmark^14^ groups opens up new approaches to polyhalogenated target molecules. The second approach (Scheme 1C) involves deoxydichlorination reactions of epoxides, reported by Tanaka and Yoshimitsu in 2009.^15^ While this reaction has found application in the synthesis of mytilipin A (**4**) and danicalipin A (**2**)^6a,7b^ it generates stoichiometric quantities of organic waste.

**Scheme 2:**
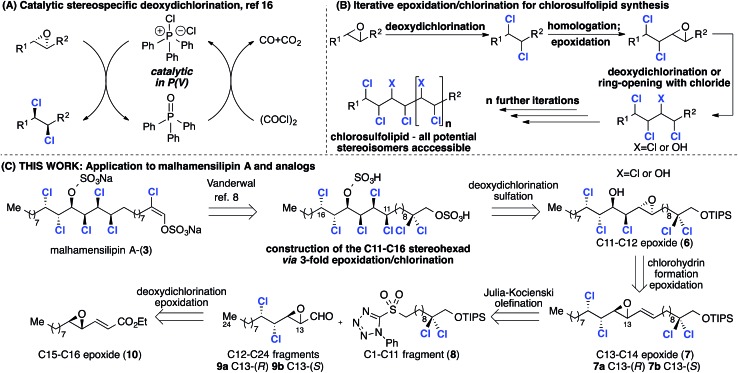
(A) Catalytic deoxydichlorination of epoxides (B) general synthesis strategy for chlorosulfolipids (C) application to malhamensilipin A.

We reasoned that a catalytic deoxydichlorination reaction would provide a powerful alternative to alkene dichlorination and in 2011 we reported a catalytic reaction that generates CO_2_ and CO as the sole by-products (Scheme 2A).^16^ The integration of this reaction into an iterative epoxidation/chlorination sequence (Scheme 2B) provides a general and systematic approach to the chlorine- and hydroxyl-containing acyclic stereochemical arrays present within the chlorosulfolipids (Scheme 2B). Significantly, any stereochemical permutation can be accessed using this approach by control of epoxide configuration.

Herein we demonstrate the power of the iterative epoxidation/chlorination strategy (Scheme 2B) with a catalytic enantioselective formal synthesis of malhamensilipin A^4*f*^ (**2**) and stereoisomeric analogues. We also illustrate the applicability of Carreira's NMR database^17^ for the assignment of polychlorinated stereotetrads and stereohexads. Finally, we analyse ring-opening reactions of γ,δ-dichlorovinyl epoxides and report computational studies that give new insights into the structure of the chloronium ion intermediates which have been proposed to intervene in these reactions.

### Synthesis plan

Our retrosynthetic analysis of malhamensilipin A (Scheme 2C) is based upon three iterations of the epoxidation/chlorination sequence for the construction of the C11–C16 stereohexad and involves epoxides **10**, **7** and **6** as intermediates. While the stereochemical outcome of the two deoxydichlorination processes was secure, the ring opening of epoxide **7** could occur with either retention or inversion of configuration at C13 (ref. 5) and therefore both C13 diastereoisomers, **7a** and **7b** were targeted. Disconnection of the C11–C12 *E*-olefin was predicated upon the modified Julia–Kocienski olefination and led to sulfone **8** and aldehydes **9a** and **9b**. The aldehydes, in either diastereoisomeric series, were projected to arise from enoate **10**, itself derived from *E*-2-undecenal.

## Results and discussion

### Fragment synthesis

**Scheme 3:**
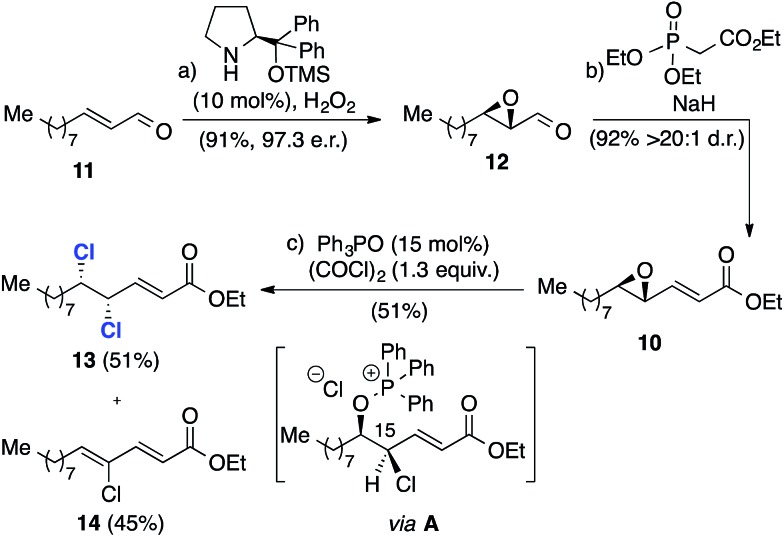
Towards the C12–C24 fragment. Reagents and conditions: (a) 10 mol% catalyst, H_2_O_2_, CHCl_3_, r.t., 91%, 97 : 3 e.r.; (b) NaH, THF –78 °C to r.t., 92%, >20 : 1 d.r.; (c) 15 mol% Ph_3_PO, 1.3 equiv. (COCl)_2_, C_6_H_6_, 80 °C, 51%.

We began with the synthesis of the more complex C12–C24 fragment (Scheme 3). Thus, catalytic enantioselective nucleophilic epoxidation of enal **11**,^18,19^ provided the corresponding epoxide **12** in excellent yield and 97 : 3 enantiomeric ratio. The conversion of the epoxide into enoate **10** was effected through a Horner–Wadsworth–Emmons reaction, which provided the enoate with a d.r. in excess of 20 : 1. Phosphorus(v)-mediated catalytic deoxydichlorination^16^ of this substrate afforded dichloride **13** with the required C15–C16 *syn* stereochemistry in 51% isolated yield. By-product **14** accounted for the remainder of the mass-balance and is most likely derived from a competing elimination from the chloroalkoxyphosphonium salt intermediate **A**.^20^

**Scheme 4:**
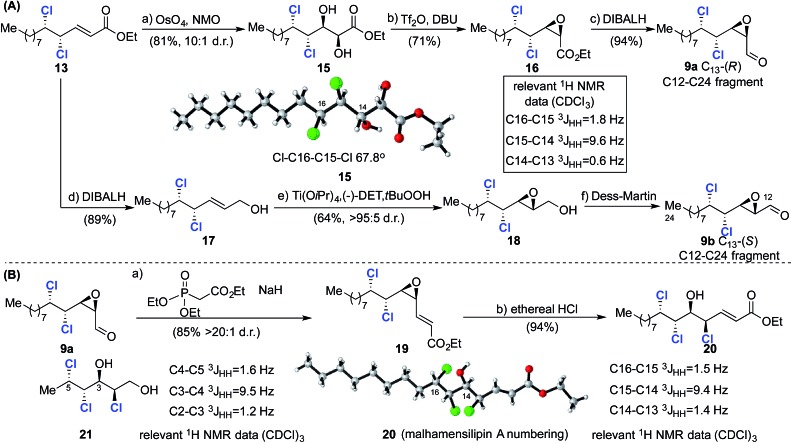
(A) Completion of the diastereoisomeric C12–C24 fragments **9a** and **9b** reagents and conditions: (a) 5 mol% OsO_4_, NMO, 2 : 1 acetone/H_2_O, r.t., 16 h, 81%, >10 : 1 d.r.; (b) Tf_2_O, DABCO, CH_2_Cl_2_, –78 °C to r.t., 16 h, 71%; (c) DIBALH, toluene, –78 °C, 1 h, 94%; (d) DIBALH, toluene, 0 °C, 15 min, 89%; (e) (–)-DET, Ti(Oi-Pr)_4_, *t*-BuOOH, CH_2_Cl_2_, –20 °C, 24 h, 64%; (f) Dess–Martin periodinane, CH_2_Cl_2_, r.t., 3 h. (B) Opening of the C13–C14 epoxide with inversion of configuration at C13 in a model system reagents and conditions: (a) NaH, THF, –78 °C to r.t., 2 h, 85%, >20 : 1 *E*/*Z*; (b) HCl·Et_2_O, 0 °C to r.t., 16 h, 94%.

With intermediate **13** in hand, the two diastereoisomeric C12–C24 aldehydes **9a** and **9b** were prepared (Scheme 4A). A sequence consisting of diastereoselective Upjohn dihydroxylation (81%, 10 : 1 d.r.), cyclization and reduction (**15** → **16** → **9a**, Scheme 4) afforded epoxide **9a**.^21^ The stereochemical outcome of the dihydroxylation was confirmed by an X-ray crystal structure of **15** and is in agreement with results previously obtained by Carreira^5^ (notwithstanding the stereochemical difference at C10) and Vanderwal, who introduced the C12 and C13 hydroxyl groups after dichlorination.^8^ Inspection of the crystal structure of **15** indicated a *gauche* conformation with respect to the C15 and C16 chlorine atoms and that the C14 hydroxyl and C15 chlorine are oriented *anti* with respect to each other. Analysis of the relevant vicinal coupling constants (Scheme 4) revealed that the molecule adopts the same conformation in solution. The diastereoisomeric epoxide **9b** was prepared *via* reduction of **13** to afford allylic alcohol **17** followed by sharpless epoxidation and oxidation with Dess–Martin periodinane.

Having established reliable and selective synthesis routes to the required C12–C24 aldehydes, we investigated the critical nucleophilic ring opening of the C13–C14 epoxide. Similar ring opening reactions had been observed to take place with retention^5,7a^ and inversion of configuration at C13.^8^ In the first instance we investigated chlorohydrin formation on model compound **19**, prepared from aldehyde **9a** *via* a Horner–Wadsworth–Emmons reaction (85%, >20 : 1 d.r., Scheme 4B).

Treatment of **19** with ethereal HCl afforded chlorohydrin **20** as a single diastereoisomer, the stereochemistry of which was assigned *via* X-ray crystallography and ^1^H NMR analysis (Scheme 4B). These data confirm inversion of configuration at C13 and hence the formation of the desired *syn*/*anti*/*syn* stereotetrad. The stereochemistry of **20** could also be assigned using model compounds in Carreira's NMR database.^17*a*^

**Scheme 5:**
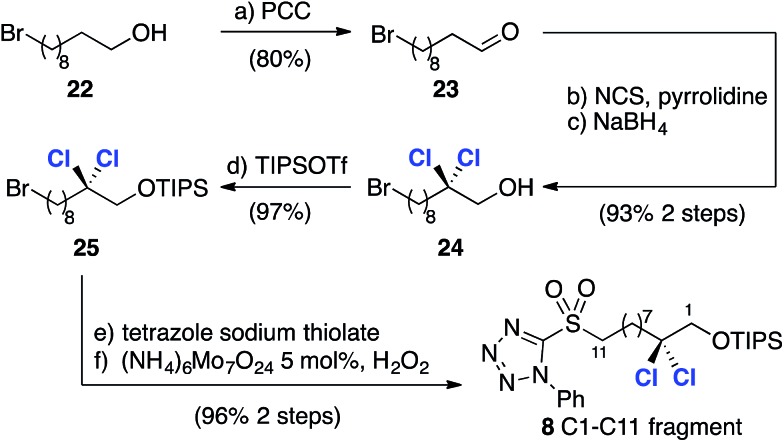
Synthesis of the C1–C11 fragment **8**. Reagents and conditions: (a) PCC, CH_2_Cl_2_, r.t., 16 h, 80%; (b) NCS, 20 mol% pyrrolidine, 1,2-dichloroethane, 60 °C, 3 h; (c) NaBH_4_, EtOH, 0 °C to r.t., 1 h, 93% over two steps; (d) TIPSOTf, 2,6-lutidine, CH_2_Cl_2_, r.t., 2 h, 97%; (e) sodium 1-phenyl-1*H*-tetrazole-5-thiolate, DMF, r.t., 4 h, quantitative; (f) 5 mol% ammonium heptamolybdate, H_2_O_2_, EtOH, 0 °C to r.t., 16 h, 96%.

Thus, comparison of **20** with reference compound **21** ^17*a*^ revealed very closely matching ^3^ *J*_HH_ coupling constants, highlighting the utility of the database approach and giving us confidence in its application to more complex fragments in the absence of X-ray crystallographic data. Following this encouraging result we focused on the synthesis of the less complex C1–C11 fragment (Scheme 5). To this end, PCC-mediated oxidation of **22** afforded aldehyde **23**, which was subjected to pyrrolidine-catalyzed α,α-dichlorination using NCS.^8^ The highly sensitive aldehyde so obtained was immediately reduced to the corresponding alcohol to afford **24** in 93% isolated yield over the two steps. Installation of the required phenyltetrazolesulfonyl group was then accomplished in two straightforward steps after protection of the alcohol to afford compound **8**.

### Fragment coupling and chlorohydrin formation

**Scheme 6:**
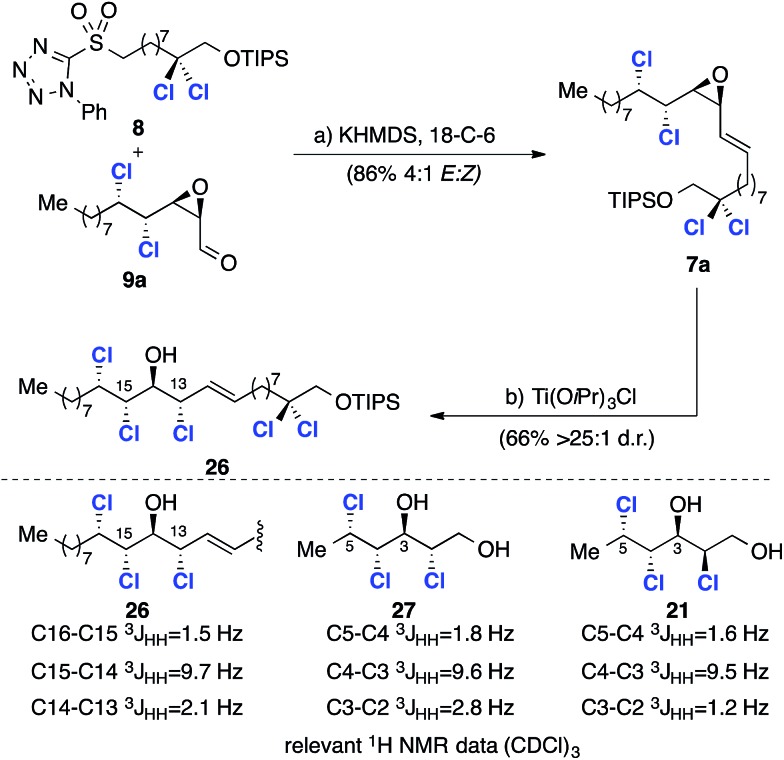
Fragment coupling and installation of C13 chlorine with retention of configuration reagents and conditions: (a) KHMDS, 18-crown-6, THF, –78 °C to r.t., 30 s, 86%, 4 : 1 *E* : *Z*; (b) TiCl(OiPr)_3_, C_6_H_6_, –20 °C, 66%, >25 : 1 d.r.

Our synthesis plan (Scheme 2C) is based on stereospecific chlorination reactions of epoxides to construct the C11–C16 stereohexad. Based upon this analysis, an *E*-selective olefination reaction was required for the fragment coupling and we selected the Julia–Kocienski reaction for this purpose.^22^ With the requisite fragments available in sufficient quantities, we examined the key olefination reaction using aldehyde **9a** (Scheme 6). The olefination reaction was examined in detail^23^ beginning with the original conditions reported by Kocienski before investigating conditions reported by Jacobsen (LiHMDS, DMF/HMPA)^24^ and, finally, Popisil (KHMDS, 18-C-6).^25^ The optimized coupling process, from which alkene **7a** was obtained in 86% yield and 4 : 1 d.r., is depicted in Scheme 6. Since **7a** was inseparable from its *Z*-isomer, the key epoxide opening and introduction of the C13 chlorine substituent was investigated using the crude product. After some experimentation with alternative reagents,^23^ namely, ethereal HCl and thionyl chloride, we discovered that chlorotitanium isopropoxide in toluene was a very effective reagent for the conversion of γ-δ-chlorovinyl epoxides into chlorohydrins and product **26** was obtained in good yield and excellent stereoselectivity (Scheme 6). While this reagent has been used for ring opening reactions of hydroxyepoxides;^17*a*^ it has not previously been applied to γ-δ-chlorovinyl epoxides and the high selectivity obtained is noteworthy. To assign the configuration at C13 we again compared the ^3^ *J*_HH_ coupling constants of the key C13–C16 region with two relevant (enantiomeric) fragments from Carreira's database (Scheme 6).^17*a*^ We began by comparing the major chlorohydrin product **26** with model compound **21**, which has the relative stereochemistry required for the natural product and corresponds to epoxide opening with inversion at C13. A significant discrepancy between the C13–C14 and C2–C3 ^3^ *J*_HH_ values was observed. However, further comparison with model compound **27** revealed very closely matching coupling constants. Therefore, the stereochemistry was assigned as depicted in structure **26**, which is the result of epoxide opening with retention of stereochemistry at C13. As noted above this outcome has been observed before, but contrasts with the result obtained with model compound **19** (Scheme 4B), pointing to a potential electronic effect (*vide infra*).

**Scheme 7:**
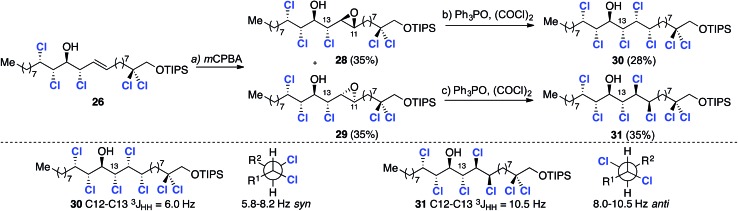
Final epoxidation/deoxydichlorination and synthesis of diastereoisomeric analogs reagents and conditions: (a) *m*CPBA, CH_2_Cl_2_, 0 °C to r.t., 70% combined yield, 1 : 1 **29** and **30**; (b) 1.5 equiv. Ph_3_PO, 1.5 equiv. (COCl)_2_, toluene, 90 °C, 3 h, 27%; (c) 1.5 equiv. Ph_3_PO, 1.5 equiv. (COCl)_2_, toluene, 90 °C, 3 h, 35%.

In order to confirm our stereochemical assignments and investigate the final epoxidation and deoxydichlorination sequence, compound **26** was taken forward (Scheme 7). Thus electrophilic epoxidation of **26** with *m*CPBA afforded a separable 1 : 1 mixture of epoxides **28** and **29** (Scheme 7). Since it was not possible to assign the configuration at C11 or C12 the stereochemical assignment was carried out retrospectively after successful deoxydichlorination of both epoxides (Scheme 7).

This transformation occurred in 28% and 36% isolated yield for epoxides **28** and **29** respectively. In each case by-products arising from elimination were also observed. Given that the deoxydichlorination reaction takes place with inversion of configuration at both stereogenic centers of the epoxide, the C11–C12 relative stereochemistry in both compounds was assumed to be *syn*. To determine the relative configuration between the chlorines at C12 and C13 the ^3^ *J*_HH_ coupling constants were examined (Scheme 7). Based upon Carreira's previous study,^17*a*^ a value of 6.0 Hz is indicative of a *syn* relationship between C13 and C12 and, therefore, compound **30** was assigned as having a *syn*,*anti*,*anti*,*syn*,*syn* stereohexad. The higher value of 10.5 Hz is characteristic of an *anti* relationship between the C13 and C12 chlorine substituents and hence a *syn*,*anti*,*anti*,*anti*,*syn* stereohexad, as depicted in structure **31**. While not providing the correct stereochemistry required for malhamensilipin A, these studies established the viability of our synthesis plan based upon a threefold epxoditation/chlorination approach. We next sought to correct the stereochemistry at C13 in order to complete a synthesis of malhamensilipin A.

### Completion of the synthesis

**Scheme 8:**
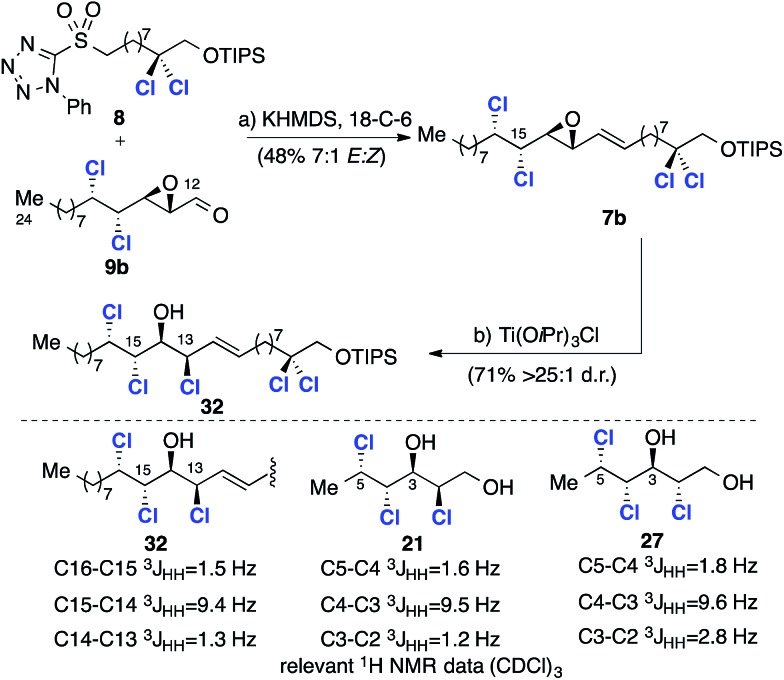
Synthesis of the carbon skeleton with inverted configuration at C13 reagents and conditions: (a) KHMDS, 18-crown-6, THF, –78 °C, 30 s, then **10b**, –78 °C to r.t., 16 h, 48% from 19, 7 : 1 *E*/*Z*; (b) TiCl(Oi-Pr)_3_, toluene, –20 °C, 2 h, 71%, >25 : 1 d.r.

**Scheme 9:**
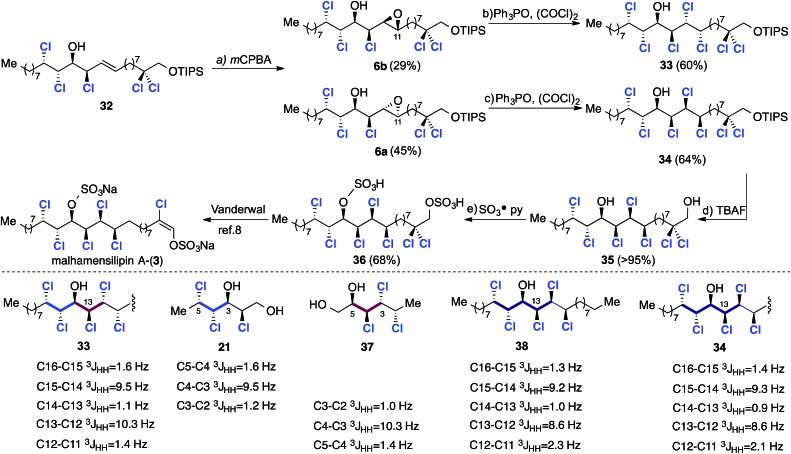
Completion of a formal synthesis of malhamensilipin A reagents and conditions: (a) *m*CPBA, CH_2_Cl_2_, 0 °C to r.t., 24 h, 74%, 2 : 1 **7a** : **7b**; (b) 1.5 eq. Ph_3_PO, 1.5 eq. (COCl)_2_, toluene, 90 °C, 3 h, 64%; (c) 1.5 eq. Ph_3_PO, 1.5 eq. (COCl)_2_, toluene, 90 °C, 3 h 60% (d) TBAF, AcOH, THF, r.t., 16 h, quantitative; (e) SO_3_-pyridine, DMF, r.t., 1 h, 68%.

Given that the C13 epoxide opening had been observed to take place with stereoretention using TiCl(OiPr)_3_, we progressed the alternative C12–C24 fragment **9b** with inverted C13 stereochemistry (Scheme 8). To this end, coupling with the C1–C11 sulfone **8** under the previously identified conditions afforded intermediate **7b** with improved selectivity (7 : 1 d.r.), but in lower yield – a consequence of the relative instability of aldehyde **9b** compared to **9a**. The critical epoxide opening reaction was then investigated (Scheme 8). We assumed that in this stereochemical series the epoxide opening would again take place with retention of configuration at C13 when using TiCl(OiPr)_3_ and this was confirmed by comparison of **32** with model compounds **21** and **27** ^17*a*^ (Scheme 8). Subsequent epoxidation of **32** with *m*CPBA (Scheme 9) gave two separable epoxides **6a** and **6b** in a 2 : 1 ratio. Again, stereochemical assignments of these key intermediates were performed retrospectively after the introduction of the final two chlorines. First, we subjected the minor diastereoisomer to deoxydichlorination and obtained the corresponding heptachlorinated compound **33** in good yield (Scheme 9).

This final deoxydichlorination process is noteworthy for two reasons: (a) the reaction is chemoselective – the C14 hydroxyl group does not undergo deoxychlorination; and (b) the introduction of chlorine at C12 occurs despite the presence of the flanking chlorines at C13 and C11. These observations demonstrate that a combination of steric and electronic effects prevents activation of the C14 hydroxyl group by the electrophilic chlorophosphonium salt.

The configuration of **33** was deduced by comparison with models **21** and **37**,^17*a*^ which contain matching stereotriads (highlighted in blue and red).

The close match of the relevant vicinal coupling constants are congruous with the non-natural *syn*/*anti*/*syn*/*anti*/*syn* stereochemistry depicted in structure **33**. With this analysis in mind, deoxydichlorination of the major epoxide **6a** was undertaken with the expectation that the correct *syn*/*anti*/*syn*/*syn*/*syn* stereochemistry would result. The heptachlorinated material **34** was obtained in good yield and compared very favourably with model compound **38**, which contains an identical stereohexad.^4*f*^ Further spectroscopic comparison of **34** with Vanderwal's TBDMS protected analogue^8^ confirmed the desired stereochemistry.

In order to complete a formal total synthesis of malhamensilipin A the silyl ether protecting group was removed in excellent yield using TBAF to afford diol **35**, which was subsequently converted into diacid **36** using sulfur trioxide pyridine complex. Compound **36** had previously been prepared by Vanderwal and was the penultimate intermediate in his total synthesis of the natural product.^8^ All spectroscopic data for **36** were in excellent agreement with those previously reported, thereby completing a 12-step formal synthesis of malhamensilipin A (**3**).

### Stereoretentive ring opening reactions of γ- and δ-dichlorovinylepoxides: the intermediacy of chloronium ion intermediates

**Scheme 10:**
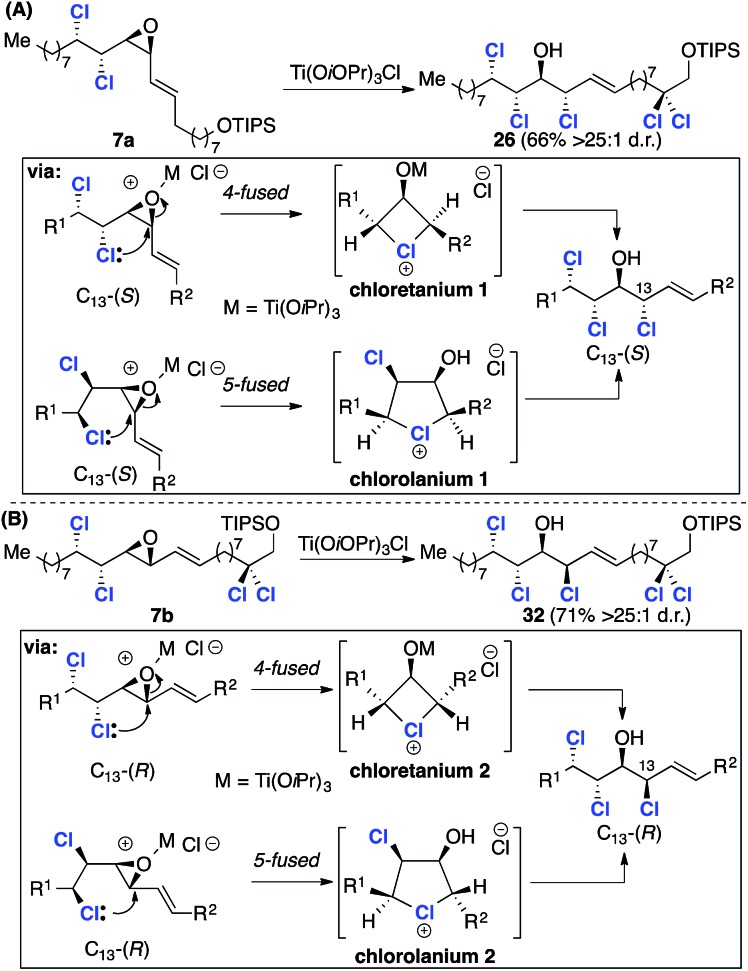
Intervention of chloronium ions in epoxide opening reactions of **7a** and **7b**.

The synthesis described in the previous sections, along with several other chlorosulfolipid syntheses, relied upon nucleophilic opening of γ,δ-dichlorovinylepoxide intermediates for the introduction of the chlorohydrin motif. The stereochemical outcome of such reactions has been shown to occur with both retention (*e.g.* **7a** → **26**, Scheme 6 and **7b** → **32**, Scheme 8) and inversion (*e.g.* **19** → **20**, Scheme 4B). Stereoretentive reactions were first observed and documented by Carreira during the synthesis of mytilipin A^5^ and have subsequently been observed by Vanderwal.^7*a*^ Carreira has proposed that retention of configuration occurs as a result of a double inversion process in which anchimeric assistance from either the γ- or δ-chlorine substituent affords a chloronium ion intermediate, which itself undergoes subsequent nucleophilic substitution.^5^ Carreira's analysis can be transferred to the ring opening reactions that were discussed in the previous section of this Article (Scheme 10).

In each case there are two potential chloronium ion intermediates arising from attack of either the γ- or δ-chlorine substituent on C13 in either a 5 or 4-fused^26^ (*endo*) sense, respectively. Two further possible chloronium ions arising from attack at C12 in either a 4- or 3-spiro (*exo*) cyclisation modes have not been considered as the products arising from these modes of attack were not isolated, presumably as a result of the alkene activating C13 with respect to nucleophilic attack.^27^ The observed retention with *both* **7a** and **7b** coupled with numerous elegant studies on chloronium species, including cyclic structures,^28,29^ makes this a compelling explanation. More recent work by Carreira^30^ on the ring opening reactions of a collection of γ- and δ-chloro vinyl epoxides has provided further results that are consistent with the intervention of four-(chloretanium) and five-(chlorolanium) membered ring ions. In comparing the early work of Peterson and Olah^28,29^ with recent studies we noted that all of the substrates that have been investigated in the context of chlorosulfolipid synthesis are vinyl epoxides. Given that the alkene substituent would be expected to influence the structure of any derived chloronium ion intermediates we carried out a theoretical study to investigate the structures and energies of the unsaturated cyclic chloronium ions depicted in Scheme 10.^31^

We began our computational investigation with a comparison of the simple dipropyl substituted chloronium ion **39** with its unsaturated analogy **40**. Equilibrium geometries for these two species were optimized using a variety of methods at the wavefunction and density functional level of theory (Table 1), with implicit solvation by an SMD description of toluene.^32^

**Table 1:** Comparison of alkyl and allyl substituted chloronium ions (BS1 = def2-TZVPP; BS2 = def2-QZVPP)

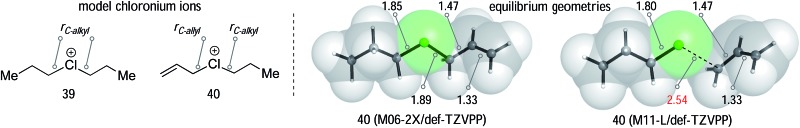						
Entry	Level of theory	*r*_C–Cl_ **39** (Å)	*r*_C–allyl_ **40** (Å)	*r*_C–alkyl_ **40** (Å)	Δ*r* (Å)	Δ*E* (kcal mol^–1^)
1	B3YLP/BS1	1.89	2.00	1.88	0.12	11.2
2	B3LYP-D3/BS1	1.89	1.98	1.88	0.10	14.8
3	ωB97XD/BS1	1.85	1.90	1.85	0.05	14.9
4	M06-2X/BS1	1.85	1.89	1.85	0.04	16.8
5	M11/BS1	—	1.89	1.85	0.04	16.2
6	M11-L/BS1	1.83	2.54	1.80	0.74	8.1
7	MP2/BS1	1.84	1.87	1.84	0.03	—
8	B2-PLYP-D3/BS1	1.87	1.93	1.86	0.07	—
9	B3LYP/BS2	1.89	2.01	1.88	0.13	—
10	B3LYP-D3/BS2	1.89	1.99	1.88	0.11	—
11	ωB97XD/BS2	1.86	1.90	1.85	0.05	—
12	M06-2X/BS2	1.86	1.89	1.85	0.04	—
13	M11/BS2	—	1.90	1.85	0.05	—
14	M11-L/BS2	1.84	1.94	1.83	0.11	—

**Fig. 1:**
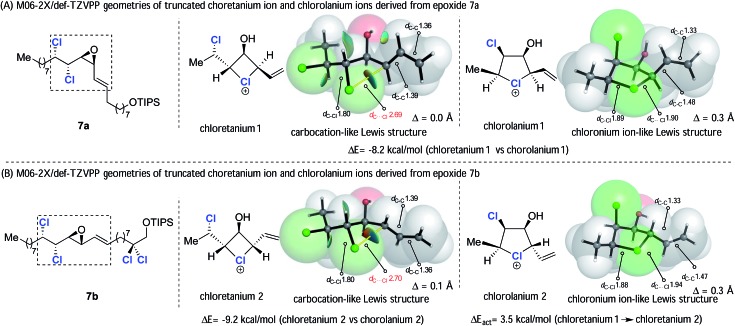
SMD-M06-2X/def2-TZVPP optimized structures of model chloronium ions. Bond distances and pyramidalization (*Δ*) in Å, with NCI isosurface shown for the chloretanium structures.

A recent study by Stoyanov and Reed, in which X-ray structures of dimethyl and diethyl chloronium salts were obtained,^33^ allowed us to compare computed and experimentally determined C–Cl bond lengths. The experimental C–Cl length in the diethylchloronium ion is 1.840 Å. A survey of different methods and basis sets (BS1 = def2-TZVPP, BS2 = def2-QZVPP) found that B3LYP, B3LYP-D3, B2PLYP overestimate this distance. However, ωB97XD and the Minnesota functionals provide a good description of the chloronium structure. The MP2/BS1 and M11-L/BS2 derived bond lengths of 1.84 Å were in excellent agreement with experiment. Smaller basis sets, such as 6-31+G(d) gave routinely worse results (see ESI†). We next examined the unsymmetrical allyl/propyl chloronium ion using the same theoretical models (Table 1). This model compound allows for a comparison of C–Cl bond lengths, and, therefore, to establish the extent of chloronium *versus* carbocationic character in these structures. All of the theoretical methods tested show that the allylic group interacts more weakly than the alkyl group, based on a longer C–Cl bond distance. The computed variation between alkyl and allyl distances ranges from 0.04–0.13 Å. The M11-L/def2-TZVPP (entry 6) optimized structure is an outlier, consistent with a carbocation (see Fig. 1A) with a *r*(C–allyl) distance 2.54 Å and bond lengths of 1.35 Å and 1.39 Å in the allylic fragment. However, all other methods generated a chloronium like structure. At all levels the calculated dissociation energy of **40** into allyl cation and propylchloride fragments (Δ*E*, Table 1) is positive by at least 8 kcal mol^–1^, signifying a stabilizing interaction between the chlorine and the allylic carbon atom even in the structures with longer (C–allyl) distances.

We returned to the ring opening reactions of epoxides **7a** and **7b** (Scheme 10). Taking epoxide **7a** first, we optimized the SMD-M06-2X/def-TZVPP geometries for truncated analogues of the two putative chloronium ion intermediates, chloretanium **1** and chlorolanium **1** (Fig. 1A) derived from anchimeric assistance of either the γ- or δ-chlorine substituents, respectively. Inspection of the geometry of chloretanium **1** reveals: (a) a long Cl-allyl distance of 2.69 Å; (b) similar bond lengths of 1.39 Å and 1.36 Å within the allylic fragment; and (c) planar allylic carbon atoms, as quantified by the extent of pyramidalization. These data are consistent with a distorted chloretanium ion with significant carbocationic character. In contrast, chlorolanium **1** has a substantially shorter carbon–chlorine bond length (1.90 Å) and substantial pyramidalization of the allylic carbon, consistent with a cyclic chloronium ion. The same pattern was seen with chloretanium **2** and chlorolanium **2**, which arise from the diastereoisomeric epoxide **7b**. The relative energy of the carbocation-like chloretanium structures was, in each case, significantly higher by more than 8 kcal mol^–1^ compared to the corresponding chlorolanium ion (Fig. 1).

While chloretanium intermediates were found to be less stable, with greater carbocationic character than the chlorolanium species, there is still an interaction between Cl and the allylic carbon. The C–Cl distance in these unsymmetrical intermediates (2.69–2.70 Å) is considerably shorter than the sum of van der Waals radii of 3.45 Å (Bondi) or 3.52 Å (Pauling). The presence of a through-space, weakly attractive C–Cl interaction in the chloretanium is also apparent from a NBO-computed Wiberg bond order^34^ of 0.06, and the Non-Covalent Interaction (NCI) isosurface shown in Fig. 1.^35^ This interaction is much weaker than the corresponding bond order obtained for the chlorolanium species (0.89–0.92), and a fairly small barrier of 3.5 kcal mol^–1^ to rotate about the C13–C14 bond was found for chloretanium **1**. It is fair to summarise that the four and five-membered chloronium intermediates lie along a continuum from free carbocation to chloronium-like Lewis structures, rather than an abrupt shift in the nature of bonding.

With these computational data in mind, we return to the stereoretentive titanium-mediated epoxide ring opening reactions depicted in Scheme 10. We suggest that these reactions take place through a pair of diastereoisomeric chlorolanium-like intermediates (chlorolanium **1** and chlorolanium **2**, Scheme 10) rather than the higher energy distorted chloretanium ions. Based upon the computed geometries of the truncated analogues the chlorolanium-like intermediates are likely to have unequal carbon–chlorine bond distances and exhibit a degree of carbocationic character at C13. We note that the epoxide opening of model compound **19** (Scheme 4B), which contained an electron withdrawing ester group took place with inversion of stereochemistry. This result corroborates the development of partial cationic character at C13 during reactions that take place with retention of configuration. Finally, it should be noted that high levels of stereoretention have been observed in systems in which the formation of a chlorolanium ion is not possible *e.g.* δ-chloro vinyl epoxides.^30^ In such cases it is likely that distorted chloretanium ions are involved and that the chlorine substituent blocks one face of the allylic subunit leading to high levels of stereoretention.

## Conclusion

In summary, we have accomplished a 12-step convergent, enantioselective formal synthesis of malhamensilipin A from commercial (*E*)-2-undecenal. The synthesis strategy was based upon an iterative epoxidation/deoxydichlorination sequence, which demonstrates the power of phosphorus(v)-mediated deoxydichlorination reactions of epoxides for the stereospecific and chemoselective construction of chlorine-containing stereochemical arrays. In addition to the formal synthesis, we have prepared three further diastereoisomeric heptachlorinated analogues and demonstrated the utility of Carreira's NMR database for the stereochemical assignment of complex polychlorinated entities. Our computational analysis has provided new insight into the stereoretentive ring-opening reactions of γ,δ-dichloro vinyl epoxides. Specifically, we have corroborated the intervention of chloronium ion intermediates both experimentally and computationally. Furthermore, the intermediate chloretanium ions have been shown to be highly distorted with pronounced carbocationic character, making them less stable than the chlorolaniums. Despite this, computations do support the existence of a weakly attractive C–Cl interaction in the four-membered intermediate. The structures of both cyclic intermediates exist along a continuum of Lewis structures from free carbocation to chloronium, such that anchimeric assistance can occur in both cases.

## References

[cit1] Gribble G. W. (2010). Prog. Chem. Org. Nat. Prod..

[cit2] Bedke D. K., Vanderwal C. D. (2011). Nat. Prod. Rep..

[cit3] Elovson J., Vagelos P. R. (1969). Proc. Natl. Acad. Sci. U. S. A..

[cit4] Ciminiello P., Fattorusso E., Forino M., Di Rosa M., Ianaro A., Poletti R. (2001). J. Org. Chem..

[cit5] Nilewski C., Geisser R. W., Carreira E. M. (2009). Nature.

[cit6] Yoshimitsu T., Fukumoto N., Nakatani R., Kojima N., Tanaka T. (2010). J. Org. Chem..

[cit7] Bedke D. K., Shibuya G. M., Pereira A., Gerwick W. H., Haines T. H., Vanderwal C. D. (2009). J. Am. Chem. Soc..

[cit8] Bedke D. K., Shibuya G. M., Pereira A. R., Gerwick W. H., Vanderwal C. D. (2010). J. Am. Chem. Soc..

[cit9] Nilewski C., Deprez N. R., Fessard T. C., Li D. B., Geisser R. W., Carreira E. M. (2011). Angew. Chem., Int. Ed..

[cit10] Shibuya G. M., Kanady J. S., Vanderwal C. D. (2008). J. Am. Chem. Soc..

[cit11] Landry M. L., Hu D. X., McKenna G. M., Burns N. Z. (2016). J. Am. Chem. Soc..

[cit12] Snyder S. A., Tang Z.-Y., Gupta R. (2009). J. Am. Chem. Soc..

[cit13] Nicolaou K. C., Simmons N.-L., Ying Y., Heretsch P. M., Chen J. S. (2011). J. Am. Chem. Soc..

[cit14] Cresswell A. J., Eey S. T.-C., Denmark S. E. (2015). Nat. Chem..

[cit15] Yoshimitsu T., Fukumoto N., Tanaka T. (2009). J. Org. Chem..

[cit16] (b) Marsden in S. P., Dunn P. J.; Hii K. K.; Krische M. J. and Williams M. T., Sustainable Catalysis: Challenges and Practices for the Pharmaceutical and Fine Chemical Industries, Wiley, New York, 1st edn, 2013, p. 440.

[cit17] Nilewski C., Geisser R. W., Ebert M.-O., Carreira E. M. (2009). J. Am. Chem. Soc..

[cit18] Zhao G. L., Ibrahem I., Sunden H., Cordova A. (2007). Adv. Synth. Catal..

[cit19] Marigo M., Franzen J., Poulsen T. B., Zhuang W., Jorgensen K. A. (2005). J. Am. Chem. Soc..

[cit20] The competing elimination has been observed in related reactions by us (ref. 16) as well as Tanaka and Yoshimitsu (ref. 15a)

[cit21] Epoxide **9a** had been previously described by Vanderwal and co-workers during the first total synthesis of malhamensilipin A, see ref. 8

[cit22] Blakemore P. R., Cole W. J., Kocienski P. J., Morley A. (1998). Synlett.

[cit23] Full details can be found in the ESI.

[cit24] Liu P., Jacobsen E. N. (2001). J. Am. Chem. Soc..

[cit25] Pospisil J. (2011). Tetrahedron Lett..

[cit26] The designation of “fused” and “spiro” mode cyclizations in place of *endo* and *exo* has been used to describe ring-opening reactions of cyclopropanes and, more recently, to describe epoxide opening reactions, see Vilotijevic I. Jamison T. F., Science, 2007, 317 , 1189 –1192 , . This terminology avoids confusion since the carbon–oxygen bond breaks outside the ring in both the *endo* and *exo* ring opening reactions .17761875

[cit27] Nicolaou K. C., Prasad C. V. C., Somers P. K., Hwang C.-K. (1989). J. Am. Chem. Soc..

[cit28] (e) Olah G. A., Laali K. K., Wang Q. and Prakash G. K. S., Onium Ions, Wiley, New York, 1998, ch. 6.

[cit29] Peterson P. E., Bopp R. J., Dhansukh M. C., Curran E. L., Dillard D., Kamat R. J. (1967). J. Am. Chem. Soc..

[cit30] Shemet A., Sarlah D., Carreira E. M. (2015). Org. Lett..

[cit31] Frisch M. J., et al., Quantum chemical calculations were performed with Gaussian09: Gaussian 09, Revision D.01, Gaussian, Inc., Wallingford CT, 2009.

[cit32] Marenich A. V., Cramer C. J., Truhlar D. G. (2009). J. Phys. Chem. B.

[cit33] Stoyanov E. S., Stoyanova I. V., Tham F. S., Reed C. A. (2010). J. Am. Chem. Soc..

[cit34] Glendening E. D., Badenhoop J. K., Reed A. E., Carpenter J. E., Bohmann J. A., Morales C. M., Landis C. R. and Weinhold F., NBO 6.0., Theoretical Chemistry Institute, University of Wisconsin, Madison, 201.

[cit35] Contreras-Garcia J., Johnson E. R., Keinan S., Chaudret R., Piquemal J.-P., Beratan D., Yang W. (2011). J. Chem. Theory Comput..

