# Supplementary material for: Synthesis of malhamensilipin A exploiting iterative epoxidation/chlorination: experimental and computational analysis of epoxide-derived chloronium ions
Source: Chem Sci. 2016 Aug 2;7(12):7040–9. doi: 10.1039/c6sc03012b (PMC5282550; doi:10.1039/c6sc03012b)
Supplement: Supplementary file 1 [file SC-007-C6SC03012B-s001.pdf]

Electronic Supplementary Material (ESI) for Chemical Science.
This journal is © The Royal Society of Chemistry 2016

Synthesis of Malhamensilipin A Exploiting Iterative Epoxidation/Chlorination: Experimental and Computational Analysis of Epoxide-Derived Chloronium Ions

Jan Saska[a], William Lewis,[a] Robert Paton*[b] and Ross M. Denton*[a]

[a]*School of Chemistry, University of Nottingham, University Park, NG7 2RD*

[b]*Chemistry Research Laboratory, Mansfield Road, Oxford OX1 3TA, UK*

SUPPORTING INFORMATION

Table of Contents

I. General experimental procedures **S2**

II. Experimental Procedures and Characterization of Novel Compounds **S3**

II.A Synthesis of the C12–C24 fragments S3

II.B Opening of the C13-C14 epoxide – model system S8

II.C Synthesis of the C1–C11 fragments S9

II.D Julia-Kocienski olefination S12

II.E Opening of the C13-C14 epoxide S14

II.F Synthesis Completion S15

III. NMR Spectra **S22**

IV. X-Ray Crystallography Data **S82**

V. Computational Data **S84**

I. General experimental procedures

Unless stated otherwise, all reactions were carried out under an atmosphere of nitrogen. All glassware was flame dried or oven dried and allowed to cool under a stream of nitrogen before use. Cooling to 0 °C was effected using an ice-water bath. Cooling to temperatures below 0 °C was effected using dry ice-acetone mixtures. Degassing was achieved by purging nitrogen through the appropriate solution for 15 min. The term petroleum ether refers to the fraction with boiling point between 40 and 60 °C.

Commercially available solvents and reagents were used as supplied with the following exceptions. Toluene, THF and diethyl ether were collected from a solvent tower, where a degassed solvent was passed through two columns of activated alumina and a 7 micron filter under a 4 bar pressure, and stored under nitrogen atmosphere over sodium wire. Benzene, HMPA and CH_2Cl_2 were stirred with calcium hydride for 2 h, distilled and stored under a nitrogen atmosphere over activated 4 Å molecular sieves. Triflic anhydride was stirred with phosphorus pentoxide for 2 h, distilled, and stored under a nitrogen atmosphere;[1] titanium isopropoxide was distilled under vacuum and stored under a nitrogen atmosphere; 18-crown-6 was dried under vacuum at 60 °C for 16 h prior to use; commercially available *m*CPBA (3.00 g) was dissolved in Et_2O (20.0 mL) and washed with a pH 7.5 buffer solution (3 x 15.0 mL). The organic layer was dried ($MgSO_4$) and carefully concentrated *in vacuo* to give pure *m*CPBA (1.50 g). The pH 7.5 buffer solution was prepared by mixing 0.1M aqueous solution of NaOH (41.0 mL), 0.2M aqueous solution of KH_2PO_4 (25.0 mL) and water (34.0 mL).[2]

Reactions were monitored using thin layer chromatography on Merck TLC silica gel 60 F_{254} pre-coated aluminium sheets with fluorescent indicator. Sheets were visualised using ultra-violet light (254 nm) and/or $KMnO_4$ solution, as appropriate. Flash column chromatography was performed using Aldrich silica gel 60, 40–63 µm, unless otherwise noted.

Melting points were measured on a Stuart SMP 3 instrument. Optical rotations were measured on a BS ADP440 instrument as solutions of a known concentration in chloroform unless otherwise noted. Infrared spectra were recorded on a Bruker Tensor 27 instrument as dilute chloroform solutions. Mass spectra were acquired on a VG micromass 70E, VG Autospec or Micromass LCTOF.

Bruker DPX400, Bruker AV400, Bruker AV(III)400 or Bruker AV(III)500 spectrometers were used to record all ^{1}H and ^{13}C NMR spectra of dilute solutions of the appropriate compound in the indicated deuterated solvent. All chemical shifts (δ) are reported in parts per million (ppm) relative to residual solvent peaks. All chemical shifts are reported relative to chloroform (δ_H = 7.27 ppm, δ_C = 77.0 ppm), benzene (δ_H = 7.16 ppm, δ_C = 128.4 ppm), methanol (δ_H = 4.87 ppm, δ_C = 49.2 ppm) or acetone (δ_H = 2.05 ppm, δ_C = 29.9 ppm). Coupling constants (*J*) are reported in Hertz and are reported after averaging. The multiplicity of a ^{1}H NMR signal is designated by one of the following abbreviations: s = singlet, d = doublet, t = triplet, q = quartet, m = multiplet, br = broad signal. The "multiplicities" of ^{13}C NMR signals were assigned using a DEPT sequence. Where appropriate, assignments were aided using COSY, HMQC and HMBC spectra.

Mass spectra were acquired on a VG micromass 70E, VG Autospec or Micromass LCTOF.

[1] Link, A. J.; Vink, M. K. S.; Tirrell, D. A. *Nat. Protoc.*, **2007**, 2, 1879–1883.
[2] Aggarwal, V. K.; Gultekin, Z.; Grainger, R. S.; Adams, H.; Spargo, P. L. *J. Chem. Soc., Perkin. Trans. 1*, **1998**, 2771–2782.

II. Experimental Procedures and Characterisation of Novel Compounds

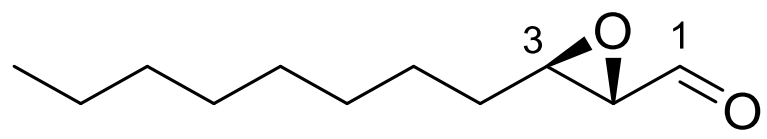

(*2S, 3R*)-2,3-Epoxyundecan-1-al (12)[3]

To a solution of (*S*)-(-)-α,α-diphenyl-2-pyrrolidinemethanoltrimethylsilyl ether (193 mg, 0.59 mmol) in $CHCl_3$ (50.0 mL) was added *E*-2-undecen-1-al (**11**) (1.18 mL, 5.94 mmol) and H_2O_2 (0.73 mL, 7.13 mmol of a 30% w/v aq. solution). The reaction mixture was stirred at r. t. for 3 hours. The reaction mixture was then diluted with H_2O (50.0 mL) and extracted with CH_2Cl_2 (3x50.0 mL). The combined organics were dried ($MgSO_4$) and concentrated *in vacuo*. Flash column chromatography (2:1, petroleum ether/Et_2O) gave **13** (996 mg, 91%, 94% *ee*) as a colourless oil.

The enantiomeric excess was measured by GC on Chromasil CP-Chirasil-Dex CB column. Temperature program: 70 °C to 160 °C, rate: 10 °C/min, hold 1 min, 160 °C to 200 °C, rate: 80 °C/min, hold 5 min. R_t (min) = major enantiomer 14.6 min, minor enantiomer 15.1 min.

$[\alpha]_D$ = −23.1 (*c* = 1.0, EtOH) [lit.[3] $[\alpha]_D$ = −21.4 (*c* = 1.0, EtOH)]; ^{1}H NMR (400MHz, $CDCl_3$) δ 9.02 (d, *J* = 6.3, 1H, H-1), 3.23 (ddd, *J* = 5.9, 5.1, 1.9, 1H, H-3), 3.14 (dd, *J* = 6.3, 1.9, 1H, H-2), 1.70–1.63 (m, 2H, H-4), 1.37–1.28 (m, 12H), 0.89 (t, *J* = 6.9, 3H, H-11); ^{13}C NMR (101 MHz, $CDCl_3$) δ 198.6 (C(O)H), 59.3 (CH), 56.9 (CH), 31.9 (CH_2), 31.3 (CH_2), 29.5 (CH_2), 29.3 (CH_2), 29.2 (CH_2), 25.9 (CH_2), 22.7 (CH_2), 14.2 (CH_3); IR ($CHCl_3$) ν_{max} 2929, 2857, 1728, 1466, 1251, 851 cm^{-1}; HRMS (ESI$^+$) $C_{11}H_{20}O_2Na^+$ $[M+Na]^+$ *m/z* calcd. 184.1463, found 184.1471.

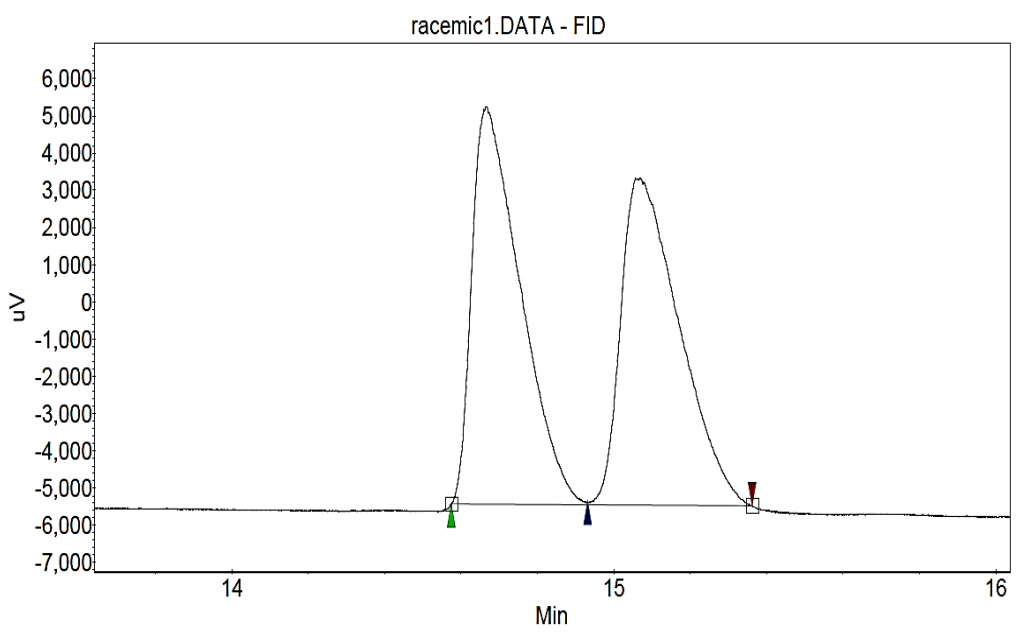

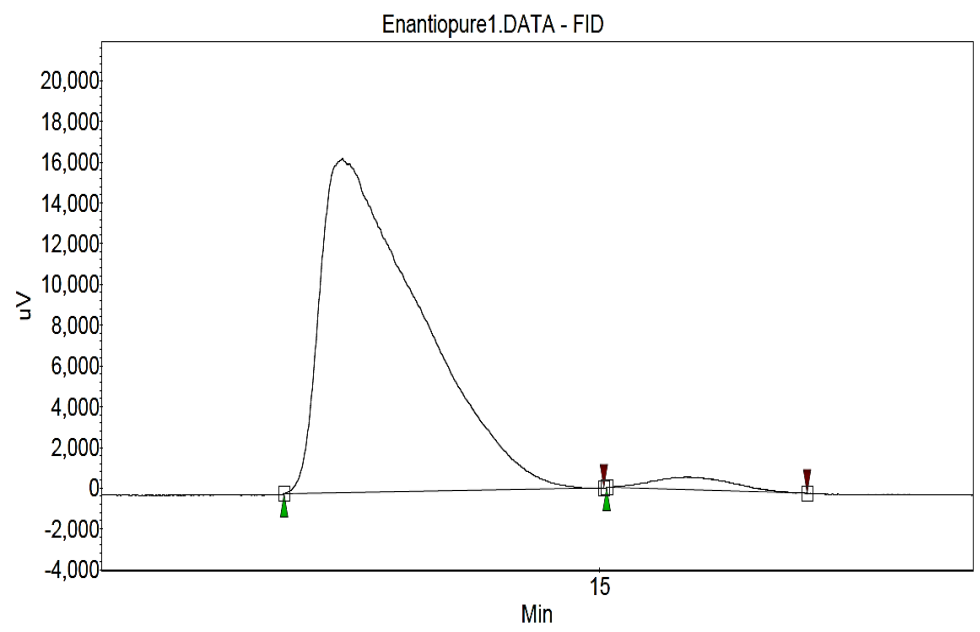

Peak results :

Index	Name	Time [Min]	Quantity [% Area]	Height [uV]	Area [uV.Min]	Area % [%]
1	UNKNOWN	14.67	50.07	10682.1	1566.7	50.068
2	UNKNOWN	15.07	49.93	8786.5	1562.5	49.932
Total			100.00	19468.6	3129.2	100.000

Peak results :

Index	Name	Time [Min]	Quantity [% Area]	Height [uV]	Area [uV.Min]	Area % [%]
1	UNKNOWN	14.60	96.96	16373.5	2949.9	96.960
2	UNKNOWN	15.14	3.04	626.3	92.5	3.040
Total			100.00	16999.9	3042.4	100.000

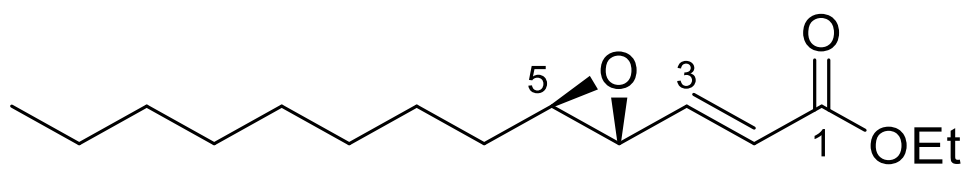

Ethyl (4*R*,5*R*,*E*)-4,5-epoxytridec-2-enoate (10)

To a suspension of sodium hydride (788 mg, 19.7 mmol of a 60% dispersion in mineral oil) in THF (90 mL) at 0 °C was added triethyl phosphonoacetate (3.91 mL, 19.7 mmol) and the reaction mixture was stirred at r. t. for 1 hour. The reaction mixture was then cooled to −78

[3] Matsuo, J.; Iida, D.; Yamanaka, H.; Mukaiyama, T. *Tetrahedron*, **2003**, 59, 6739–6750.

°C, a solution of aldehyde **12** (2.42 g, 13.1 mmol) in THF (40.0 mL) was added, and the reaction mixture was stirred at −78 °C for 30 min and then at r. t. for further 1 hour. The reaction mixture was then diluted with Et_2O (100 mL), washed with H_2O (3x150 mL), dried ($MgSO_4$) and concentrated *in vacuo*. Flash column chromatography (2:1, petroleum ether/Et_2O) gave **10** (3.01 g, 92%) as a colourless oil.

$[\alpha]_D$ = 1.3 (*c* = 1.0, $CHCl_3$); ^1H NMR (400 MHz, $CDCl_3$) δ 6.68 (dd, *J* = 15.7, 7.2, 1H, H-3), 6.12 (d, *J* = 15.7, 1H, H-2), 4.21 (q, *J* = 7.1, 1H, $RCO_2CH_2CH_3$), 3.21 (dd, *J* = 7.2, 2.0, 1H, H-4), 2.89 (ddd, *J* = 5.6, 5.6, 2.0, 1H, H-5), 1.64–1.56 (m, 2H, H-6), 1.37–1.25 (m, 15H), 0.89 (t, *J* = 6.9, 3H, H-13); ^{13}C NMR (101 MHz, $CDCl_3$) δ 165.8 (C), 144.9 (CH), 123.6 (CH), 61.6 (CH), 60.7 (CH_2), 56.4 (CH), 32.0 (CH_2), 31.9 (CH_2), 29.5 (CH_2), 29.4 (CH_2), 29.3 (CH_2), 25.9 (CH_2), 22.7 (CH_2), 14.3 (CH_3), 14.2 (CH_3); IR ($CHCl_3$) ν_{max} 2929, 2857, 1714, 1657, 1466, 1393, 1260, 852 cm^{-1}; HRMS (ESI^+) $C_{15}H_{26}O_3Na^+$ $[M+Na]^+$ *m/z* calcd. 277.1774, found 277.1772.

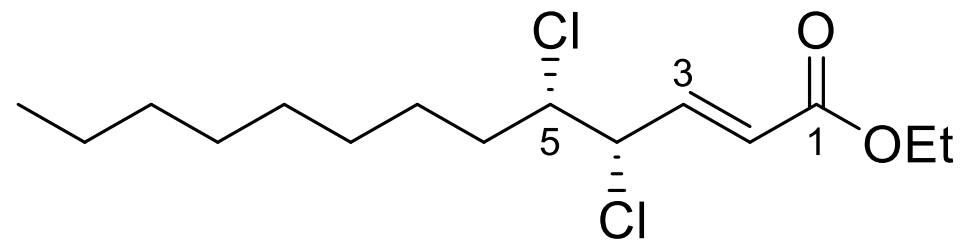

Ethyl (*4S,5S,E*)-4,5-dichlorotridec-2-enoate (13)

To a solution of triphenylphosphine oxide (367 mg, 1.32 mmol) in toluene (10.0 mL) was added oxalyl chloride (0.11 mL, 1.32 mmol) and the reaction mixture was stirred at r. t. for 15 minutes. Solution of epoxide **10** (2.25 g, 8.85 mmol) in toluene (10.0 mL) and a solution of oxalyl chloride (0.87 mL, 10.2 mmol) in toluene (10.0 mL) were added separately but simultaneously *via* syringe pump over 6 hours at 90 °C. The reaction mixture was then cooled to r. t. and concentrated *in vacuo*. Flash column chromatography (3:2, petroleum ether/CH_2Cl_2) gave **13** (1.40 g, 51%) and **14** (1.09 g, 45%) as colourless oils.

$[\alpha]_D$ = −25.3 (*c* = 1.0, $CHCl_3$); ^1H NMR (400 MHz, $CDCl_3$) δ 7.00 (dd, *J* = 15.4, 7.2, 1 H, H-3), 6.15 (dd, *J* = 15.4, 1.2, 1 H, H-2), 4.71 (ddd, *J* = 7.2, 3.7, 1.2, 1 H, H-4), 4.24 (q, *J* = 7.2, 2 H, $RCO_2C\underline{H}_2CH_3$), 4.09 (ddd, *J* = 10.0, 10.0, 3.7, 1 H, H-5), 2.02–1.92 (m, 1 H, H-6), 1.75–1.64 (m, 1 H, H-6), 1.48–1.03 (m, 15 H), 0.89 (t, *J* = 6.9, 3 H, H-13); ^{13}C NMR (101 MHz, $CDCl_3$) δ 165.5 (C), 141.7 (CH), 125.4 (CH), 64.2 (CH), 62.6 (CH), 61.0 (CH_2), 33.7 (CH_2), 31.9 (CH_2), 29.4 (CH_2), 29.3 (CH_2), 29.0 (CH_2), 26.6 (CH_2), 22.7 (CH_2), 14.3 (CH_3), 14.2 (CH_3); IR ($CHCl_3$) ν_{max} 2928, 2857, 1717, 1466, 1279, 1178, 909 cm^{-1}; HRMS (ESI^+) $C_{15}H_{26}{}^{35}Cl_2O_2Na^+$ $[M+Na]^+$ *m/z* calcd. 331.1202, found 331.1213.

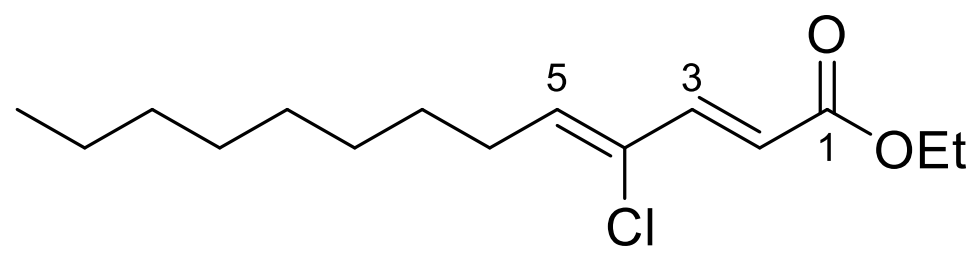

Ethyl (2*E*,4*Z*)-4-chlorotrideca-2,4-dienoate (14)

^1H NMR (400 MHz, $CDCl_3$) δ 7.61 (d, *J* = 14.8, 1 H, H-3), 6.29 (d, *J* = 14.8, 1 H, H-2), 6.12 (t, *J* = 8.2, 1 H, H-5), 4.23 (q, *J* = 7.2, 2 H, $RCO_2C\underline{H}_2CH_3$), 2.37–2.26 (m, 2 H), 1.50–1.38 (m, 2 H), 1.36–1.19 (m, 13 H), 0.87 (t, *J* = 6.7, 3 H, H-13); ^{13}C NMR (101 MHz, $CDCl_3$) δ 166.7 (C), 139.7 (CH), 135.8 (CH), 128.4 (C), 122.1 (CH), 60.6 (CH_2), 31.8 (CH_2), 29.3 (CH_2), 29.1 (CH_2), 29.1 (CH_2), 29.0 (CH_2), 28.9 (CH_2), 22.6 (CH_2), 14.2 (CH_3), 14.0 (CH_3); IR ($CHCl_3$) ν_{max} 2958, 2929, 2857, 1709, 1631, 1466, 1306, 1269, 1183, 1034, 964 cm^{-1}; HRMS (ESI^+) $C_{15}H_{25}{}^{35}ClO_2Na^+$ $[M+Na]^+$ *m/z* calcd. 295.1435, found 295.1440.

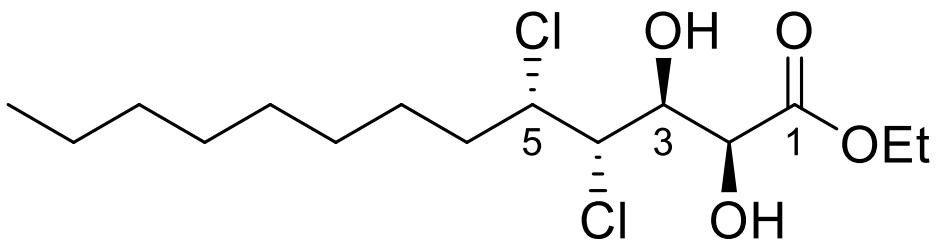

(*2S,3S,4S,5S*)-Ethyl 4,5-dichloro-2,3-dihydroxytridecanoate (15)

To a solution of dichloride **13** (0.91 g, 2.94 mmol) in a 2:1 acetone/water mixture (30.0 mL) at 0 °C was added osmium tetroxide (0.90 mL, 0.15 mmol of a 4% w/v solution in H_2O) and NMO (0.67 mL, 3.23 mmol of a 50% w/v solution in H_2O) and the reaction mixture was stirred at r. t. for 16 hours. The reaction was then quenched with sodium bisulfite (30.0 mL of 20% w/v aq. solution) and, after a further 3 hours stirring, was extracted with CH_2Cl_2 (3x50.0 mL). The combined organics were dried ($MgSO_4$) and concentrated *in vacuo*. Flash column chromatography (3:1, petroleum ether/EtOAc) gave **15** (823 mg, 81%) as a colourless solid. Recrystallisation from minimal amount of CH_2Cl_2/pentane gave analytically pure sample of **15** as colourless needles.

$[\alpha]_D = -25.3$ (c = 1.0, $CHCl_3$); ^{1}H NMR (400 MHz, $CDCl_3$) δ 4.68 (dd, J = 4.3, 0.9, 1 H, H-2), 4.53 (ddd, J = 8.8, 5.4, 1.8, 1 H, H-5), 4.40–4.27 (m, 2 H, $RCO_2C\underline{H_2}CH_3$), 4.24 (dd, J = 9.6, 0.9, 1 H, H-3), 4.15 (dd, J = 9.6, 1.8, 1 H, H-4), 3.18 (d, J = 4.3, 1 H, RO$\underline{H}$), 2.36 (d, J = 9.8, 1 H, RO$\underline{H}$), 2.07–1.96 (m, 1 H, H-6), 1.87–1.78 (m, 1 H, H-6), 1.46–1.03 (m, 15 H), 0.89 (t, J = 6.9, 3 H, H-13); ^{13}C NMR (101 MHz, $CDCl_3$) δ 173.2 (C), 73.4 (CH), 70.1 (CH), 63.1 (CH), 62.8 (CH_2), 61.7 (CH), 36.4 (CH_2), 31.9 (CH_2), 29.5 (CH_2), 29.3 (CH_2), 29.1 (CH_2), 26.6 (CH_2), 22.7 (CH_2), 14.2 (CH_3), 14.2 (CH_3); IR ($CHCl_3$) ν_{max} 3605, 3544, 2928, 2857, 1734, 1466, 1269, 1113, 1058, 909 cm^{-1}; HRMS (ESI$^+$) $C_{15}H_{28}{}^{35}Cl_2O_4Na^+$ $[M+Na]^+$ *m/z* calcd. 365.1262, found 365.1244; m. p. 67–69 °C.

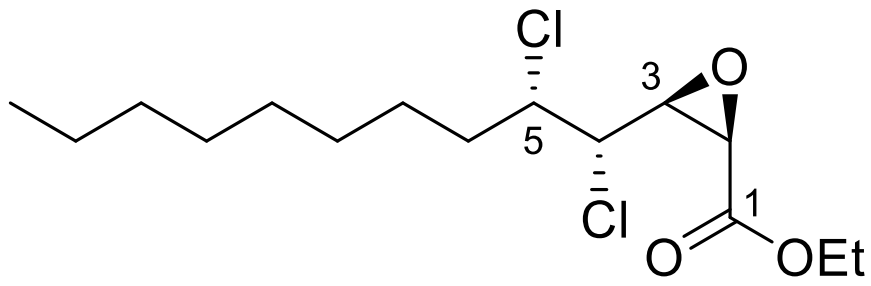

Ethyl ((*2R,3S,4S,5S*)-4,5-dichloro-2,3-epoxytridecanoate (16)[4]

To a solution of DABCO (1.20 g, 10.7 mmol) in CH_2Cl_2 (11.0 mL) at −78 °C was added triflic anhydride (0.60 mL, 3.57 mmol) dropwise over 30 seconds and the reaction mixture was stirred for 10 min. A solution of diol **15** (612 mg, 1.78 mmol) in CH_2Cl_2 (6.00 mL) was precooled to −78 °C and added to the reaction mixture *via* a cannula. The reaction mixture was warmed to r. t. over 16 hours and then quenched with sat. aq. $NaHCO_3$ (20.0 mL). The aqueous layer was extracted with CH_2Cl_2 (3x20.0 mL). The combined organics were dried ($MgSO_4$) and concentrated *in vacuo*. Flash column chromatography (1:1, CH_2Cl_2/petroleum ether) gave **16** (410 mg, 71%) as a colourless oil.

$[\alpha]_D = -61.2$ (c = 1.0, CH_2Cl_2) [lit.[4] $[\alpha]_D = -65.8$ (c = 1.18, CH_2Cl_2)]; ^{1}H NMR (400 MHz, C_6D_6) δ 4.46 (dd, J = 8.6, 2.5, 1 H, H-4), 4.21 (ddd, J = 8.9, 5.0, 2.5, 1 H, H-5), 3.93−3.80 (m, 2 H, $RCO_2C\underline{H_2}CH_3$), 3.51 (dd, J = 8.6, 4.3, 1 H, H-3), 3.19 (d, J = 4.3, 1 H, H-2), 1.88–1.78 (m, 1 H, H-6), 1.58–1.48 (m, 1 H, H-6), 1.40–1.00 (m, 12 H), 0.91 (t, J = 7.2, 3 H, $RCO_2CH_2C\underline{H_3}$), 0.86 (t, J = 7.1, 3 H, H-13); ^{13}C NMR (101 MHz, C_6D_6) δ 167.8 (C), 64.1 (CH), 62.1 (CH_2), 60.2 (CH), 58.0 (CH), 52.9 (CH), 36.6 (CH_2), 32.5 (CH_2), 30.0 (CH_2), 29.9 (CH_2), 29.5 (CH_2), 26.9 (CH_2), 23.4 (CH_2), 14.7 (CH_3), 14.3 (CH_3); IR ($CHCl_3$) ν_{max} 2928,

[4] Bedke, D. K.; Shibuya, G. M.; Pereira, A. R.; Gerwick, W. H.; Vanderwal, C. D. *J. Am. Chem. Soc.*, **2010**, 132, 2542–2543.

2857, 1744, 1466, 1382, 1017 cm^{-1}; HRMS (ESI^+) $C_{15}H_{26}{}^{35}Cl_2O_3Na^+$ $[M+Na]^+$ *m/z* calcd. 347.1151, found 347.1164.

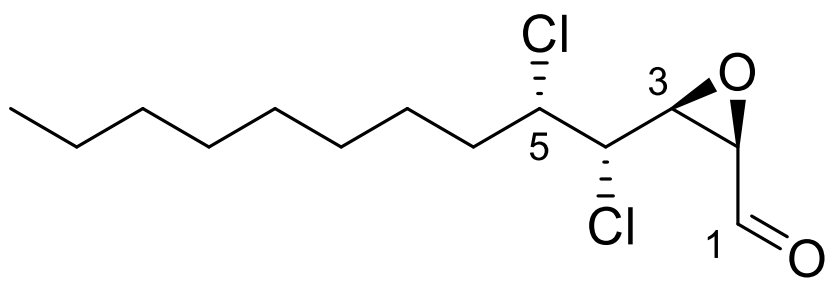

(*2R,3S,4S,5S*)-4,5-Dichloro-2,3-epoxytridecan-1-al (9a)[4]

To a solution of ester **16** (350 mg, 1.08 mmol) in toluene (11.0 mL) at −78 °C was added DIBAL (1.61 mL, 1.61 mmol of 1.0 M solution in hexanes) over 30 seconds and the reaction mixture was stirred for 1 hour. Saturated aqueous potassium sodium tartrate (10.0 mL) and Et_2O (10.0 mL) were added to the reaction mixture and stirred at r. t. overnight. The aqueous layer was extracted with Et_2O (3x10.0 mL). The combined organics were dried ($MgSO_4$) and concentrated *in vacuo*. Flash column chromatography (1:1, CH_2Cl_2/petroleum ether) gave **9a** (286 mg, 94%) as a colourless solid.

$[\alpha]_D$ = −34.2 (*c* = 1.1, CH_2Cl_2) [lit.[4] $[\alpha]_D$ = −35.3 (*c* = 0.25, CH_2Cl_2)]; ^1H NMR (400 MHz, C_6D_6) δ 8.91 (d, *J* = 2.1, 1 H, H-1), 4.05 (ddd, *J* = 9.4, 4.5, 2.6, 1 H, H-5), 3.72 (dd, *J* = 8.7, 2.6, 1 H, H-4), 3.41 (dd, *J* = 8.7, 4.5, 1 H, H-3), 2.81 (dd, *J* = 4.5, 2.1, 1 H, H-2), 1.80–1.70 (m, 1 H, H-6), 1.41–1.02 (m, 13 H), 0.92 (t, *J* = 7.1, 3 H, H-13); ^{13}C NMR (101 MHz, C_6D_6) δ 195.5 (CH), 63.9 (CH), 60.0 (CH), 59.7 (CH), 57.4 (CH), 36.3 (CH_2), 32.5 (CH_2), 30.0 (CH_2), 29.9 (CH_2), 29.5 (CH_2), 26.8 (CH_2), 23.4 (CH_2), 14.7 (CH_3); IR ($CHCl_3$) ν_{max} 2928, 2856, 1731, 1466, 1386, 1265, 1148 cm^{-1}; HRMS (ESI^+) $C_{13}H_{22}{}^{35}Cl_2O_2Na^+$ $[M+Na]^+$ *m/z* calcd. 335.0889, found 335.0906; m. p. 59–61 °C (lit.[4] m. p. 58–60 °C).

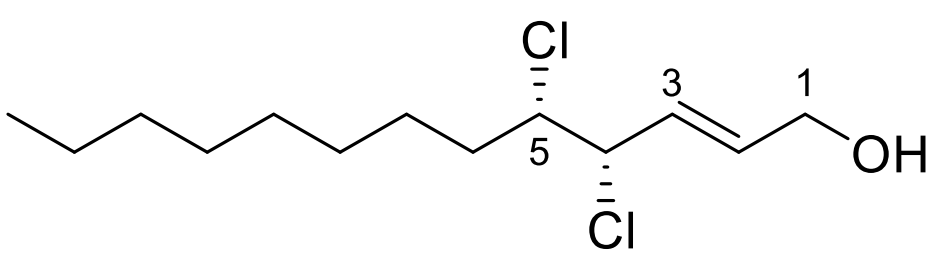

(4*S*,5*S*,*E*)-4,5-Dichlorotridec-2-en-1-ol (17)

To a solution of unsaturated ester **13** (140 mg, 0.45 mmol) in toluene (4.50 mL) at 0 °C was added DIBAL (1.36 mL, 1.36 mmol of 1.0 M solution in toluene) and the reaction mixture was stirred at 0 °C for 15 min. A saturated aqueous solution of potassium sodium tartrate (7.00 mL) and Et_2O (5.00 mL) were added and the reaction mixture was stirred at r. t. for 16 h. The reaction mixture was extracted with Et_2O (3x7.00 mL) and the combined organics were dried ($MgSO_4$) and concentrated *in vacuo*. Flash column chromatography (1:1 petroleum ether/Et_2O) gave **17** (107 mg, 89%) as a colourless oil.

$[\alpha]_D$ = −25.4 (*c* = 0.3, $CHCl_3$); ^1H NMR (400 MHz, C_6D_6) δ 5.85 (ddt, *J* = 15.3, 8.4, 1.8, 1 H, H-3), 5.56 (dtd, *J* = 15.3, 4.7, 0.7, 1 H, H-2), 4.35 (ddd, *J* = 8.4, 3.6, 0.7, 1 H, H-4), 3.82 (ddd, *J* = 9.9, 3.6, 3.6, 1 H, H-5), 3.68 (br, 2 H, H-1), 1.85–1.76 (m, 1 H), 1.73–1.62 (m, 1 H), 1.56–1.45 (m, 1 H), 1.33–1.08 (m, 12 H), 0.92 (t, *J*=7.0 Hz, 3 H, H-13); ^{13}C NMR (101 MHz, C_6D_6) δ 135.6 (CH), 126.6 (CH), 66.1 (CH), 65.6 (CH), 62.3 (CH_2), 34.6 (CH_2), 32.6 (CH_2), 30.1 (CH_2), 30.0 (CH_2), 29.6 (CH_2), 27.2 (CH_2), 23.4 (CH_2), 14.7 (CH_3); IR ($CHCl_3$) ν_{max} 3611, 2928, 2857, 1466, 1380, 1261, 1089, 971 cm^{-1}; HRMS (ESI^+) $C_{13}H_{24}{}^{35}Cl_2NaO^+$ $[M+Na]^+$ *m/z* calcd. 289.1096, found 289.1097.

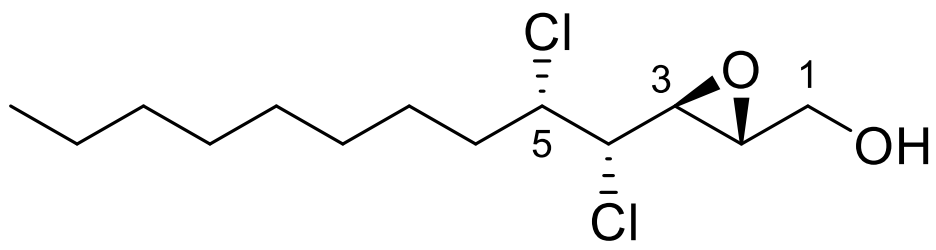

(2*R*,3*S*,4*S*,5*S*)-4,5-dichloro-2,3-epoxy-tridecan-1-ol (18)

To a suspension of powdered 4Å molecular sieves (60.0 mg) in CH_2Cl_2 (4.00 mL) at −20 °C was added (+)-diethyl-L-tartrate (0.48 mL, 0.48 mmol of 1.0 M solution in CH_2Cl_2) and Ti(O*i*-Pr)$_4$ (0.40 mL, 0.40 mmol of 1.0 M solution in CH_2Cl_2) and the reaction mixture was stirred for 30 min after which *t*-BuOOH (0.46 mL, 2.32 mmol of 5.0 M solution in decane) was added and the reaction mixture was stirred for further 30 min. A solution of allylic alcohol **17** (107 mg, 0.40 mmol) in CH_2Cl_2 (2.00 mL) was added and the reaction mixture was stirred at −20 °C for 16 h. A solution of citric acid (108 mg) and $FeSO_4$ (324 mg) in H_2O (3.00 mL) was added and the reaction mixture was stirred at r. t. for 1 h and then filtered through a pad of celite. The filtrate was extracted with EtOAc (3x5.00 mL). The combined organics were dried (Na_2SO_4) and concentrated *in vacuo*. Flash column chromatography (1:1 petroleum ether/Et_2O) gave **18** (72 mg, 64%) as a colourless oil.

$[\alpha]_D$ = −10.4 (*c* = 0.3, $CHCl_3$); ^{1}H NMR (400 MHz, C_6D_6) δ 4.04 (ddd, *J* = 9.2, 4.6, 2.0, 1 H, H-5), 3.37–3.30 (m, 3 H, H-1 + H-3 + H-4), 3.15 (ddd, *J* = 12.7, 6.7, 4.2, 1 H, H-1), 2.69 (ddd, *J* = 4.2, 2.6, 1.4, 1 H, H-2), 1.89–1.77 (m, 1 H), 1.58–1.49 (m, 1 H), 1.39–1.36 (m, 1 H), 1.34–1.06 (m, 12 H), 0.93 (t, *J* = 7.0 Hz, 3 H); ^{13}C NMR (101 MHz, C_6D_6) δ 64.8 (CH), 63.7 (CH), 61.3 (CH_2), 59.3 (CH), 56.0 (CH), 36.3 (CH_2), 32.6 (CH_2), 30.1 (CH_2), 29.9 (CH_2), 29.6 (CH_2), 27.0 (CH_2), 23.4 (CH_2), 14.7 (CH_3); IR ($CHCl_3$) ν_{max} 3604, 2957, 2929, 2857, 1465, 1379, 1261, 1081, 1021, 904 cm^{-1}; HRMS (ESI$^+$) $C_{13}H_{24}{}^{35}Cl_2NaO_2{}^+$ $[M+Na]^+$ *m/z* calcd. 305.1046, found 305.1053.

II.B Opening of the C13-C14 epoxide – model system

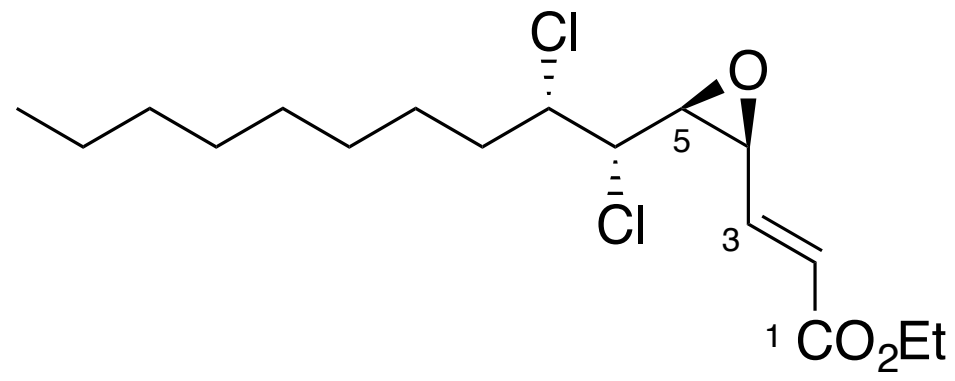

Ethyl (*4S,5S,6S,7S,E*)-6,7-dichloro-4,5-epoxypentadec-2-enoate (19)

To a suspension of sodium hydride (33.0 mg, 0.82 mmol of 60% dispersion in mineral oil) in THF (3.00 mL) at 0 °C was added triethyl phosphonoacetate (**17**) (0.16 mL, 0.82 mmol) and the reaction mixture was stirred at r. t. for 1 hour. The reaction mixture was then cooled to −78 °C and a solution of aldehyde **9a** (154 mg, 0.55 mmol) in THF (2.50 mL) was added. The reaction mixture was stirred at −78 °C for 30 min and then at r. t. for a further 1 h. The reaction mixture was then diluted with Et_2O (5.00 mL), washed with H_2O (3x5.00 mL), dried ($MgSO_4$) and concentrated *in vacuo*. Flash column chromatography (1:1, CH_2Cl_2/petroleum ether) gave **19** (164 mg, 85%) as a colourless oil.

$[\alpha]_D$ = −17.0 (*c* = 1.0, $CHCl_3$); ^{1}H NMR (400 MHz, C_6D_6) δ 6.85 (dd, *J* = 15.7, 5.7, 1 H, H-3), 6.09 (dd, *J* = 15.7, 1.1, 1 H, H-2), 4.07 (ddd, *J* = 9.5, 3.9, 2.5, 1 H, H-7), 3.95 (q, *J* = 7.1, 2 H, $RCO_2C\underline{H}_2CH_3$), 3.54 (dd, *J* = 9.1, 2.5, 1 H, H-6), 3.47 (dd, *J* = 9.1, 4.3, 1 H, H-5), 3.03 (ddd, *J* = 5.7, 4.3, 1.1, 1 H, H-4), 1.83–1.70 (m, 1 H, H-8), 1.39–1.02 (m, 13 H), 0.94–0.90 (m, 6 H, $RCO_2CH_2C\underline{H}_3$ + H-15); ^{13}C NMR (101 MHz, C_6D_6) δ 165.1 (C), 140.1 (CH), 127.0 (CH), 64.1 (CH), 61.0 (CH), 61.0 (CH_2), 59.9 (CH), 56.0 (CH), 36.2 (CH_2), 32.5 (CH_2), 30.0 (CH_2), 29.9 (CH_2), 29.6 (CH_2), 26.9 (CH_2), 23.4 (CH_2), 14.7 (CH_3), 14.5 (CH_3); IR ($CHCl_3$) ν_{max} 2927, 2855, 1734, 1457, 1192, 1029 cm^{-1}; HRMS (ESI$^+$) $C_{17}H_{28}{}^{35}Cl_2O_3Na^+$ $[M+Na]^+$ *m/z* calcd. 373.1308, found 373.1321.

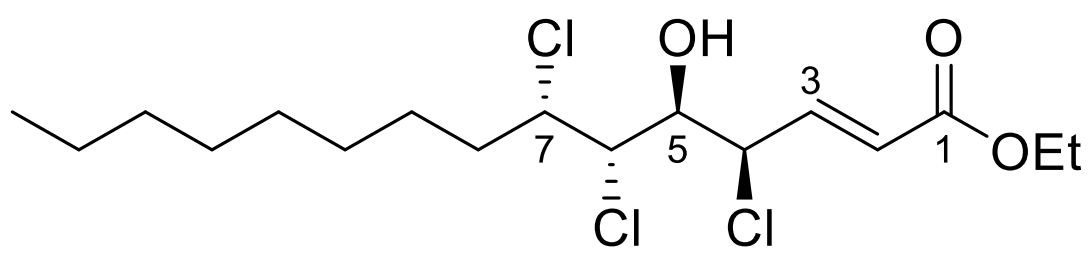

(*4R,5S,6S,7S,E*)-Ethyl-4,6,7-trichloro-5-hydroxypentadec-2-enoate (20)

To a solution of epoxide **19** (97.0 mg, 0.28 mmol) in Et_2O (1.00 mL) was added HCl (2.76 mL, 2.76 mmol of 1.0 M solution in Et_2O) at 0 °C and the reaction mixture was stirred at r. t. for 16 hours. The reaction mixture was concentrated *in vacuo*. Flash column chromatography (CH_2Cl_2) gave **20** (101 mg, 94%) as a colourless solid. Recrystallisation from minimal amount of CH_2Cl_2 gave an analytically pure sample of **20** as colourless needles.

$[\alpha]_D$ = 20.8 (*c* = 0.3, $CHCl_3$); ^{1}H NMR (400 MHz, C_6D_6) δ 6.93 (dd, *J* = 15.4, 7.2, 1 H, H-3), 5.97 (dd, *J* = 15.4, 1.4, 1 H, H-2), 4.92 (apparent dt, *J* = 7.1, 1.3, 1 H, H-4), 4.36 (ddd, *J* = 9.4, 4.5, 1.5, 1 H, H-7), 4.01 (q, *J* = 7.0, 2 H, $RCO_2C\underline{H}_2CH_3$), 4.00 (dd, *J* = 9.4, 1.6, 1 H, H-6), 3.92 (ddd, *J* = 9.5, 8.5, 1.4, 1 H, H-5), 1.99−1.87 (m, 1 H, H-8), 1.73 (d, *J* = 8.5, 1 H, $RO\underline{H}$), 1.47−1.10 (m, 13 H), 0.97 (t, *J* = 7.2, 3 H, $RCO_2CH_2C\underline{H}_3$), 0.92 (t, *J* = 7.1, 3 H, H-15); ^{13}C NMR (101 MHz, C_6D_6) δ 165.7 (C), 143.3 (CH), 125.5 (CH), 74.3 (CH), 65.3 (CH), 62.5 (CH), 62.3 (CH), 61.0 (CH_2), 37.0 (CH_2), 32.6 (CH_2), 30.1 (CH_2), 29.9 (CH_2), 29.7 (CH_2), 27.3 (CH_2), 23.4 (CH_2), 14.7 (CH_3), 14.5 (CH_3); IR ($CHCl_3$) ν_{max} 3601, 2928, 2855, 1718, 1465, 1371, 1266, 1178 cm^{-1}; HRMS (ESI$^+$) $C_{17}H_{29}{}^{35}Cl_3O_3Na^+$ $[M+Na]^+$ *m/z* calcd. 409.1074, found 409.1079; m. p. 70–72 °C; Elemental analysis $C_{17}H_{29}Cl_3O_3$ calcd. (%) C = 52.66, H = 7.54, found (%) C = 52.65, H = 7.55.

II.C Synthesis of the C_1–C_{11} fragment

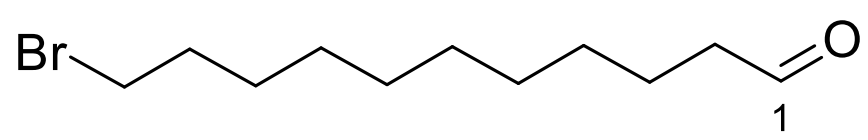

11-Bromoundecan-1-al (23)

To a solution of PCC (1.29 g, 5.97 mmol) in CH_2Cl_2 (20.0 mL) were added silica gel (1.29 g) and 11-bromoundecan-1-ol (**22**) (1.00 g, 3.98 mmol). Reaction mixture was stirred at r. t. for 16 h and filtered through a pad of celite. The filtrate was concentrated *in vacuo*. Flash column chromatography (4:1 petroleum ether/Et_2O) gave **23** (0.79 g, 80%) as a colourless oil.

^{1}H NMR (400 MHz, $CDCl_3$) δ 9.77 (t, J = 1.8, 1 H, H-1), 3.41 (t, J = 6.8, 2 H, H-11), 2.43 (td, J = 7.3, 1.9, 2 H, H-2), 1.90–1.82 (m, 2 H), 1.67–1.59 (m, 2 H), 1.47–1.37 (m, 2 H), 1.37–1.26 (m, 10 H); ^{13}C NMR (101 MHz, $CDCl_3$) δ 202.9 (CH), 43.9 (CH_2), 34.0 (CH_2), 32.8 (CH_2), 29.3 (CH_2), 29.3 (2x CH_2), 29.1 (CH_2), 28.7 (CH_2), 28.1 (CH_2), 22.0 (CH_2); IR ($CHCl_3$) ν_{max} 2930, 2857, 2281, 1709, 1463, 1286, 909 cm^{-1}; HRMS (ESI$^+$) $C_{11}H_{21}{}^{79}BrONa^+$ $[M+Na]^+$ *m/z* calcd. 271.0668, found 271.0663.

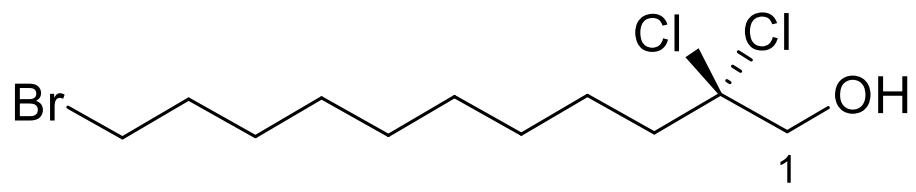

11-Bromo-2,2-dichloroundecan-1-ol (24)

To a solution of 11-bromoundecan-1-al (**23**) (0.68 g, 2.73 mmol) in 1,2-dichloroethane (13.5 mL) were added pyrrolidine (44.0 µL, 0.54 mmol) and NCS (0.91 g, 6.82 mmol) and the reaction mixture was stirred at 60 °C for 3 h. The reaction was quenched with sat. aq. Na_2SO_3 (15.0 mL) and extracted with EtOAc (3x15.0 mL). The combined organics were washed with sat. aq. $Na_2S_2O_3$ (4x50.0 mL), dried ($MgSO_4$) and concentrated *in vacuo* to give crude 10-bromo-2,2-dichlorodecanal (0.89 g, 99%) as a yellow oil.

To a solution of crude 10-bromo-2,2-dichlorodecanal (0.89 g, 2.73 mmol) in EtOH (4.00 mL) at 0 °C was added sodium borohydride (93.0 mg, 2.46 mmol) portionwise over 5 min. The reaction mixture was stirred at r. t. for 1 h, poured into conc. HCl (5.00 mL)/ ice mixture and exctracted with CH_2Cl_2 (3x10.0 mL). The combined organics were dried ($MgSO_4$) and concentrated *in vacuo*. Flash column chromatography (1:1, petroleum ether/Et_2O) gave **24** (0.81 g, 93% over 2 steps) as a colourless oil.

^{1}H NMR (400 MHz, $CDCl_3$) δ 3.91 (s, 2 H, H-1), 3.42 (t, J = 6.8, 2 H, H-11), 2.25–2.19 (m, 2 H), 1.91–1.82 (m, 2 H), 1.69–1.61 (m, 2 H), 1.49–1.29 (m, 11 H); ^{13}C NMR (101 MHz, $CDCl_3$) δ 94.7 (C), 72.1 (CH_2), 43.5 (CH_2), 34.0 (CH_2), 32.8 (CH_2), 29.2 (CH_2), 29.2(CH_2), 29.0 (CH_2), 28.7 (CH_2), 28.1 (CH_2), 24.8 (CH_2); IR ($CHCl_3$) ν_{max} 3590, 2931, 2857, 1464, 1065, 909 cm^{-1}; HRMS (ESI)$^+$ $C_{11}H_{21}{}^{79}Br^{35}Cl_2ONa^+$ $[M+Na]^+$ *m/z* calcd. 341.0045, found 341.0042.

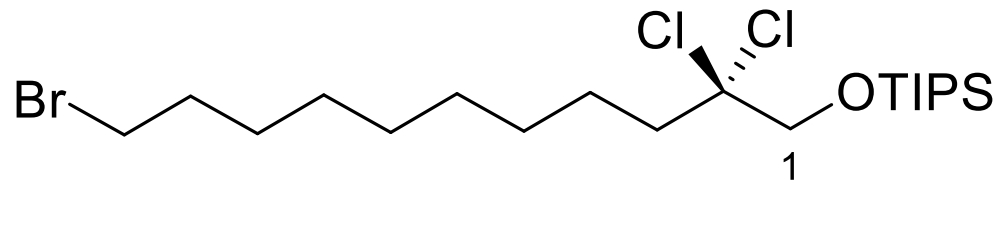

((11-Bromo-2,2-dichloroundecyl)oxy)triisopropylsilane (25)

To a solution of 11-bromo-2,2-dichloroundecan-1-ol (**24**) (0.81 g, 2.53 mmol) in CH_2Cl_2 (7.50 mL) at 0 °C were added TIPSOTf (0.68 mL, 2.53 mmol) and 2,6-lutidine (0.44 mL, 3.80 mmol). The reaction mixture was stirred at r. t. for 1 h, then more TIPSOTf (0.68 mL,

2.53 mmol) and 2,6-lutidine (0.44 mL, 3.80 mmol) were added. The reaction was quenched with sat. aq. $NaHCO_3$ (10.0 mL) and extracted with CH_2Cl_2 (3x10.0 mL). The combined organics were dried ($MgSO_4$) and concentrated *in vacuo*. Flash column chromatography (9:1, petroleum ether/CH_2Cl_2) gave **25** (1.18 g, 97%) as a colourless oil.

^{1}H NMR (400 MHz, $CDCl_3$) δ 4.04 (s, 2 H, H-1), 3.42 (t, *J* = 6.9, 2 H, H-11), 2.25–2.20 (m, 2 H), 1.91–1.82 (m, 2 H), 1.65–1.52 (m, 3 H, ROSiC<u>H</u>(CH_3)$_2$), 1.48–1.37 (m, 3 H), 1.37–1.29 (m, 8 H), 1.09 (d, *J* = 5.4, 18 H, ROSiCH(C<u>H</u>$_3$)$_2$); ^{13}C NMR (101 MHz, $CDCl_3$) δ 93.5 (C), 72.4 (CH_2), 43.5 (CH_2), 34.0 (CH_2), 32.8 (CH_2), 30.9 (CH_2), 29.2 (CH_2), 29.0 (CH_2), 28.7 (CH_2), 28.1 (CH_2), 24.8 (CH_2), 17.9 (6xCH_3), 12.0 (3xCH); IR ($CHCl_3$) ν_{max} 3042, 2941, 2867, 1463, 1125, 909 cm^{-1}; HRMS (ESI$^+$) $C_{20}H_{41}{}^{79}Br^{35}Cl_2OSiNa^+$ $[M^+Na]^+$ *m/z* calcd. 497.1379, found 497.1379.

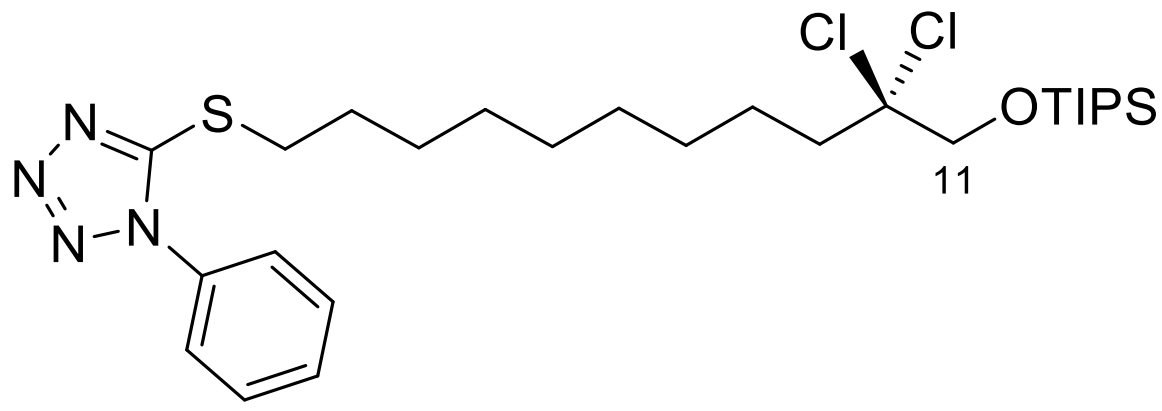

5-((10,10-Dichloro-11-((triisopropylsilyl)oxy)undecyl)thio)-1-phenyl-1H-tetrazole

To a solution of alkyl bromide **25** (1.18 g, 2.48 mmol) in DMF (12.0 mL) was added sodium 1-phenyl-1*H*-tetrazole-5-thiolate (645 mg, 3.22 mmol) and stirred at r. t. for 4 h. The reaction was quenched with H_2O (20.0 mL) and extracted with EtOAc (3x30.0 mL). The combined organics were washed with H_2O (4x100 mL), dried ($MgSO_4$) and concentrated *in vacuo* to give the title compound (1.42 g, quantitative) as a colourless oil.

^{1}H NMR (400 MHz, $CDCl_3$) δ 7.62–7.52 (m, 5 H, Ar<u>H</u>), 4.04 (s, 2 H, H-11), 3.41 (t, *J* = 7.4, 2 H, H-1), 2.25–2.19 (m, 2 H), 1.88–1.79 (m, 2 H), 1.64–1.55 (m, 3 H, ROSiC<u>H</u>(CH_3)$_2$), 1.50–1.41 (m, 2 H), 1.38–1.29 (m, 8 H), 1.16–1.12 (m, 2 H), 1.09 (d, *J* = 5.4, 18 H, ROSiCH(C<u>H</u>$_3$)$_2$); ^{13}C NMR (101 MHz, $CDCl_3$) δ 154.5 (C), 133.8 (C), 130.0 (CH), 129.8 (2xCH), 123.9 (2xCH), 93.5 (C), 72.4 (CH_2), 43.5 (CH_2), 33.4 (CH_2), 29.3 (CH_2), 29.2 (CH_2), 29.1 (CH_2), 29.0 (CH_2), 29.0 (CH_2), 28.6 (CH_2), 24.8 (CH_2), 17.9 (6xCH_3), 12.0 (3xCH); IR ($CHCl_3$) ν_{max} 2929, 2866, 1500, 1463, 1388, 1265, 1124, 909 cm^{-1}; HRMS (ESI$^+$) $C_{27}H_{47}{}^{35}Cl_2N_4OSSi^+$ $[M+H]^+$ *m/z* calcd. 573.2611, found 573.2613.

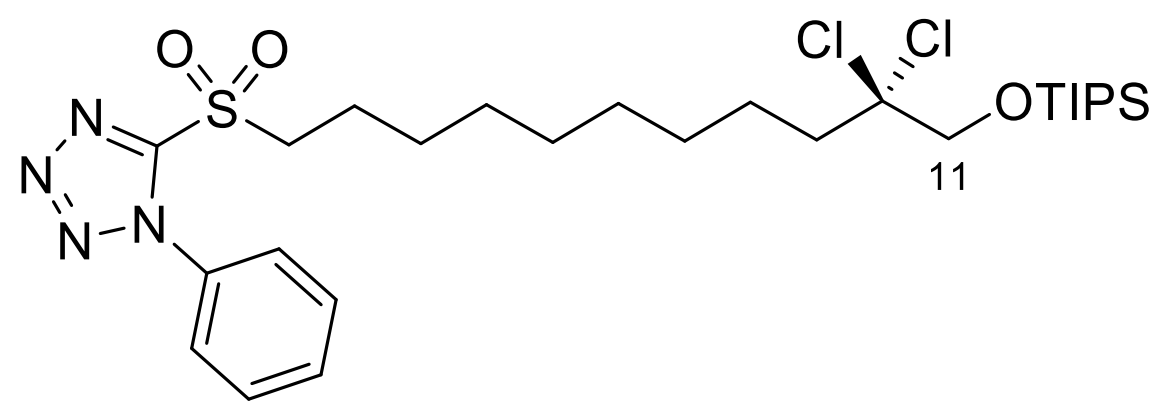

5-((10,10-Dichloro-11-((triisopropylsilyl)oxy)undecyl)sulfonyl)-1-phenyl-1H-tetrazole (8)

To a solution of the above sulfide (2.36 g, 4.11 mmol) in EtOH (40.0 mL) at 0 °C was slowly added a solution of ammonium molybdate (240 mg, 0.21 mmol) in H_2O_2 (2.94 mL, 28.8 mmol of 30% w/v aq. solution). The reaction mixture was stirred at r. t. for 24 h, then diluted with EtOAc (50.0 mL) and stirred for further 30 min. The organic layer was washed with H_2O (3x80.0 mL), dried ($MgSO_4$) and concentrated *in vacuo*. Flash column chromatography (4:1, petroleum ether/Et_2O) gave **8** (2.39 g, 96%) as a colourless oil.

^{1}H NMR (400 MHz, $CDCl_3$) δ 7.72–7.69 (m, 2 H, ArH), 7.65–7.58 (m, 3 H), 4.04 (s, 2 H, H-11), 3.77–3.71 (m, 2 H), 2.25–2.19 (m, 2 H), 2.01–1.92 (m, 2 H), 1.65–1.55 (m, 3 H,

ROSiC<u>H</u>(CH_3)$_2$), 1.54–1.47 (m, 2 H), 1.40–1.29 (m, 9 H), 1.17–1.12 (m, 2 H), 1.08 (d, *J* = 5.4, 18 H, ROSiCH(C<u>H</u>$_3$)$_2$); ^{13}C NMR (101 MHz, $CDCl_3$) δ 153.5 (C), 133.1 (C), 131.4 (CH), 129.7 (2xCH), 125.1 (2xCH), 93.5 (C), 72.4 (CH_2), 56.0 (CH_2), 43.5 (CH_2), 29.2 (CH_2), 29.0 (CH_2), 29.0 (CH_2), 28.8 (CH_2), 28.1 (CH_2), 24.8 (CH_2), 21.9 (CH_2), 17.9 (6x CH_3), 12.0 (3xCH); IR ($CHCl_3$) ν_{max} 3009, 2943, 2866, 2281, 1596, 1498, 1463, 1343, 1153, 1014 cm^{-1}; HRMS (ESI$^+$) $C_{27}H_{47}{}^{35}Cl_2N_4O_3SSi^+$ [M+H]$^+$ *m/z* calcd. 605.2510, found 605.2518.

II.D Julia-Kocienski olefination

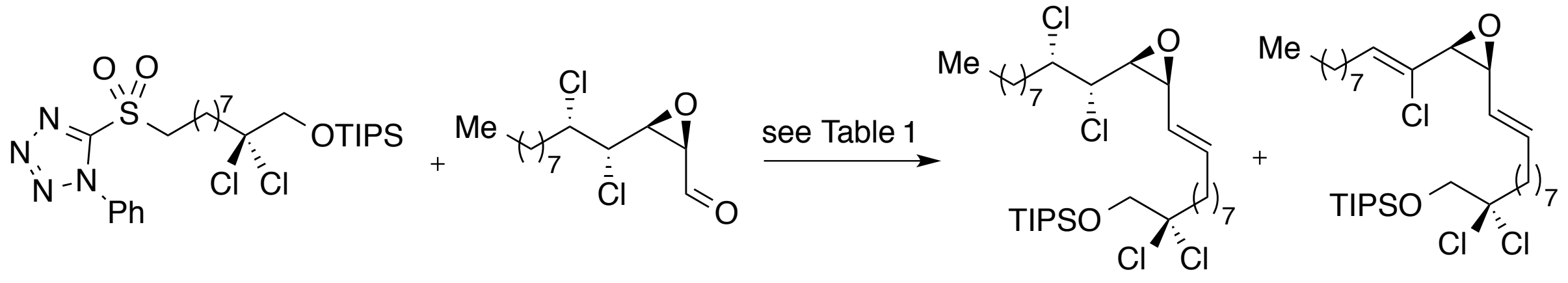

8 **9a** **7a**

Table S1. Reaction optimization

Entry	Base (Equiv.)	Equiv. **8**	Solvent	Additive (Equiv.)	Temp.	Deprotonation Time	Yield (%)[b]	*E*/*Z* ratio[b]
1	KHMDS (1.4)	1.2	THF	none	−78 °C	45 min.	31	1.5:1
2	NaHMDS (1.4)	1.2	THF	none	−78 °C	45 min.	48	1.5:1
3	KHMDS (1.4)	1.2	DME	none	−50 °C	45 min.	0	N/A
4	NaHMDS (1.4)	1.2	THF	none	−78 °C	90 min.	23	1.5:1
5	NaHMDS (1.4)	1.2	THF	none	−78 °C	20 min.	37	1.5:1
6	NaHMDS (1.4)	1.2	THF	none	−78 °C	5 min.	0	N/A
7	LiHMDS (1.3)	1.3	DMF/HMPA	none	−35 °C	5 min.	43	2.5:1
8	KHMDS (1.3)	1.1	THF	18-C-6 (2.2)	−78 °C	0 min.	64 (3.5:1 **8a**/**S4**)	4:1
9	KHMDS (1.3)	1.5	THF	18-C-6 (2.2)	−78 °C	0.5 min.	75	4:1
10	KHMDS (1.3)	1.5	THF	18-C-6 (2.6)	−78 °C	0.5 min.	85	4:1
11	KHMDS (1.3)	1.5	THF	18-C-6 (5.0)	−78 °C	0.5 min.	86	4:1

[a] *Reactions were typically conducted with 0.14 mmol with respect to* ***9a*** *in 4.20 mL of solvent.*
[b] *Isolated yield following chromatography (short silica pad).*

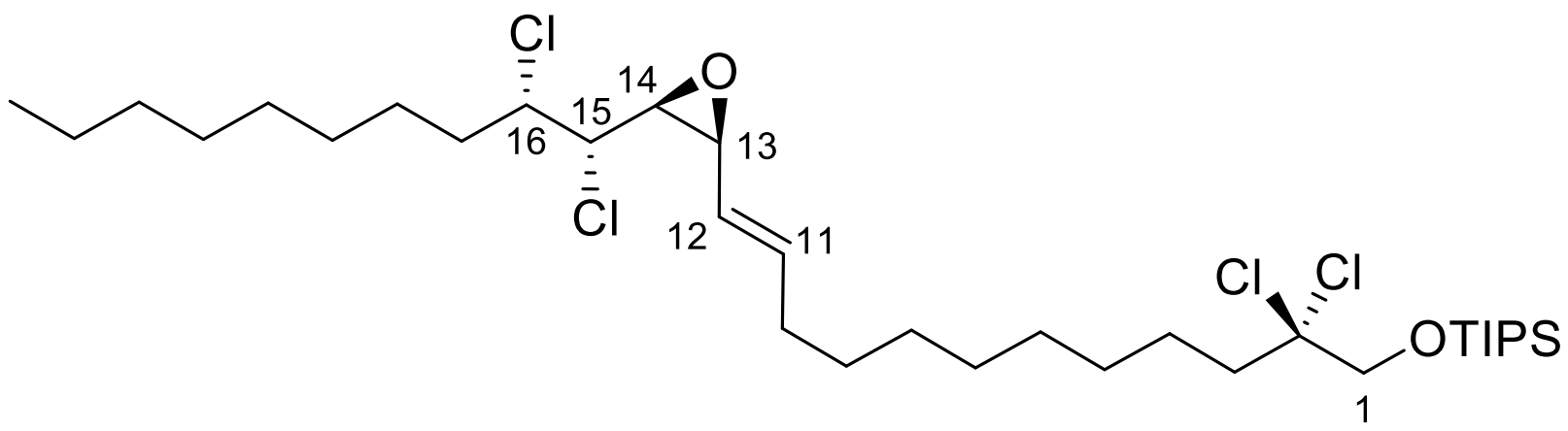

(13*S*,14*S*,15*S*,16*S*,*E*)-15,16-Dichloro-13,14-epoxy-1-((triisopropylsilyl)oxy)tetracos-11-ene (7a)

To a solution of sulfone **8** (323 mg, 0.53 mmol) and 18-crown-6 (230 mg, 0.87 mmol) in THF (1.80 mL) at −78 °C was added KHMDS (0.92 mL, 0.46 mmol of 0.5 M solution in toluene) and the reaction mixture was stirred for 0.5 min. A solution of aldehyde **9a** (100 mg, 0.36 mmol) in THF (3.60 mL) was slowly added to the reaction mixture at −78 °C and stirred at r. t. for 16 h. The reaction mixture concentrated *in vacuo*. Flash column chromatography (short plug of silica; 3:1, petroleum ether/CH_2Cl_2) gave **7a** (201 mg, 86% of 4:1 *E*/*Z* stereoisomers) as a colourless oil.

^{1}H NMR (400 MHz, C_6D_6) δ 5.78 (dt, *J* = 15.5, 6.7, 1 H, H-11), 5.32 (dd, *J* = 15.5, 6.1, 1 H, H-12), 4.25 (ddd, *J* = 8.7, 5.2, 2.9, 1 H, H-16), 4.09 (s, 2 H, H-1), 3.78 (dd, *J* = 8.9, 2.9, 1 H, H-15), 3.52 (dd, *J* = 8.9, 4.1, 1 H, H-14), 3.28 (dd, *J* = 6.1, 4.1, 1 H, H-13), 2.34–2.27 (m, 2

H), 1.95–1.85 (m, 2 H), 1.75 - 1.67 (m, 3 H, ROSiCH(CH_3)$_2$), 1.31–1.13 (m, 25 H), 1.06 (d, *J* = 5.4, 18 H, ROSiCH(CH_3)$_2$), 0.92 (t, *J* = 7.1, 3 H, H-24); ^{13}C NMR (101 MHz, C_6D_6) δ 138.8 (CH), 123.2 (CH), 94.3 (C), 73.3 (CH_2), 64.4 (CH), 61.7 (CH), 59.1 (CH), 57.5 (CH), 44.4 (CH_2), 36.6 (CH_2), 32.6 (CH_2), 30.1 (CH_2), 30.1 (CH_2), 30.0 (CH_2), 30.0 (CH_2), 29.9 (CH_2), 29.8 (CH_2), 29.7 (CH_2), 29.7 (CH_2), 29.6 (CH_2), 27.0 (CH_2), 25.7 (CH_2), 23.4 (CH_2), 18.4 (6xCH_3), 14.7 (CH_3), 12.6 (3xCH); IR ($CHCl_3$) ν_{max} 2930, 2865, 2281, 1665, 1463, 1382, 1330, 1250, 1124, 1070, 996, 963, 920 cm^{-1}; HRMS (ESI$^+$) $C_{33}H_{63}{}^{35}Cl_4O_2Si^+$ [M+H]$^+$ *m/z* calcd. 659.3346, found 659.3330.

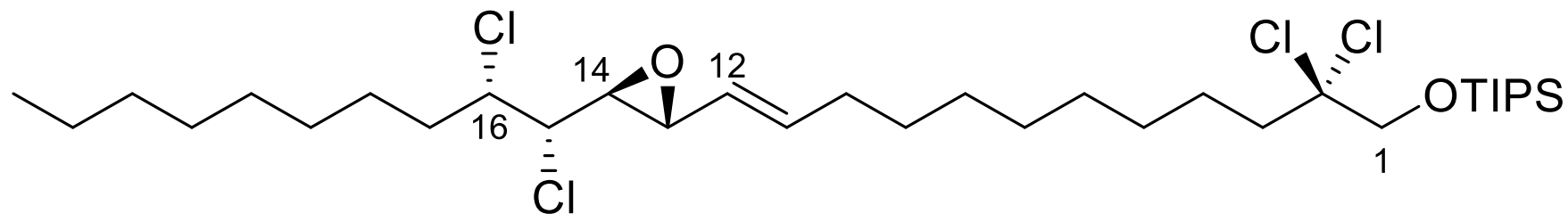

(13*R*,14*S*,15*S*,16*S*,*E*)-15,16-dichloro-13,14-epoxy-1-((triisopropylsilyl)oxy)tetracos-11-ene (8b)

To a solution of alcohol **18** (72.0 mg, 0.25 mmol) in CH_2Cl_2 (2.50 mL) at 0 °C was added DMP (162 mg, 0.38 mmol) and the reaction mixture was stirred at r. t. for 3 h. A saturated aqueous solution of $Na_2S_2O_4$ (2.00 mL) and a saturated aqueous solution of $NaHCO_3$ (2.00 mL) were added and the reaction mixture was stirred at r. t. for 30 min and extracted with CH_2Cl_2 (3x 5.00 mL). The combined organics were dried ($MgSO_4$) and concentrated *in vacuo* to give crude aldehyde **9b**, which was used without purification.

To a solution of sulfone **8** (204 mg, 0.34 mmol) and 18-crown-6 (154 mg, 0.58 mmol) in THF (1.80 mL) at −78 °C was added KHMDS (0.58 mL, 0.29 mmol of 0.5 M solution in toluene) and the reaction mixture was stirred for 0.5 min. A solution of crude aldehyde **9b** (70.0 mg, 0.25 mmol) in THF (3.60 mL) was slowly added to the reaction mixture at −78 °C and stirred at r. t. for 16 h. The reaction mixture concentrated *in vacuo*. Flash column chromatography (short plug of silica; 3:1, petroleum ether/CH_2Cl_2) gave **7b** (70 mg, 48% of 7:1 *E*/*Z* stereoisomers) as a colourless oil.

$[\alpha]_D$ = 16.6 (*c* = 0.5, $CHCl_3$); ^{1}H NMR (400 MHz, C_6D_6) δ 5.80 (dt, *J* = 15.5, 6.8, 1 H, H-11), 5.12 (ddt, *J* = 15.5, 7.9, 1.4, 1 H, H-12), 4.14 (ddd, *J* = 9.2, 4.7, 2.3, 1 H, H-16), 4.10 (s, 2 H, H-1), 3.44–3.37 (m, 2 H, H-14 + H-15), 3.18 (dd, *J* = 7.9, 1.3, 1 H, H-13), 2.36–2.29 (m, 2 H), 1.93–1.86 (m, 3 H), 1.77–1.68 (m, 3 H), 1.64–1.54 (m, 1 H), 1.41–1.24 (m, 9 H), 1.24–0.97 (m, 33 H), 0.93 (t, *J*=7.1 Hz, 3 H); ^{13}C NMR (101 MHz, C_6D_6) δ 137.4 (CH), 126.2 (CH), 93.7 (C), 72.7 (CH_2), 64.7 (CH), 63.1 (CH), 59.9 (CH), 59.0 (CH), 43.8 (CH_2), 35.7 (CH_2), 32.4 (CH_2), 32.0 (CH_2), 29.5 (CH_2), 29.4 (CH_2), 29.3 (CH_2), 29.2 (CH_2), 29.1 (CH_2), 28.8 (CH_2), 26.4 (CH_2), 25.1 (CH_2), 22.8 (CH_2), 17.8 (6xCH_3), 14.1 (CH_3), 12.0 (3xCH); IR ($CHCl_3$) ν_{max} 2929, 2866, 1464, 1382, 1124, 1070, 883 cm^{-1}; HRMS (ESI$^+$) $C_{33}H_{66}{}^{35}Cl_4NO_2Si^+$ [M+NH_4]$^+$ *m/z* calcd. 676.3611, found 676.3580.

II.E Opening of the C13-C14 epoxide

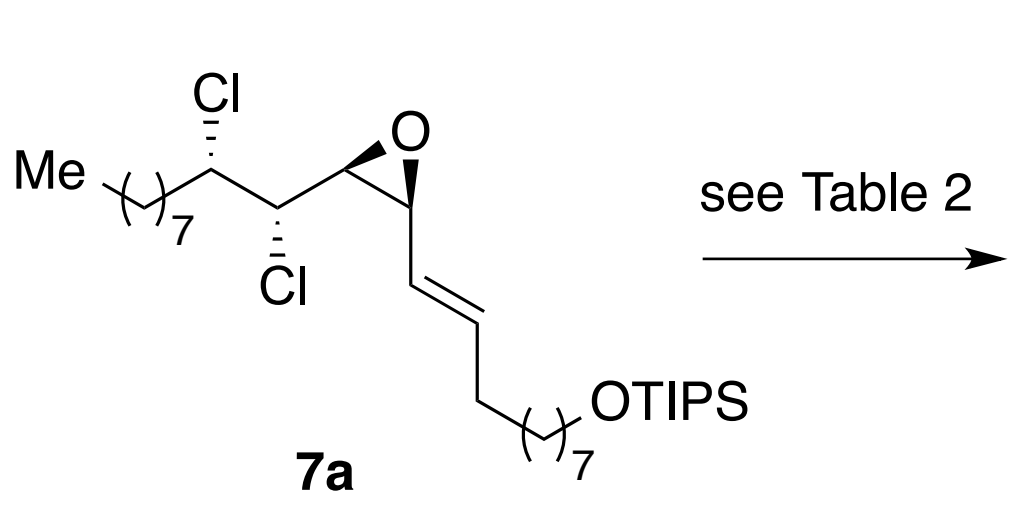

Table S2. Reaction optimization

Entry	Reagent	Solvent	Temp. (°C)	Time (min)	Yield (%)	dr
1	HCl	Et_2O	0	30	50	1:1
2	$SOCl_2$	Et_2O	0	30	48	1:1
3	TiCl(O*i*-Pr)$_3$	C_6H_6	r. t.	30	71	3:1
4	TiCl(O*i*-Pr)$_3$	C_6H_6	10	20	62	7:1
5	TiCl(O*i*-Pr)$_3$	C_6H_6	0	20	66	10:1
6	TiCl(O*i*-Pr)$_3$	C_6H_6	−20	120	66	25:1

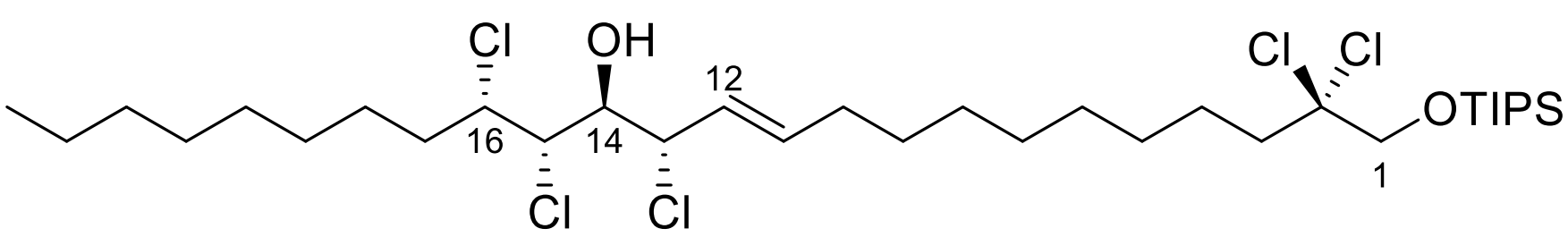

(13*S*,14*S*,15*S*,16*S*,*E*)-2,2,13,15,16-Pentachloro-1-((triisopropylsilyl)oxy)tetracos-11-en-14-ol (26)

To a solution of epoxide **7a** (130 mg, 0.20 mmol) in toluene (2.0 mL) at −20 °C was added TiCl(O*i*-Pr)$_3$ (0.26 mL, 0.26 mmol of 1.0 M solution in hexanes) and the reaction mixture was stirred for 2 h. The reaction was warmed to r. t. and saturated aqueous solution of potassium sodium tartrate (3.00 mL) and EtOAc (3.00 mL) were added. The reaction mixture was stirred at r. t. for 16 h and extracted with EtOAc (3x3.00 mL). The combined organics were dried (Na_2SO_4) and concentrated *in vacuo*. Flash column chromatography (2:1, petroleum ether/CH_2Cl_2) gave **26** (91.0 mg, 66%) as a colourless oil.

$[\alpha]_D$ = −15.6 (*c* = 0.2, $CHCl_3$); ^{1}H NMR (400 MHz, C_6D_6) δ 5.79–5.62 (m, 2 H, H-11 + H-12), 4.97 (dd, *J* = 9.3, 2.1, 1 H, H-13), 4.54 (ddd, *J* = 9.1, 5.1, 1.6, 1 H, H-16), 4.26 (ddd, *J* = 9.8, 3.9, 2.1, 1 H, C-14), 4.10 (s, 2 H, C-1), 3.75 (dd, *J* = 9.8, 1.6, 1 H, C-15), 2.35–2.28 (m, 2 H), 2.24 (d, *J* = 3.9, 1 H, RO<u>H</u>), 2.00–1.81 (m, 3 H), 1.76–1.67 (m, 2 H), 1.56–1.46 (m, 1 H), 1.32–1.23 (m, 4 H), 1.23–1.12 (m, 20 H), 1.09–1.05 (m, 21 H), 0.91 (t, *J*=7.0 Hz, 3 H); ^{13}C NMR (101 MHz, C_6D_6) δ 138.4 (CH), 124.8 (CH), 94.3 (C), 76.0 (CH), 73.4 (CH_2), 65.9 (CH), 65.1 (CH), 62.4 (CH), 44.4 (CH_2), 36.9 (CH_2), 32.8 (CH_2), 32.6 (CH_2), 30.1 (CH_2), 30.0 (CH_2), 29.9 (CH_2), 29.7 (CH_2), 29.7 (CH_2), 29.7 (CH_2), 29.4 (CH_2), 27.2 (CH_2), 25.7 (CH_2), 23.4 (CH_2), 18.4 (6xCH_3), 14.7 (CH_3), 12.6 (3xCH); IR ($CHCl_3$) ν_{max} 3692, 2929, 2865, 1601, 1464, 1261, 1121, 1070, 973 cm^{-1}; HRMS (ESI$^+$) $C_{33}H_{63}{}^{35}Cl_5O_2SiNa^+$ $[M+H]^+$ *m/z* calcd. 717.2932, found 717.3010.

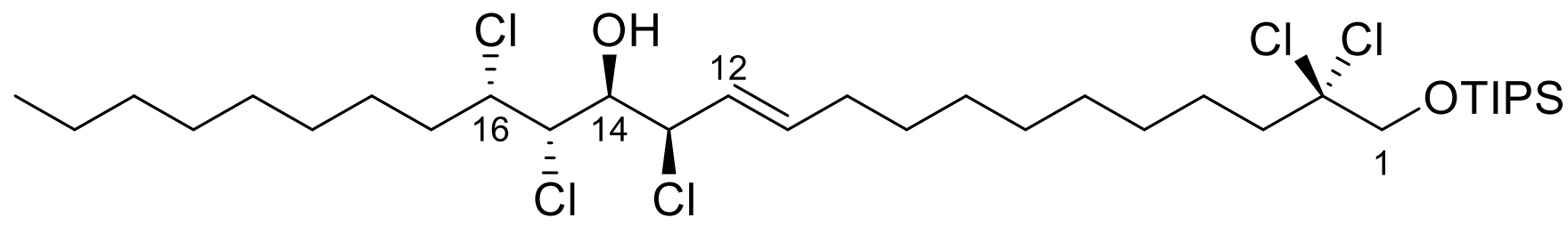

(13R,14S,15S,16S,E)-2,2,13,15,16-pentachloro-1-((triisopropylsilyl)oxy)tetracos-11-en-14-ol (32)

To a solution of epoxide **9b** (66.0 mg, 0.10 mmol) in toluene (1.00 mL) at −20 °C was added $TiCl(Oi\text{-}Pr)_3$ (0.13 mL, 0.13 mmol of 1.0 M solution in hexanes) and the reaction mixture was stirred for 2 h. The reaction mixture was warmed to r. t. and saturated aqueous solution of potassium sodium tartrate (2.00 mL) and EtOAc (1.50 mL) were added. The reaction mixture was stirred at r. t. for 16 h and extracted with EtOAc (3x3.00 mL). The combined organics were dried (Na_2SO_4) and concentrated *in vacuo*. Flash column chromatography (2:1, petroleum ether/CH_2Cl_2) gave **32** (50.0 mg, 71%) as a colourless oil.

$[\alpha]_D$ = 13.2 (*c* = 0.5, $CHCl_3$); ^{1}H NMR (400 MHz, CD_3OD) δ 5.91–5.76 (m, 2 H, H-11 + H-12), 4.96 (dd, *J* = 8.1, 1.5, 1 H, H-13), 4.60 (ddd, *J* = 8.8, 5.4, 1.3, 1 H, H-16), 4.17 (dd, *J* = 9.4, 1.5, 1 H, H-14), 4.09 (s, 2 H, H-1), 3.88 (dd, *J* = 9.4, 1.3, 1 H, H-15), 2.30–2.24 (m, 2 H), 2.14–2.09 (m, 2 H), 2.03–1.92 (m, 1 H), 1.90–1.79 (m, 1 H), 1.68–1.59 (m, 3 H), 1.50–1.40 (m, 6 H), 1.40–1.29 (m, 17 H), 1.25–1.11 (m, 24 H), 0.93 (t, *J* = 7.0, 3 H, H-24); ^{13}C NMR (101 MHz, CD_3OD) δ 136.2 (CH), 129.6 (CH), 94.7 (C), 76.0 (CH), 73.7 (CH_2), 66.4 (CH), 65.9 (CH), 62.8 (CH), 44.9 (CH_2), 37.7 (CH_2), 33.3 (CH_2), 33.1 (CH_2), 30.7 (CH_2), 30.5 (CH_2), 30.5 (CH_2), 30.3 (CH_2), 30.2 (CH_2), 30.1 (CH_2), 27.8 (CH_2), 26.1 (CH_2), 23.9 (CH_2), 18.6 (6xCH_3), 14.6 (CH_3), 13.4 (3xCH); IR ($CHCl_3$) ν_{max} 3695, 2929, 2858, 1601, 1464, 1261, 1099, 1014, 882 cm^{-1}; HRMS (ESI$^+$) $C_{33}H_{63}{}^{35}Cl_5NaO_2Si^+$ $[M+Na]^+$ *m/z* calcd. 717.2932, found 717.2935.

II.F Synthesis completion

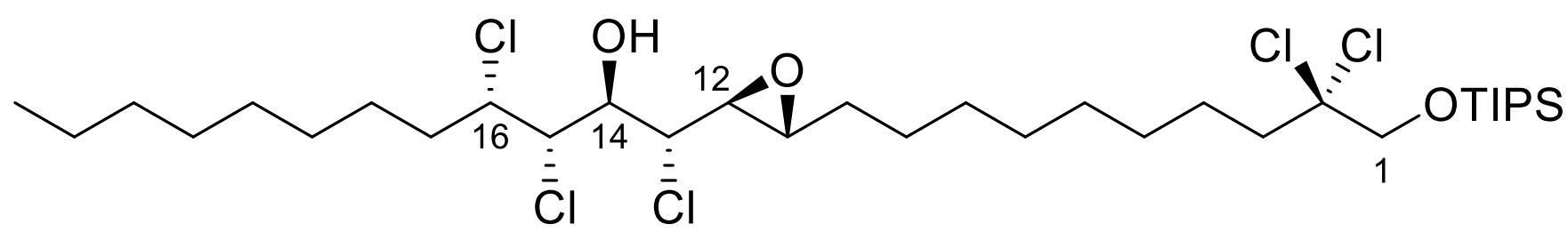

(11*R*,12*S*,13*R*,14*S*,15*S*,16*S*)-11,12-Epoxy-2,2,13,15,16-pentachloro-1-((triisopropylsilyl)oxy)tetracosan-14-ol (28)

To a solution of olefin **26** (35.0 mg, 0.05 mmol) in CH_2Cl_2 (0.10 mL) at 0 °C was added *m*CPBA (17.0 mg, 0.10 mmol) and the reaction mixture was stirred at r. t. for 24 h. A saturated aqueous solution of $Na_2S_2O_4$ (0.30 mL) and saturated aqueous solution of $NaHCO_3$ (0.50 mL) were then added and the reaction mixture stirred at r. t. for 1 h. The reaction mixture was extracted wit CH_2Cl_2 (3x1.50 mL). The combined organics were dried ($MgSO_4$) and concentrated *in vacuo*. Flash column chromatography (1:1, petroleum ether/CH_2Cl_2) gave **28** (13 mg, 36%) and **29** (13 mg, 36%) as colourless oils.

$[\alpha]_D$ = 57.7 (*c* = 0.1, $CHCl_3$); ^{1}H NMR (400 MHz, C_6D_6) δ 4.51 (ddd, *J* = 8.7, 5.7, 1.6, 1 H, H-16), 4.21 (ddd, *J* = 9.7, 4.7, 2.3, 1 H, H-14), 4.15 (dd, *J* = 8.2, 2.3, 1 H, H-13), 4.10 (s, 2 H, H-1), 3.93 (dd, *J* = 9.7, 1.6, 1 H, H-15), 3.08 (dd, *J* = 8.2, 1.9, 1 H, H-12), 2.99 (td, *J* = 5.6, 1.9, 1 H, H-11), 2.35 (d, *J* = 4.7, 1 H, RO<u>H</u>), 2.34–2.28 (m, 2 H), 1.99–1.89 (m, 1 H), 1.76–1.68 (m, 2 H), 1.66–1.56 (m, 1 H), 1.37–1.35 (m, 2 H), 1.32–1.11 (m, 22 H), 1.09–1.01 (m, 21 H), 0.93 (t, *J*=6.9 Hz, 3 H, H-24); ^{13}C NMR (101 MHz, C_6D_6) δ 94.3 (C), 75.7 (CH), 73.4 (CH_2), 65.7 (CH), 64.3 (CH), 62.1 (CH), 59.8 (CH), 57.4 (CH), 44.4 (CH_2), 36.9 (CH_2), 32.6

(CH_2), 32.4 (CH_2), 30.8 (CH_2), 30.6 (CH_2), 30.1 (CH_2), 30.0 (CH_2), 30.0 (CH_2), 29.9 (CH_2), 29.7 (CH_2), 27.2 (CH_2), 26.5 (CH_2), 25.7 (CH_2), 23.4 (CH_2), 18.4 (6xCH_3), 14.7 (CH_3), 12.6 (3xCH); IR ($CHCl_3$) ν_{max} 3692, 2961, 2929, 2859, 1601, 1464, 1261, 1097, 1014, 881 cm^{-1}; HRMS (ESI^+) $C_{33}H_{63}{}^{35}Cl_5O_3SiNa^+$ $[M+H]^+$ *m/z* calcd. 733.2881, found 733.2915.

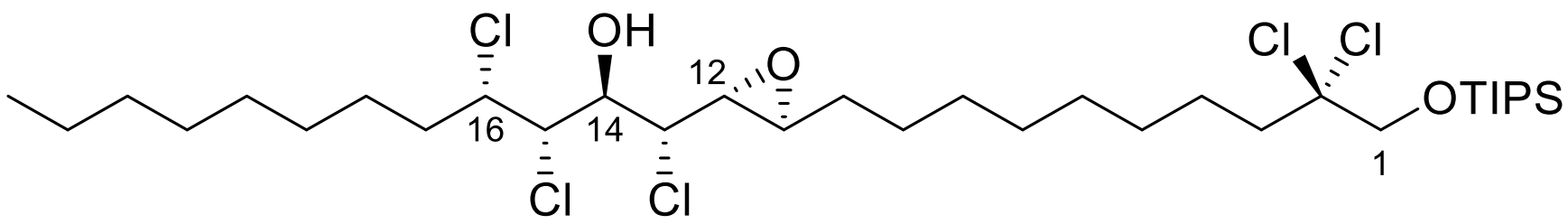

(11*S*,12*R*,13*R*,14*S*,15*S*,16*S*)-11,12-Epoxy-2,2,13,15,16-pentachloro-1-((triisopropylsilyl)oxy)tetracosan-14-ol (29)

$[\alpha]_D$ = 25.2 (*c* = 0.2, $CHCl_3$); ^1H NMR (400 MHz, C_6D_6) δ 4.55–4.49 (m, 2 H, H-16 + H-14), 4.28 (dd, *J* = 9.8, 4.1, 1 H, H-15), 4.08 - 4.12 (m, 3 H, H-1 + H-13), 3.22 (dd, *J* = 8.8, 2.0, 1 H, H-12), 2.52 (td, *J* = 5.3, 2.0, 1 H, H-11), 2.34–2.27 (m, 2 H), 2.25 (d, *J*=5.1 Hz, 1 H, RO<u>H</u>), 1.99–1.88 (m, 1 H), 1.75–1.66 (m, 2 H), 1.58–1.48 (m, 1 H), 1.39–1.34 (m, 2 H), 1.31–1.01 (m, 43 H), 0.91 (t, *J*=7.1 Hz, 3 H); ^{13}C NMR (101 MHz, C_6D_6) δ 94.3 (C), 76.8 (CH), 73.4 (CH_2), 64.4 (CH), 63.3 (CH), 62.8 (CH), 58.6 (CH), 56.0 (CH), 44.4 (CH_2), 36.8 (CH_2), 32.5 (CH_2), 32.1 (CH_2), 30.0 (CH_2), 30.0 (CH_2), 30.0 (CH_2), 29.9 (CH_2), 29.8 (CH_2), 29.7 (CH_2), 29.7 (CH_2), 27.2 (CH_2), 26.5 (CH_2), 25.7 (CH_2), 23.4 (CH_2), 18.4 (6xCH_3), 14.7 (CH_3), 12.6 (3xCH); IR ($CHCl_3$) ν_{max} 3603, 2929, 2865, 1602, 1464, 1261, 1098, 1014, 882 cm^{-1}; HRMS (ESI^+) $C_{33}H_{63}{}^{35}Cl_5O_3SiNa^+$ $[M+H]^+$ *m/z* calcd. 733.2881, found 733.2911.

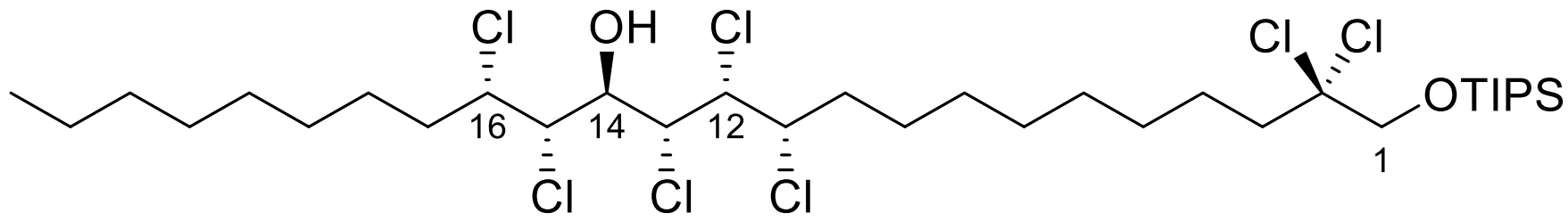

(11*S*,12*R*,13*R*,14*R*,15*S*,16*S*)-2,2,11,12,13,15,16-Heptachloro-1-((triisopropylsilyl)oxy)tetracosan-14-ol (30)

To a solution of triphenylphosphine oxide (70 mg, 0.25 mmol) in $CHCl_3$ (0.50 mL) was added oxalyl chloride (21.0 µL, 0.25 mmol) and the reaction mixture was stirred at r. t. for 5 min. The resulting solution of chlorotriphenylphosphonium chloride (0.10 mL, 0.05 mmol) was then added to a solution of epoxide **28** (24.0 mg, 0.03 mmol) in toluene (240 µL) at 90 °C and stirred for 3 h. The reaction mixture was concentrated *in vacuo*. Flash column chromatography (3:1, petroleum ether/CH_2Cl_2) gave **30** (7.00 mg, 27%) as a colourless oil.

$[\alpha]_D$ = −9.9 (c = 0.7, $CHCl_3$); ^{1}H NMR (400 MHz, C_6D_6) δ 4.71 (dd, J = 6.0, 4.1, 1 H, H-12), 4.64 (dd, J = 6.0, 3.6, 1 H, H-13), 4.48 (ddd, J = 8.4, 6.7, 3.6, 1 H, H-14), 4.38 (ddd, J = 9.0, 5.0, 1.8, 1 H, H-16), 4.35–4.31 (m, 1 H, H-11), 4.18 (dd, J = 8.4, 1.8, 1 H, H-15), 4.10 (s, 2 H, H-1), 2.35–2.28 (m, 2 H), 2.17 (d, J=6.7 Hz, 1 H), 1.91–1.76 (m, 3 H), 1.74–1.67 (m, 2 H), 1.58–1.47 (m, 2 H), 1.37–1.05 (m, 42 H), 0.93 (t, J = 6.9 Hz, 3 H, H-24); ^{13}C NMR (101 MHz, C_6D_6) δ 94.3 (C), 74.1 (CH), 73.3 (CH_2), 67.2 (CH), 66.9 (CH), 64.9 (CH), 64.3 (CH), 62.7 (CH), 44.4 (CH_2), 36.8 (CH_2), 36.0 (CH_2), 32.5 (CH_2), 30.8 (CH_2), 30.1 (CH_2), 29.9 (CH_2), 29.8 (CH_2), 29.7 (CH_2), 29.7 (CH_2), 29.4 (CH_2), 27.1 (CH_2), 26.7 (CH_2), 25.7 (CH_2), 23.4 (CH_2), 18.4 (6xCH_3), 14.7 (CH_3), 12.6 (3xCH); IR ($CHCl_3$) ν_{max} 3598, 3011, 2929, 2859, 1464, 1379, 1124, 882 cm^{-1}; HRMS (ESI$^-$) $C_{34}H_{64}{}^{35}Cl_7O_4Si^-$ [M+COOH]$^-$ m/z calcd. 809.2399, found 809.2424.

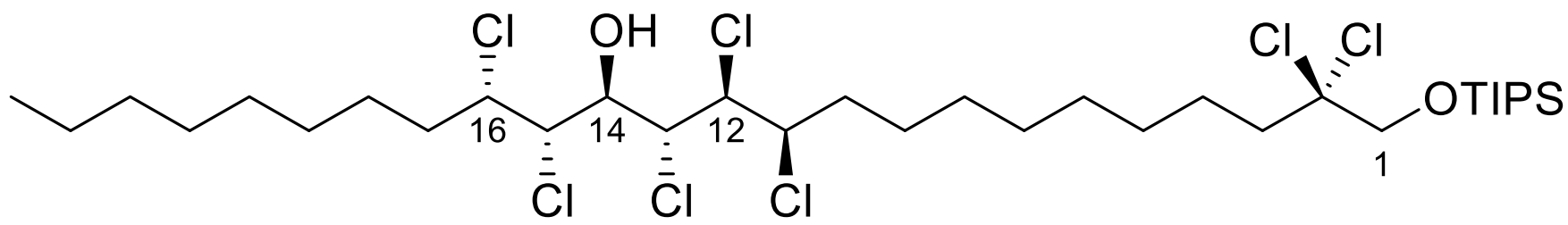

(11*R*,12*S*,13*R*,14*R*,15*S*,16*S*)-2,2,11,12,13,15,16-Heptachloro-1-((triisopropylsilyl)oxy)tetracosan-14-ol (31)

To a solution of triphenylphosphine oxide (70 mg, 0.25 mmol) in $CHCl_3$ (0.50 mL) was added oxalyl chloride (21.0 µL, 0.25 mmol) and the reaction mixture was stirred at r. t. for 5 min. The resulting solution of chlorotriphenylphosphonium chloride (0.10 mL, 0.05 mmol) was then added to a solution of epoxide **29** (25.0 mg, 0.04 mmol) in toluene (240 µL) at 90 °C and stirred for 3 h. The reaction mixture was concentrated *in vacuo*. Flash column chromatography (3:1, petroleum ether/CH_2Cl_2) gave **31** (9.00 mg, 35%) as a colourless oil.

$[\alpha]_D$ = 37.7 (c = 0.9, $CHCl_3$); ^{1}H NMR (400 MHz, C_6D_6) δ 4.93 (d, J = 10.5, 1 H, H-13), 4.69 (dd, J = 9.4, 6.9, 1 H, H-14), 4.65 (dd, J = 10.5, 1.4, 1 H, H-12), 4.62–4.57 (m, 2 H, H-15 + H-16), 4.42 (ddd, J = 8.9, 5.2, 1.4, 1 H, H-11), 4.10 (s, 2 H), 2.36–2.29 (m, 2 H), 1.98–1.90 (m, 2 H), 1.87 (d, J = 6.9, 1 H), 1.76–1.67 (m, 2 H), 1.60–1.47 (m, 3 H), 1.37–1.01 (m, 42 H), 0.92 (t, J = 7.1 Hz, 3 H, H-24); ^{13}C NMR (101 MHz, C_6D_6) δ 94.3 (C), 75.3 (CH), 73.4 (CH_2), 66.6 (CH), 65.1 (CH), 64.6 (CH), 64.0 (CH), 63.0 (CH), 44.4 (CH_2), 36.9 (CH_2), 36.7 (CH_2), 32.5 (CH_2), 30.8 (CH_2), 30.1 (CH_2), 29.9 (CH_2), 29.9 (CH_2), 29.7 (CH_2), 29.7 (CH_2), 29.6 (CH_2), 27.2 (CH_2), 27.1 (CH_2), 25.7 (CH_2), 23.4 (CH_2), 18.4 (6xCH_3), 14.7 (CH_3), 12.6 (3xCH); IR ($CHCl_3$) ν_{max} 3602, 2929, 2865, 1602, 1464, 1382, 1261, 1123, 1014, 882 cm^{-1}; HRMS (ESI$^-$) $C_{34}H_{64}{}^{35}Cl_7O_4Si^-$ [M+COOH]$^-$ m/z calcd. 809.2399, found 809.2428.

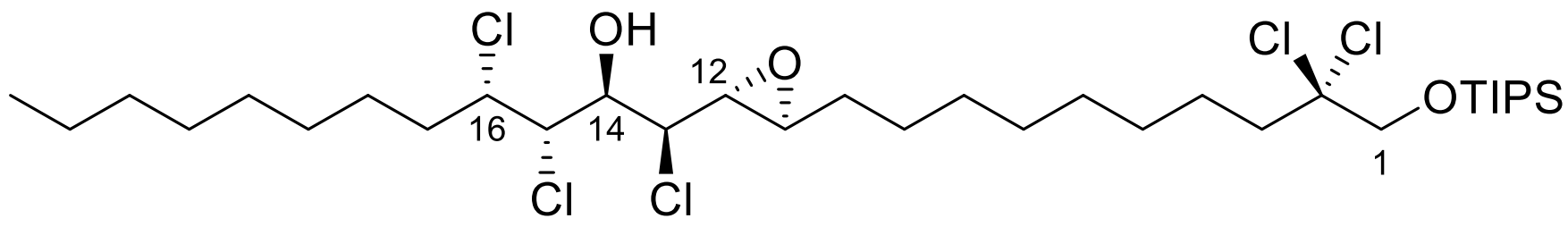

(11*S*,12*R*,13*S*,14*S*,15*S*,16*S*)-11,12-Epoxy-2,2,13,15,16-pentachloro-1-((triisopropylsilyl)oxy)tetracosan-14-ol (6a)

To a solution of olefin **32** (82.0 mg, 0.12 mmol) in CH_2Cl_2 (0.60 mL) at 0 °C was added *m*CPBA (40.0 mg, 0.24 mmol) and the reaction mixture was stirred at r. t. for 24 h. A saturated aqueous solution of $Na_2S_2O_4$ (0.50 mL) and a saturated aqueous solution of $NaHCO_3$ (0.50 mL) were then added and the reaction mixture was stirred at r. t. for 1 h. The reaction mixture was extracted with CH_2Cl_2 (3x2.00 mL) and the combined organics were dried ($MgSO_4$) and concentrated *in vacuo*. Flash column chromatography (17:3, petroleum ether/Et_2O) gave **6a** (37.0 mg, 45%) and **6b** (24.0 mg, 29%) as colourless oils.

$[\alpha]_D$ = 62.3 (*c* = 0.2, $CHCl_3$); ^{1}H NMR (400 MHz, C_6D_6) δ 4.45 (ddd, *J* = 9.2, 4.4, 1.6, 1 H, H-16), 4.43–4.38 (m, 1 H, H-14), 4.26 (dd, *J* = 7.0, 1.3, 1 H, H-13), 4.17 (dd, *J* = 9.4, 1.6, 1 H, H-15), 4.10 (s, 2 H, H-1), 3.05 (dd, *J* = 7.0, 2.0, 1 H, H-12), 2.61 (td, *J* = 5.0, 2.0, 1 H, H-11), 2.36–2.30 (m, 3 H), 2.01–1.91 (m, 1 H), 1.78–1.69 (m, 3 H), 1.52–1.46 (m, 1 H), 1.39–1.35 (m, 3 H), 1.33–1.12 (m, 20 H), 1.10–1.01 (m, 21 H), 0.93 (t, *J*=7.0 Hz, 3 H, H-24); ^{13}C NMR (101 MHz, C_6D_6) δ 94.3 (C), 73.4 (CH_2), 72.7 (CH), 65.1 (CH), 63.2 (CH), 62.2 (CH), 60.0 (CH), 58.8 (CH), 44.4 (CH_2), 37.0 (CH_2), 32.6 (CH_2), 32.1 (CH_2), 30.8 (CH_2), 30.1 (CH_2), 30.0 (CH_2), 30.0 (CH_2), 30.0 (CH_2), 29.9 (CH_2), 29.7 (CH_2), 27.3 (CH_2), 26.4 (CH_2), 25.7 (CH_2), 23.4 (CH_2), 18.4 (6xCH_3), 14.7 (CH_3), 12.6 (3xCH); IR ($CHCl_3$) ν_{max} 3689, 2929, 2860, 1602, 1464, 1261, 1239, 1099, 1013, 882 cm^{-1}; HRMS (ESI$^+$) $C_{33}H_{63}{}^{35}Cl_5NaO_3Si^+$ $[M+Na]^+$ *m/z* calcd. 733.2881, found 733.2885.

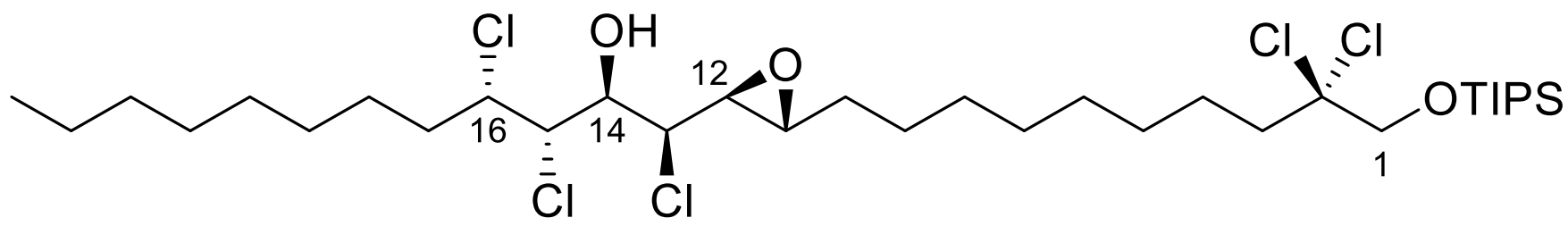

(11*R*,12*S*,13*S*,14*S*,15*S*,16*S*)-11,12-Epoxy-2,2,13,15,16-pentachloro-1-((triisopropylsilyl)oxy)tetracosan-14-ol (6b)

$[\alpha]_D$ = 101 (*c* = 0.1, $CHCl_3$); ^{1}H NMR (400 MHz, C_6D_6) δ 4.48 (ddd, *J* = 9.3, 3.9, 1.4, 1 H, H-16), 4.41 (dd, *J* = 6.2, 1.1, 1 H, H-13), 4.30 (dd, *J* = 9.5, 8.4, 1 H, H-14), 4.10 (s, 2 H), 3.99 (dd, *J* = 9.5, 1.4, 1 H, H-15), 2.84 (dd, *J* = 6.2, 1.9, 1 H, H-12), 2.73 (td, *J* = 4.9, 1.9 Hz, 1 H, H-11), 2.36–2.31 (m, 2 H), 2.27 (d, *J* = 8.4, 1 H, RO<u>H</u>) 2.02–1.91 (m, 1 H), 1.79–1.69 (m, 3 H), 1.55–1.42 (m, 4 H), 1.26–0.97 (m, 41 H), 0.93 (t, *J* = 7.0, 3 H, H-24); ^{13}C NMR (101 MHz, C_6D_6) δ 94.3 (C), 74.4 (CH), 73.3 (CH_2), 65.3 (CH), 64.4 (CH), 62.1 (CH), 60.0 (CH), 58.4 (CH), 44.4 (CH_2), 37.1 (CH_2), 32.6 (CH_2), 31.9 (CH_2), 30.8 (CH_2), 30.6 (CH_2), 30.1 (CH_2), 30.0 (CH_2), 30.0 (CH_2), 29.9 (CH_2), 29.7 (CH_2), 27.3 (CH_2), 26.4 (CH_2), 25.7 (CH_2), 23.4 (CH_2), 18.4 (6xCH_3), 14.7 (CH_3), 12.6 (3xCH); IR ($CHCl_3$) ν_{max} 3689, 2928, 2858, 1601, 1521, 1423, 1260, 1100, 1015, 820 cm^{-1}; HRMS (ESI$^+$) $C_{33}H_{63}{}^{35}Cl_5NaO_3Si^+$ $[M+Na]^+$ *m/z* calcd. 733.2881, found 733.2875.

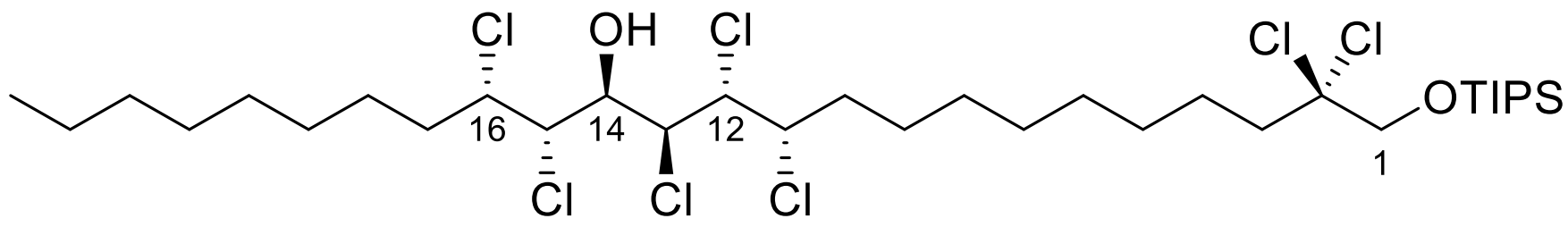

(11*S*,12*R*,13*S*,14*R*,15*S*,16*S*)-2,2,11,12,13,15,16-Heptachloro-1-((triisopropylsilyl)oxy)tetracosan-14-ol (33)

To a solution of triphenylphosphine oxide (70 mg, 0.25 mmol) in $CHCl_3$ (0.50 mL) was added oxalyl chloride (21.0 µL, 0.25 mmol) and the reaction mixture was stirred at r. t. for 5 min. The resulting solution of chlorotriphenylphosphonium chloride (0.04 mL, 0.02 mmol) was then added to a solution of epoxide **6b** (9.50 mg, 0.01 mmol) in toluene (90.0 µL) at 90 °C and stirred for 3 h. The reaction mixture was concentrated *in vacuo*. Flash column chromatography (3:1, petroleum ether/CH_2Cl_2) gave **33**(6.50 mg, 64%) as a colourless oil.

$[\alpha]_D$ = 89.0 (*c* = 0.1, $CHCl_3$); ^{1}H NMR (400 MHz, C_6D_6) δ 5.19 (dd, *J* = 10.3, 1.1, 1 H, H-13), 4.89 (ddd, *J* = 9.5, 8.9, 1.1, 1 H, H-14), 4.57 (ddd, *J* = 8.8, 4.6, 1.6, 1 H, H-16), 4.31 (ddd, *J* = 8.7, 4.9, 1.4, 1 H, H-11), 4.25 (dd, *J* = 10.3, 1.4 Hz, 1 H, H-12), 4.14 (dd, *J* = 9.5, 1.6 Hz, 1 H, H-15), 4.11 (s, 2 H, H-1), 2.37–2.31 (m, 2 H), 2.00–1.91 (m, 2 H), 1.79 (d, *J* = 8.9, 1 H, RO<u>H</u>), 1.77–1.70 (m, 3 H), 1.56–1.45 (m, 4 H), 1.27–1.057 (m, 40 H), 0.93 (t, *J* = 7.0, 3 H, H-24); ^{13}C NMR (101 MHz, C_6D_6) δ 94.3 (C), 73.4 (CH_2), 72.2 (CH), 65.4 (CH), 65.1 (CH), 64.9 (CH), 63.2 (CH), 62.5 (CH), 44.4 (CH_2), 37.2 (CH_2), 36.9 (CH_2), 32.6 (CH_2), 30.8 (CH_2), 30.6 (CH_2), 30.1 (CH_2), 30.0 (CH_2), 29.9 (CH_2), 29.7 (CH_2), 29.6 (CH_2), 27.3 (CH_2), 27.1 (CH_2), 25.7 (CH_2), 23.4 (CH_2), 18.4 (6xCH_3), 14.7 (CH_3), 12.6 (3xCH); IR ($CHCl_3$) ν_{max} 3691, 2928, 2858, 1602, 1464, 1377, 1264, 1126, 1014, 882 cm^{-1}; HRMS (ESI$^-$) $C_{34}H_{64}{}^{35}Cl_7O_4Si^-$ $[M+COOH]^-$ *m/z* calcd. 809.2399, found 809.2422.

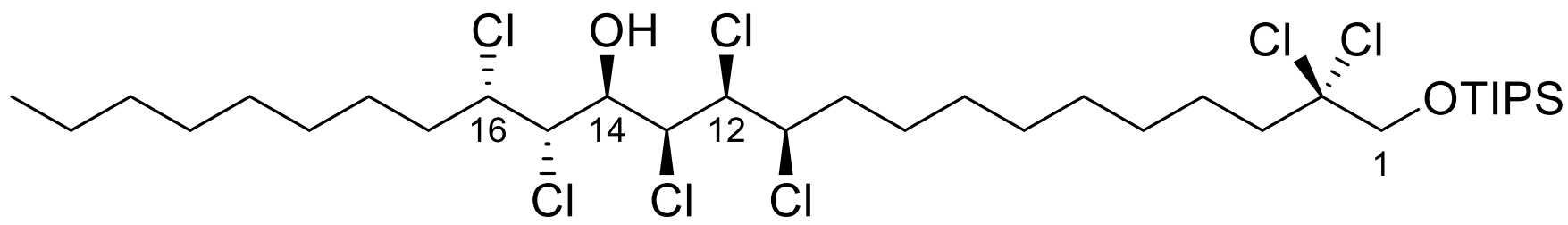

(11*R*,12*S*,13*S*,14*R*,15*S*,16*S*)-2,2,11,12,13,15,16-Heptachloro-1-((triisopropylsilyl)oxy)tetracosan-14-ol (34)

To a solution of triphenylphosphine oxide (70 mg, 0.25 mmol) in $CHCl_3$ (0.50 mL) was added oxalyl chloride (21.0 µL, 0.25 mmol) and the reaction mixture was stirred at r. t. for 5 min. The resulting solution of chlorotriphenylphosphonium chloride (0.12 mL, 0.06 mmol) was then added to a solution of epoxide **6a** (37.0 mg, 0.05 mmol) in toluene (0.40 mL) at 90 °C and stirred for 3 h. The reaction mixture was concentrated *in vacuo*. Flash column chromatography (3:1, petroleum ether/CH_2Cl_2) gave **34** (24.0 mg, 60%) as a colourless oil.

$[\alpha]_D$ = 14.8 (*c* = 0.1, $CHCl_3$); ^{1}H NMR (400 MHz, C_6D_6) δ 5.14 (dd, *J* = 8.6, 0.9, 1 H, H-13), 4.53 (ddd, *J* = 10.9, 9.3, 0.9, 1 H, H-14), 4.31 (ddd, *J* = 9.7, 4.0, 1.4, 1 H, H-16), 4.20 (dd, *J* = 8.6, 2.1, 1 H, H-12), 4.12–4.07 (m, 3 H, H-1 + H-11), 3.96 (dd, *J* = 9.3, 1.4 Hz, 1 H, H-15), 2.36–2.29 (m, 2 H), 2.00–1.90 (m, 1 H), 1.87–1.76 (m, 1 H), 1.76–1.62 (m, 4 H), 1.52–1.45 (m, 2 H), 1.32–1.30 (m, 1 H), 1.27–1.01 (m, 41 H), 0.93 (t, *J*=7.0 Hz, 3 H, H-24); ^{13}C NMR (101 MHz, C_6D_6) δ 94.3 (C), 73.4 (CH_2), 72.4 (CH), 68.5 (CH), 68.4 (CH), 65.4 (CH), 62.1 (CH), 62.0 (CH), 44.4 (CH_2), 37.2 (CH_2), 36.9 (CH_2), 32.6 (CH_2), 30.1 (CH_2), 30.0 (CH_2), 29.9 (CH_2), 29.8 (CH_2), 29.8 (CH_2), 29.7 (CH_2), 29.6 (CH_2), 27.3 (CH_2), 26.8 (CH_2), 25.7 (CH_2), 23.4 (CH_2), 18.4 (6xCH_3), 14.7 (CH_3), 12.6 (3xCH); IR ($CHCl_3$) ν_{max} 3691, 2929, 2858, 1602, 1464, 1261, 1094, 1014, 882 cm^{-1}; HRMS (ESI$^-$) $C_{34}H_{64}{}^{35}Cl_7O_4Si^-$ $[M+COOH]^-$ *m/z* calcd. 809.2399, found 809.2417.

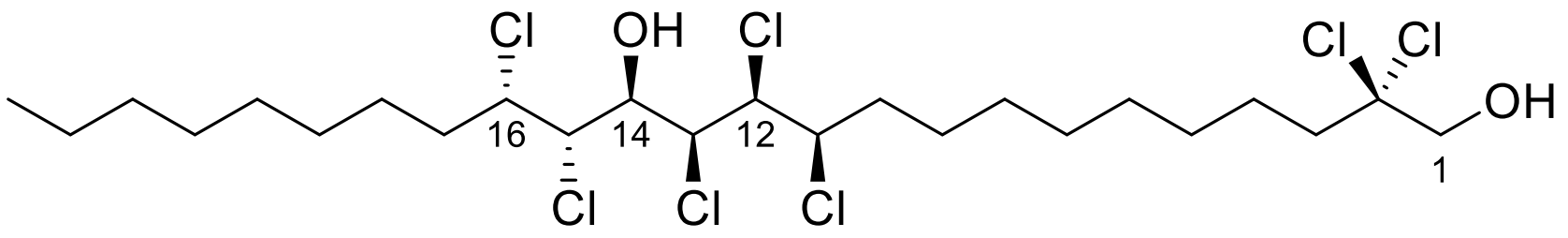

(11*R*,12*S*,13*S*,14*R*,15*S*,16*S*)-2,2,11,12,13,15,16-heptachlorotetracosane-1,14-diol (35)

To a solution of silyl ether **34** (24.0 mg, 0.03 mmol) in THF (0.15 mL) was added acetic acid (2 drops from a 1 mL syringe) and tetrabutylammonium fluoride trihydrate (39.0 mg, 0.12 mmol). The reaction was stirred at r. t. for 4 h after which time further tetrabutylammonium fluoride trihydrate (39.0 mg, 0.12 mmol) was added and the reaction mixture was stirred at r. t. for 20 h. The reaction was quenched with NH_4Cl (0.50 mL of 1.0 M aqueous solution) and the reaction mixture was extracted with EtOAc (3x1.00 mL). The combined organics were dried (Na_2SO_4) and concentrated in vacuo. Flash column chromatography (3:1, petroleum ether/EtOAc) gave **36** (19 mg, quantitative) as a yellow oil.

$[\alpha]_D$ = 53.2 (*c* = 0.3, $CHCl_3$); ^{1}H NMR (400 MHz, C_6D_6) δ 5.15 (dd, *J* = 8.6, 1.0, 1 H, H-13), 4.55 (dd, *J* = 9.3, 1.0, 1 H, H-14), 4.32 (ddd, *J* = 9.3, 4.1, 1.4, 1 H, H-16), 4.22 (dd, *J* = 8.6, 2.2, 1 H, H-12), 4.12 (ddd, *J* = 8.0, 5.6, 2.2, 1 H, H-11), 3.98 (dd, *J* = 9.3, 1.4, 1 H, H-15), 3.59 (s, 2 H, H-1), 2.11–2.03 (m, 2 H), 2.02–1.91 (m, 1 H), 1.88–1.64 (m, 4 H), 1.62–1.55 (m, 2 H), 1.51–1.44 (m, 2 H), 1.37–0.99 (m, 19 H), 0.93 (t, *J* = 7.0 Hz, 3 H, H-24); ^{13}C NMR (101 MHz, C_6D_6) δ 95.4 (C), 72.5 (CH_2), 72.4 (CH), 68.5 (CH), 68.4 (CH), 65.5 (CH), 62.1 (CH), 61.9 (CH), 44.1 (CH_2), 37.2 (CH_2), 36.9 (CH_2), 32.6 (CH_2), 30.6 (CH_2), 30.1 (CH_2), 30.0 (CH_2), 29.8 (CH_2), 29.7 (CH_2), 29.5 (CH_2), 29.5 (CH_2), 27.3 (CH_2), 26.7 (CH_2), 25.5 (CH_2), 23.4 (CH_2), 14.7 (CH_3); IR ($CHCl_3$) ν_{max} 3587, 3545, 2929, 2857, 1465, 1377, 1272, 1091, 851 cm^{-1}; HRMS (ESI$^+$) $C_{24}H_{43}{}^{35}Cl_7NaO_2^+$ $[M+Na]^+$ *m/z* calcd. 631.0975, found 631.0997.

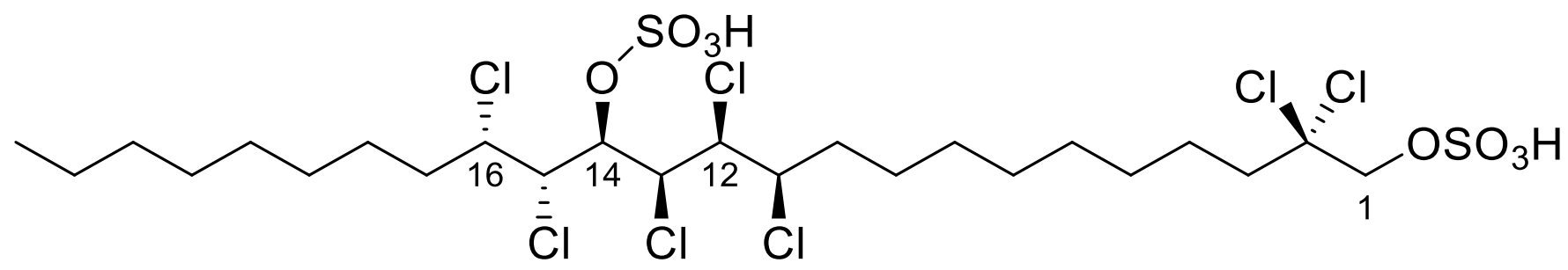

(11*R*,12*S*,13*S*,14*R*,15*S*,16*S*)-2,2,11,12,13,15,16-heptachlorotetracosane-1,14-diyl bis(hydrogen sulfate) (36)[4]

To a solution of diol **35** (10.0 mg, 0.02 mmol) in DMF (0.80 mL) was added SO_3-pyridine (13.0 mg, 0.08 mmol) and the reaction mixture was stirred at r. t. for 1 h and was immediately filtered through a short plug of silica (9:1, CH_2Cl_2/MeOH). The filtrate was concentrated in vacuo. Flash column chromatography (5:1 CH_2Cl_2/MeOH) gave **36** (8.40 mg, 68%) as an off-white solid.

$[\alpha]_D$ = 49.9 (*c* = 0.3, CH_3OH) [lit.[4,5] $[\alpha]_D$ = 6.4 (*c* = 0.5, CH_3OH)]; ^{1}H NMR (400 MHz, CD_3OD, 330 K) δ 5.04 (dd, *J* = 9.1, 0.9, 1 H, H-14), 4.96 (ddd, *J* = 8.6, 4.5, 1.8, 1 H, H-11), 4.84 (dd, *J* = 9.9, 0.9, 1 H, H-13), 4.79 (ddd, *J* = 8.7, 5.5, 1.1, 1 H, H-16), 4.66 (dd, *J* = 9.9, 1.8, 1 H, H-12), 4.31 (s, 2 H, H-1), 4.27 (dd, *J* = 9.1, 1.1, 1 H, H-15), 2.29–2.23 (m, 2 H), 2.00–1.76 (m, 5 H), 1.69–1.62 (m, 2 H), 1.60–1.50 (m, 2 H), 1.48–1.26 (m, 19 H), 0.89 (t, *J* = 7.0 Hz, 3 H, H-24); ^{13}C NMR (126 MHz, CD_3OD) δ 91.5 (C), 77.6 (CH_2), 75.7 (CH), 69.2 (CH), 67.7 (CH), 66.2 (CH), 63.3 (CH), 62.2 (CH), 45.3 (CH_2), 38.6 (CH_2), 37.9 (CH_2), 33.2 (CH_2), 30.7 (CH_2), 30.6 (CH_2), 30.6 (CH_2), 30.5 (CH_2), 30.3 (CH_2), 30.3 (CH_2), 30.2 (CH_2), 27.8 (CH_2), 27.4 (CH_2), 26.0 (CH_2), 23.9 (CH_2), 14.6 (CH_3); IR ($CHCl_3$) ν_{max} 3633, 3008, 2929, 2857, 1710, 1457, 1363, 1261, 1112, 1033, 862 cm^{-1}; HRMS (ESI$^-$) $C_{24}H_{41}{}^{35}Cl_7O_8S_2{}^{2-}$ $[M-2H]^{2-}$ *m/z* calcd. 383.0037, found 383.0042.

[5] The literature compound was isolated as the bis sodium salt.

III. NMR Spectra

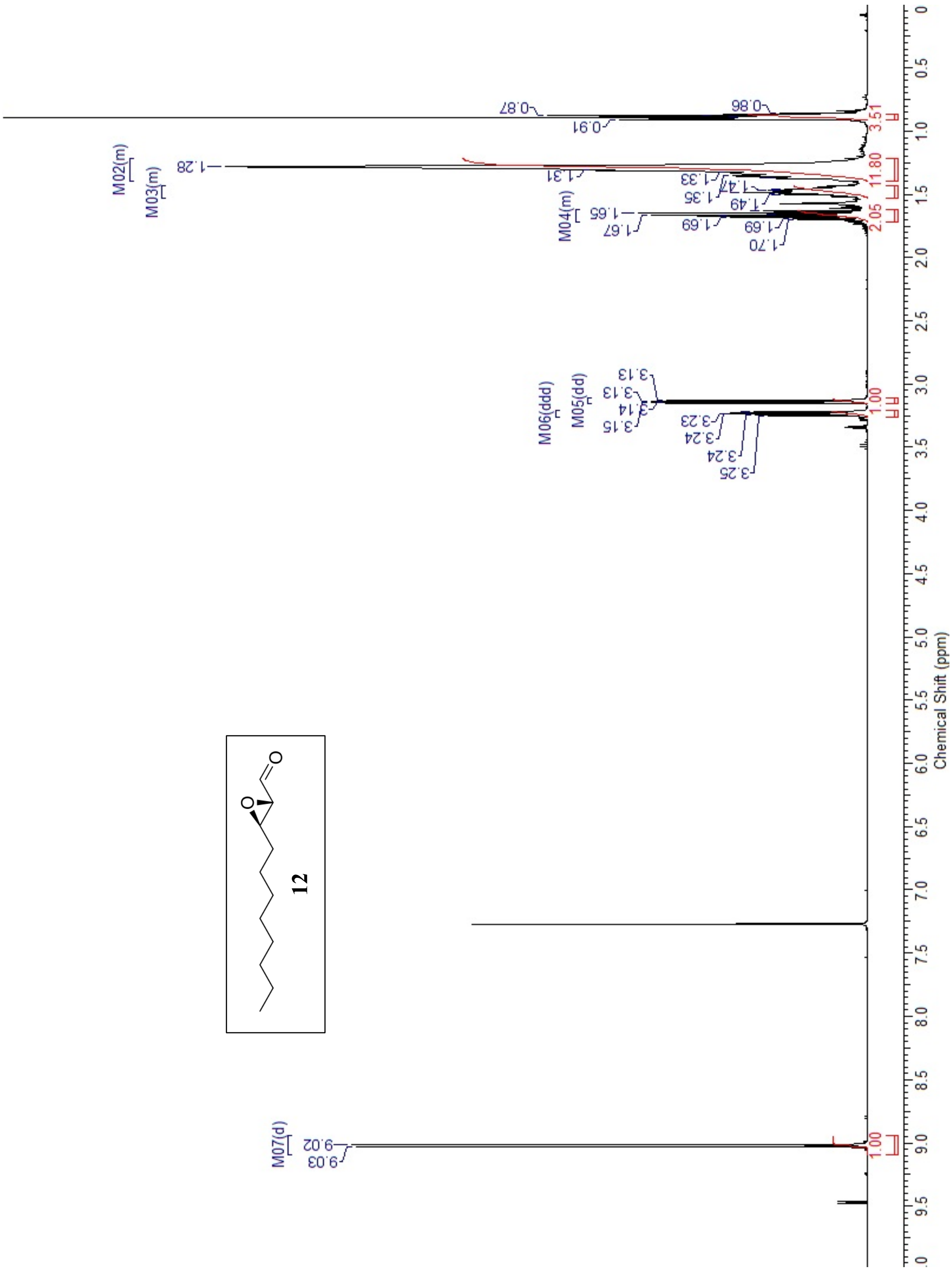

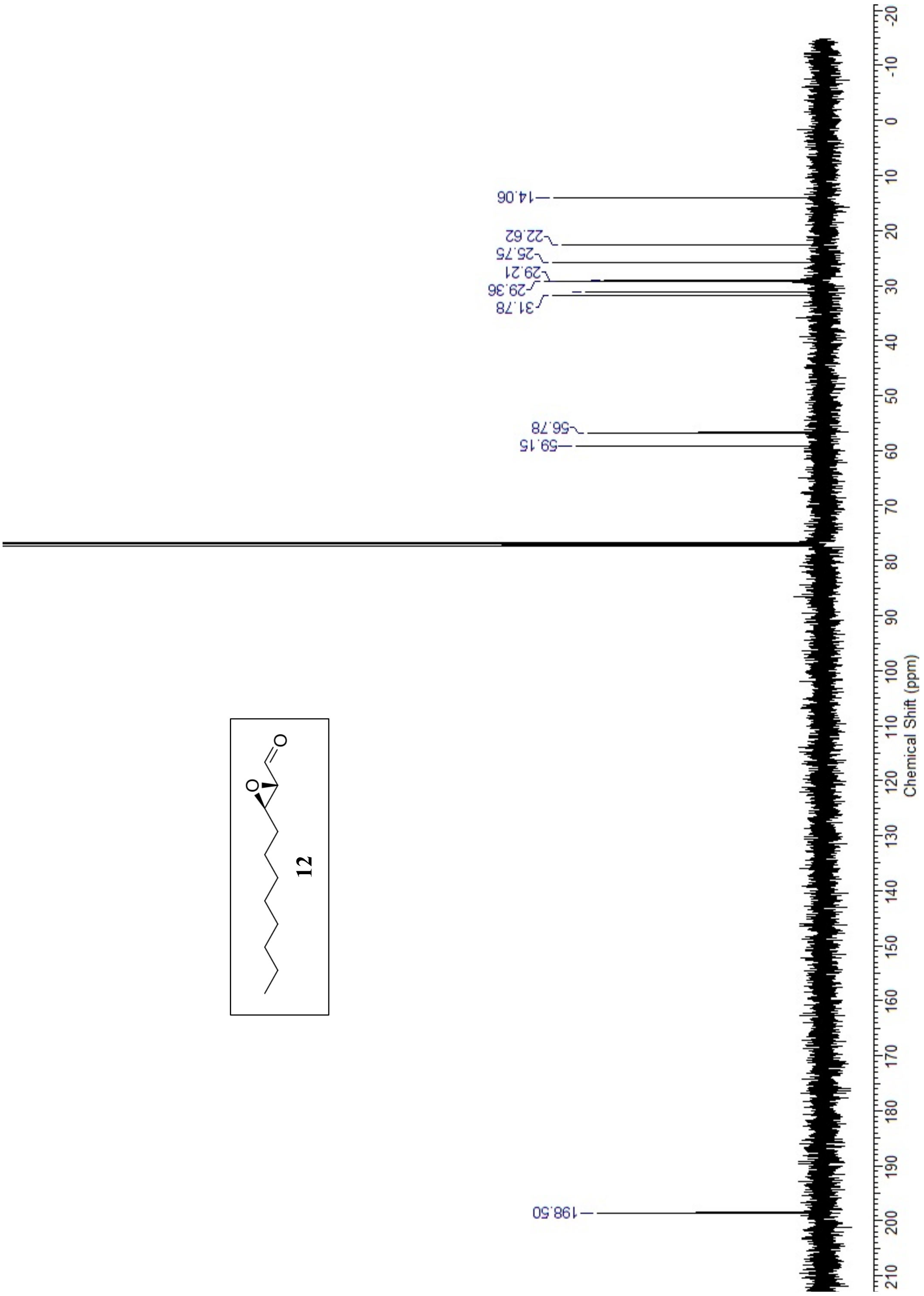
14.06
22.62
25.75
29.21
29.36
31.78
56.78
59.15
198.50
Chemical Shift (ppm)
12

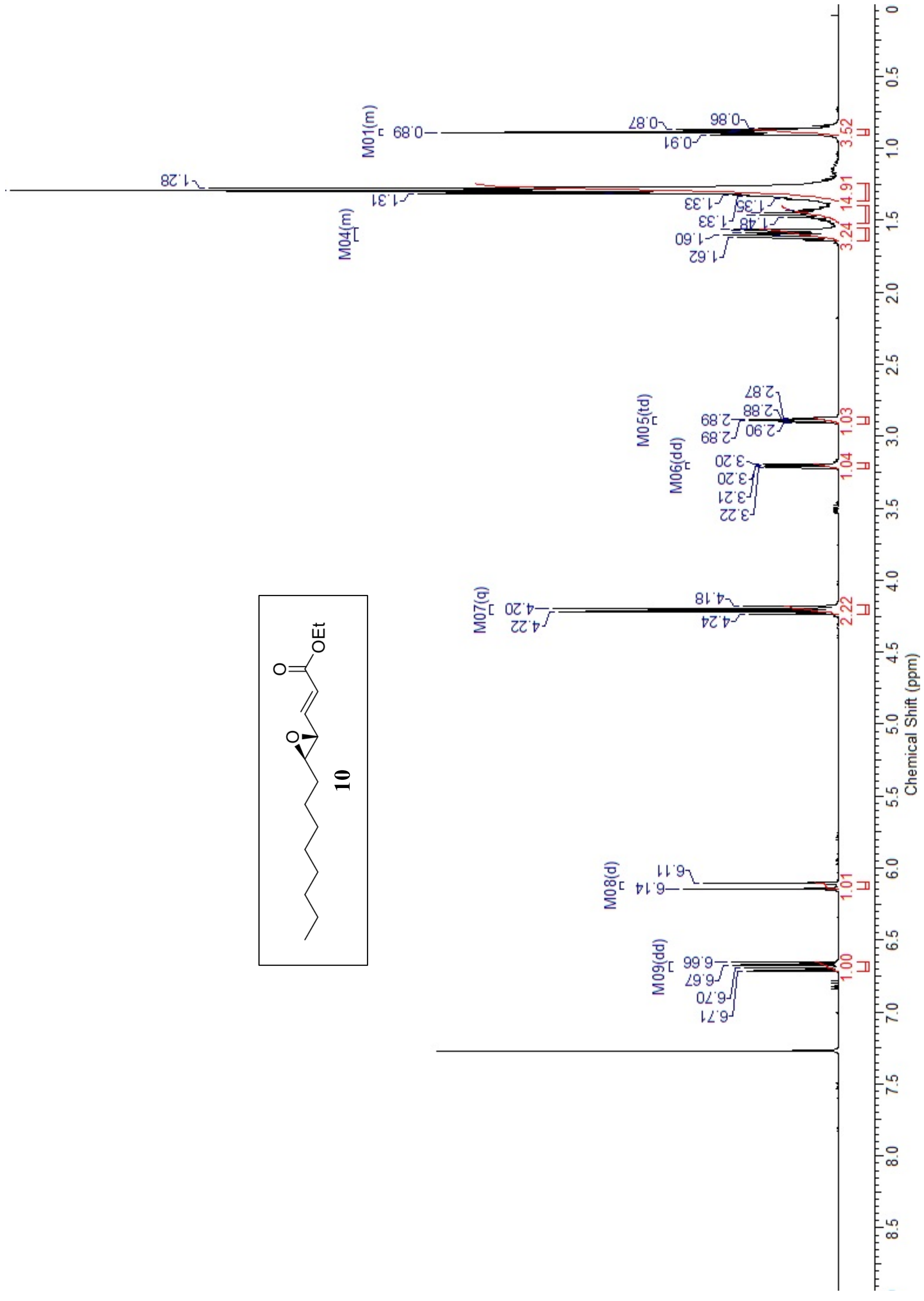
OEt
O
O
10
M01(m)
M04(m)
M05(td)
M06(dd)
M07(q)
M08(d)
M09(dd)
3.52
14.91
3.24
1.03
1.04
2.22
1.01
1.00
Chemical Shift (ppm)

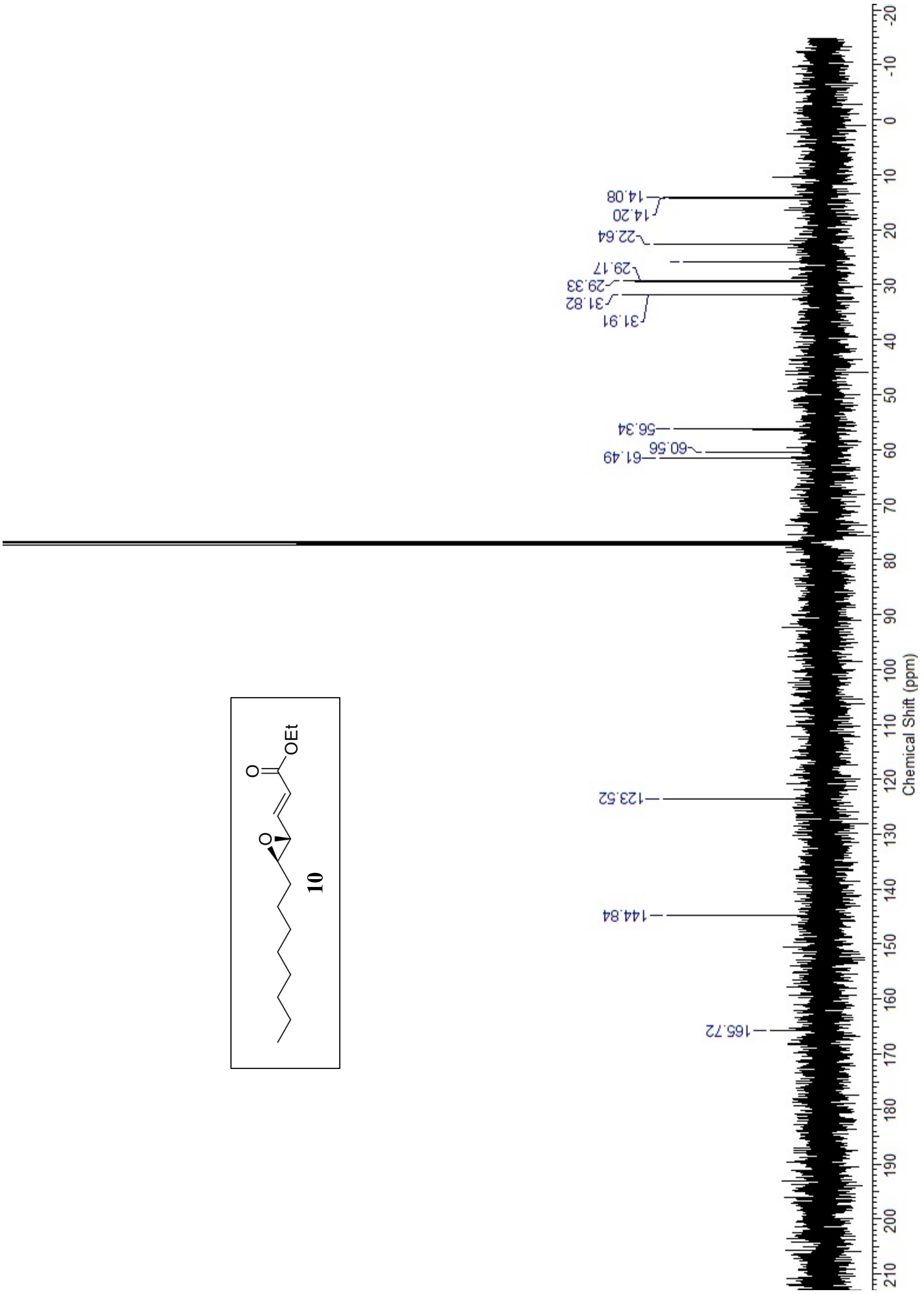
14.08
14.20
22.64
29.17
29.33
31.82
31.91
56.34
60.56
61.49
123.52
144.84
165.72
Chemical Shift (ppm)
O
OEt
10

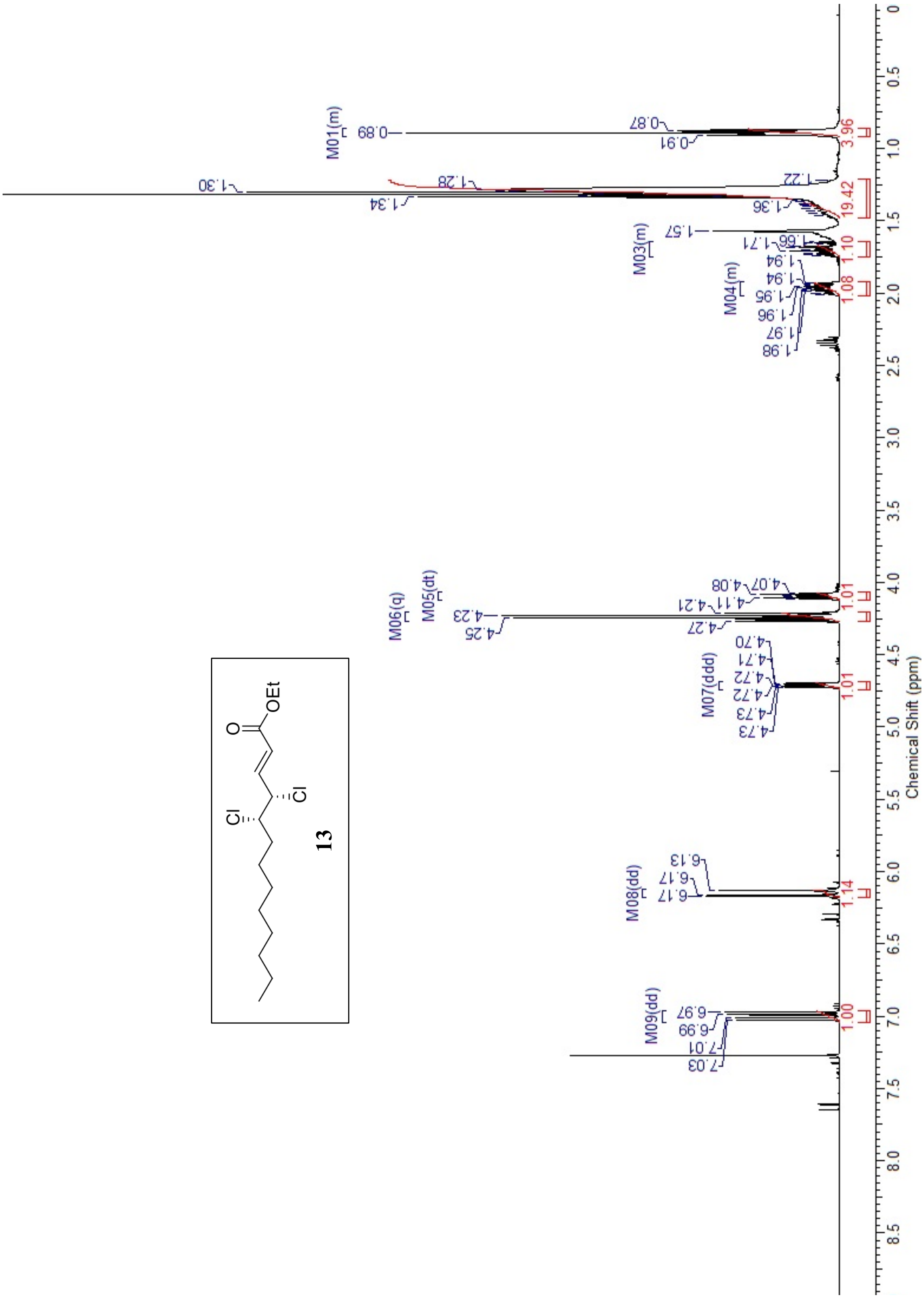

OEt
Cl
13
M01(m)
M03(m)
M04(m)
M05(dt)
M06(q)
M07(ddd)
M08(dd)
M09(dd)
Chemical Shift (ppm)

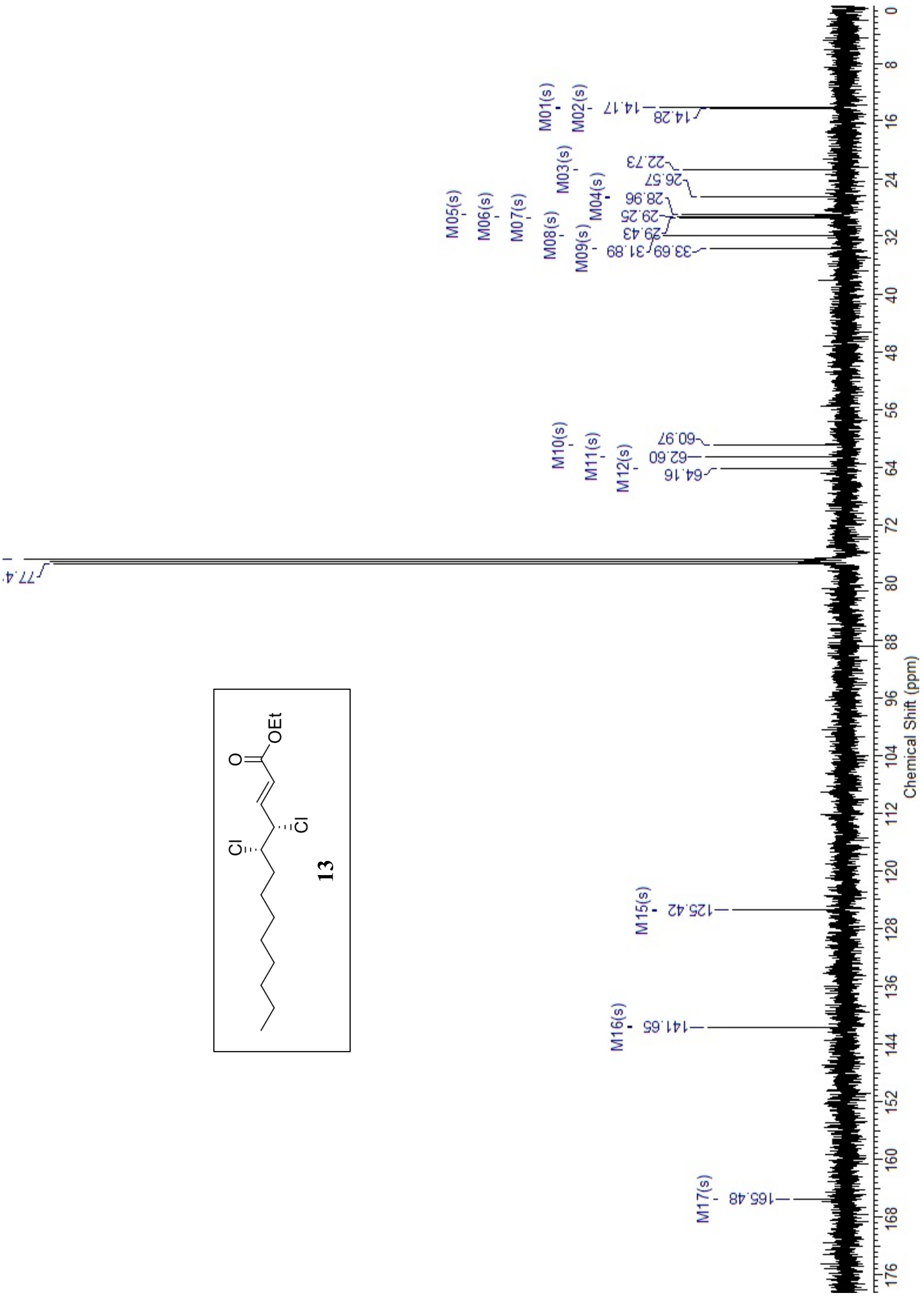
M01(s)
M02(s)
14.17
14.28
M03(s)
22.73
26.57
M04(s)
28.96
29.25
29.43
M05(s)
M06(s)
M07(s)
M08(s)
M09(s)
31.89
33.69
M10(s)
60.97
M11(s)
62.60
M12(s)
64.16
77.4
M15(s)
125.42
M16(s)
141.65
M17(s)
165.48
Chemical Shift (ppm)
O
OEt
Cl
Cl

13

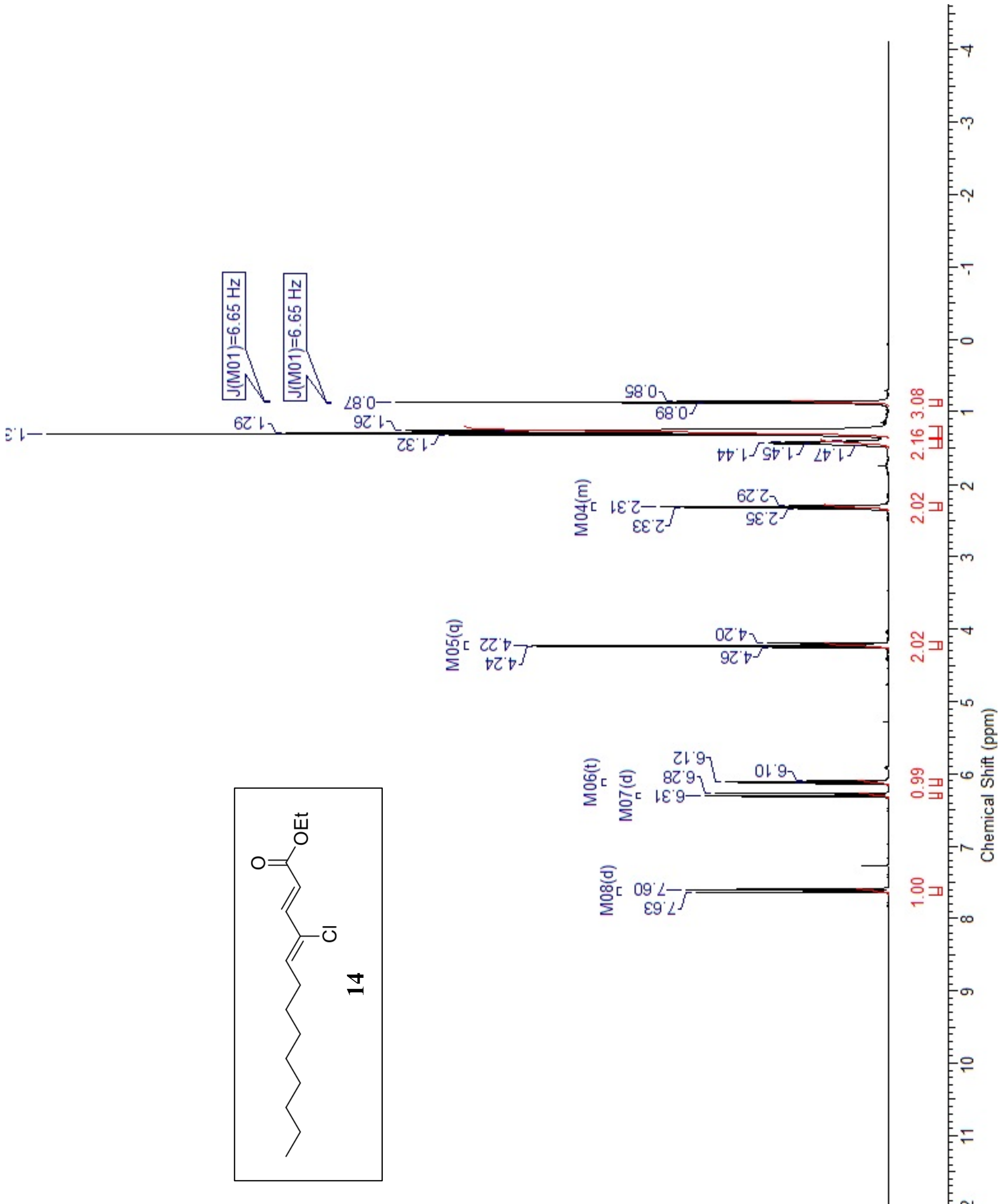

O
OEt
Cl
14
J(M01)=6.65 Hz
J(M01)=6.65 Hz
0.87
0.85
0.89
1.26
1.29
1.32
1.47
1.45
1.44
M04(m)
2.29
2.31
2.33
2.35
M05(q)
4.20
4.22
4.24
4.26
M06(t)
6.10
6.12
M07(d)
6.28
6.31
M08(d)
7.60
7.63
3.08
2.16
2.02
2.02
0.99
1.00
Chemical Shift (ppm)

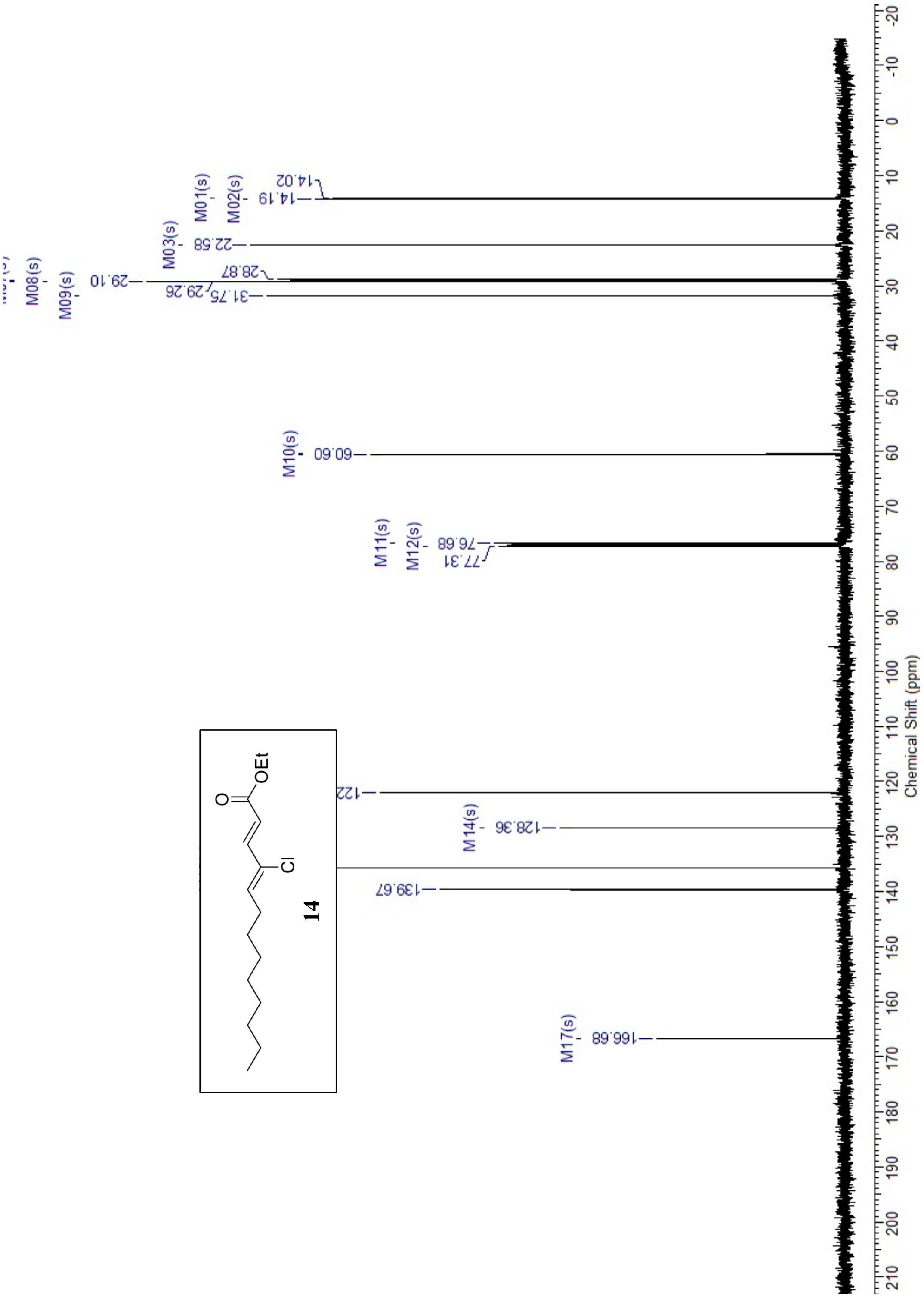

14
OEt
O
Cl
166.68
139.67
128.36
122
77.31
76.68
60.60
31.75
29.26
29.10
28.87
22.58
14.19
14.02
M01(s)
M02(s)
M03(s)
M08(s)
M09(s)
M10(s)
M11(s)
M12(s)
M14(s)
M17(s)
Chemical Shift (ppm)

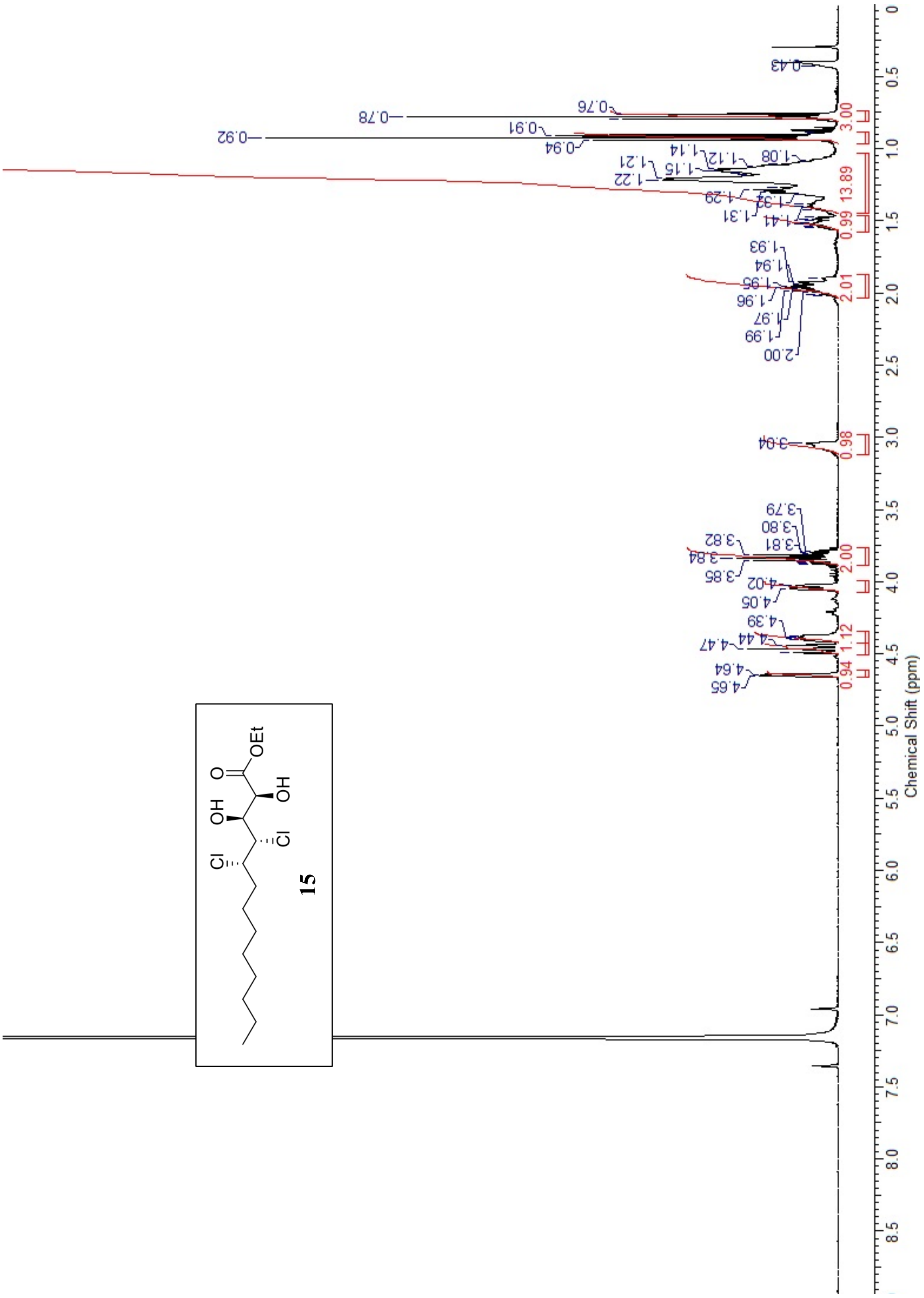

Chemical Shift (ppm)
OEt
OH
Cl
15

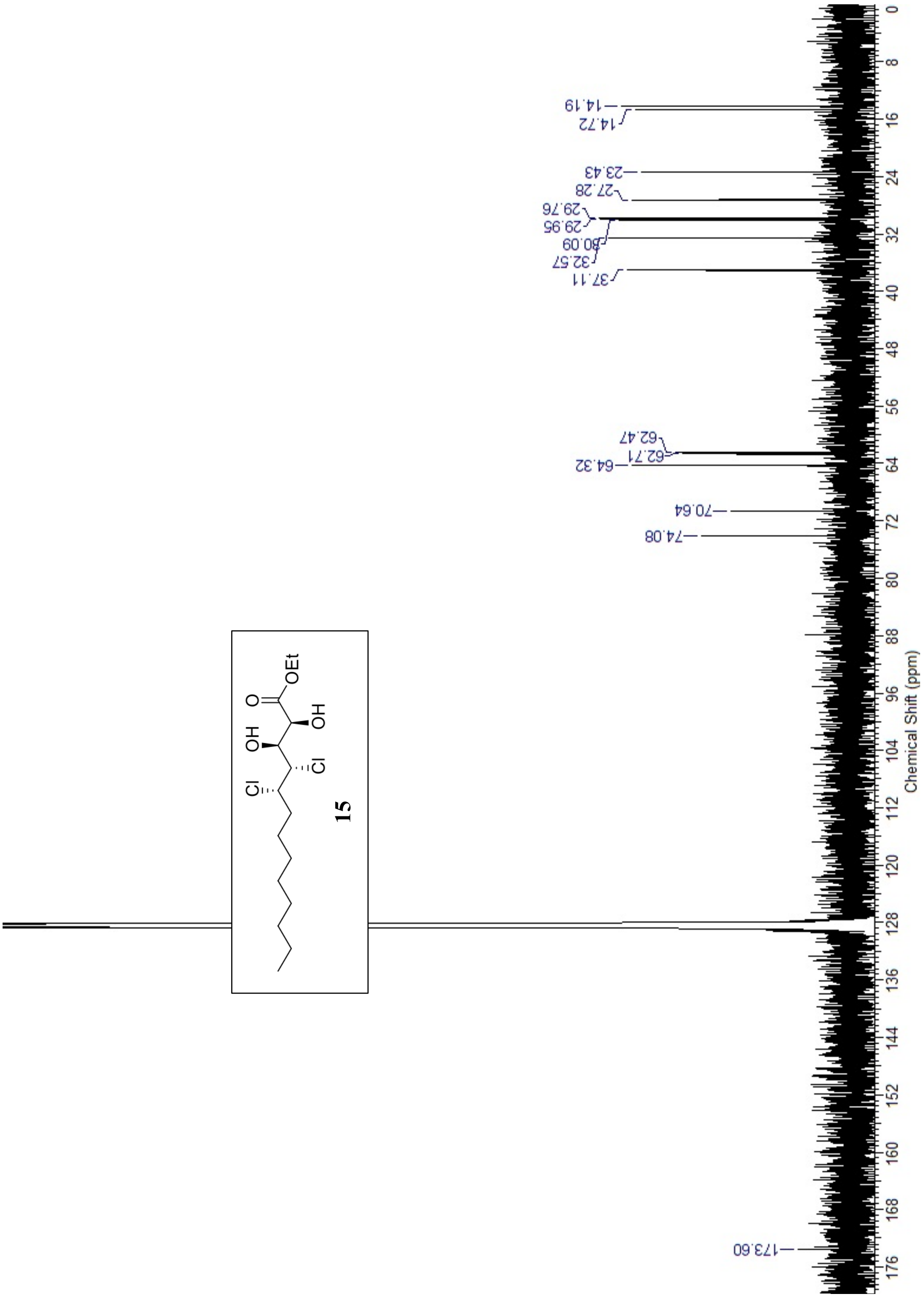

15
OEt
OH
Cl
173.60
74.08
70.64
64.32
62.71
62.47
37.11
32.57
30.09
29.95
29.76
27.28
23.43
14.72
14.19
Chemical Shift (ppm)

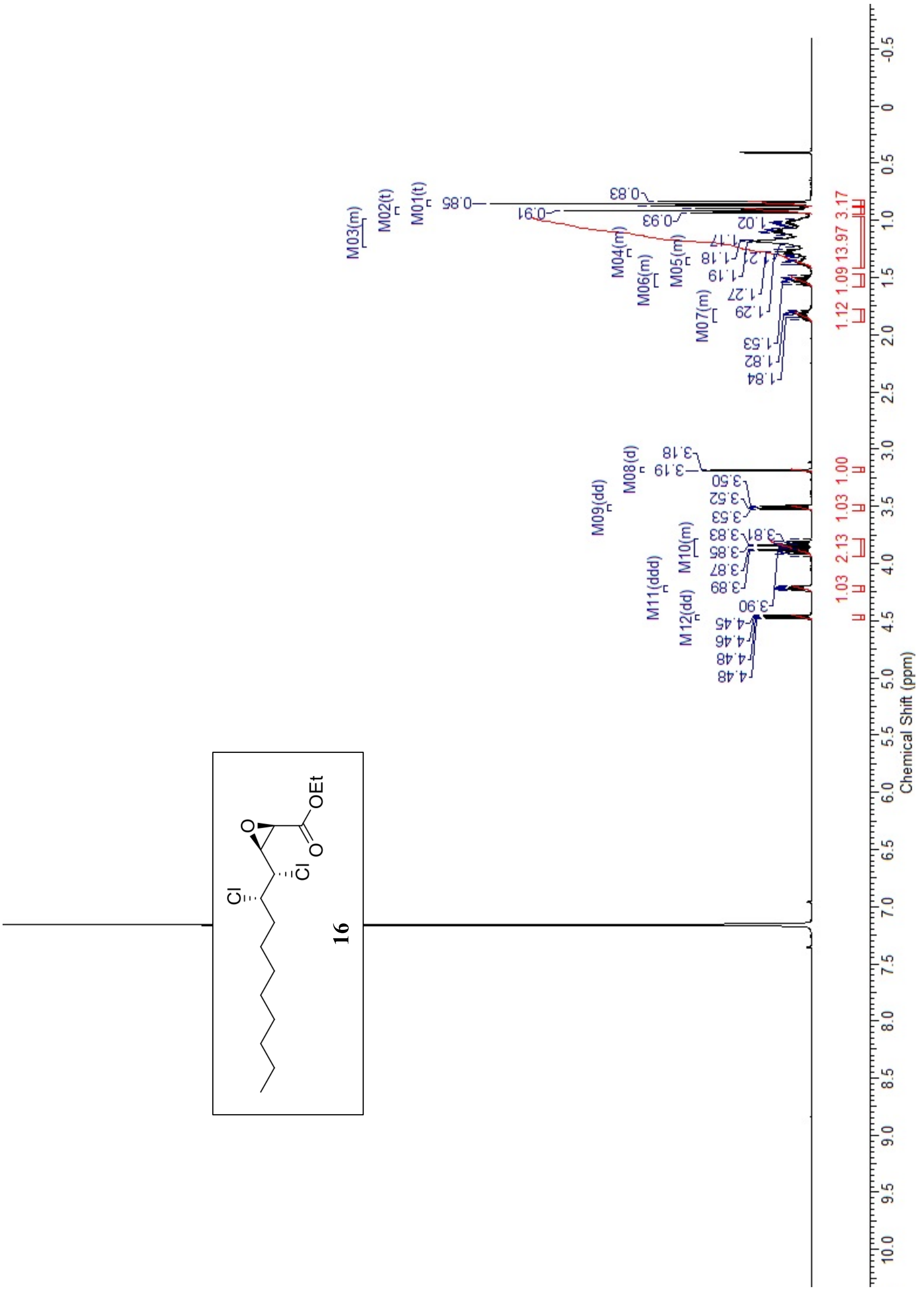
16
OEt
O
Cl
Cl
M01(t)
M02(t)
M03(m)
M04(m)
M05(m)
M06(m)
M07(m)
M08(d)
M09(dd)
M10(m)
M11(ddd)
M12(dd)
1.12 1.09 13.97 3.17
1.00
1.03
2.13
1.03
Chemical Shift (ppm)

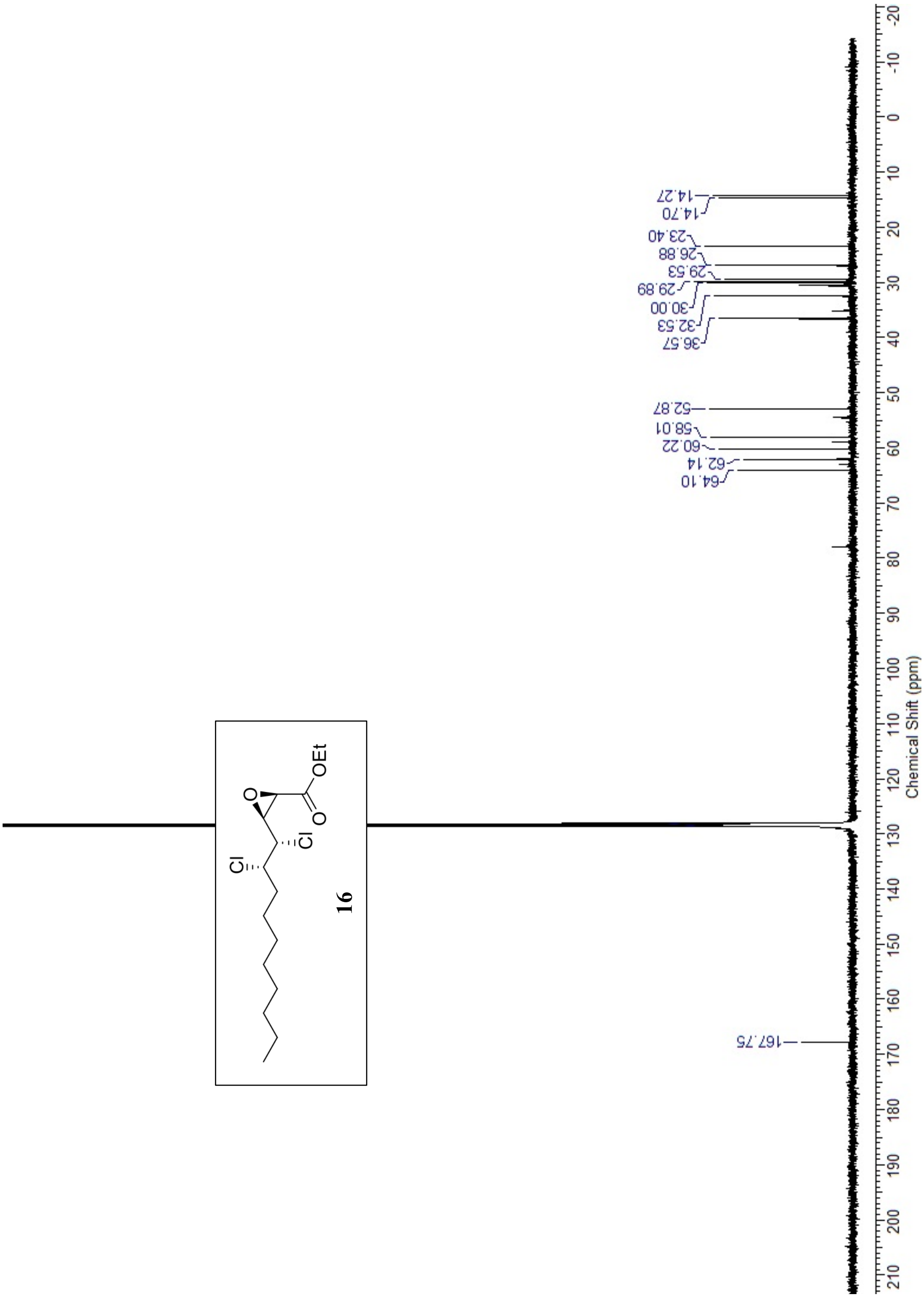
16
OEt
Cl
Cl
O
14.27
14.70
23.40
26.88
29.53
29.89
30.00
32.53
36.57
52.87
58.01
60.22
62.14
64.10
167.75
Chemical Shift (ppm)

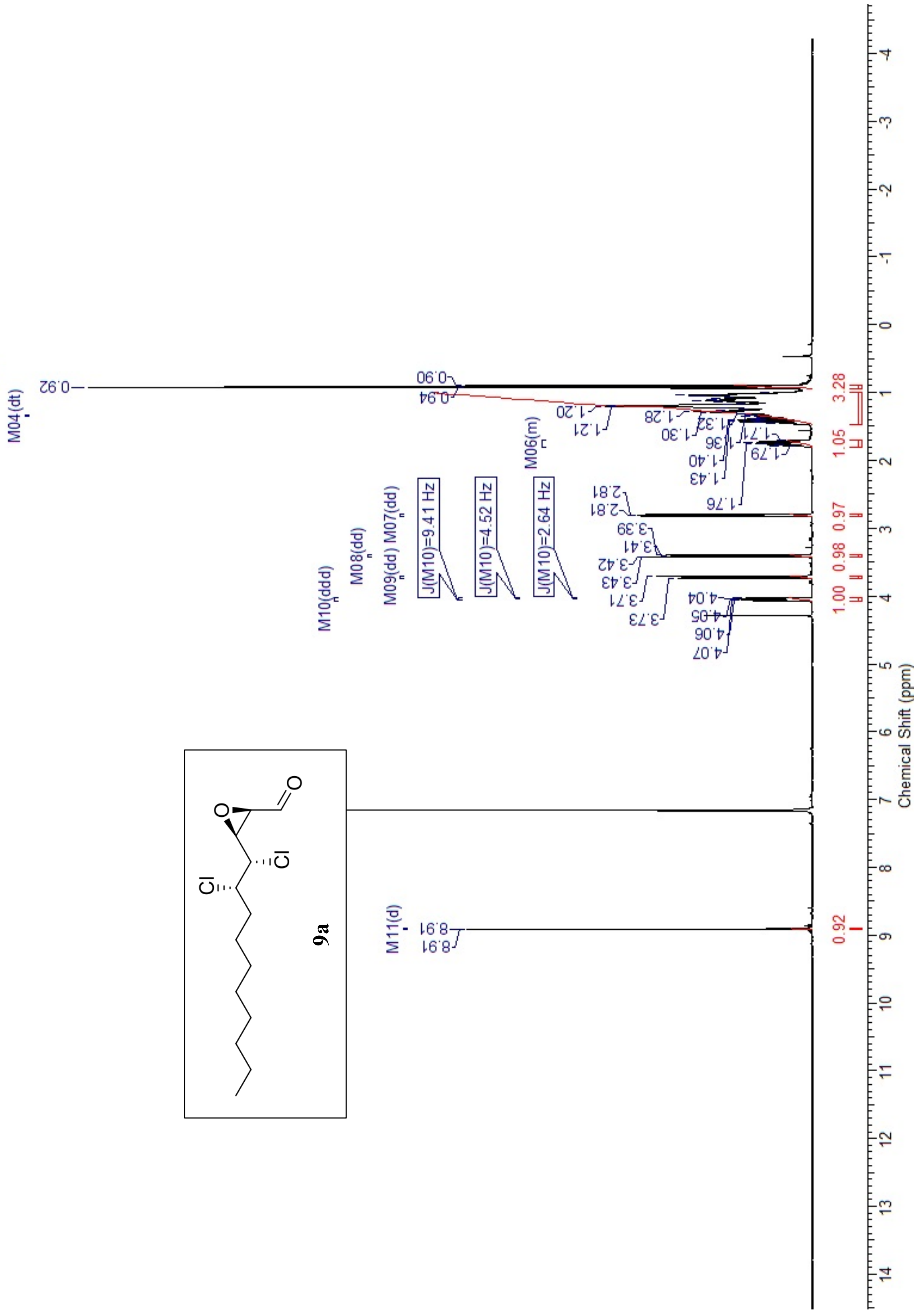

9a
Chemical Shift (ppm)
M04(dt)
M06(m)
M07(dd)
M08(dd)
M09(dd)
M10(ddd)
M11(d)
J(M10)=9.41 Hz
J(M10)=4.52 Hz
J(M10)=2.64 Hz

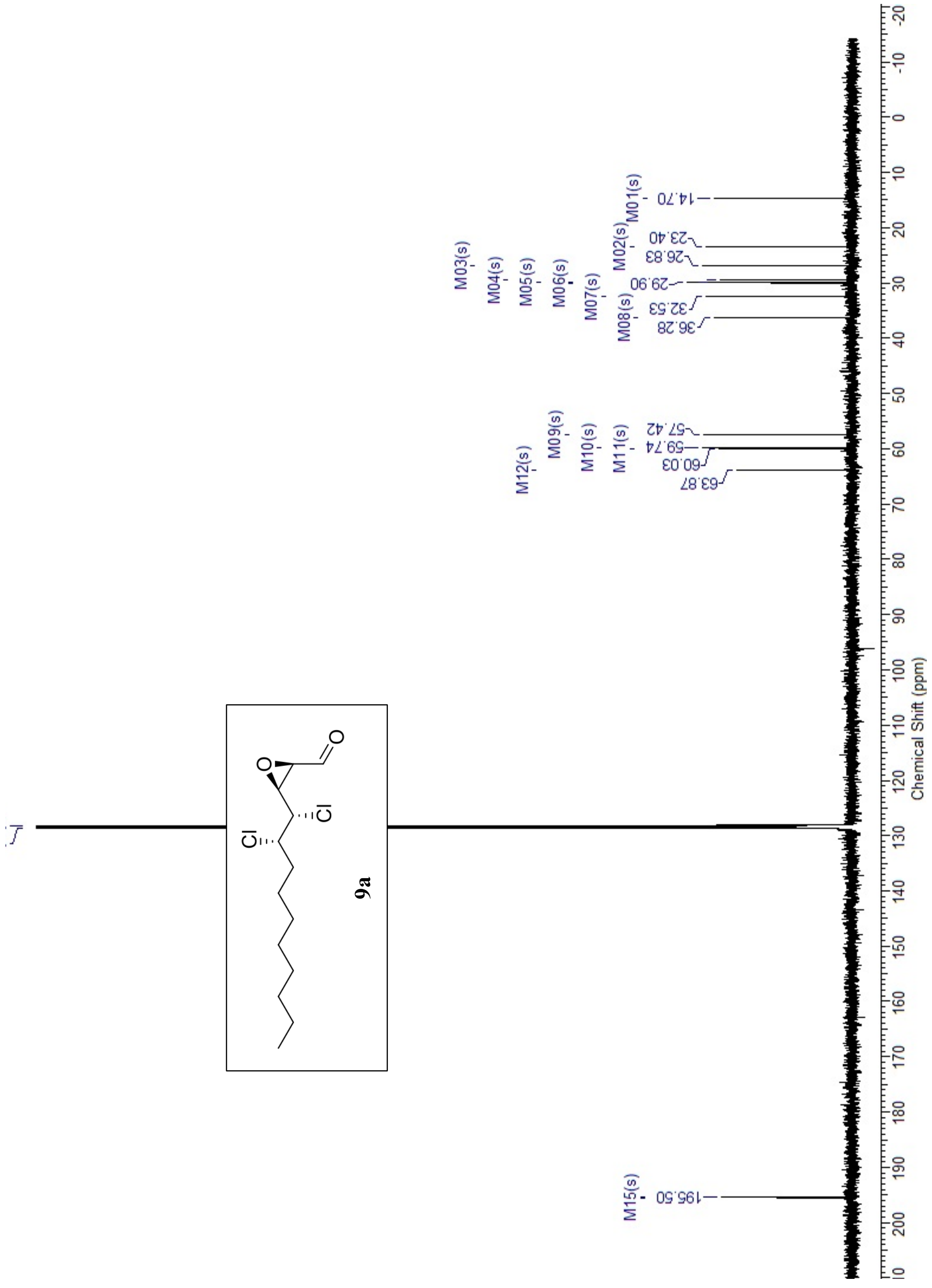
9a
Cl
Cl
O
O
M01(s) 14.70
M02(s) 23.40
26.83
M03(s)
M04(s)
M05(s)
M06(s)
M07(s) 29.90
M08(s) 32.53
36.28
M09(s) 57.42
M10(s) 59.74
M11(s) 60.03
M12(s) 63.87
M15(s) 195.50
Chemical Shift (ppm)

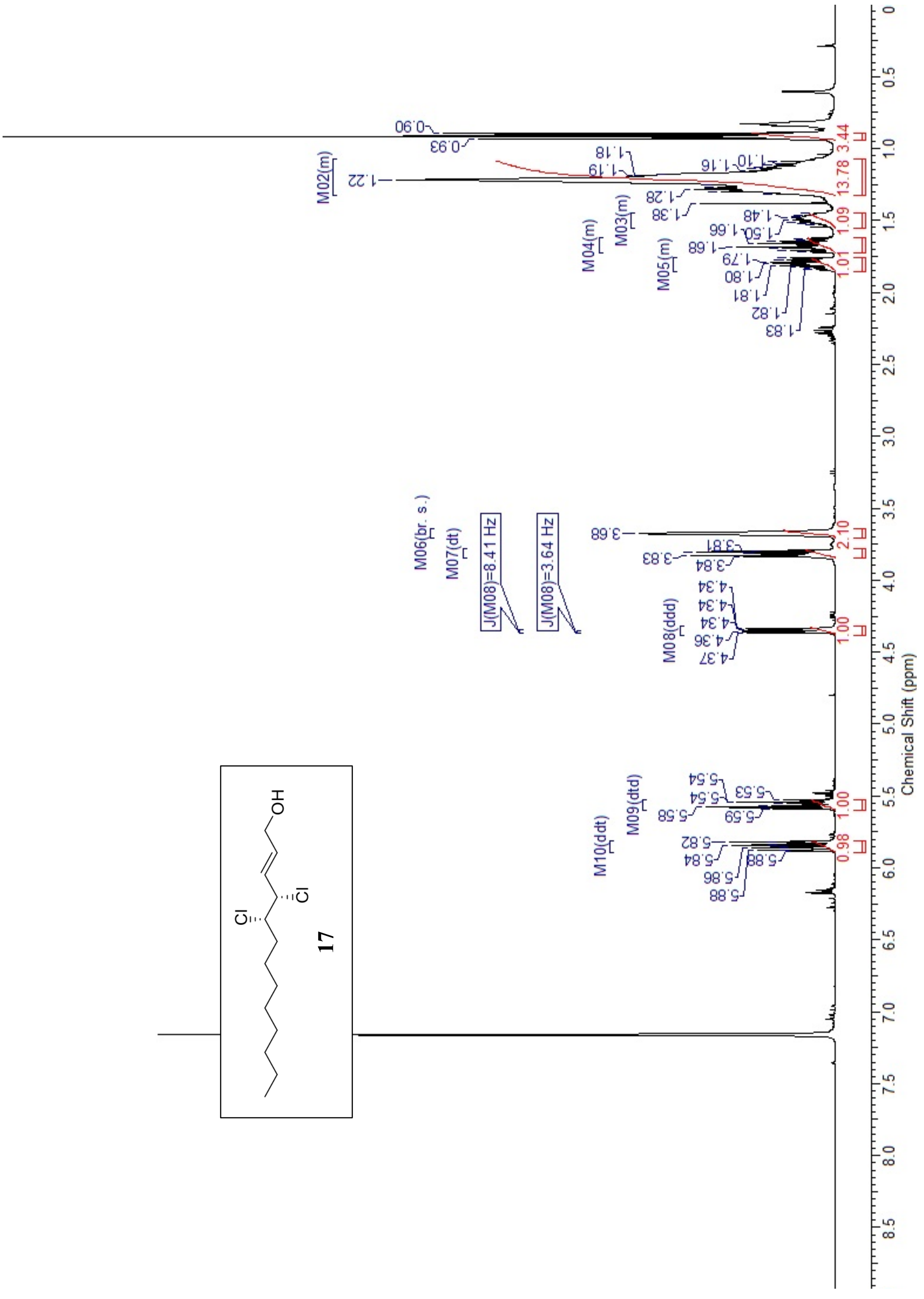

17
Cl
Cl
OH
M02(m)
M03(m)
M04(m)
M05(m)
M06(br. s.)
M07(dt)
M08(ddd)
M09(dtd)
M10(ddt)
J(M08)=8.41 Hz
J(M08)=3.64 Hz
0.90
0.93
1.10
1.16
1.18
1.19
1.22
1.28
1.38
1.48
1.50
1.66
1.68
1.79
1.80
1.81
1.82
1.83
3.68
3.81
3.83
3.84
4.34
4.34
4.34
4.36
4.37
5.53
5.54
5.54
5.58
5.59
5.82
5.84
5.86
5.88
5.88
3.44
13.78
1.09
1.01
2.10
1.00
1.00
0.98
Chemical Shift (ppm)

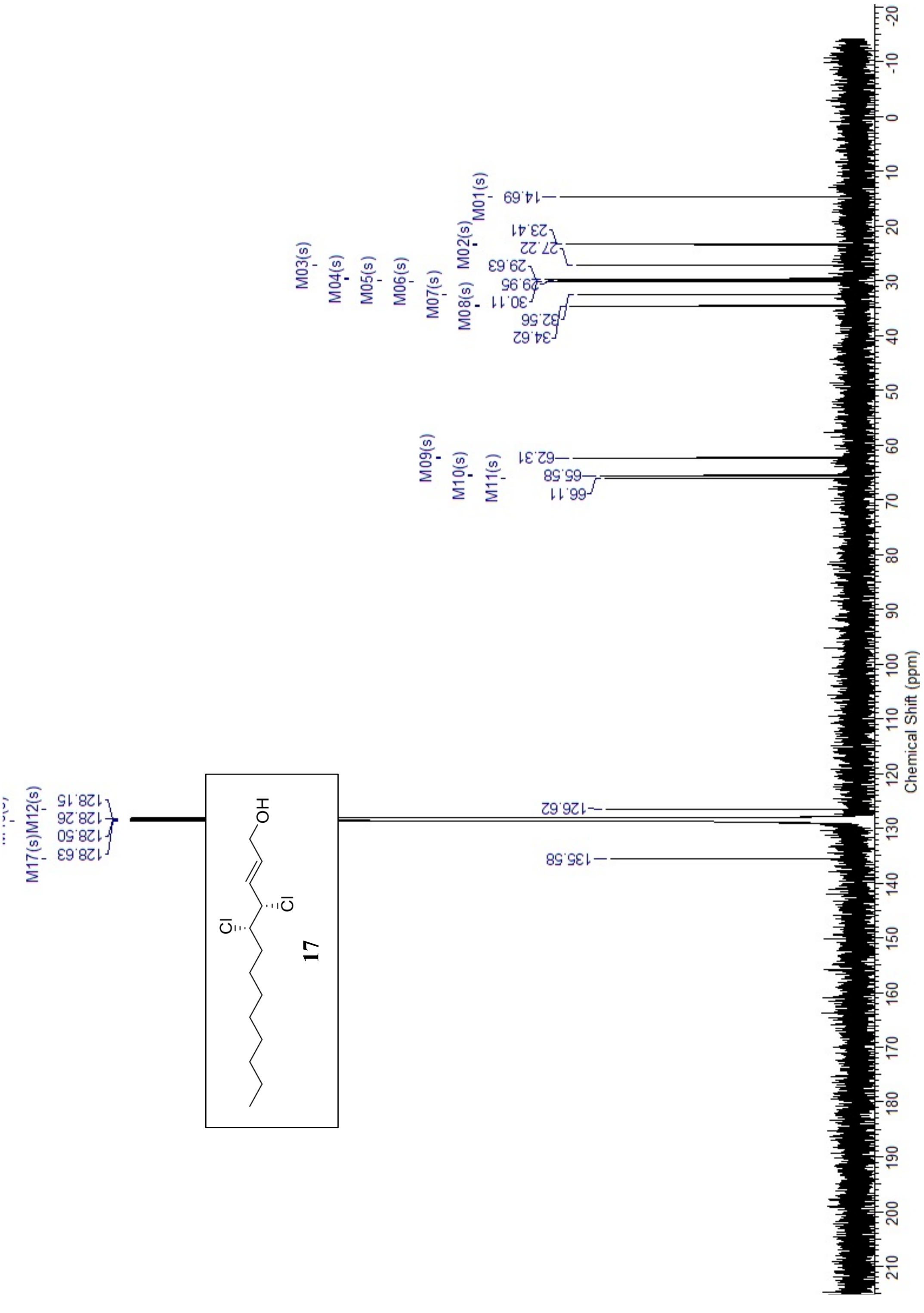
14.69
23.41
27.22
29.63
29.95
30.11
32.56
34.62
62.31
65.58
66.11
126.62
128.15
128.26
128.50
128.63
135.58
Chemical Shift (ppm)
Cl
Cl
OH
17

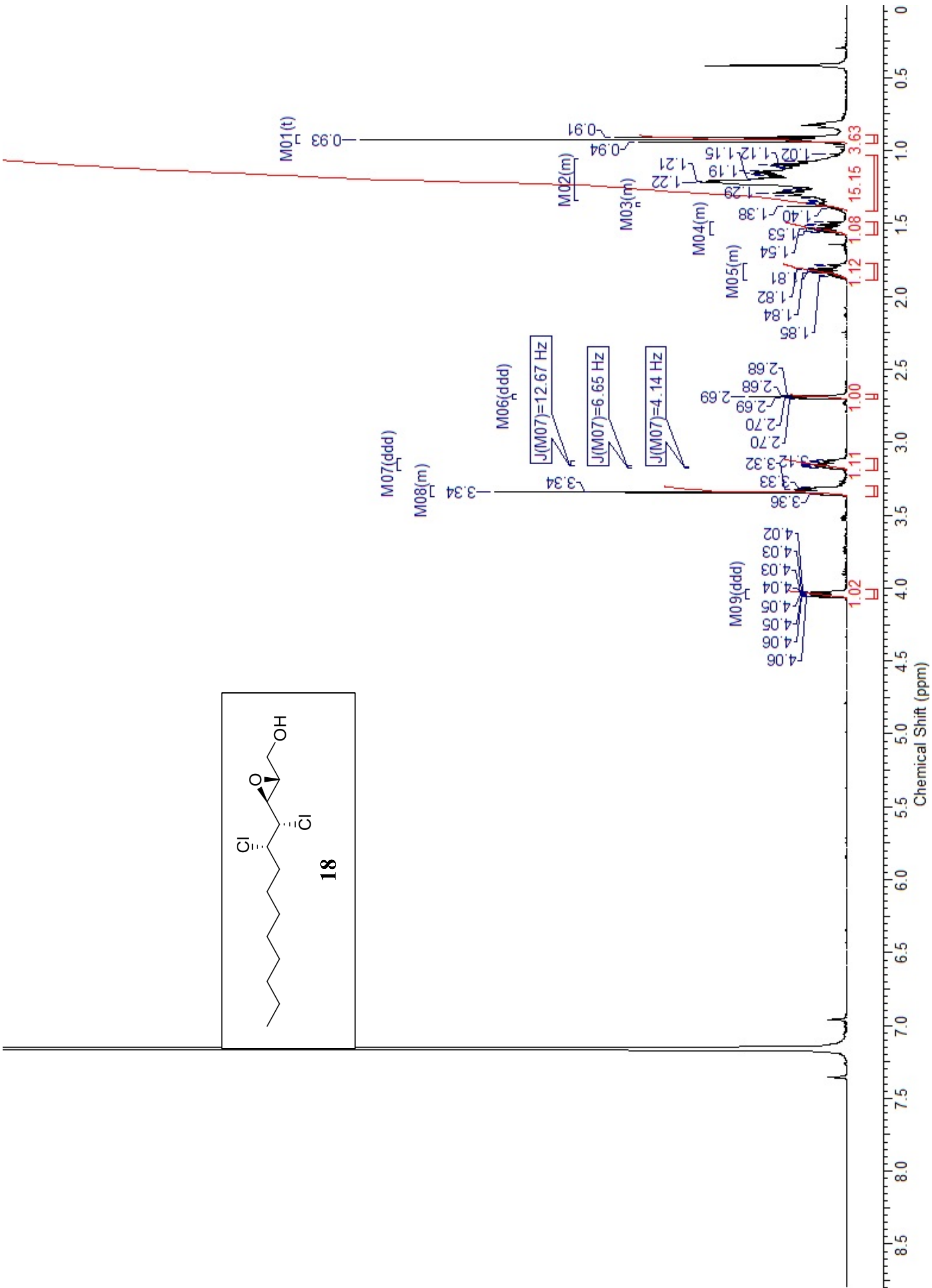

Chemical Shift (ppm)
18

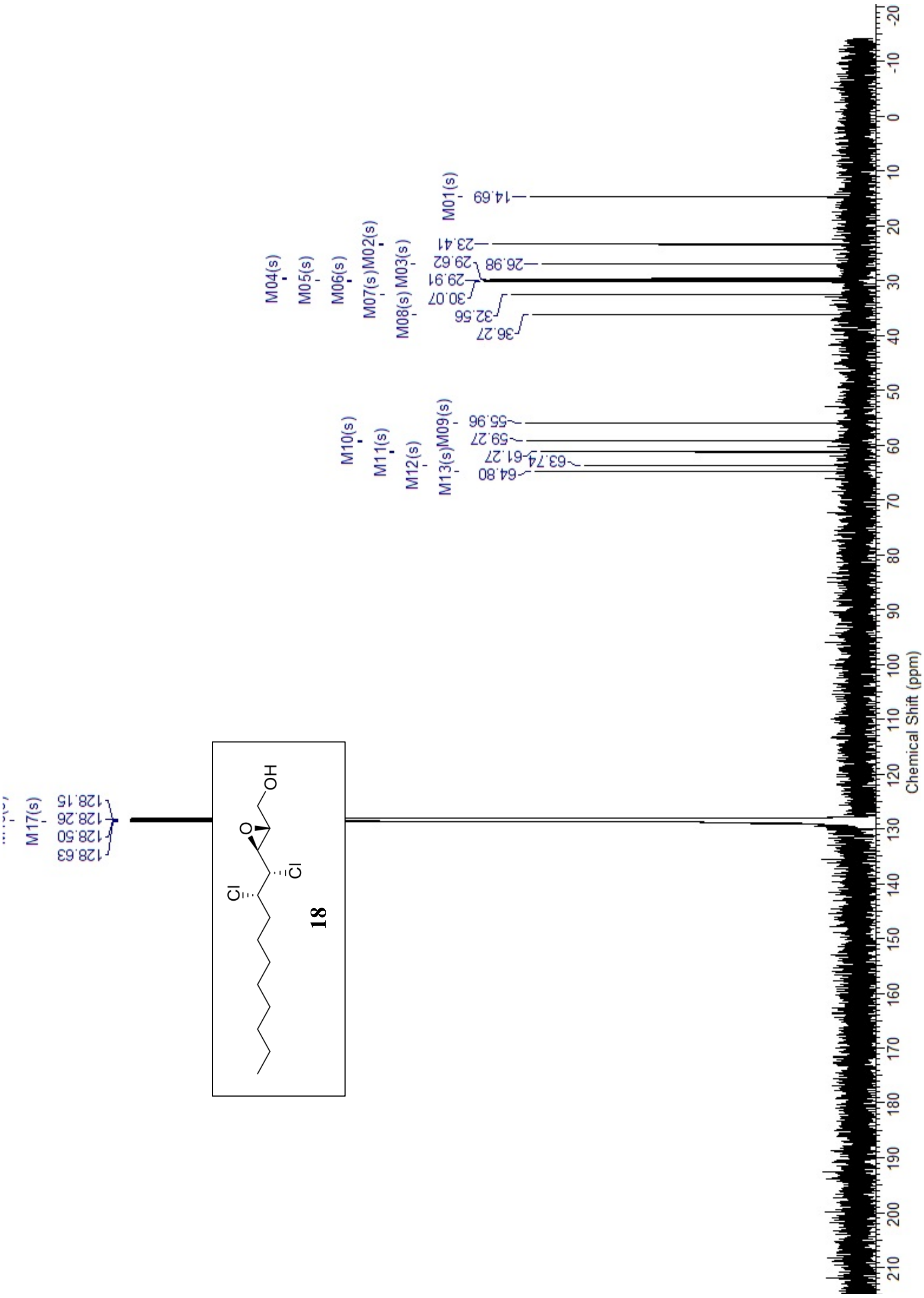
M01(s)
14.69
M02(s)
M03(s)
M04(s)
M05(s)
M06(s)
M07(s)
M08(s)
23.41
26.98
29.62
29.91
30.07
32.56
36.27
M09(s)
M10(s)
M11(s)
M12(s)
M13(s)
55.96
59.27
61.27
63.74
64.80
M17(s)
128.15
128.26
128.50
128.63
Cl
Cl
O
OH
18
Chemical Shift (ppm)

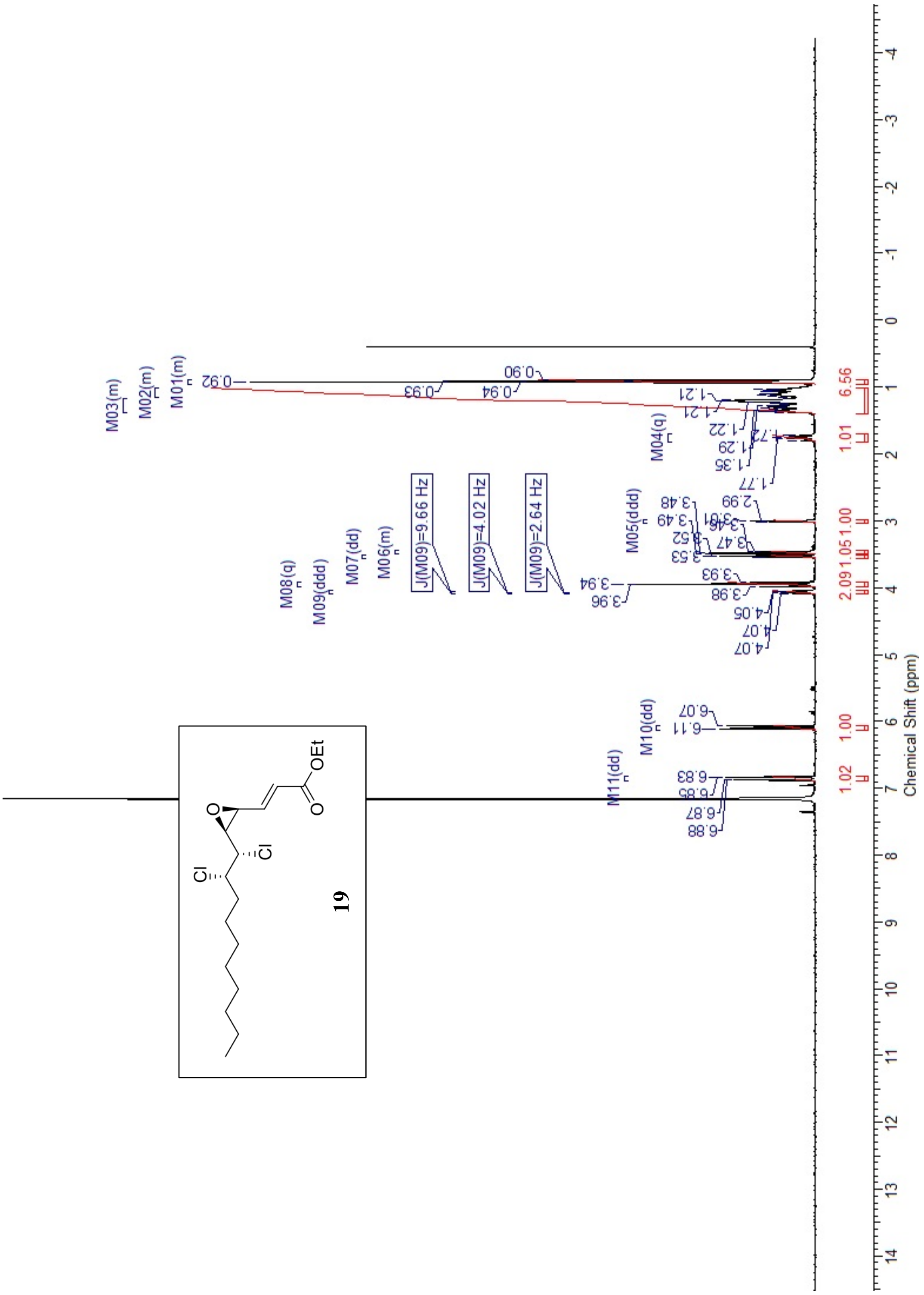
19
OEt
Cl
Cl
O
M01(m)
M02(m)
M03(m)
M04(q)
M05(ddd)
M06(m)
M07(dd)
M08(q)
M09(ddd)
M10(dd)
M11(dd)
J(M09)=9.66 Hz
J(M09)=4.02 Hz
J(M09)=2.64 Hz
6.56
1.01
1.00
1.05
1.09
2.09
1.00
1.02
Chemical Shift (ppm)

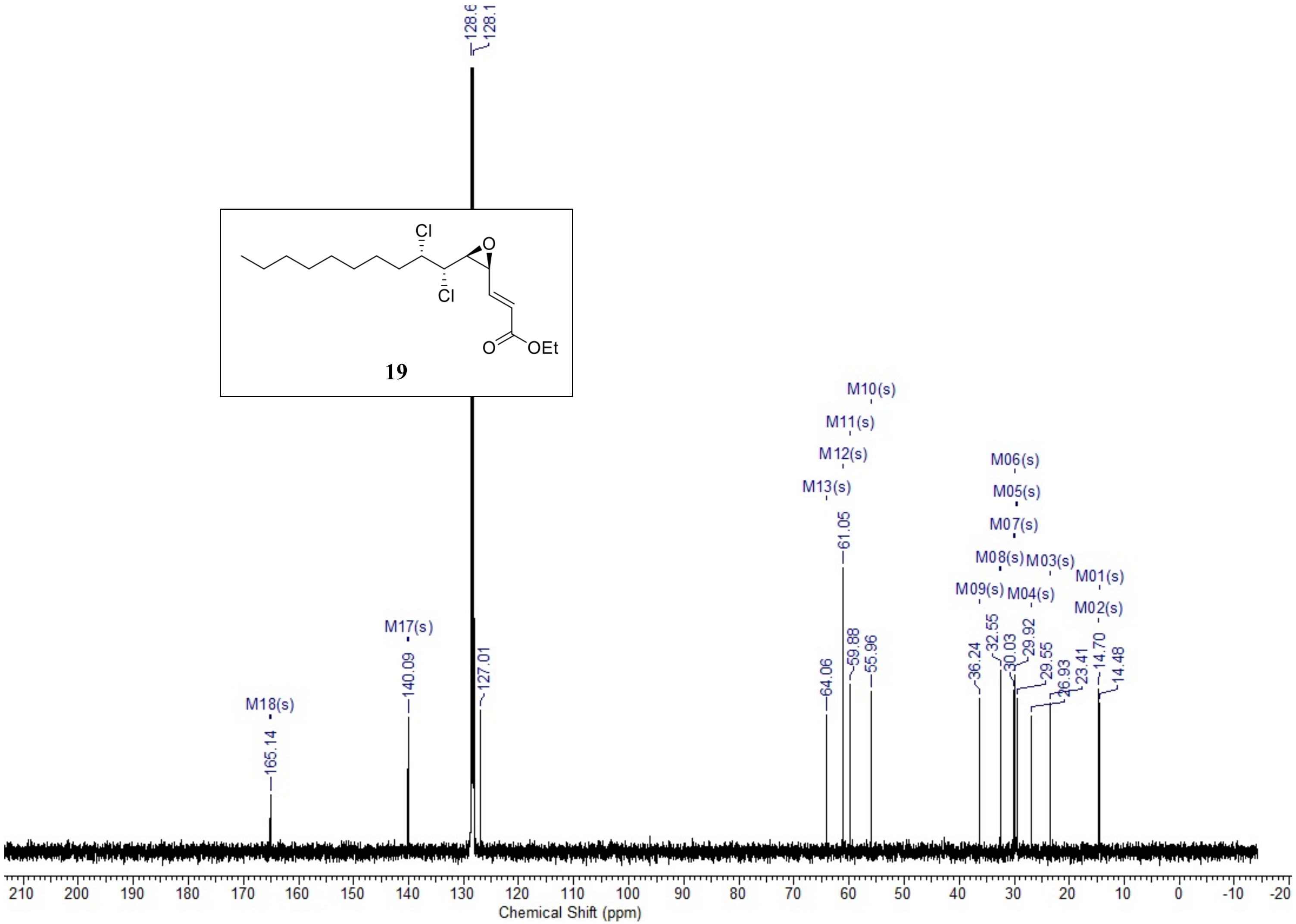

Cl
Cl
O
O
OEt
19
128.6
128.1
M18(s)
165.14
M17(s)
140.09
127.01
64.06
61.05
59.88
55.96
M10(s)
M11(s)
M12(s)
M13(s)
M06(s)
M05(s)
M07(s)
M08(s)
M03(s)
M09(s)
M04(s)
M01(s)
M02(s)
36.24
32.55
30.03
29.92
29.55
26.93
23.41
14.70
14.48
Chemical Shift (ppm)

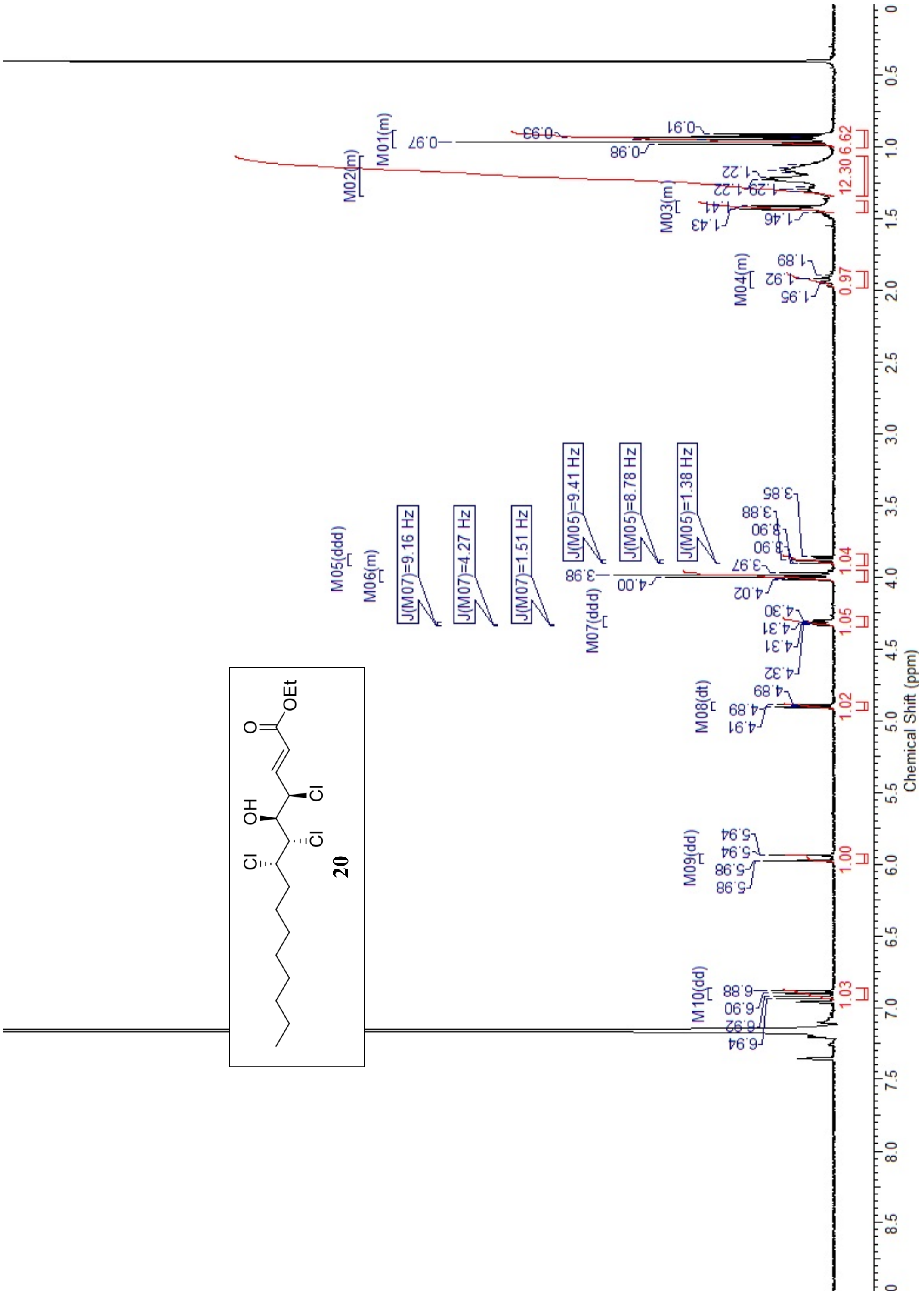
Chemical Shift (ppm)
M01(m)
M02(m)
M03(m)
M04(m)
M05(ddd)
M06(m)
M07(ddd)
M08(dt)
M09(dd)
M10(dd)
J(M07)=9.16 Hz
J(M07)=4.27 Hz
J(M07)=1.51 Hz
J(M05)=9.41 Hz
J(M05)=8.78 Hz
J(M05)=1.38 Hz
6.62
12.30
0.97
1.04
1.05
1.02
1.00
1.03
OH
Cl
Cl
Cl
O
OEt
20

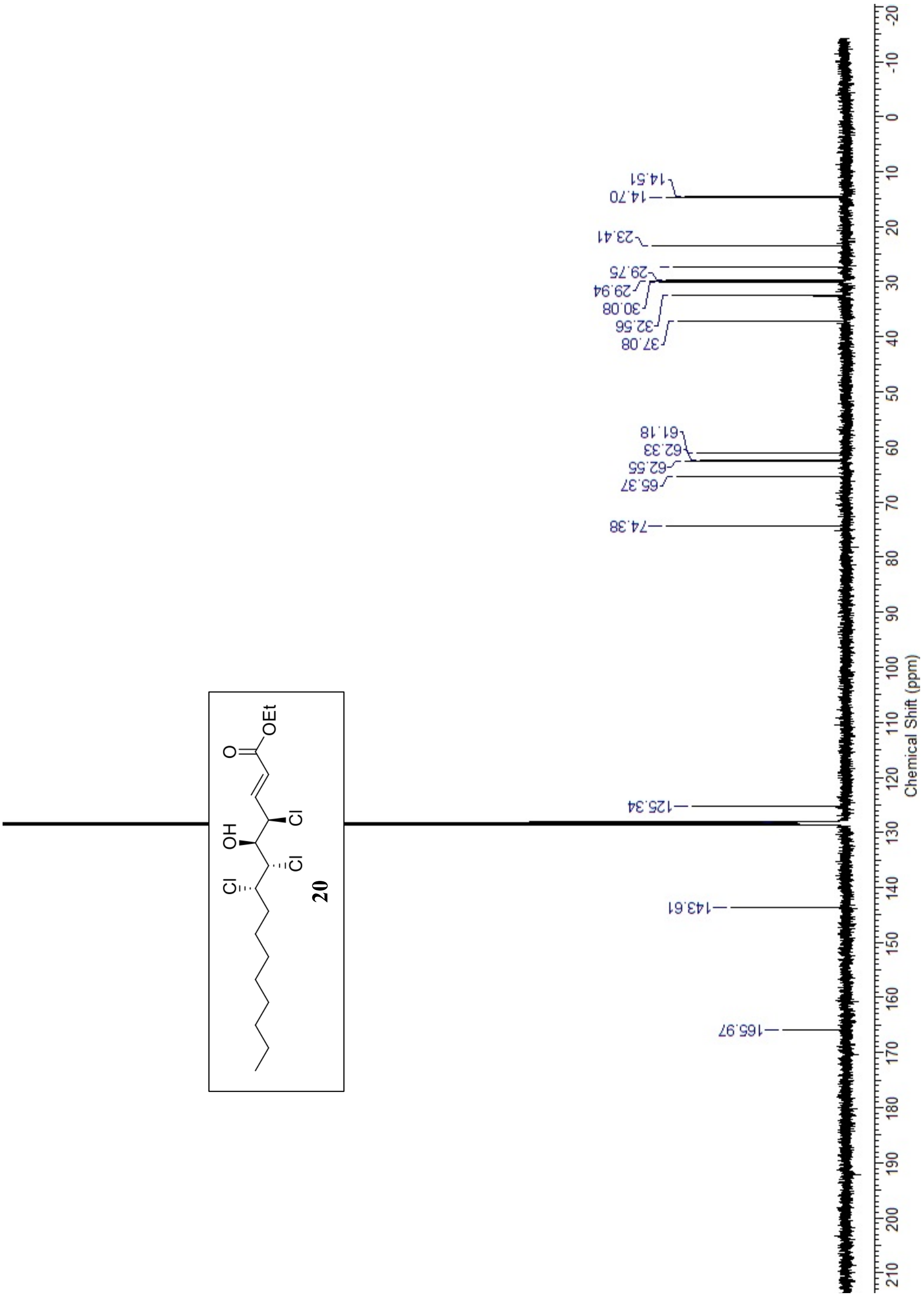

14.51
14.70
23.41
29.75
29.94
30.08
32.56
37.08
61.18
62.33
62.55
65.37
74.38
125.34
143.61
165.97
Chemical Shift (ppm)
O
OEt
OH
Cl
Cl
Cl
20

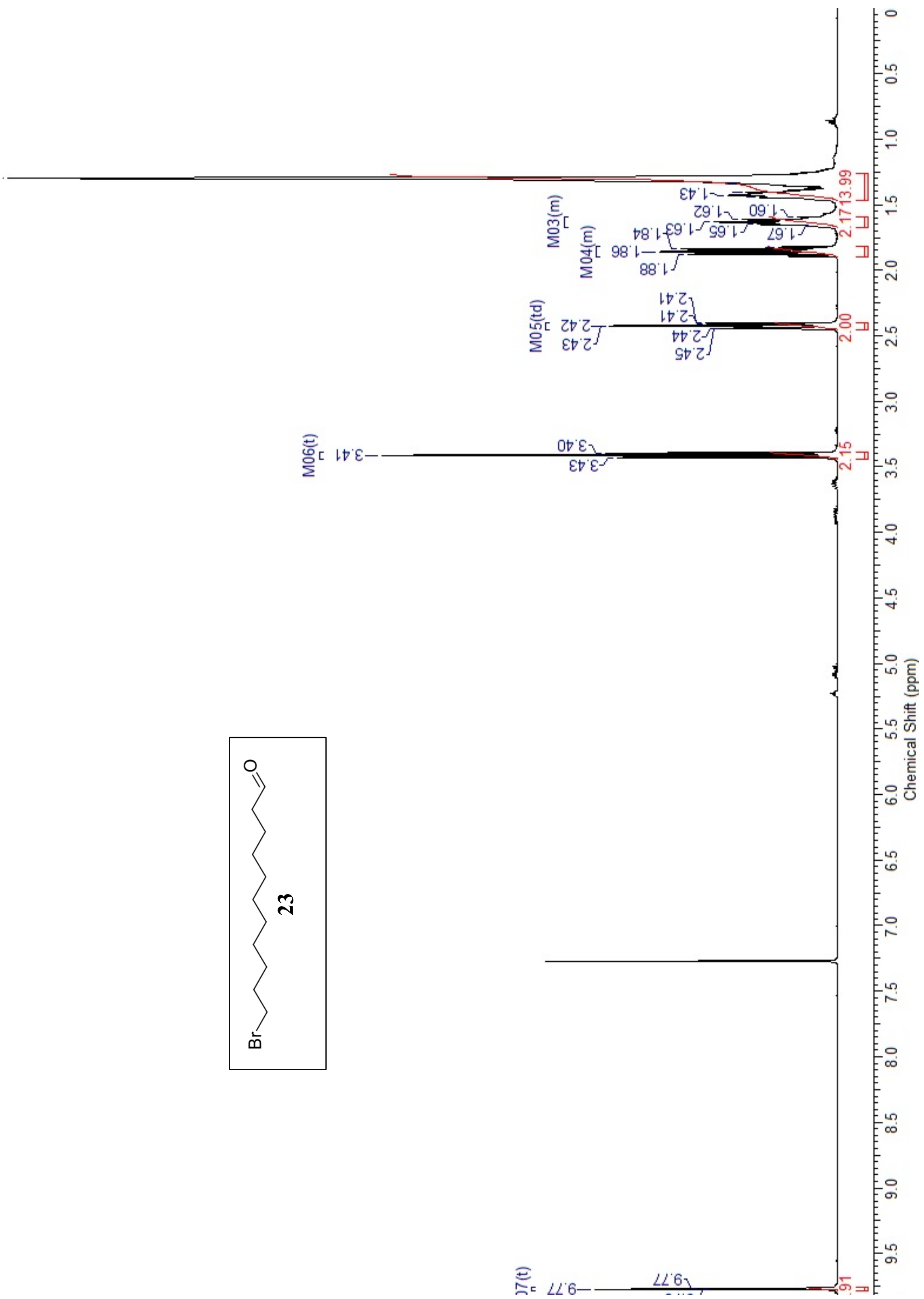

Br
O
23
M03(m)
M04(m)
M05(td)
M06(t)
9.77
3.41
3.40
3.43
2.42
2.43
2.41
2.41
2.44
2.45
1.43
1.62
1.60
1.65
1.63
1.67
1.84
1.86
1.88
2.15
2.00
2.17
13.99
Chemical Shift (ppm)

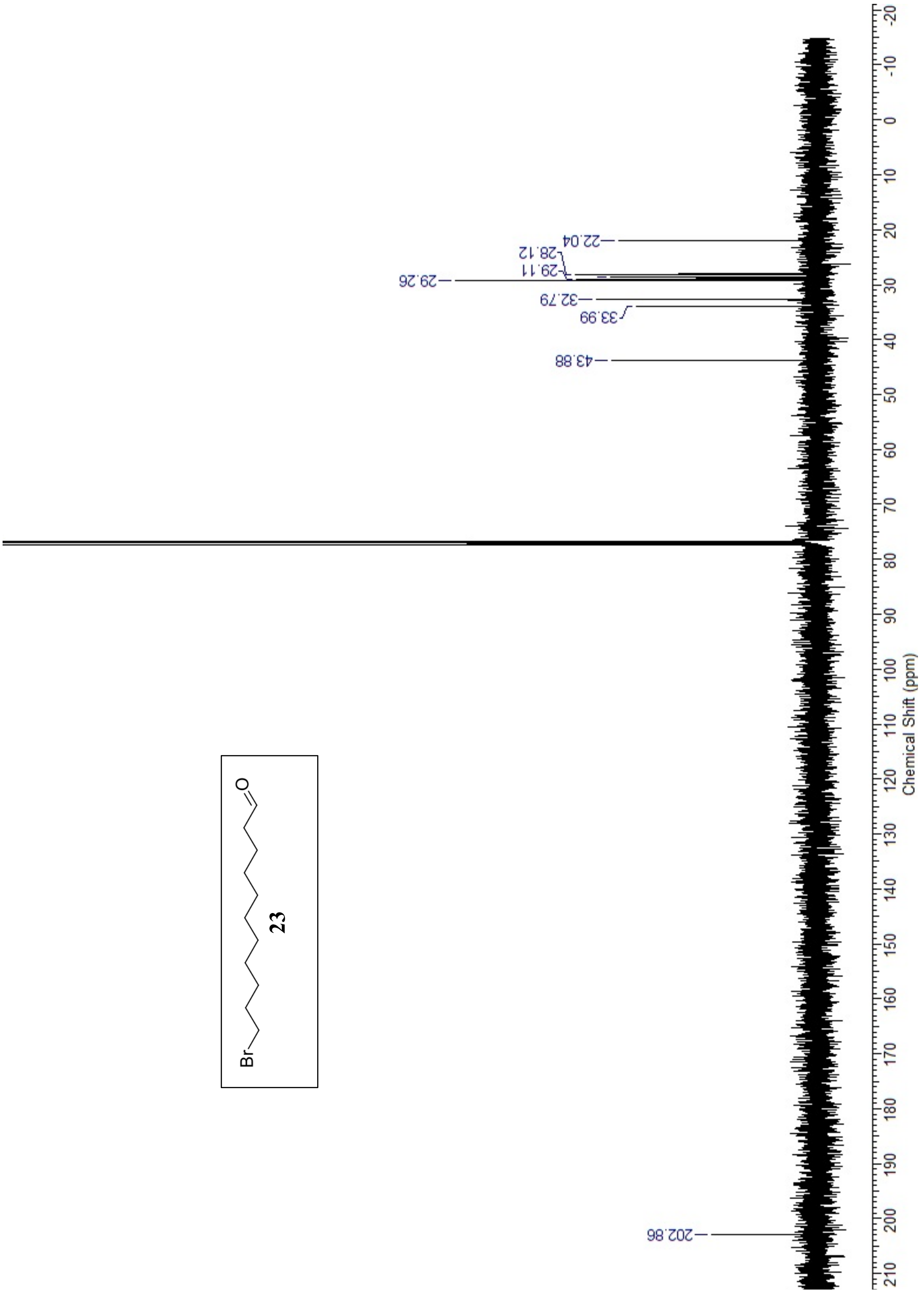
202.86
43.88
33.99
32.79
29.26
29.11
28.12
22.04
Chemical Shift (ppm)
Br
O
23

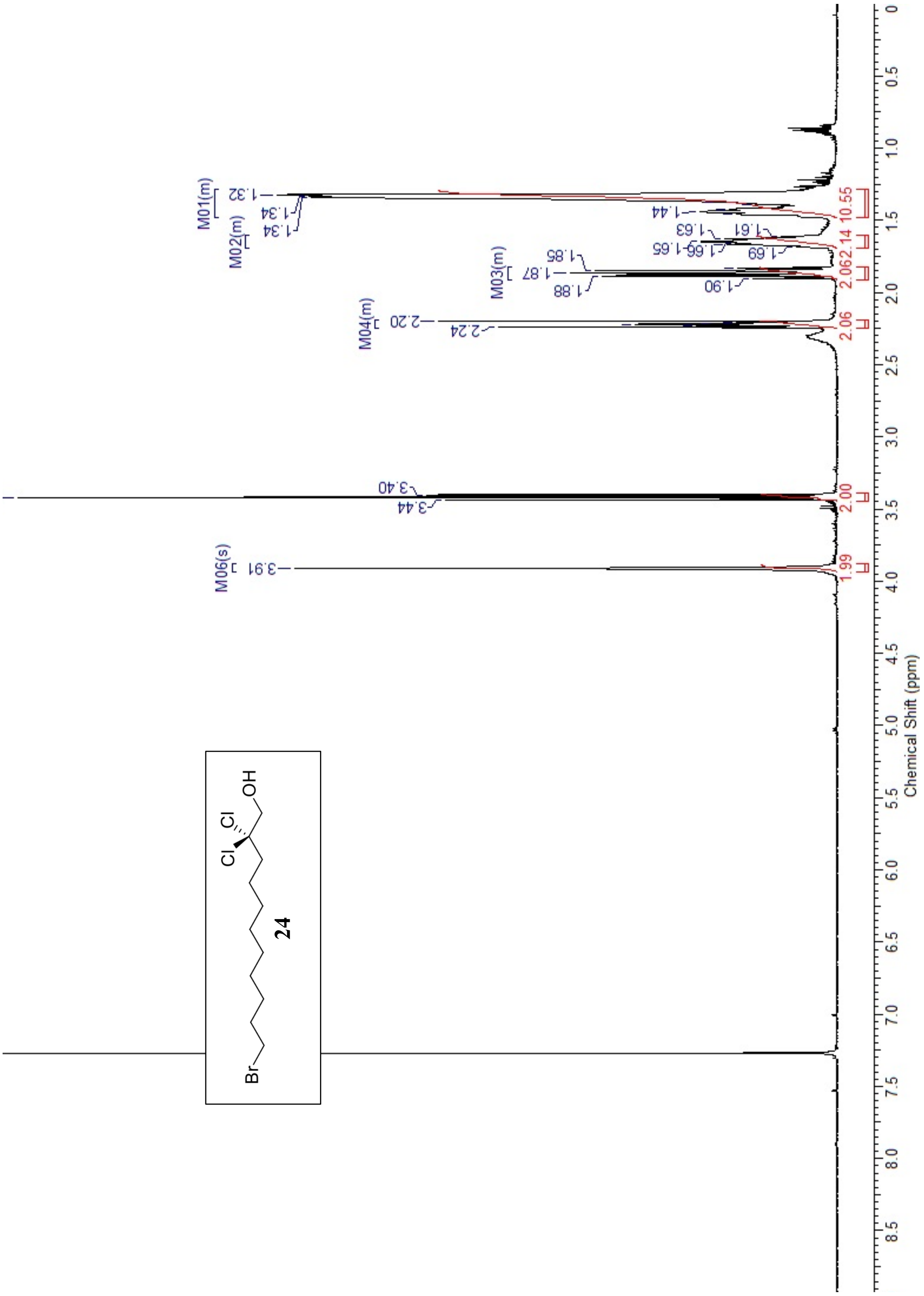

Br
Cl
Cl
OH
24
M01(m)
1.32
1.34
1.34
M02(m)
1.44
1.61
1.63
1.65
1.66
1.69
M03(m)
1.85
1.87
1.88
1.90
M04(m)
2.20
2.24
3.40
3.44
M06(s)
3.91
10.55
2.14
2.06
2.06
2.00
1.99
Chemical Shift (ppm)

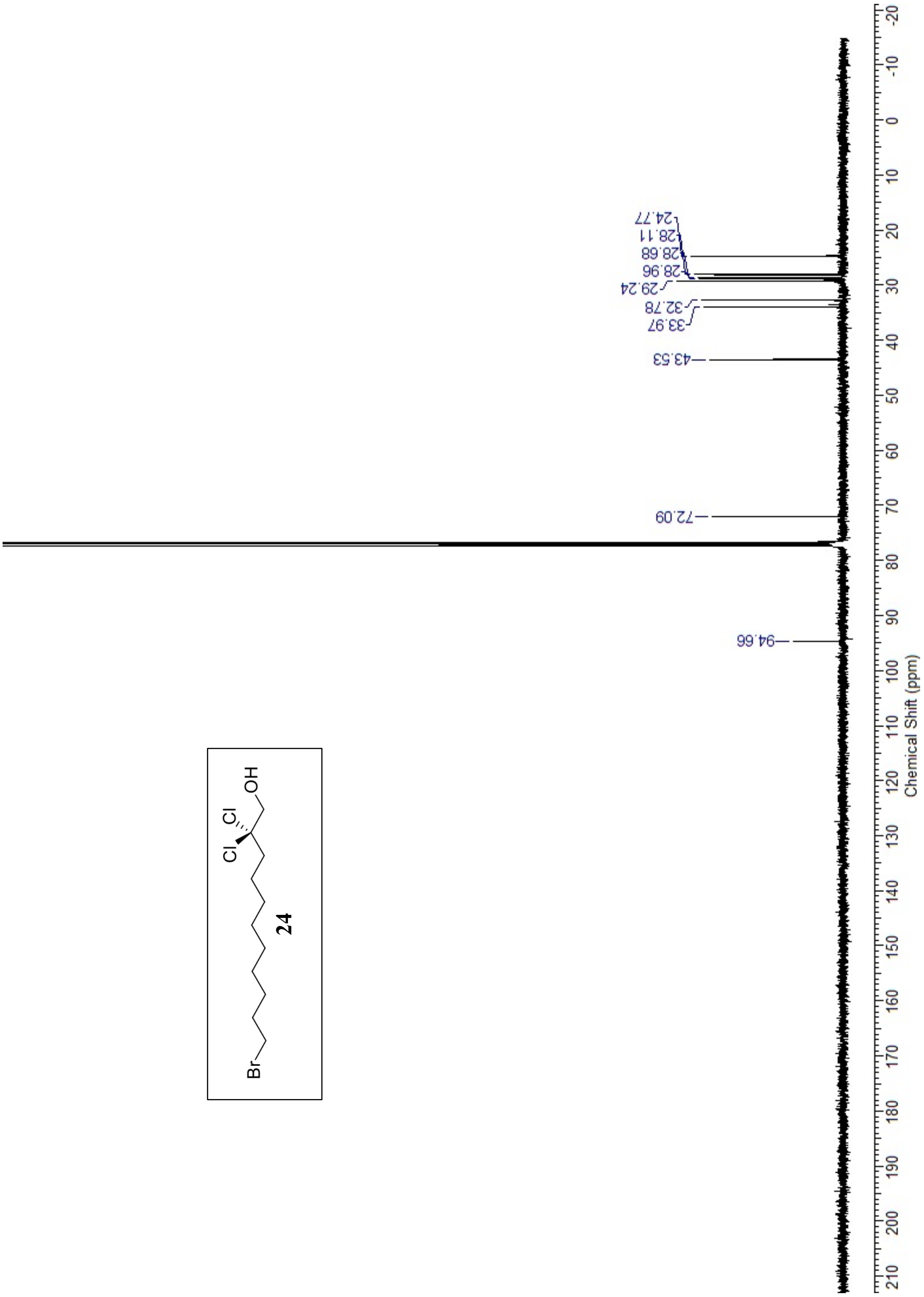
Br
Cl
Cl
OH
24
24.77
28.11
28.68
28.96
29.24
32.78
33.97
43.53
72.09
94.66
Chemical Shift (ppm)

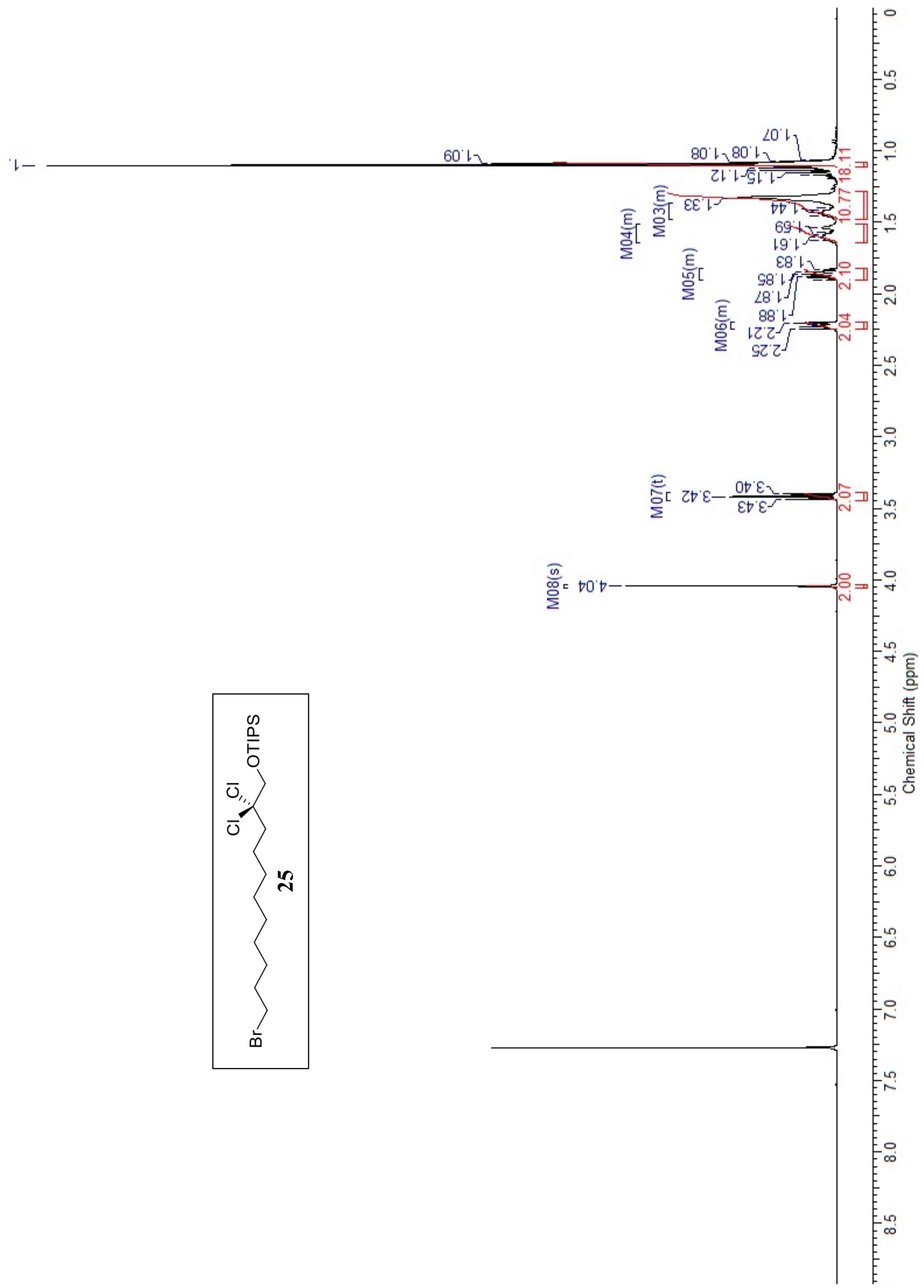
Br
Cl
Cl
OTIPS
25
M08(s)
4.04
M07(t)
3.40
3.42
3.43
M06(m)
2.21
2.25
M05(m)
1.83
1.85
1.87
1.88
M04(m)
M03(m)
1.33
1.44
1.61
1.69
1.07
1.08
1.08
1.09
1.12
1.15
2.00
2.07
2.04
2.10
10.77
18.11
Chemical Shift (ppm)

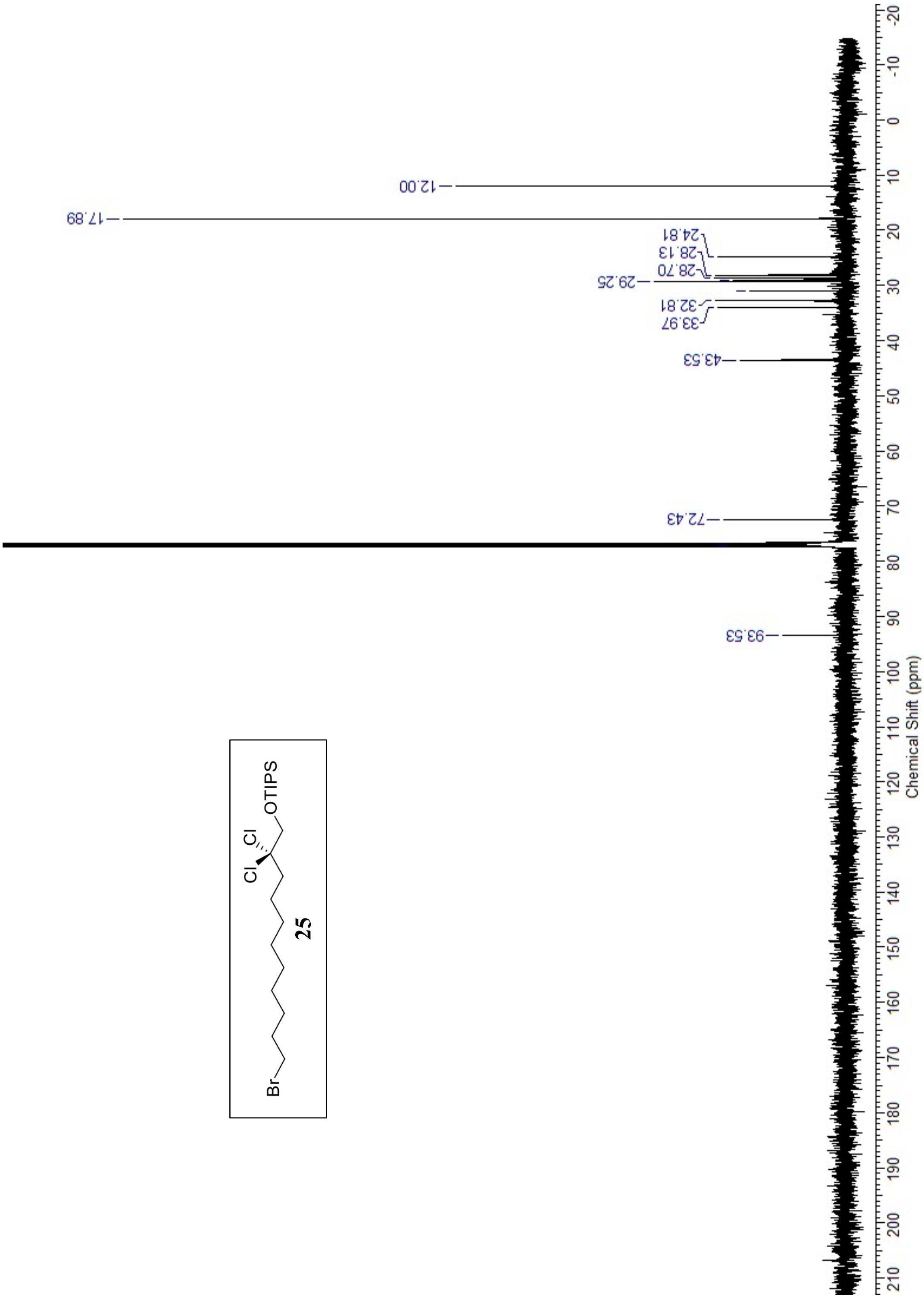

12.00
17.89
24.81
28.13
28.70
29.25
32.81
33.97
43.53
72.43
93.53
Chemical Shift (ppm)
Br
Cl
Cl
OTIPS
25

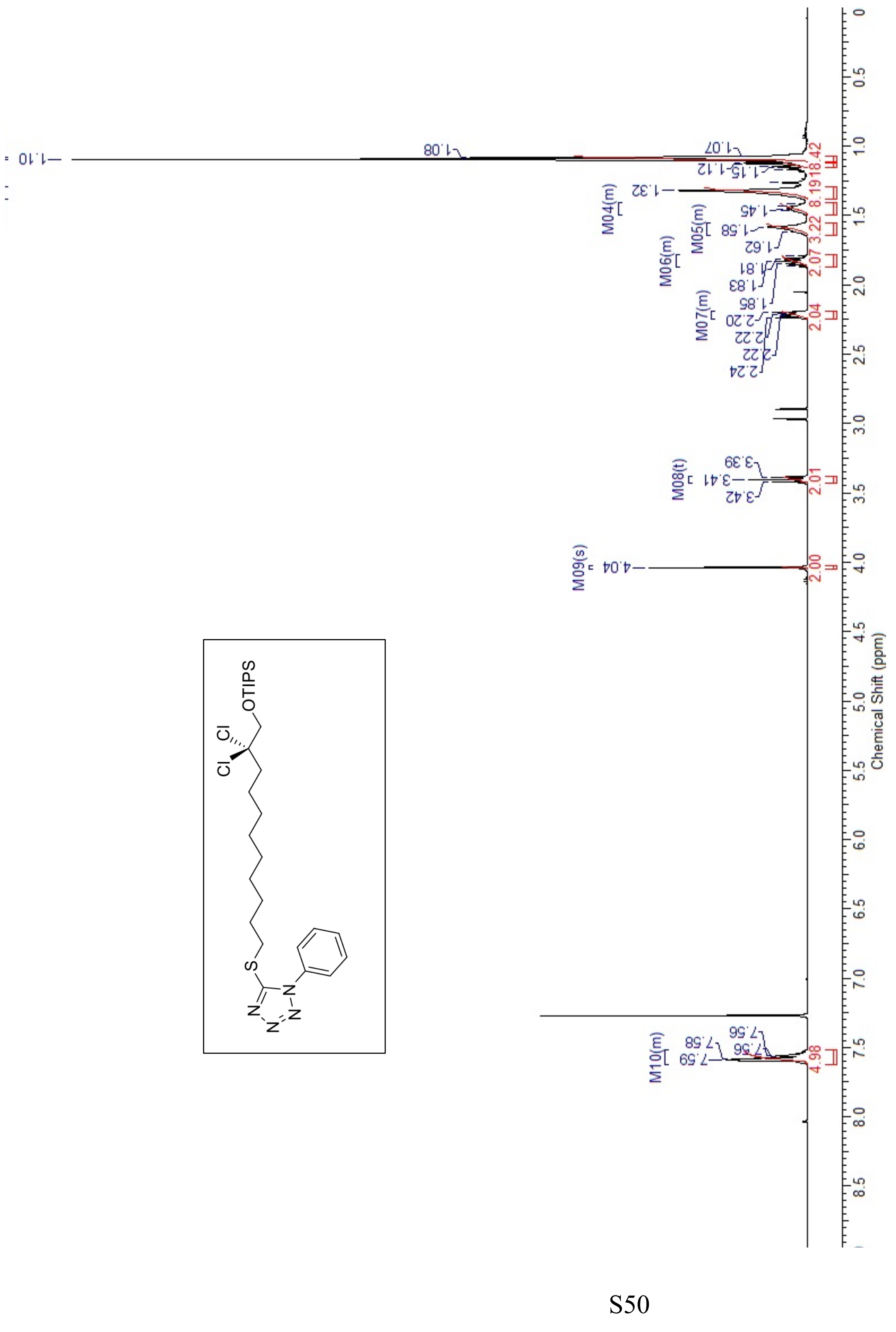
OTIPS
Cl
Cl
S
N
N
N
N
Chemical Shift (ppm)
M04(m)
M05(m)
M06(m)
M07(m)
M08(t)
M09(s)
M10(m)

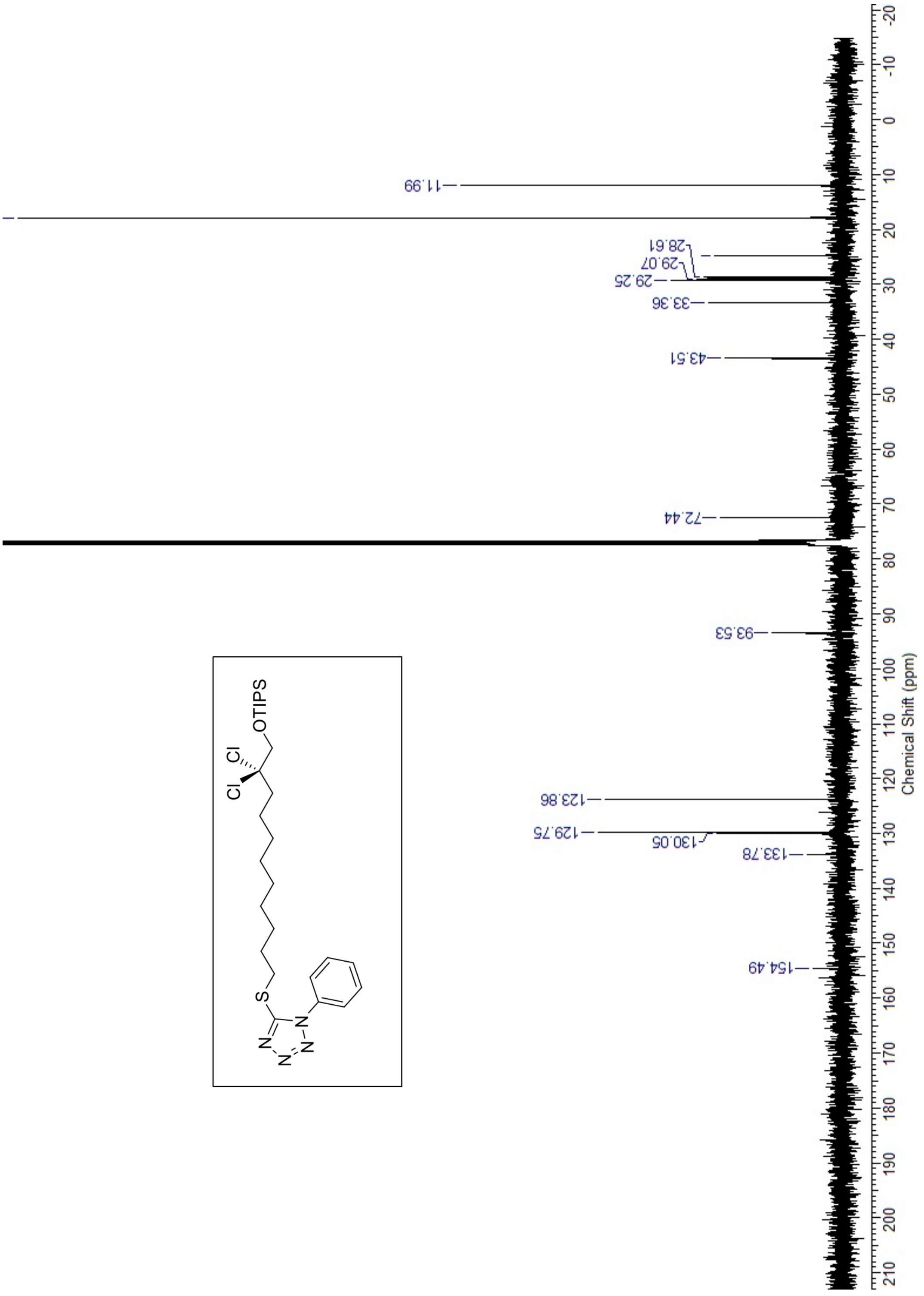
11.99
28.61
29.07
29.25
33.36
43.51
72.44
93.53
123.86
129.75
130.05
133.78
154.49
Chemical Shift (ppm)
Cl
Cl
OTIPS
S
N
N
N
N

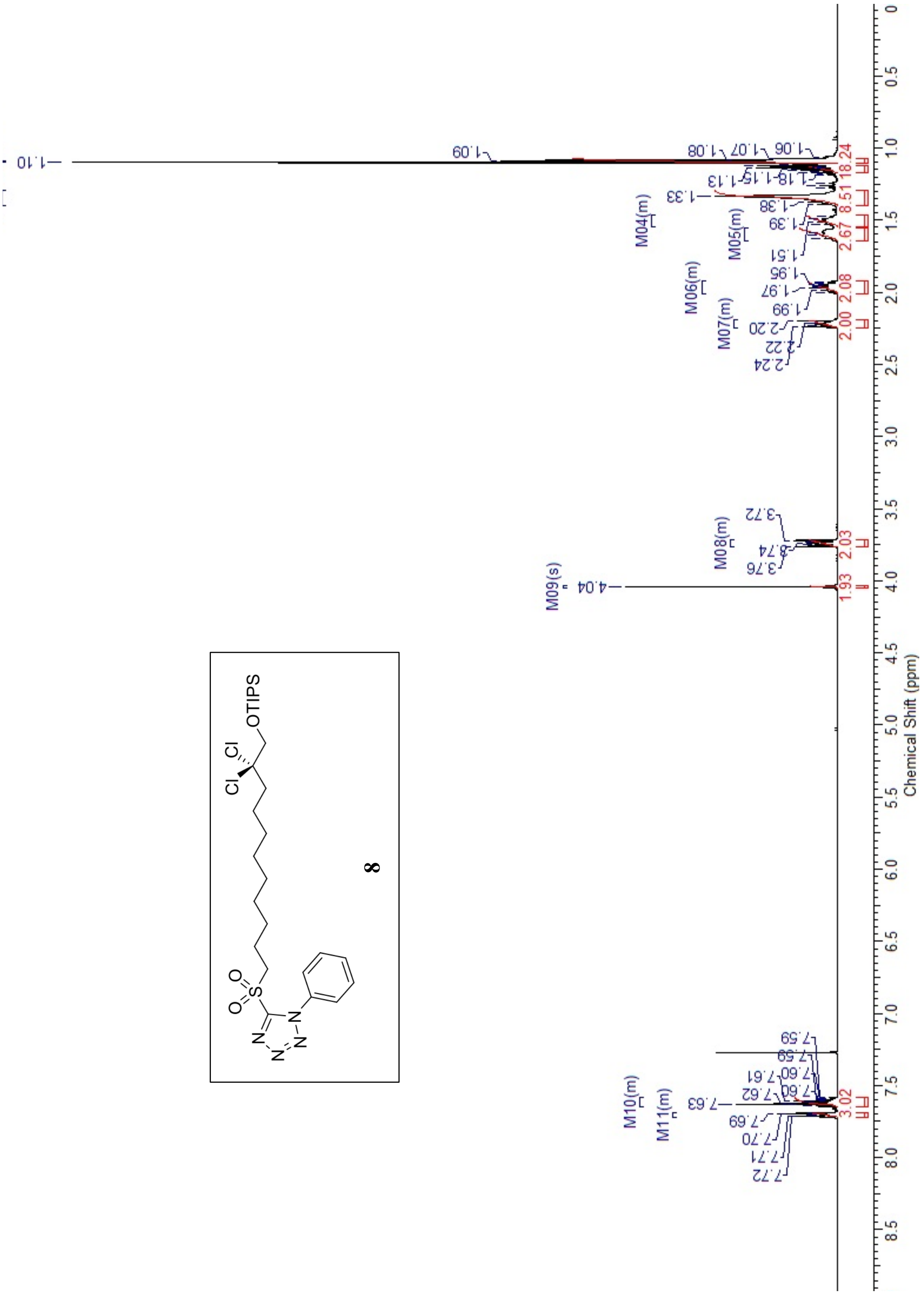
OTIPS
Cl
Cl
8
Chemical Shift (ppm)
M04(m)
M05(m)
M06(m)
M07(m)
M08(m)
M09(s)
M10(m)
M11(m)

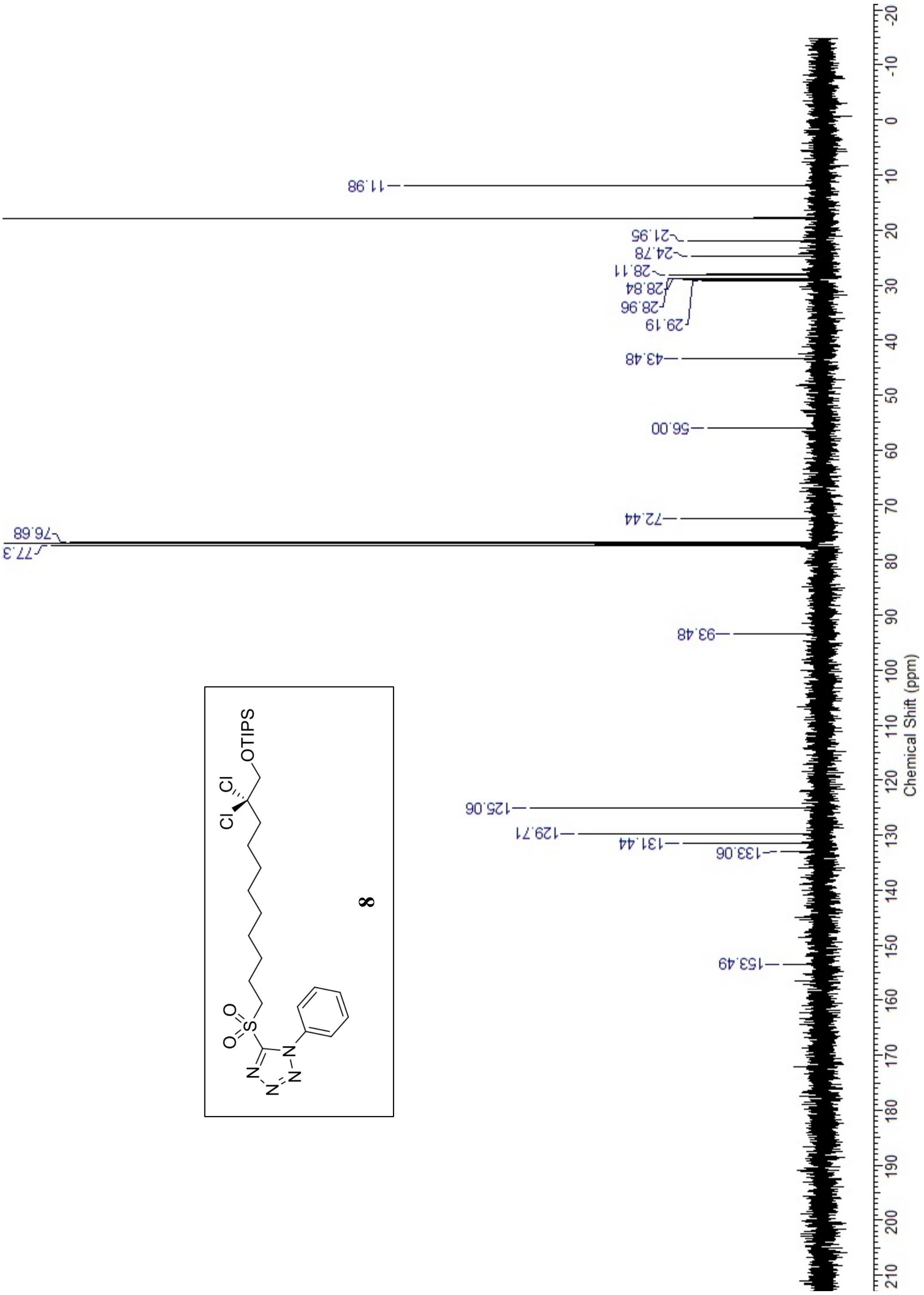
11.98
21.95
24.78
28.11
28.84
28.96
29.19
43.48
56.00
72.44
76.68
77.3
93.48
125.06
129.71
131.44
133.06
153.49
Chemical Shift (ppm)
OTIPS
Cl
Cl
S
O
O
N
N
N
N
8

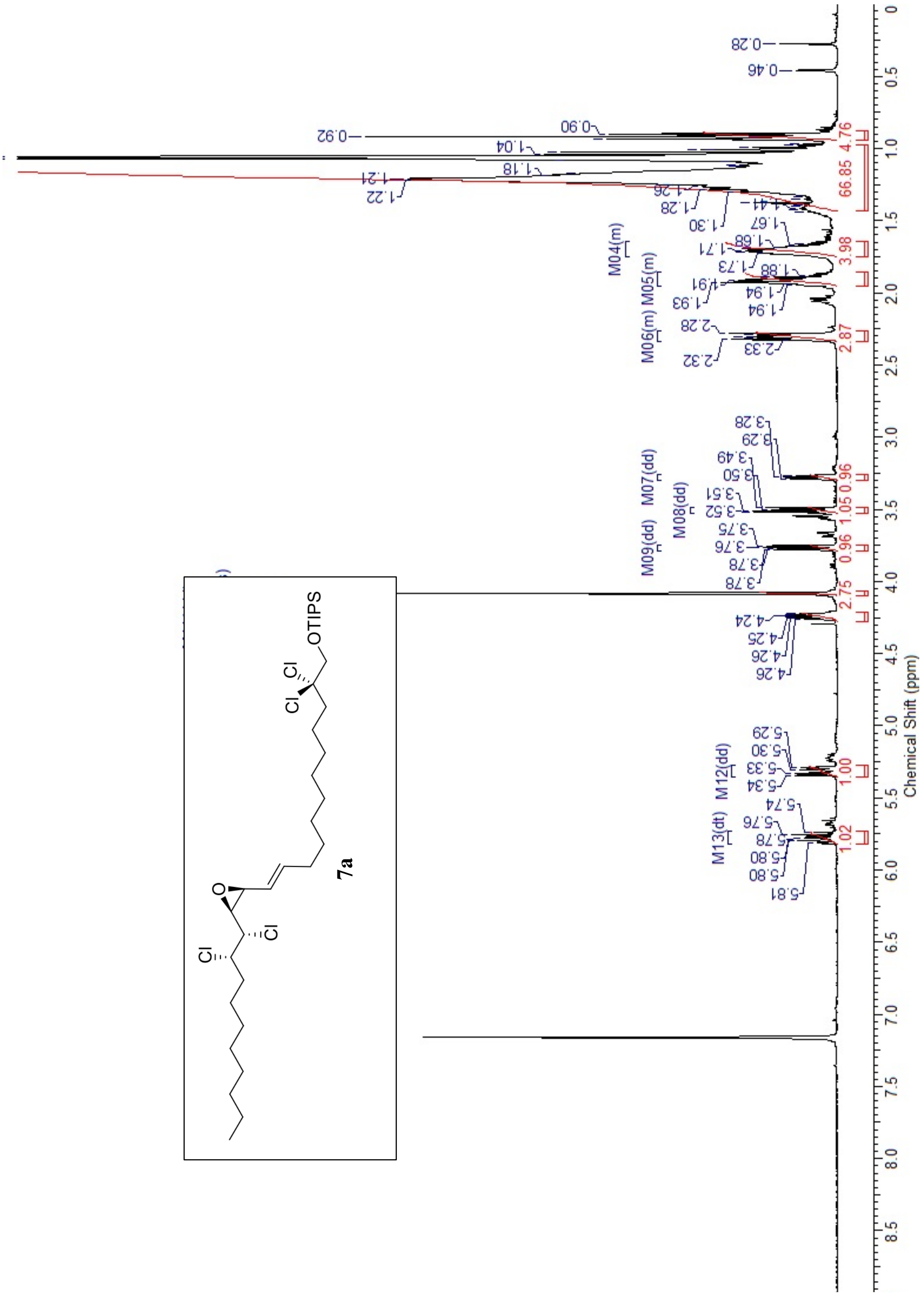
OTIPS
Cl
O
7a
Chemical Shift (ppm)

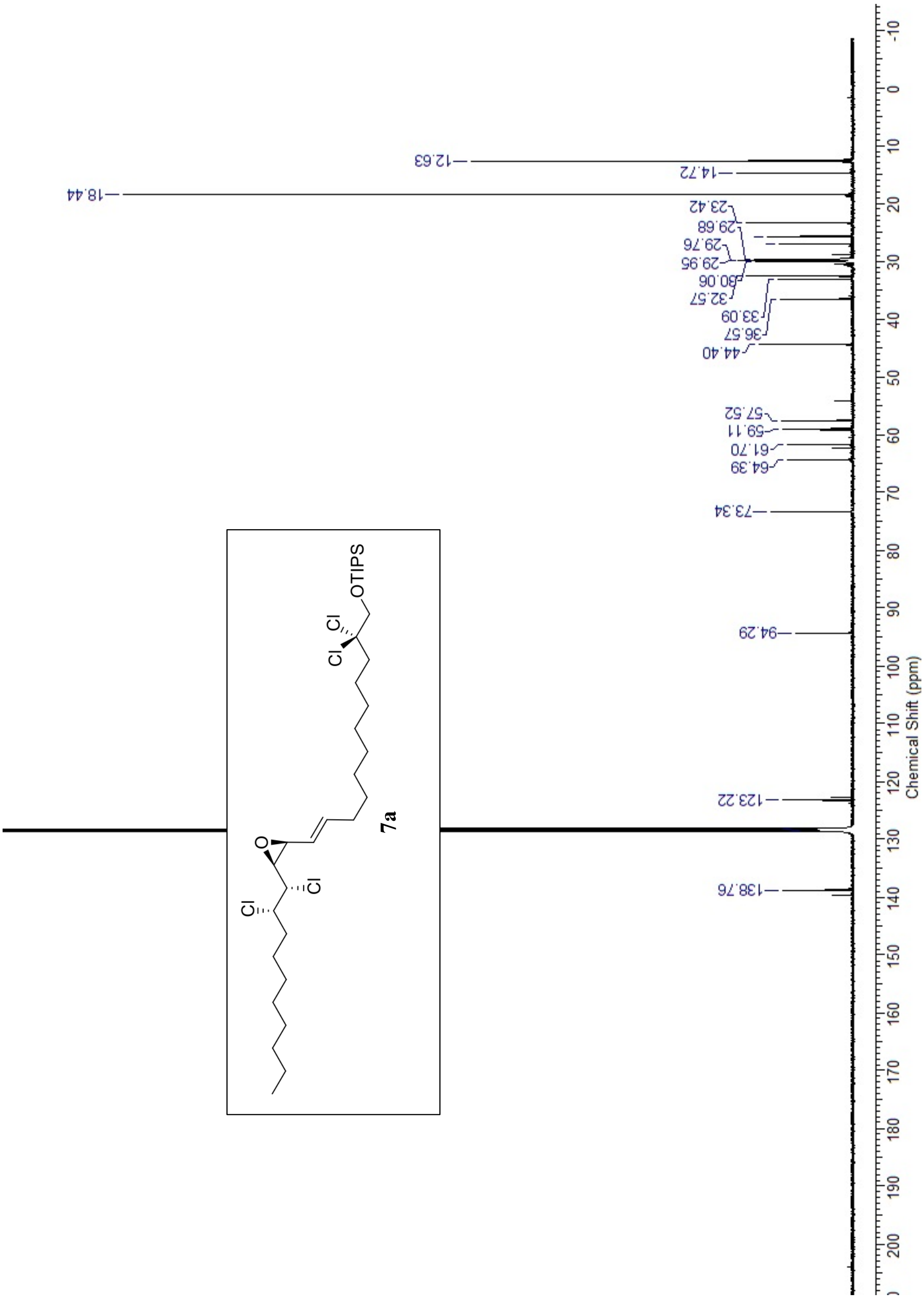

Cl
Cl
O
Cl
Cl
OTIPS
7a
138.76
123.22
94.29
73.34
64.39
61.70
59.11
57.52
44.40
36.57
33.09
32.57
30.06
29.95
29.76
29.68
23.42
18.44
14.72
12.63
200
190
180
170
160
150
140
130
120
110
100
90
80
70
60
50
40
30
20
10
0
-10
Chemical Shift (ppm)

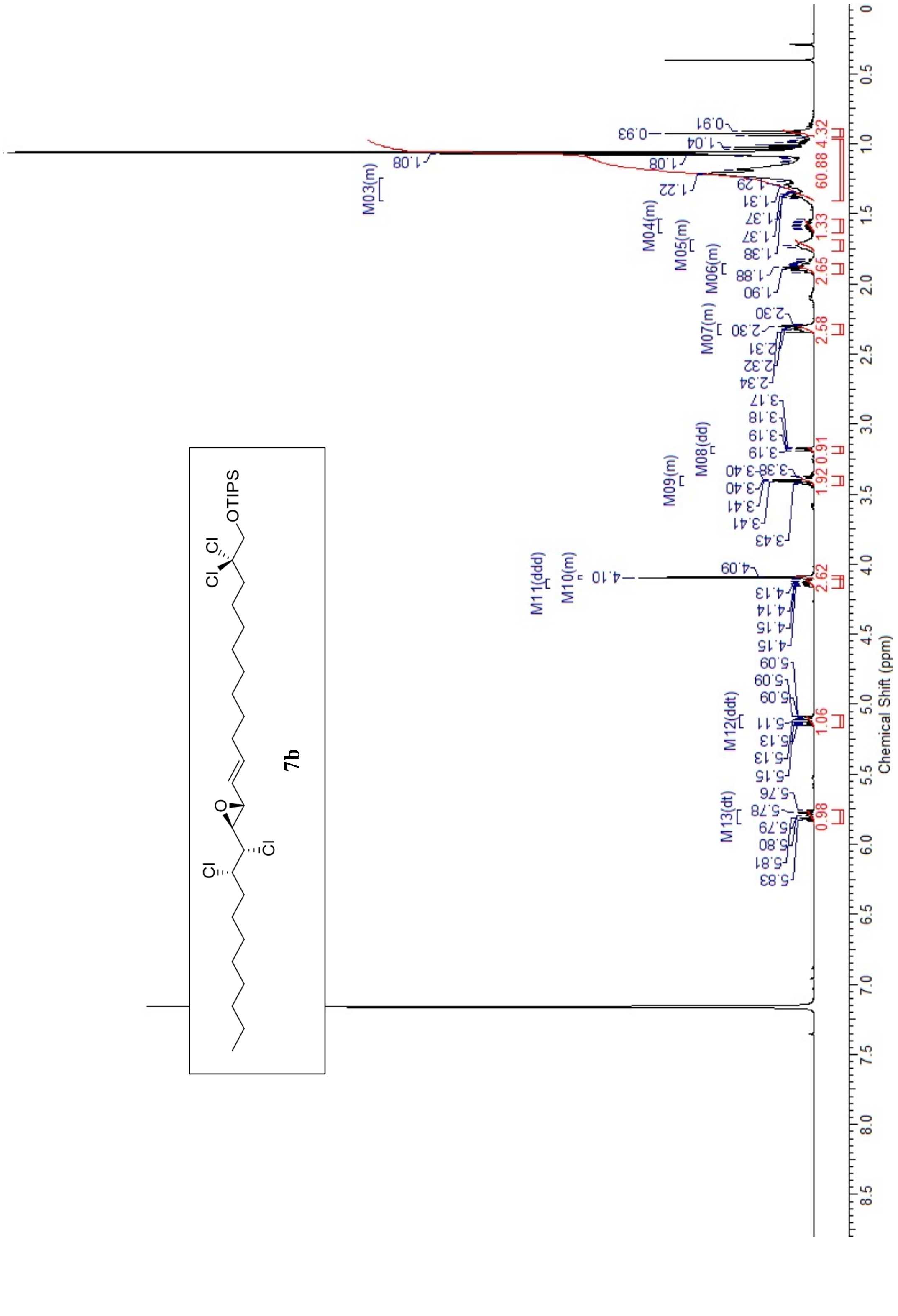
OTIPS
7b
Chemical Shift (ppm)

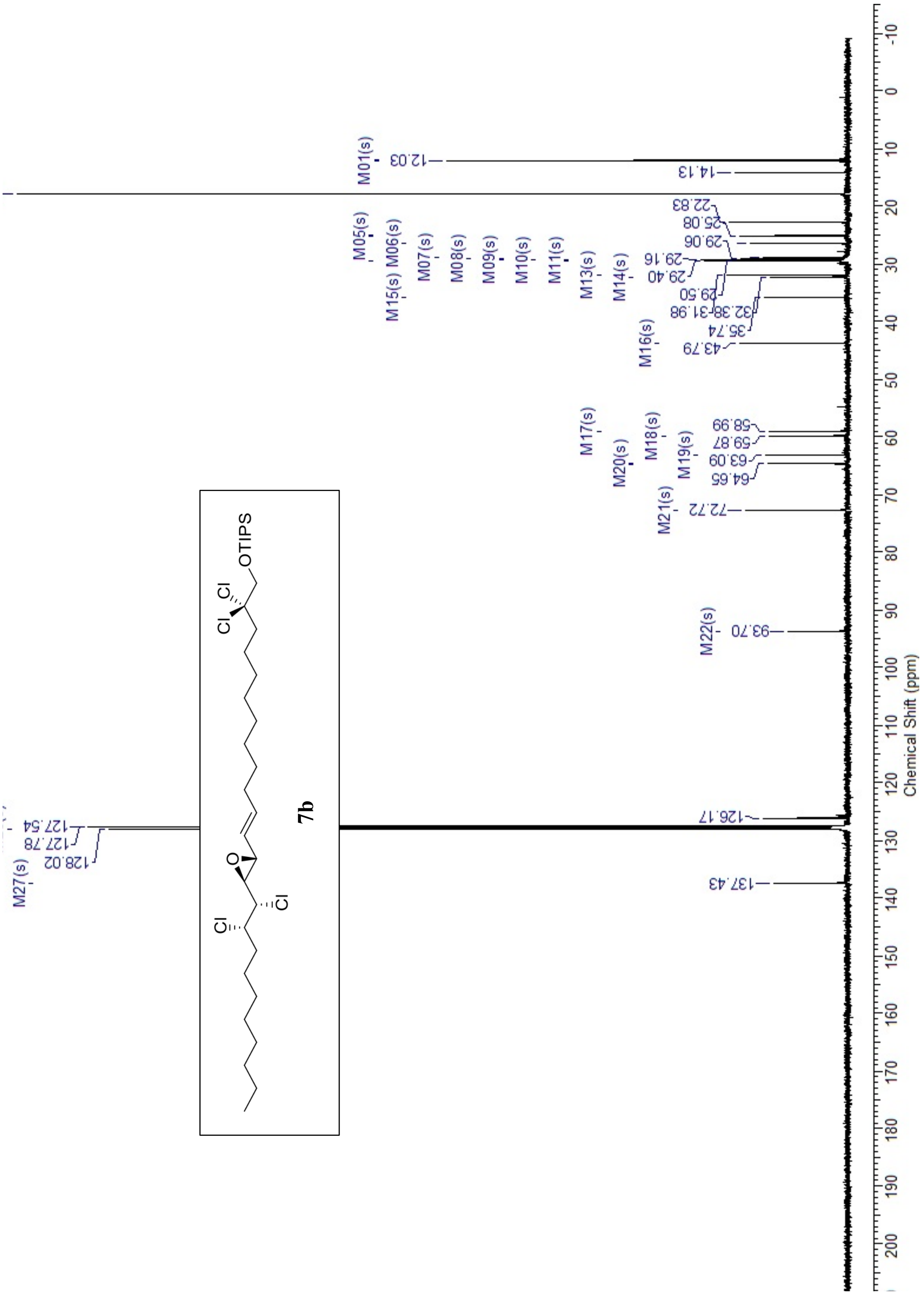

7b
Cl
Cl
Cl
Cl
O
OTIPS
12.03
14.13
22.83
25.08
29.06
29.16
29.40
29.50
31.98
32.38
35.74
43.79
58.99
59.87
63.09
64.65
72.72
93.70
126.17
127.54
127.78
128.02
137.43
M01(s)
M05(s)
M06(s)
M07(s)
M08(s)
M09(s)
M10(s)
M11(s)
M13(s)
M14(s)
M15(s)
M16(s)
M17(s)
M18(s)
M19(s)
M20(s)
M21(s)
M22(s)
M27(s)
Chemical Shift (ppm)

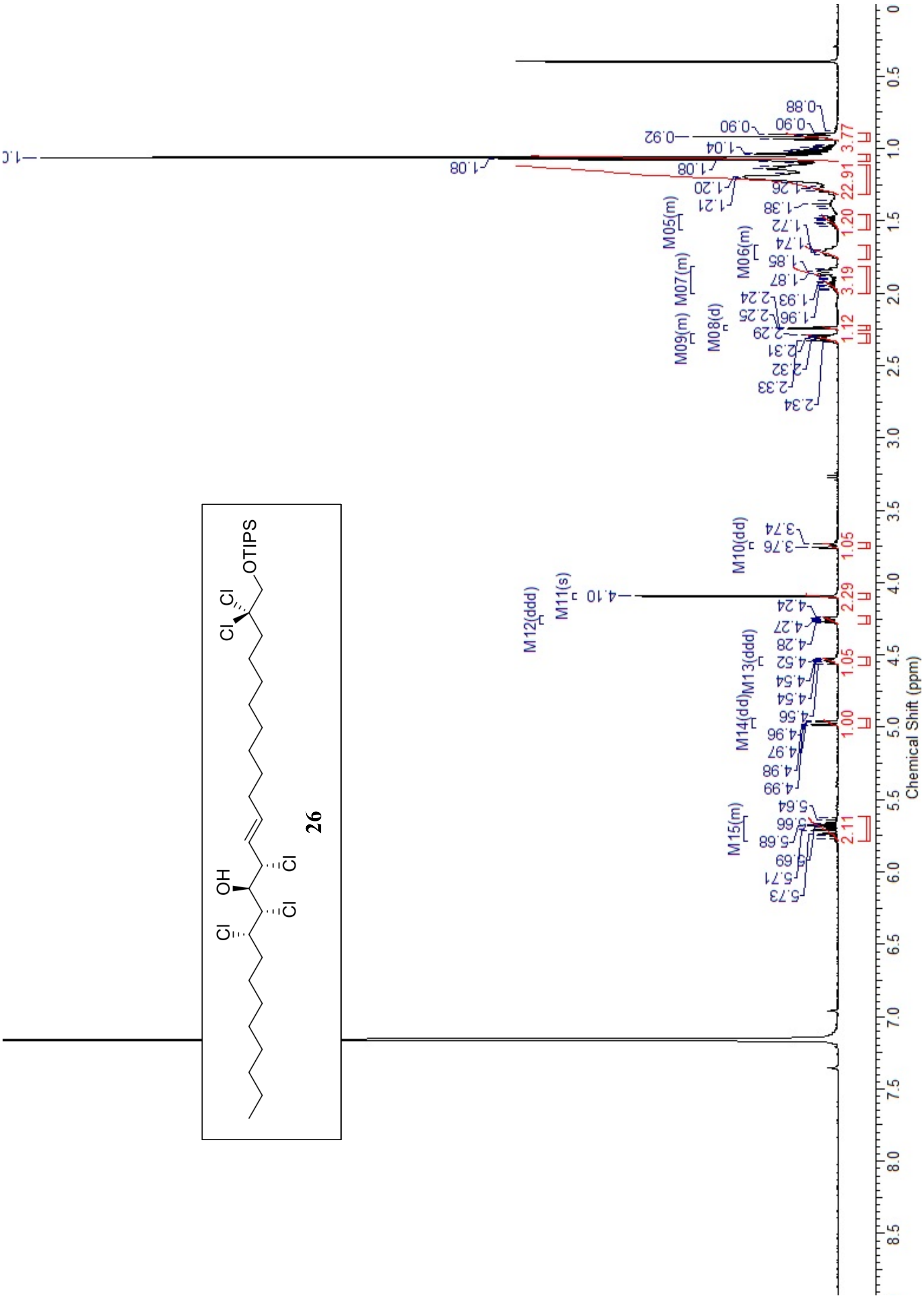
OTIPS
OH
26
Chemical Shift (ppm)
M05(m)
M06(m)
M07(m)
M08(d)
M09(m)
M10(dd)
M11(s)
M12(ddd)
M13(ddd)
M14(dd)
M15(m)
3.77
22.91
1.20
3.19
1.12
1.05
2.29
1.05
1.00
2.11

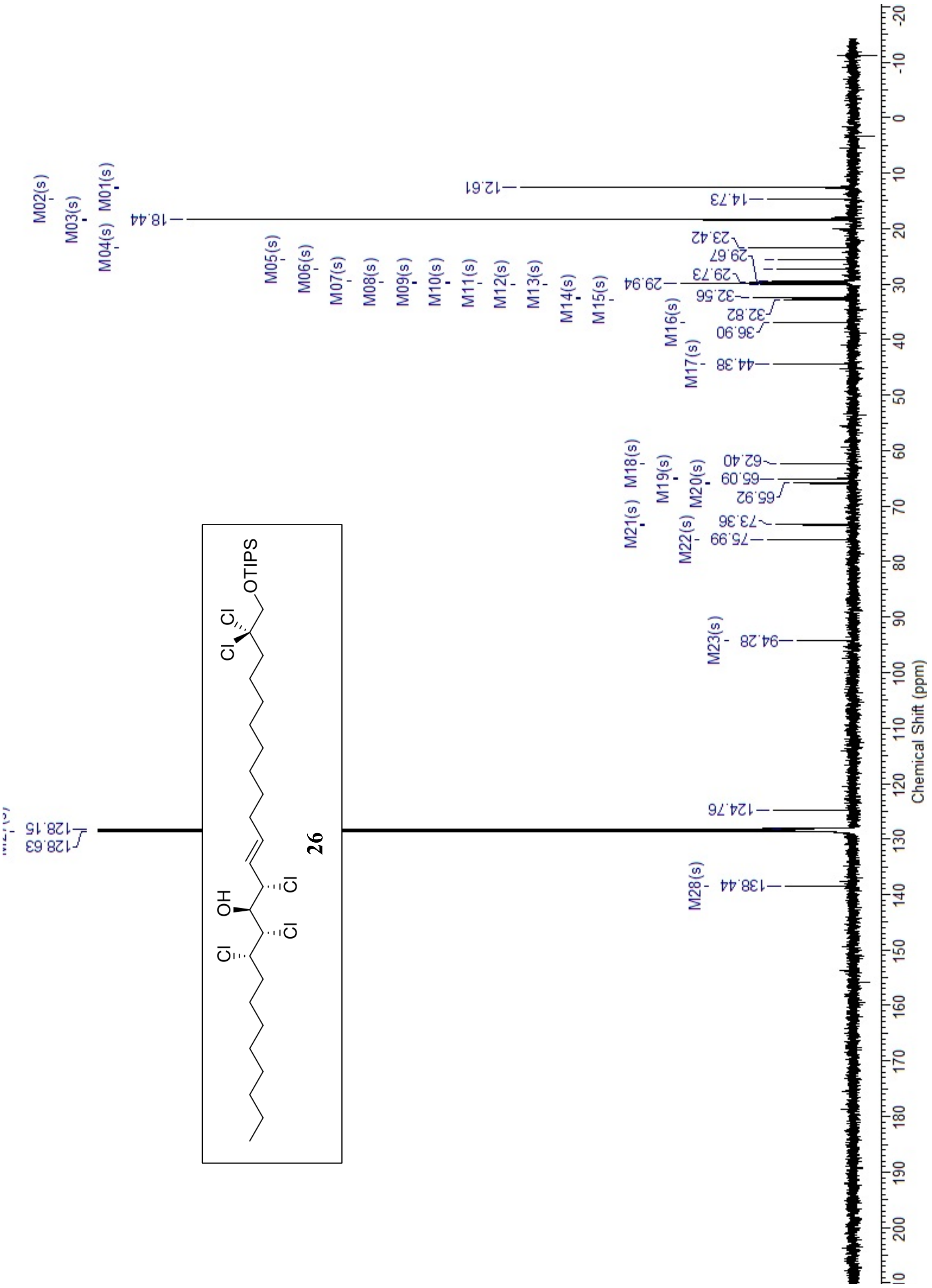
12.61
14.73
18.44
23.42
29.67
29.73
29.94
32.56
32.82
36.90
44.38
62.40
65.09
65.92
73.36
75.99
94.28
124.76
128.15
128.63
138.44
Chemical Shift (ppm)
OTIPS
Cl
OH
26

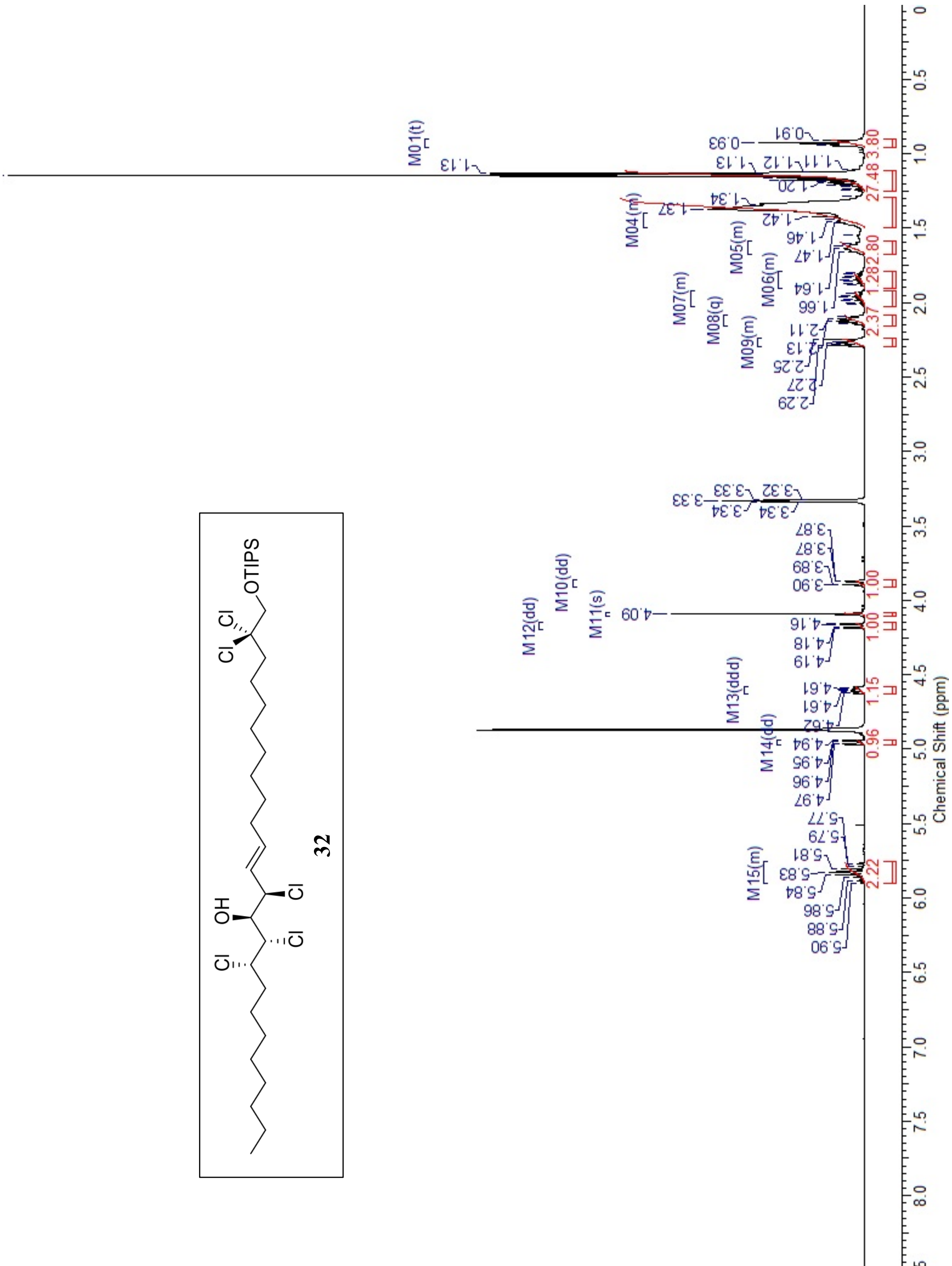

Cl
Cl
OTIPS
OH
Cl
Cl
Cl
32
M01(t)
M04(m)
M05(m)
M06(m)
M07(m)
M08(q)
M09(m)
M10(dd)
M11(s)
M12(dd)
M13(ddd)
M14(dd)
M15(m)
Chemical Shift (ppm)

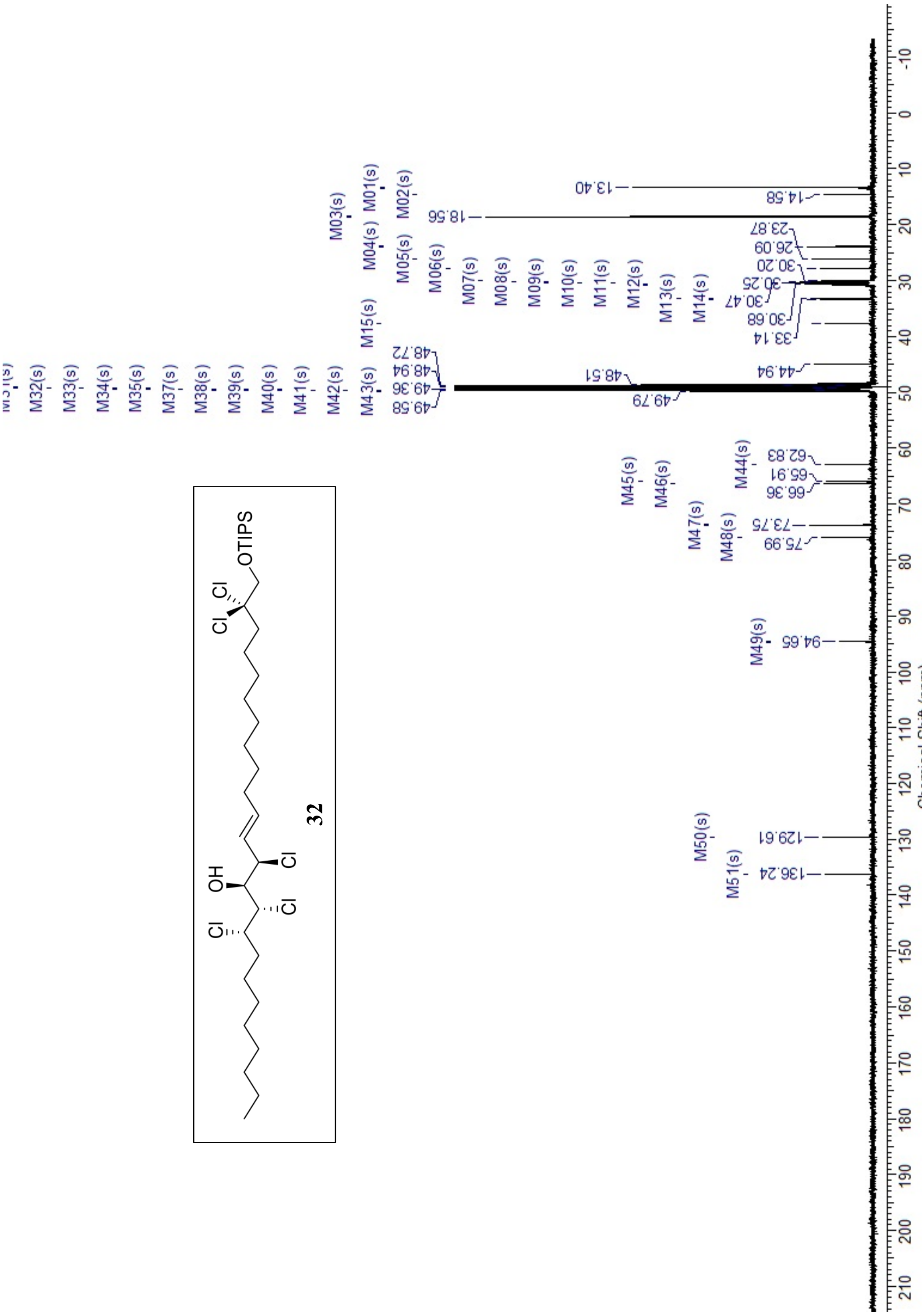

32
Cl
OH
Cl
Cl
Cl
Cl
OTIPS
13.40
14.58
18.56
23.87
26.09
30.20
30.25
30.47
30.68
33.14
44.94
48.51
48.72
48.94
49.36
49.58
49.79
62.83
65.91
66.36
73.75
75.99
94.65
129.61
136.24
Chemical Shift (ppm)

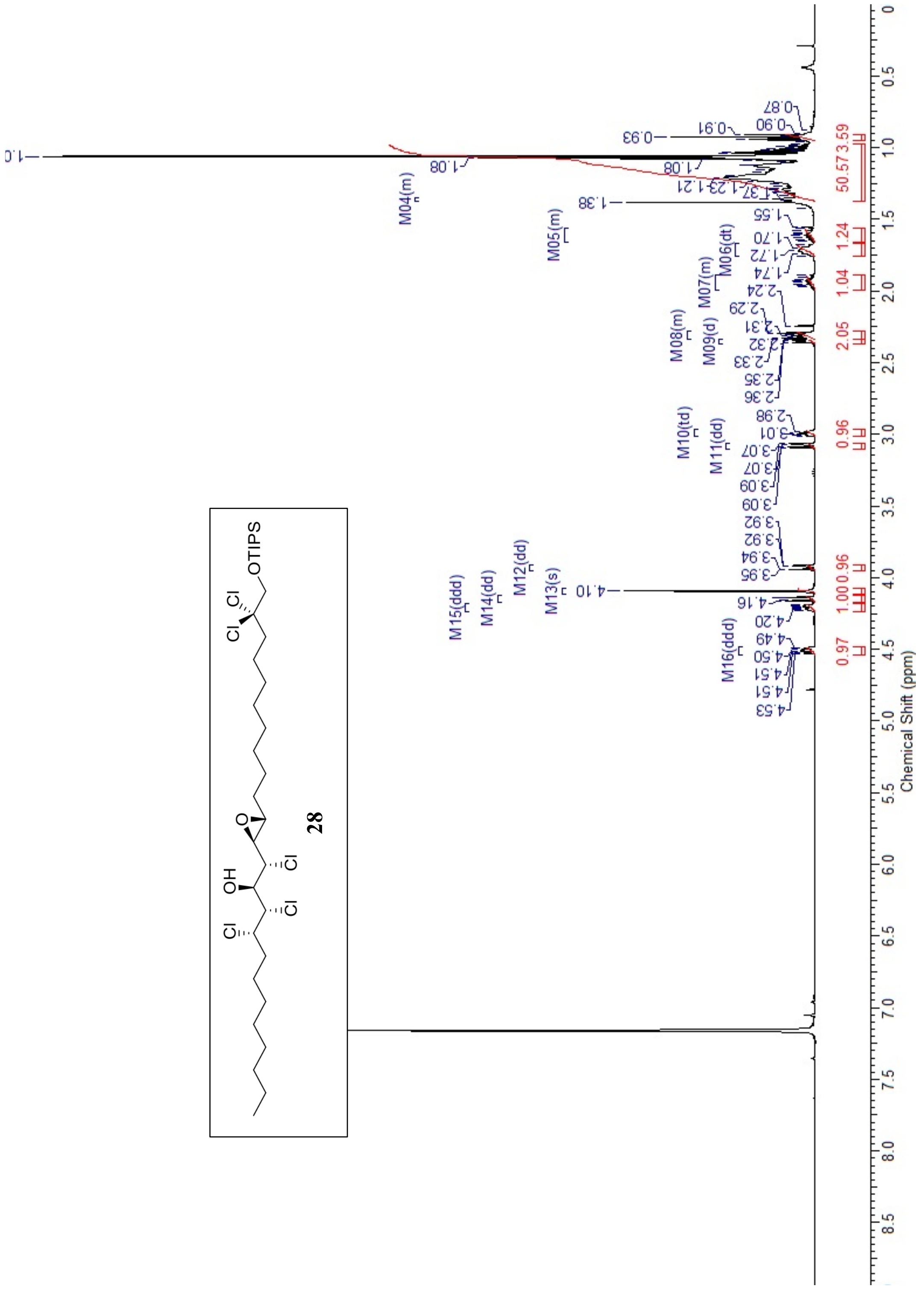
OTIPS
28
Chemical Shift (ppm)

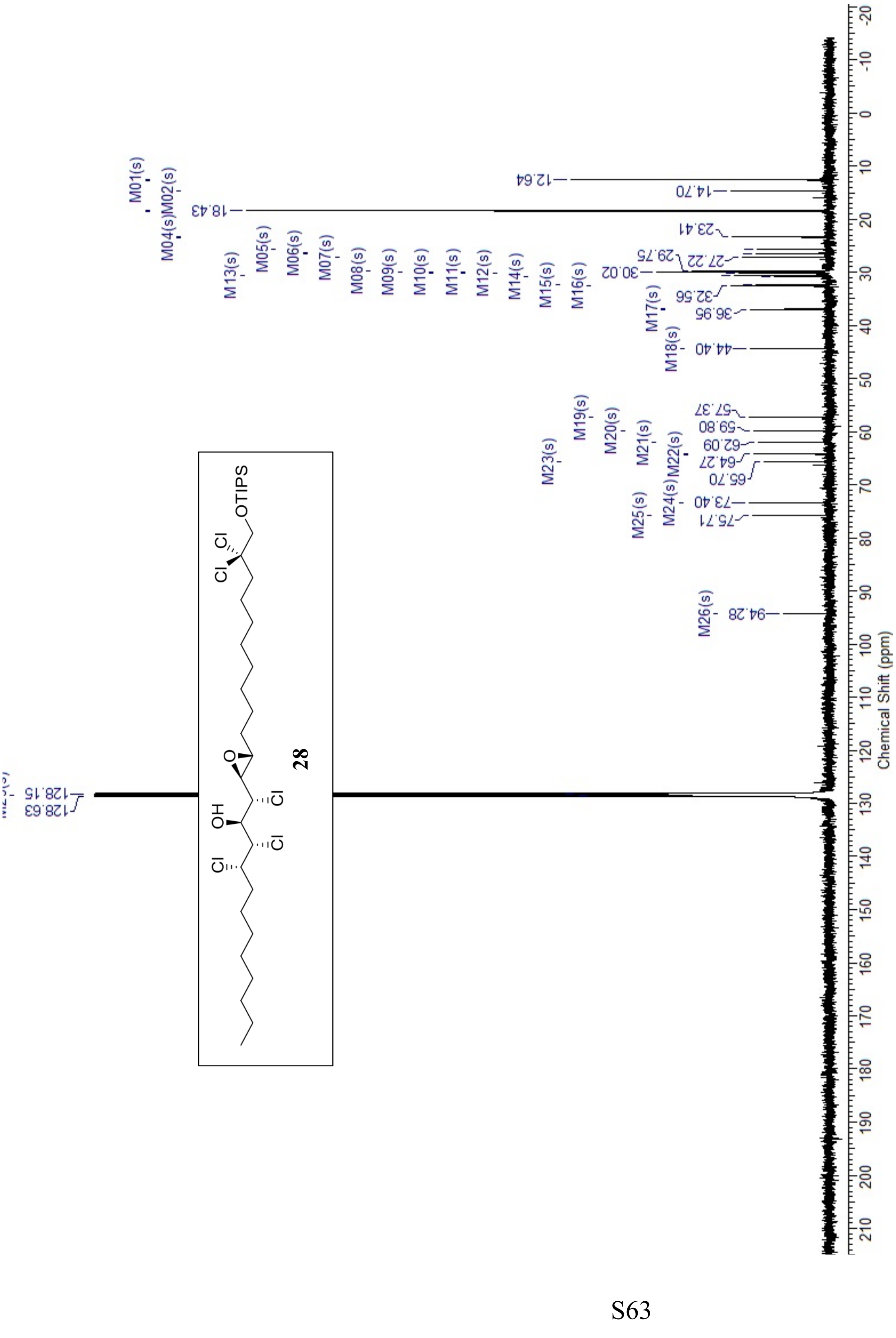
12.64
14.70
18.43
23.41
27.22
29.75
30.02
32.56
36.95
44.40
57.37
59.80
62.09
64.27
65.70
73.40
75.71
94.28
128.15
128.63
Chemical Shift (ppm)
OTIPS
OH
28

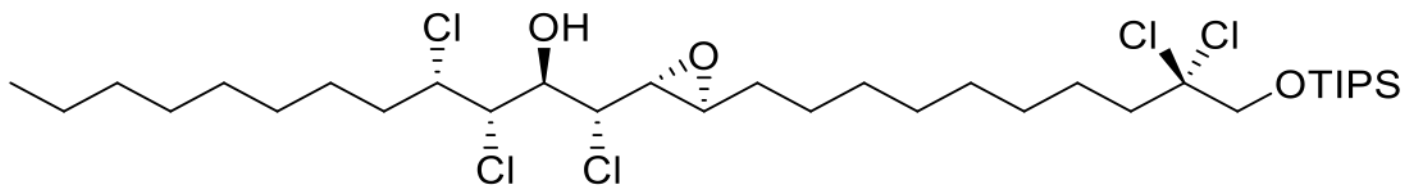
Cl
OH
Cl
Cl
Cl
Cl
OTIPS
O

29

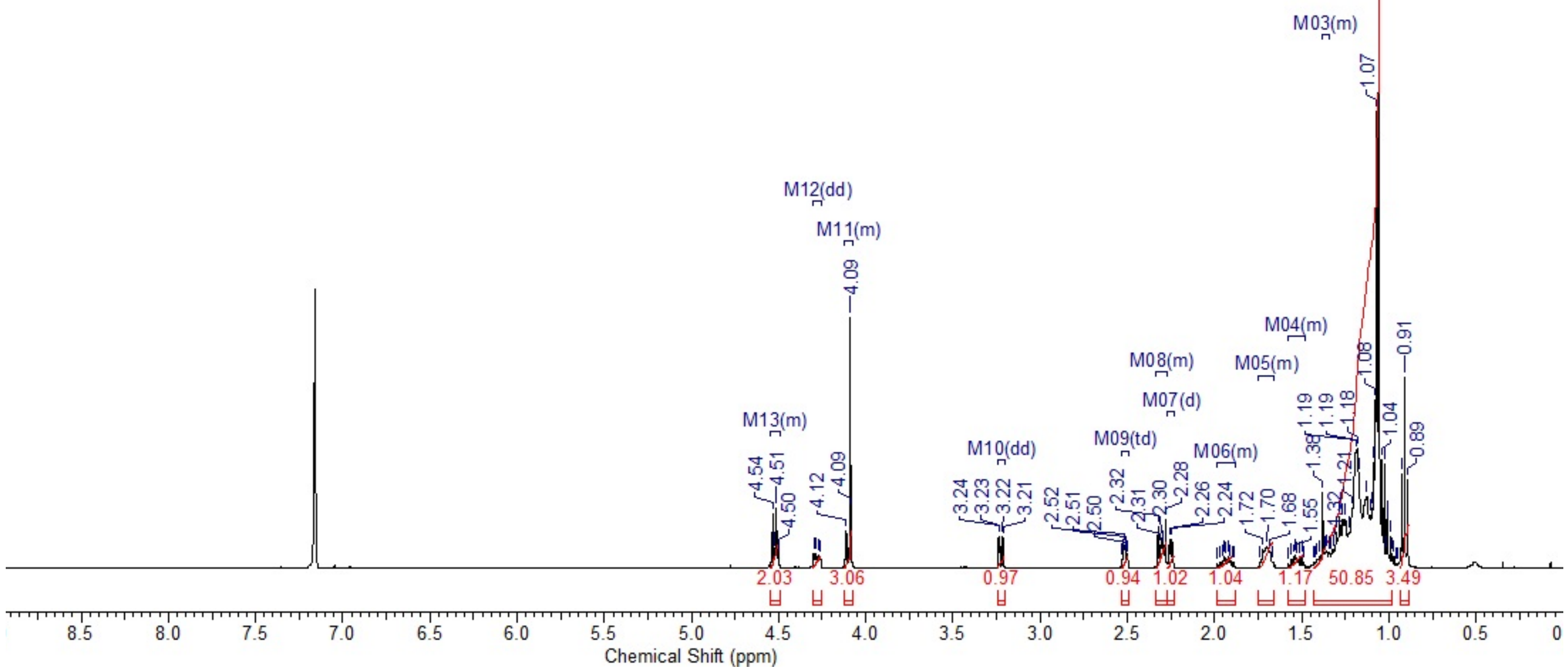
M03(m)
M04(m)
M05(m)
M06(m)
M07(d)
M08(m)
M09(td)
M10(dd)
M11(m)
M12(dd)
M13(m)
4.54
4.51
4.50
4.12
4.09
4.09
3.24
3.23
3.22
3.21
2.52
2.51
2.50
2.32
2.31
2.30
2.28
2.26
2.24
1.72
1.70
1.68
1.55
1.38
1.32
1.21
1.19
1.19
1.18
1.08
1.07
1.04
0.91
0.89
2.03
3.06
0.97
0.94
1.02
1.04
1.17
50.85
3.49
8.5
8.0
7.5
7.0
6.5
6.0
5.5
5.0
4.5
4.0
3.5
3.0
2.5
2.0
1.5
1.0
0.5
0
Chemical Shift (ppm)

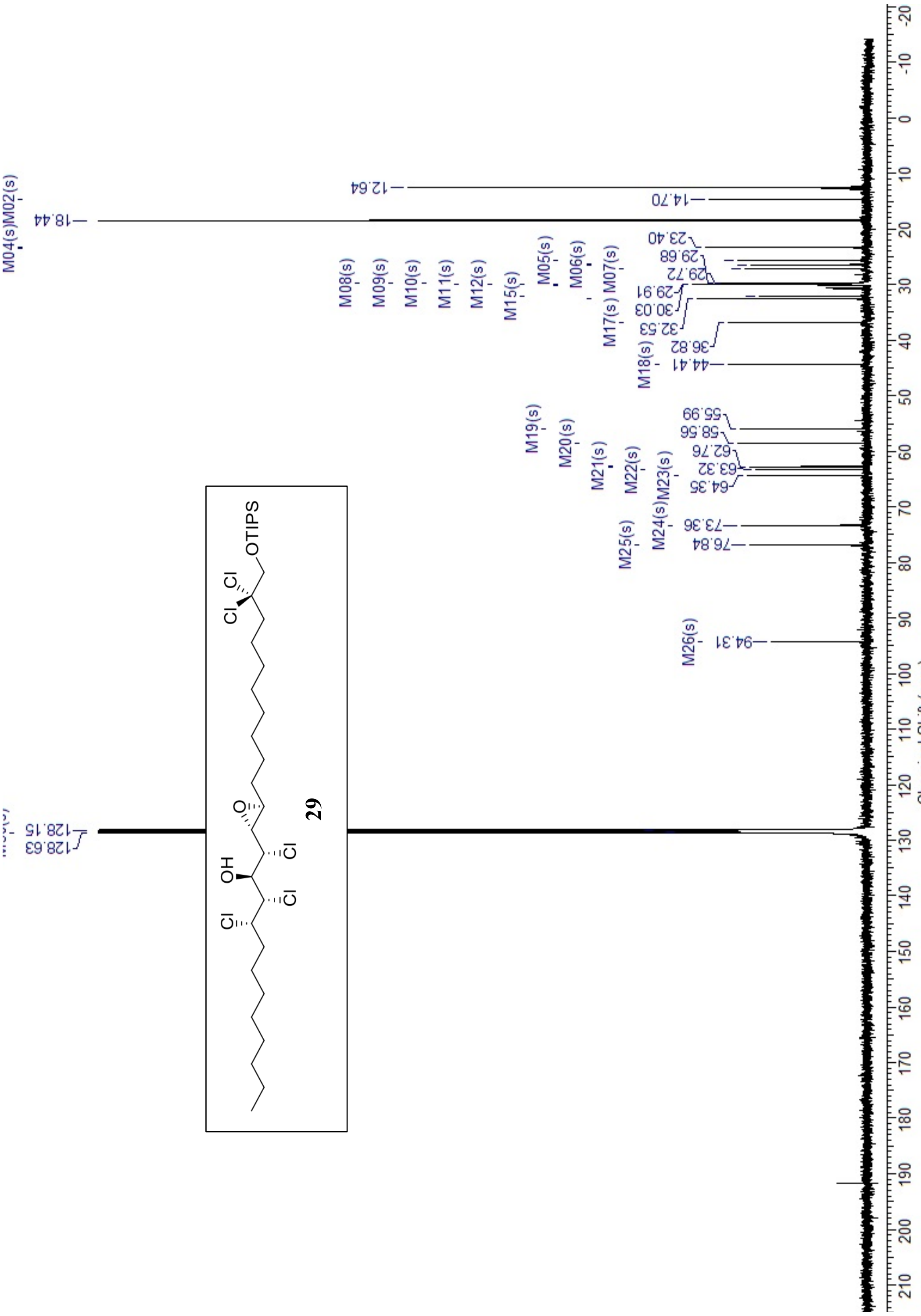

Chemical Shift (ppm)
OTIPS
29
12.64
14.70
18.44
23.40
29.68
29.72
29.91
30.03
32.53
36.82
44.41
55.99
58.56
62.76
63.32
64.35
73.36
76.84
94.31
128.15
128.63

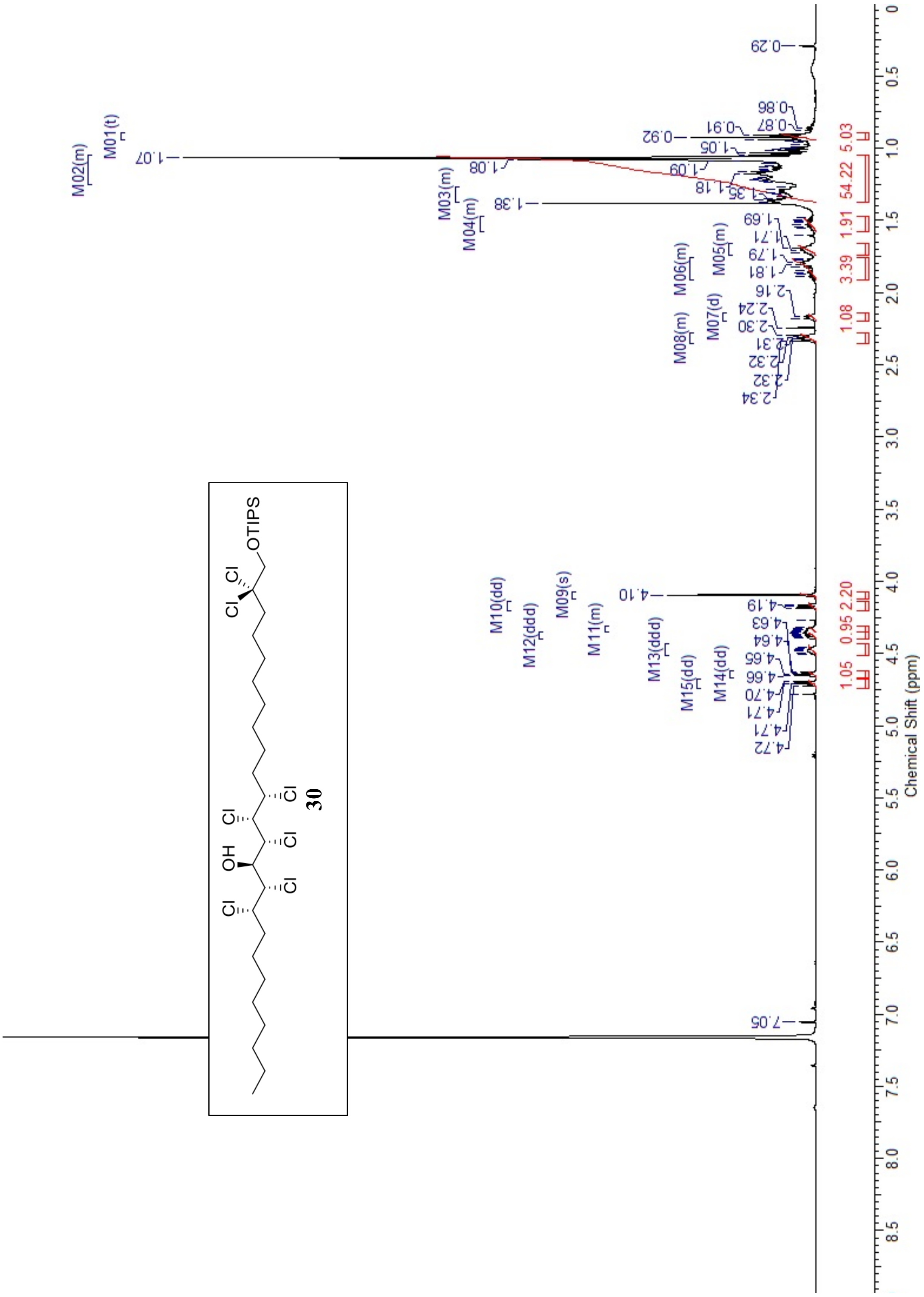
OTIPS
OH
Cl
30
Chemical Shift (ppm)

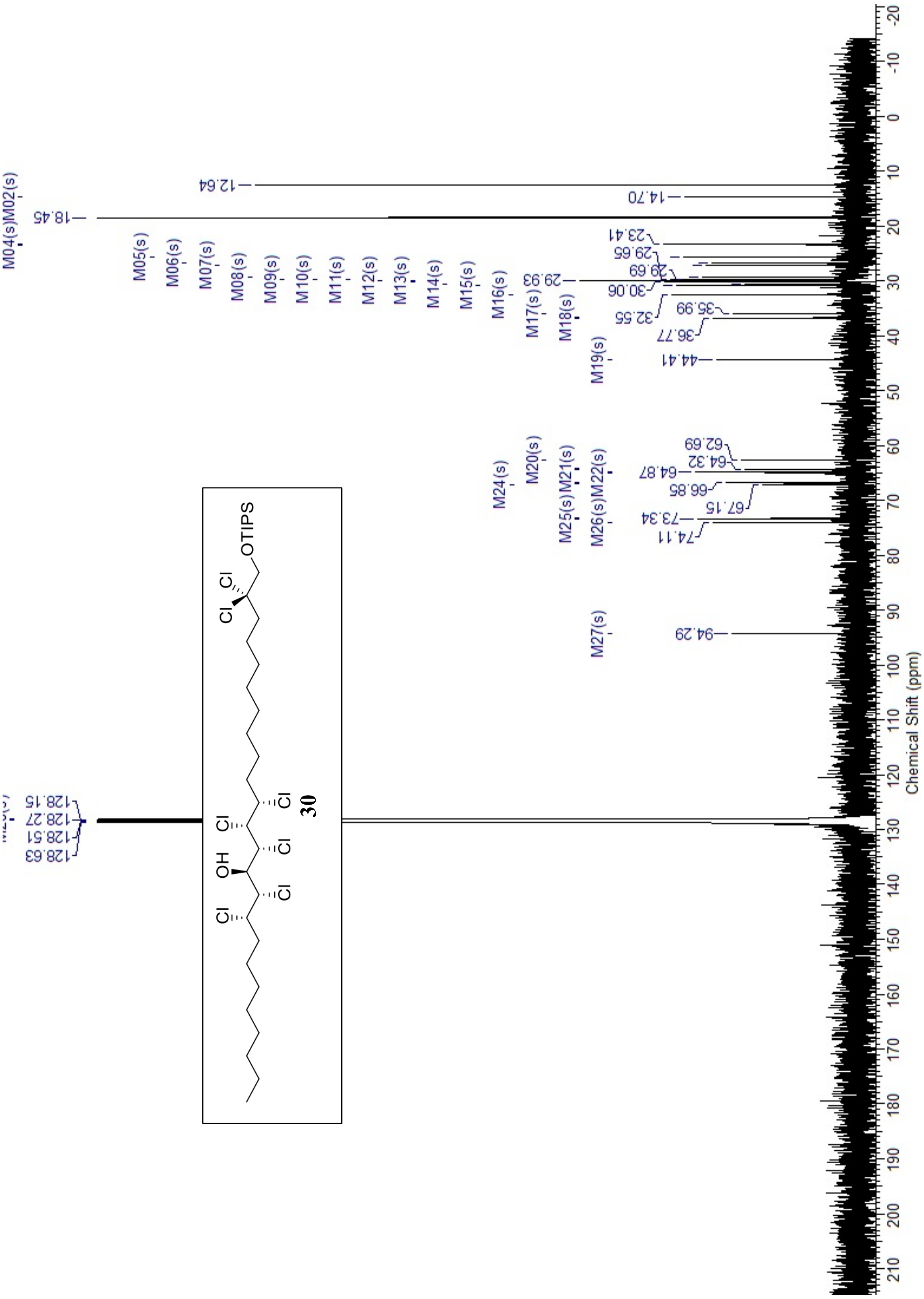

12.64
14.70
18.45
23.41
29.65
29.69
29.93
30.06
32.55
35.99
36.77
44.41
62.69
64.32
64.87
66.85
67.15
73.34
74.11
94.29
128.15
128.27
128.51
128.63
Chemical Shift (ppm)
OTIPS
OH
Cl
30

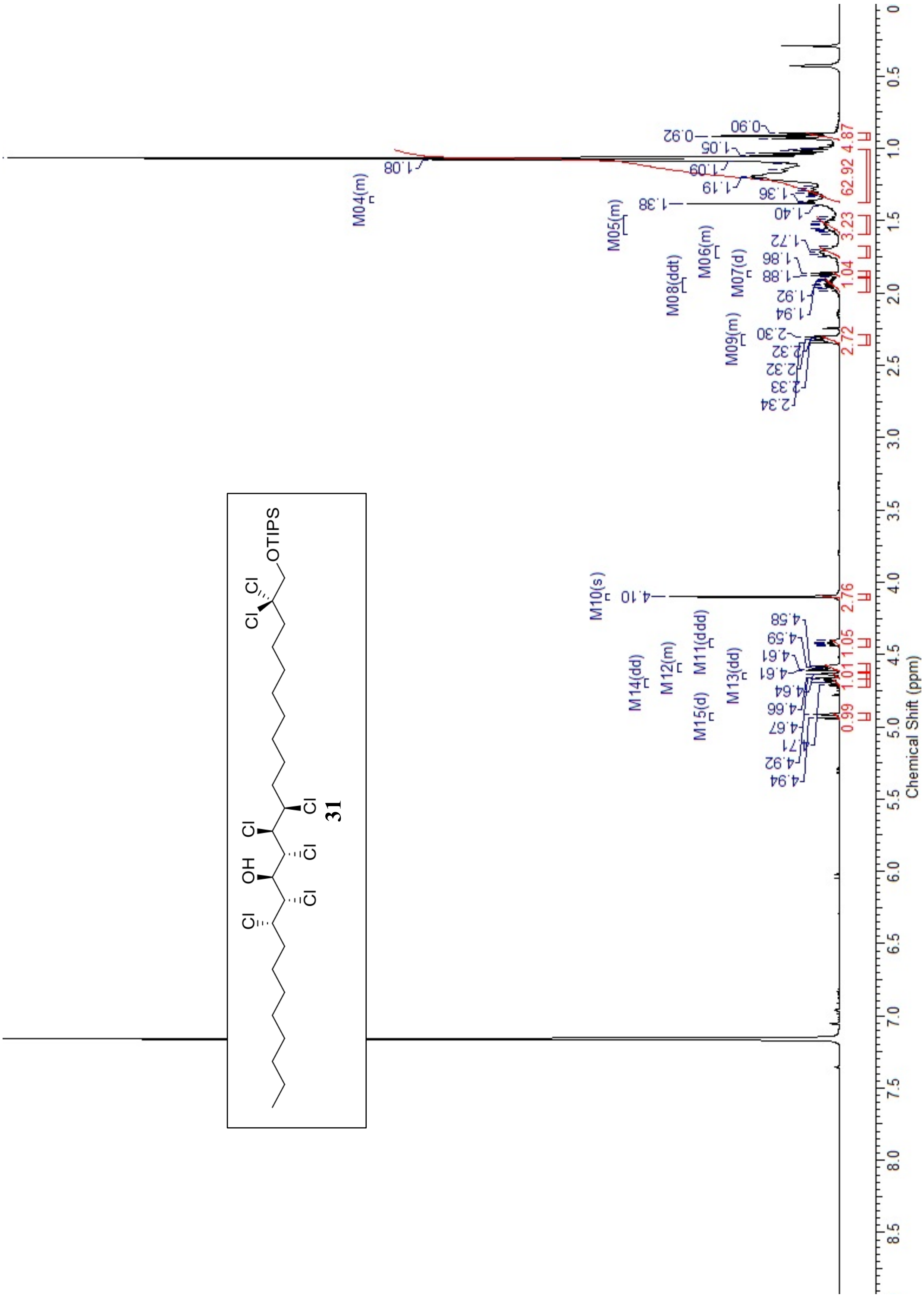
Chemical Shift (ppm)
31

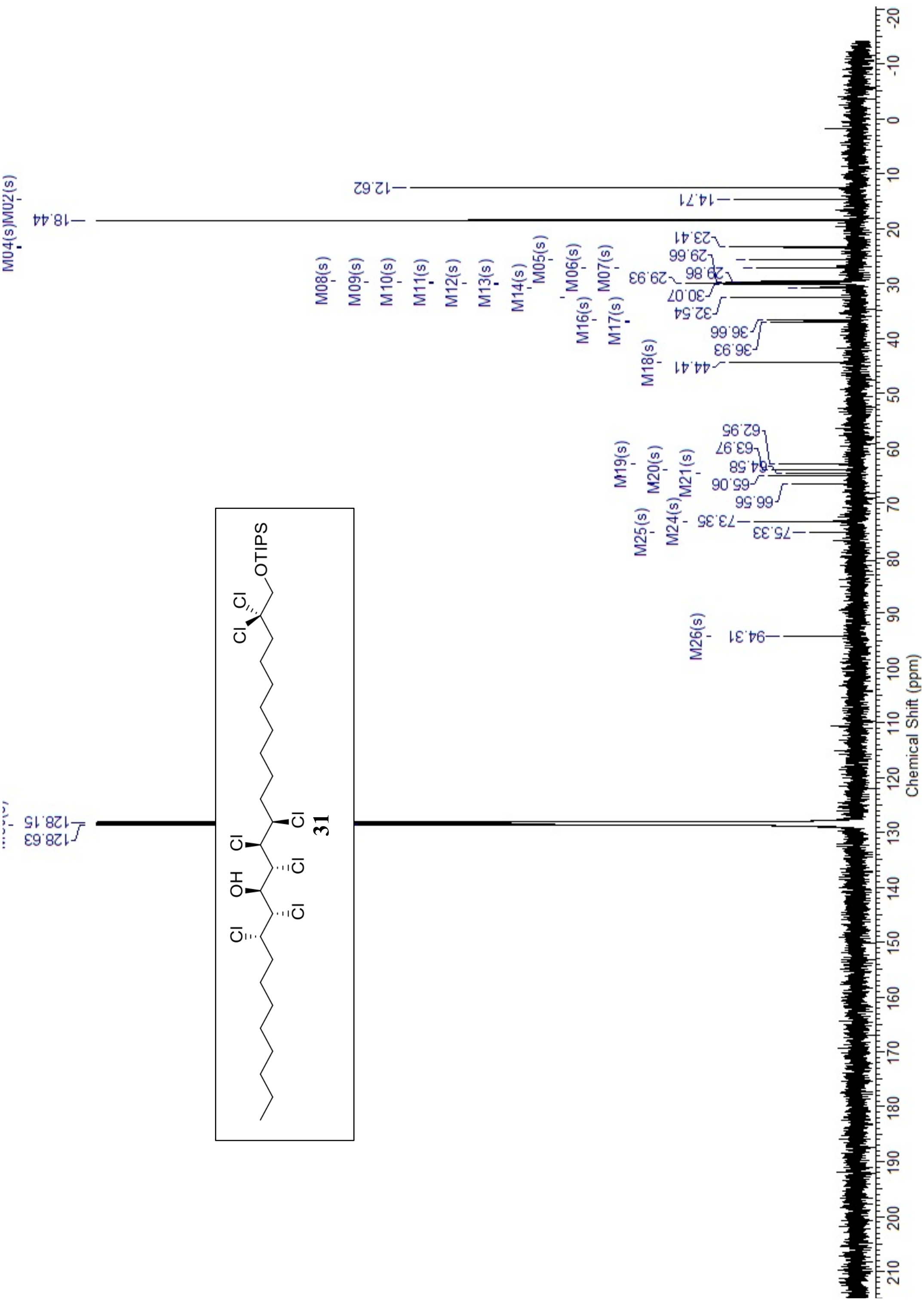

Chemical Shift (ppm)
OTIPS
31

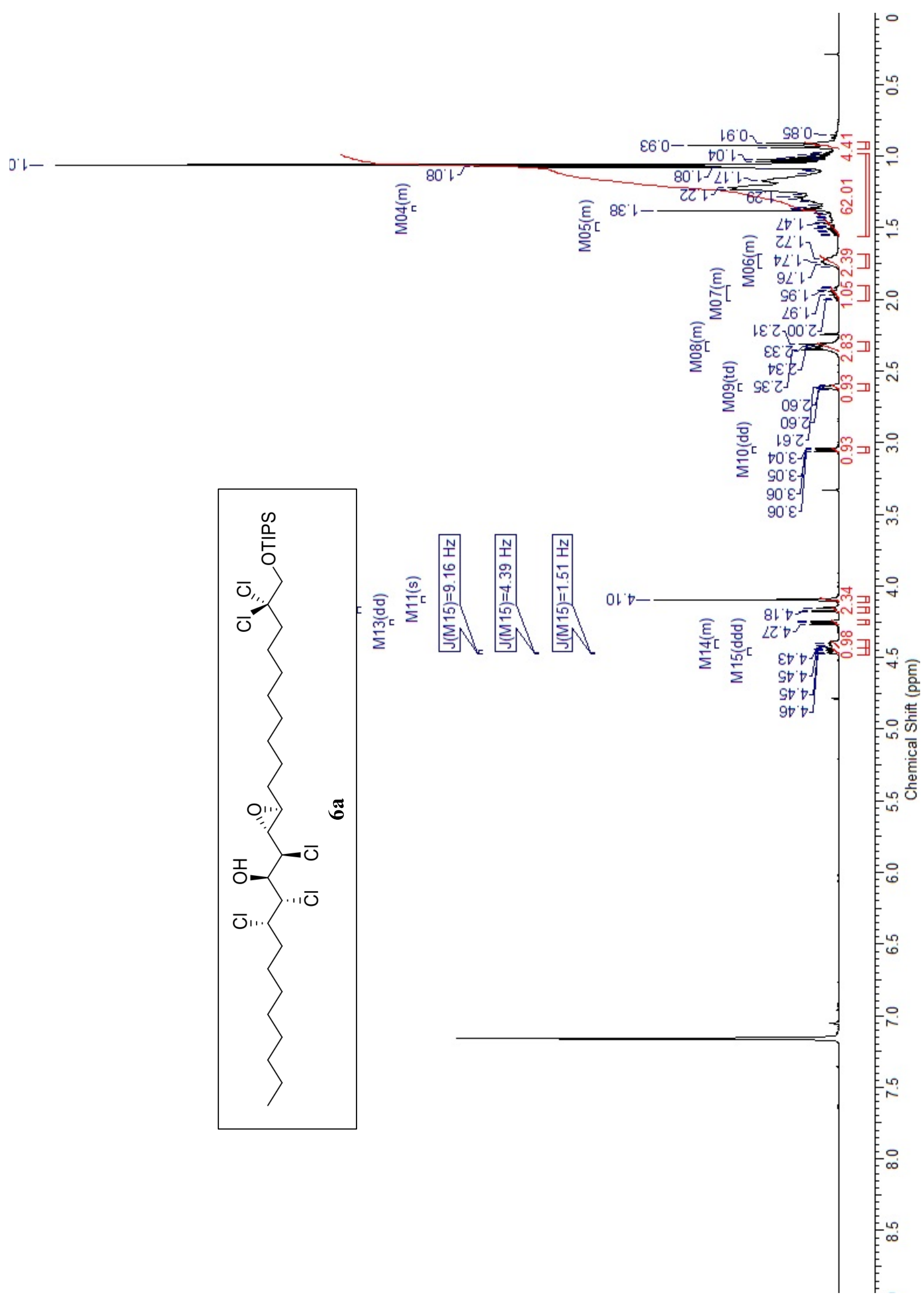
OTIPS
OH
6a
M04(m)
M05(m)
M06(m)
M07(m)
M08(m)
M09(td)
M10(dd)
M11(s)
M13(dd)
M14(m)
M15(ddd)
J(M15)=9.16 Hz
J(M15)=4.39 Hz
J(M15)=1.51 Hz
4.41
62.01
2.39
1.05
2.83
0.93
0.93
2.34
0.98
Chemical Shift (ppm)

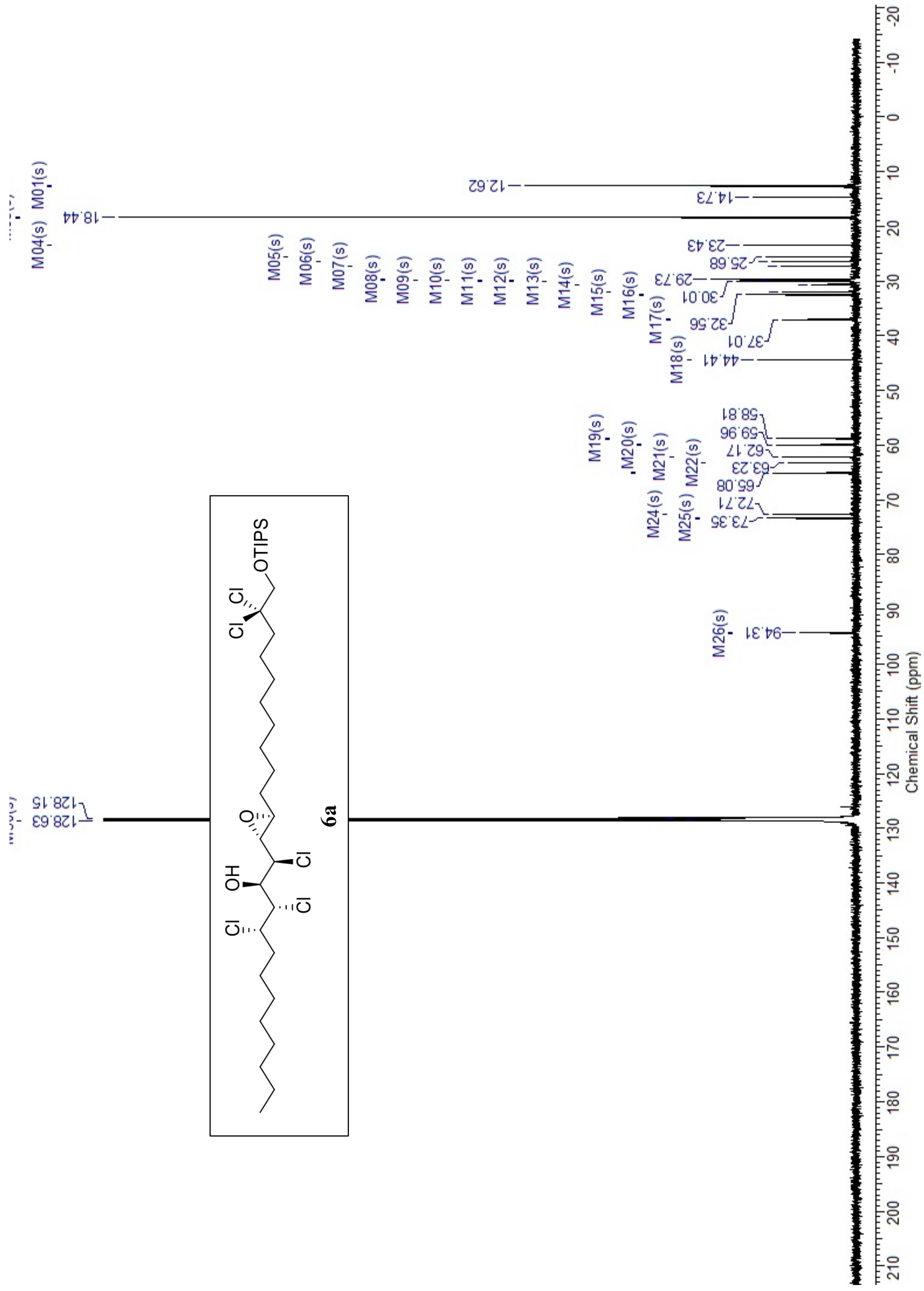

12.62
14.73
18.44
23.43
25.68
29.73
30.01
32.56
37.01
44.41
58.81
59.96
62.17
63.23
65.08
72.71
73.35
94.31
128.15
128.63
Chemical Shift (ppm)
OTIPS
OH
Cl
6a

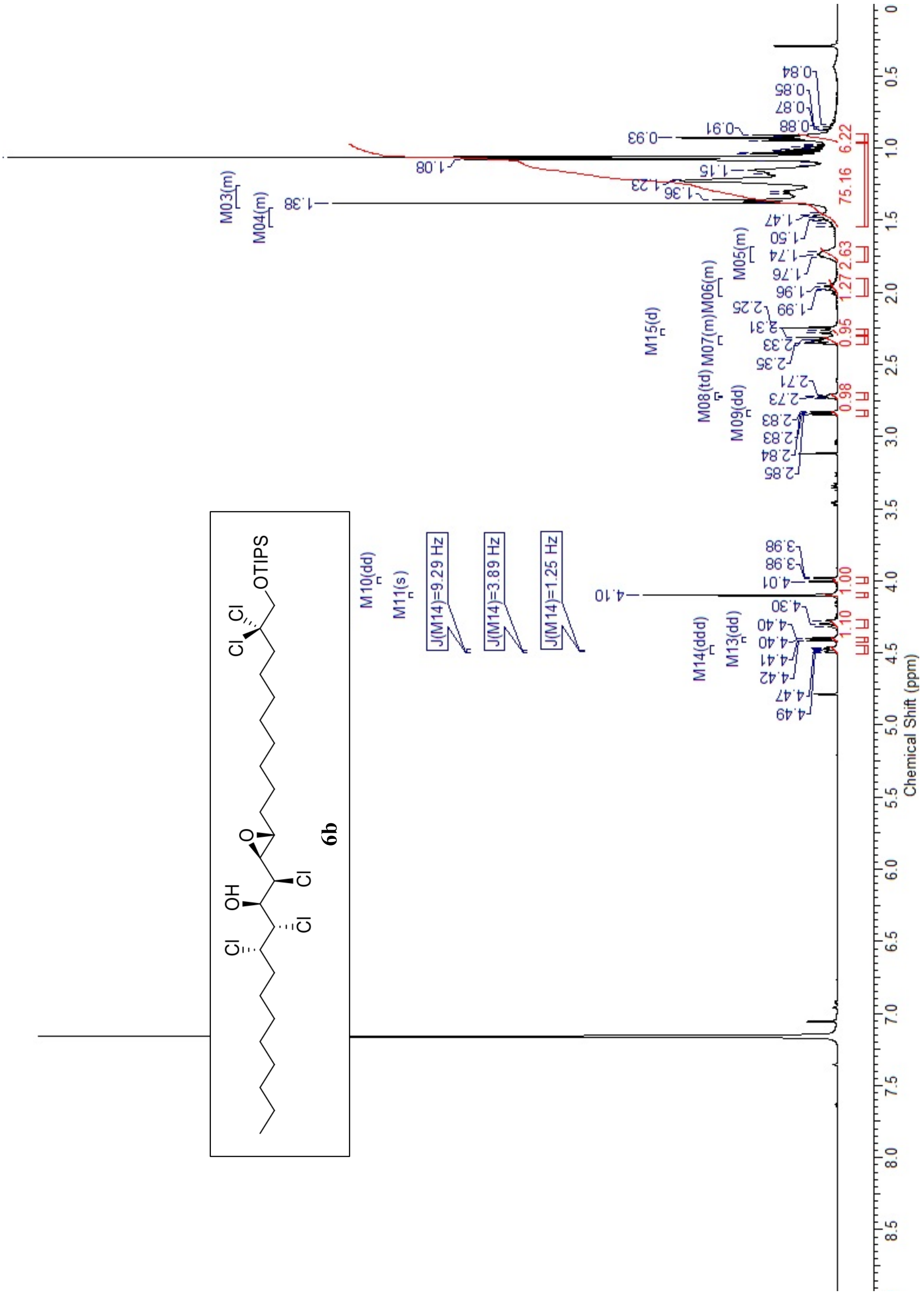

OTIPS
6b
Chemical Shift (ppm)

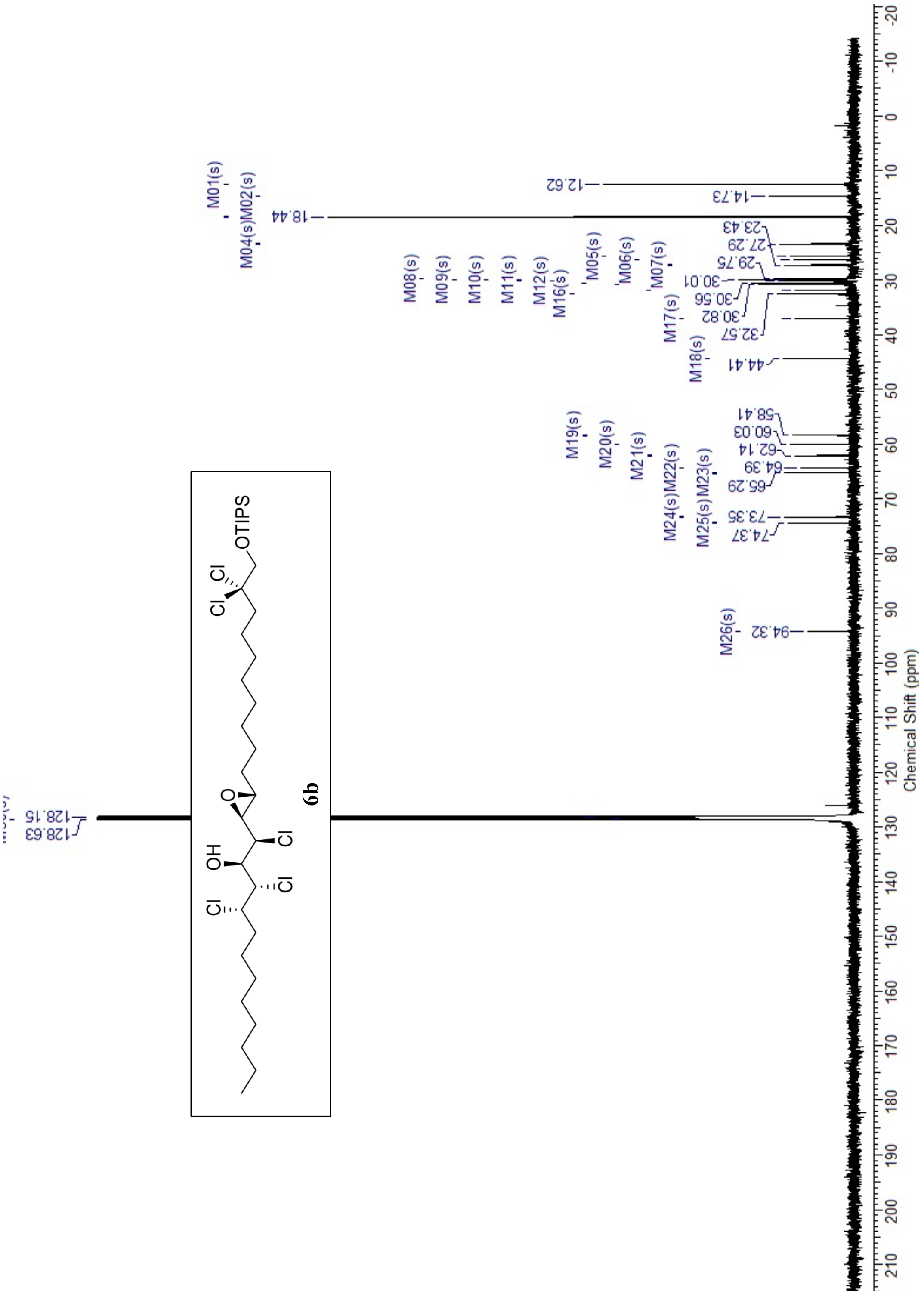

6b
OTIPS
Cl
OH
Chemical Shift (ppm)
128.15
128.63
94.32
74.37
73.35
65.29
64.39
62.14
60.03
58.41
44.41
32.57
30.82
30.56
30.01
29.75
27.29
23.43
18.44
14.73
12.62

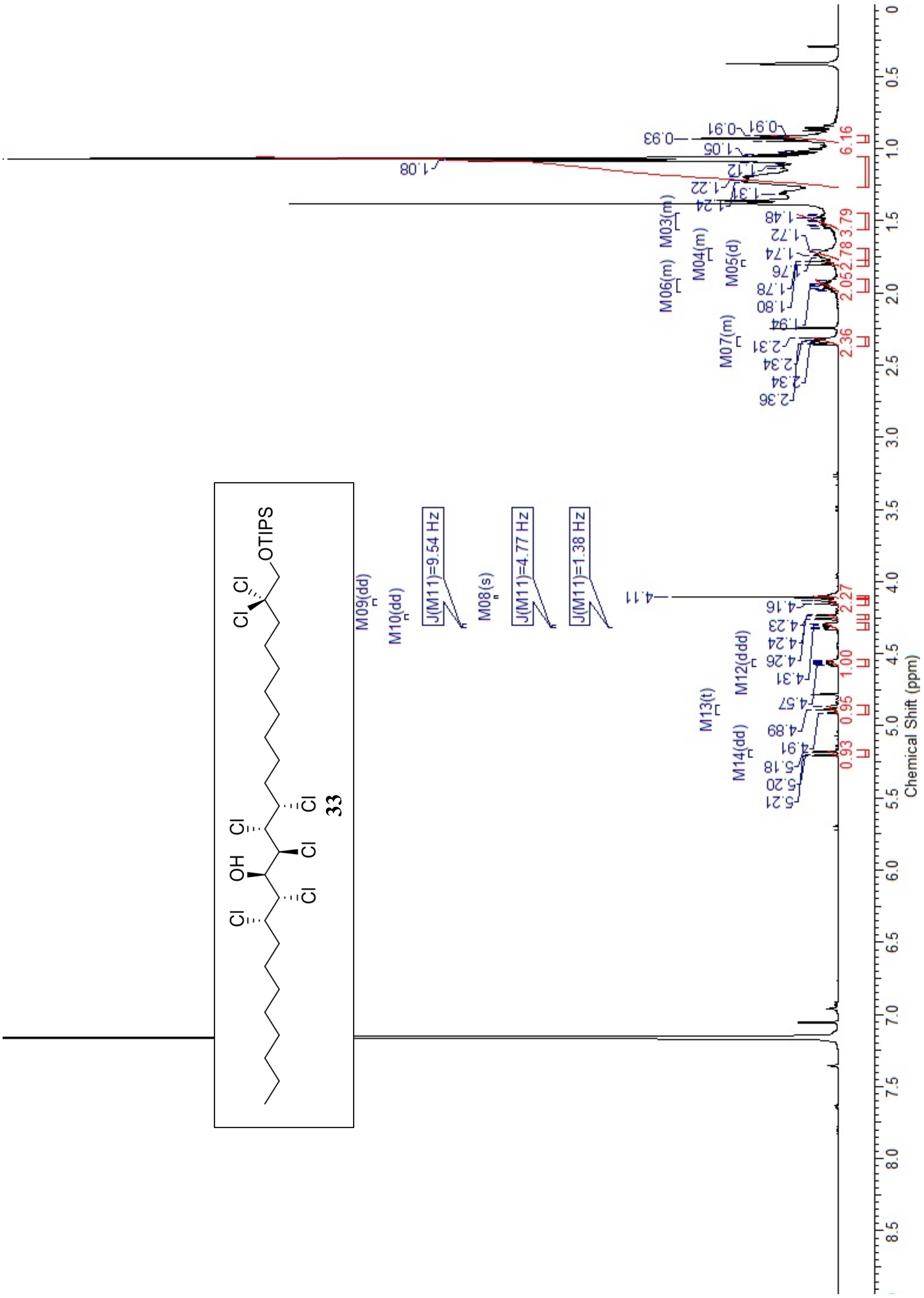

OTIPS
33
Chemical Shift (ppm)
J(M11)=9.54 Hz
J(M11)=4.77 Hz
J(M11)=1.38 Hz

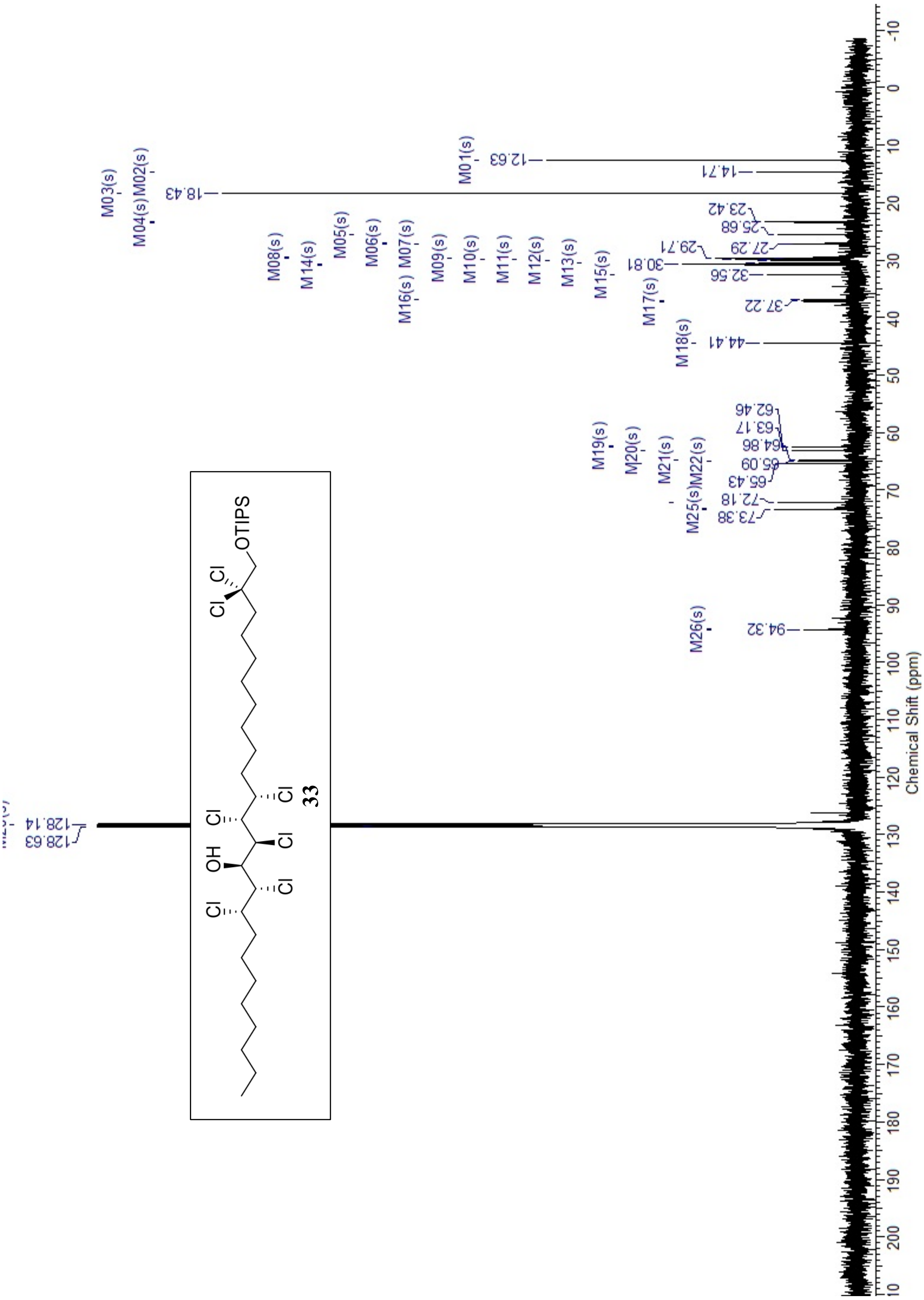
OTIPS
Cl
OH
33
12.63
14.71
18.43
23.42
25.68
27.29
29.71
30.81
32.56
37.22
44.41
62.46
63.17
64.86
65.09
65.43
72.18
73.38
94.32
128.14
128.63
Chemical Shift (ppm)

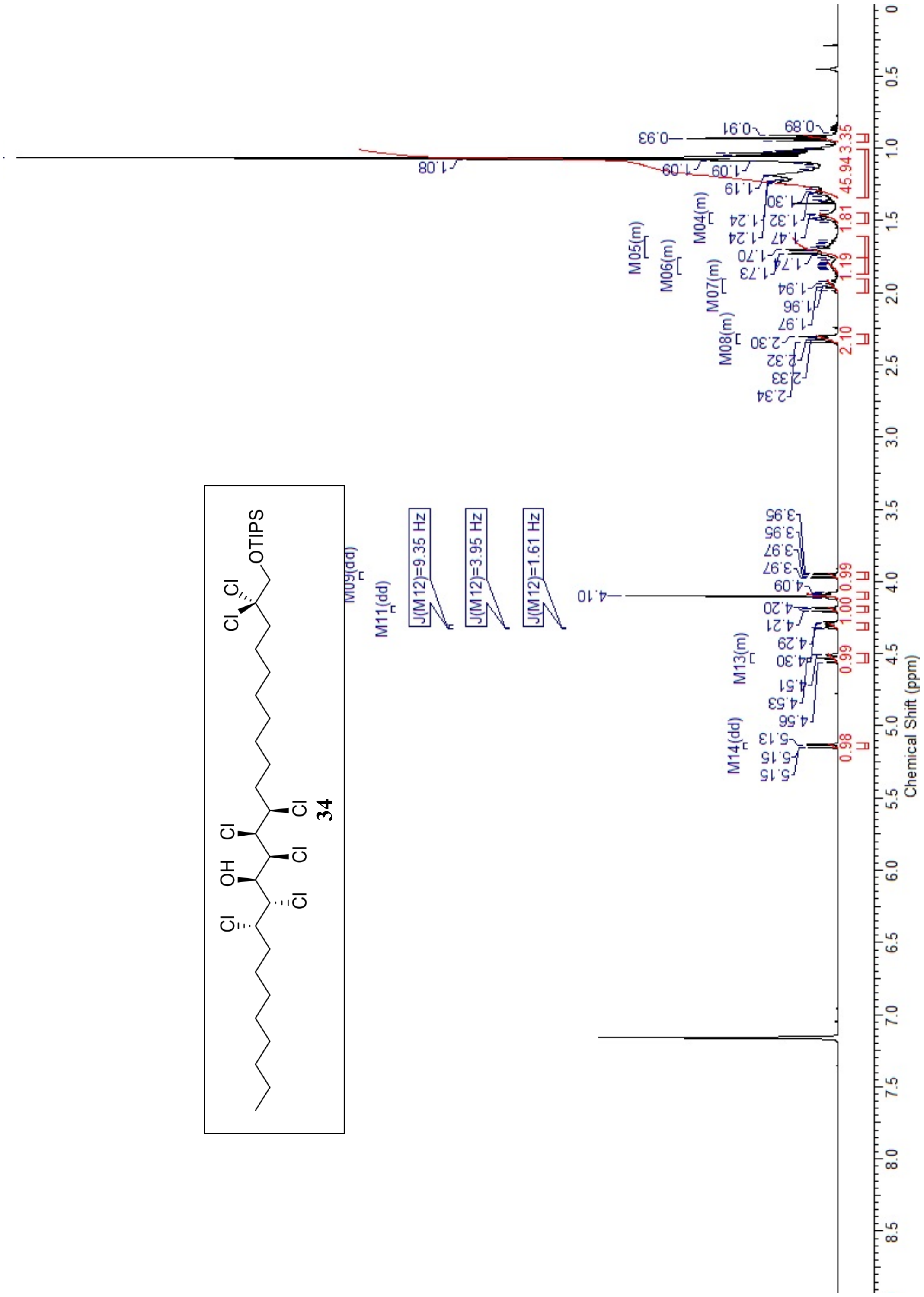
OTIPS
Cl
OH
34
Chemical Shift (ppm)
J(M12)=9.35 Hz
J(M12)=3.95 Hz
J(M12)=1.61 Hz
M04(m)
M05(m)
M06(m)
M07(m)
M08(m)
M09(dd)
M11(dd)
M13(m)
M14(dd)
45.94
3.35
1.81
1.19
2.10
0.99
1.00
0.99
0.98

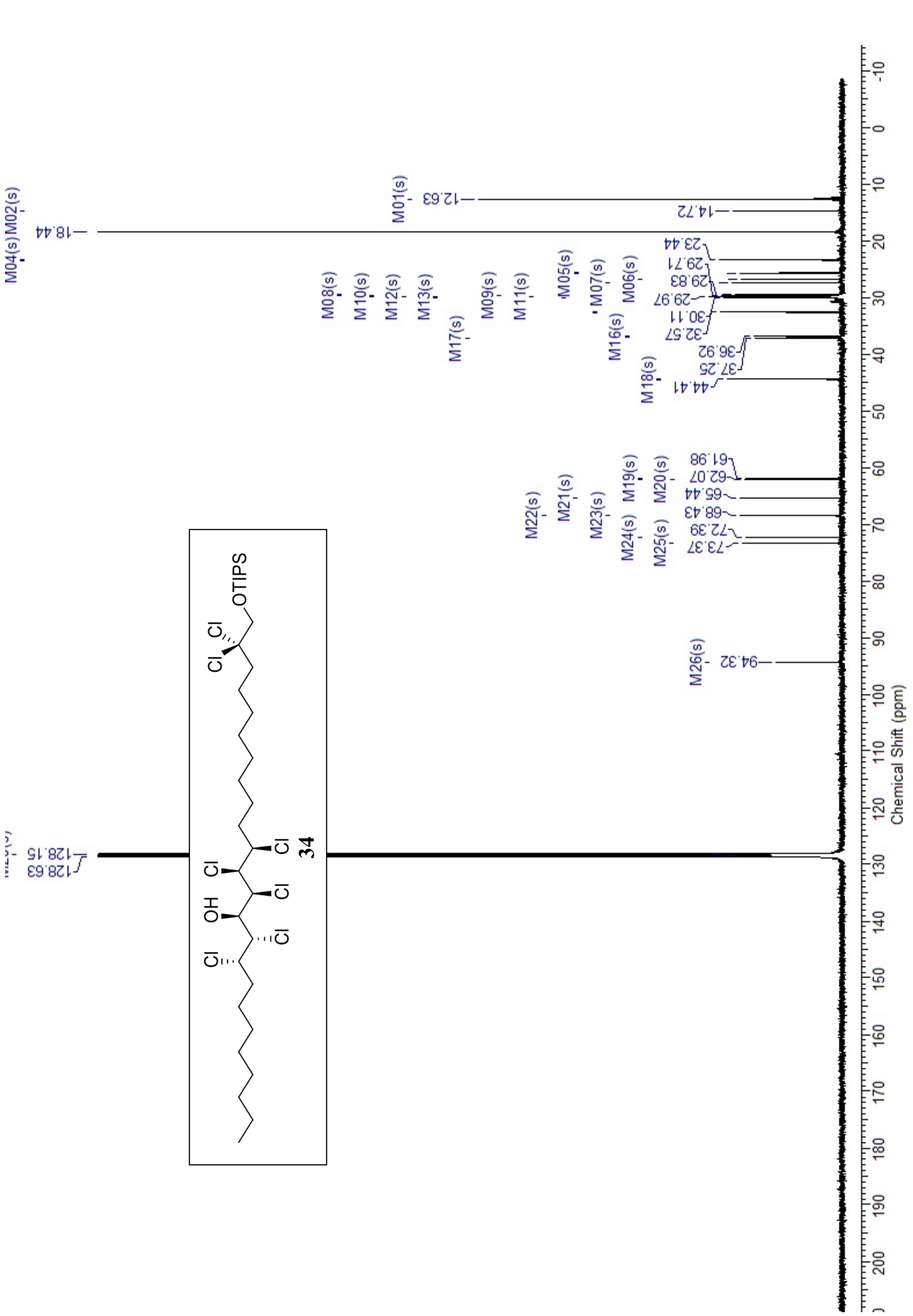

12.63
14.72
18.44
23.44
29.71
29.83
29.97
30.11
32.57
36.92
37.25
44.41
61.98
62.07
65.44
68.43
72.39
73.37
94.32
128.15
128.63
OTIPS
34
Chemical Shift (ppm)

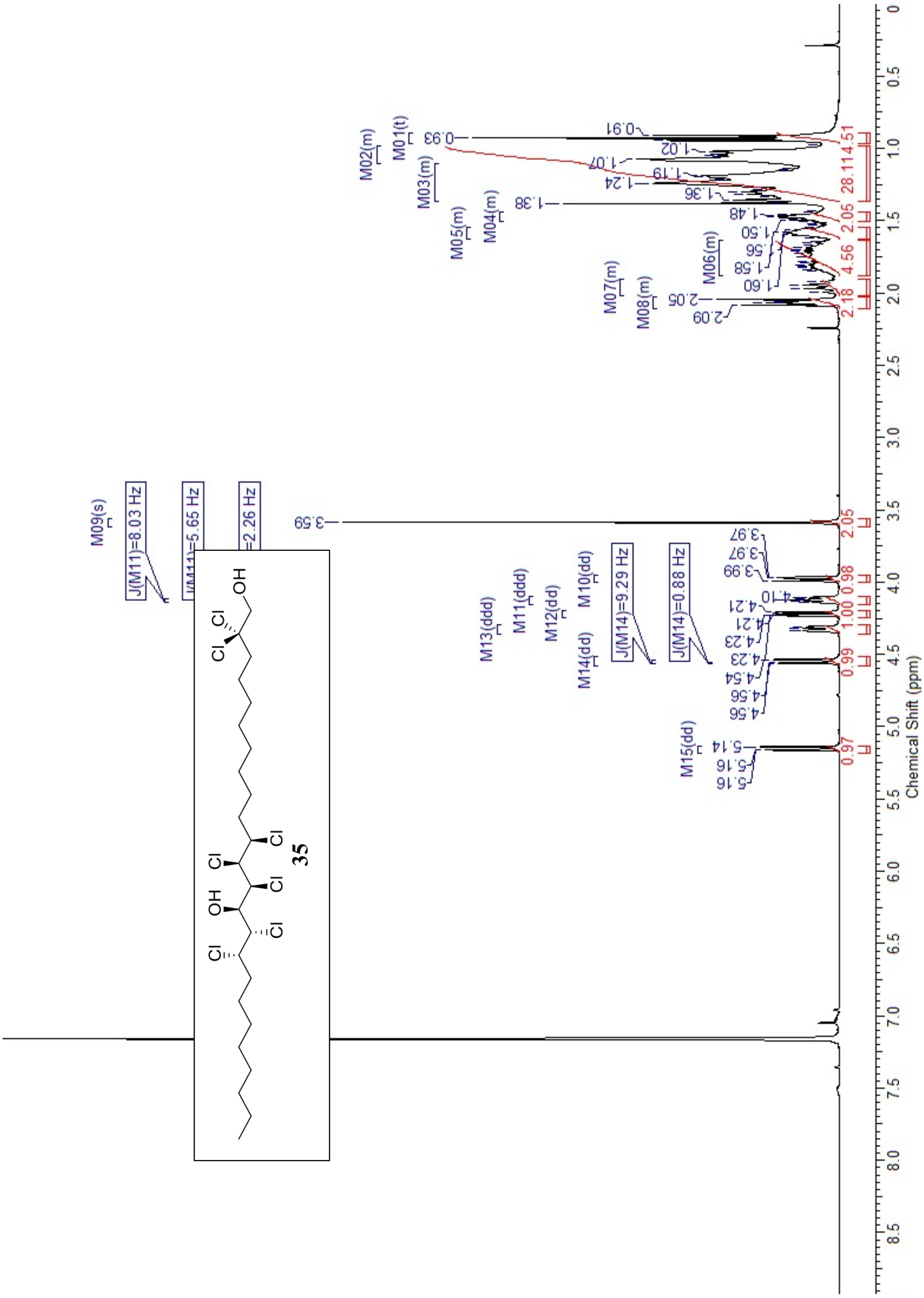
35
Chemical Shift (ppm)
M01(t)
M02(m)
M03(m)
M04(m)
M05(m)
M06(m)
M07(m)
M08(m)
M09(s)
M10(dd)
M11(ddd)
M12(dd)
M13(ddd)
M14(dd)
M15(dd)
J(M11)=8.03 Hz
J(M14)=9.29 Hz
J(M14)=0.88 Hz
28.11
4.51
2.05
4.56
2.18
2.05
0.98
1.00
0.99
0.97

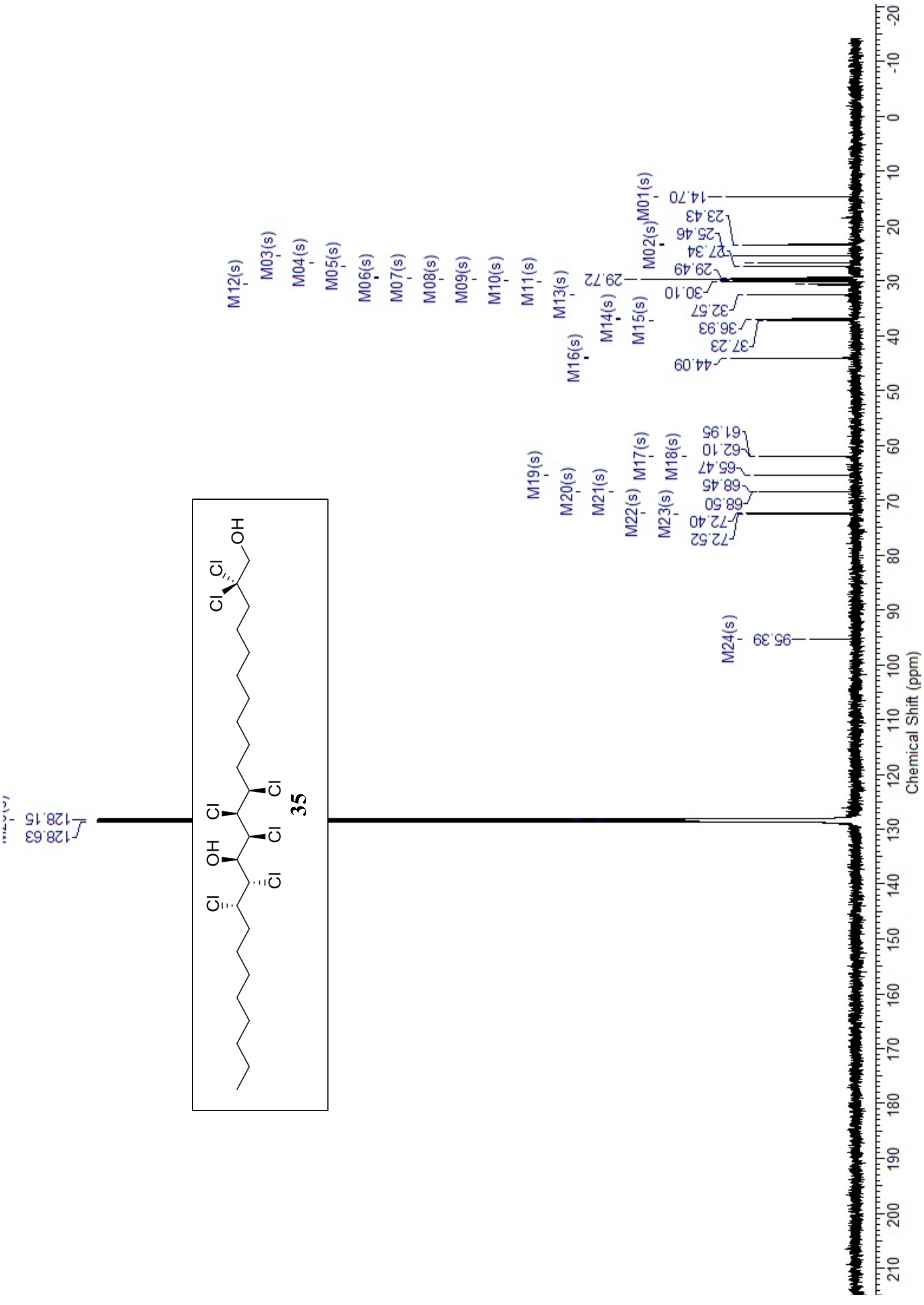

14.70
23.43
25.46
27.34
29.49
29.72
30.10
32.57
36.93
37.23
44.09
61.95
62.10
65.47
68.45
68.50
72.40
72.52
95.39
128.15
128.63
Cl
OH
35
Chemical Shift (ppm)

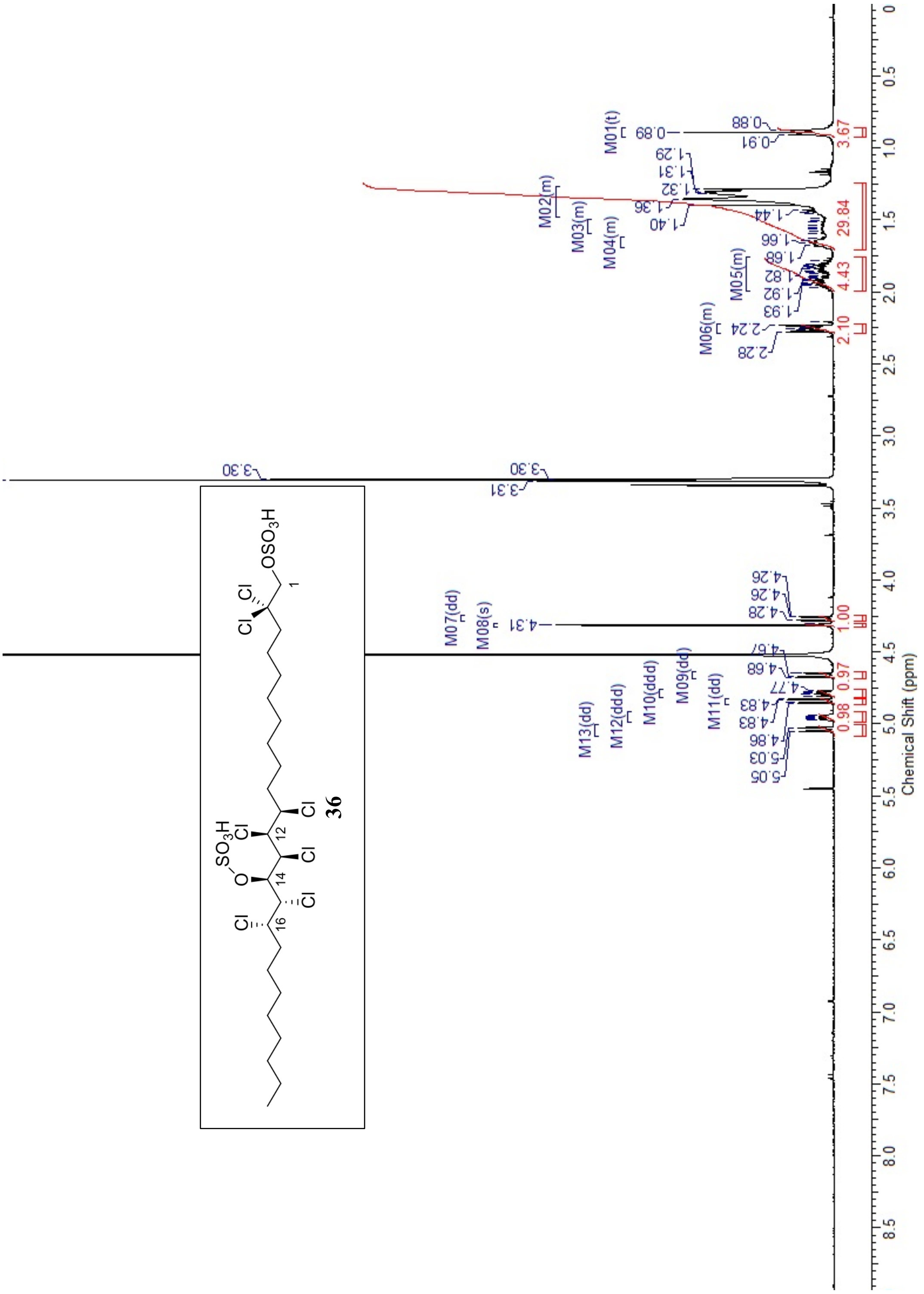

Chemical Shift (ppm)
36

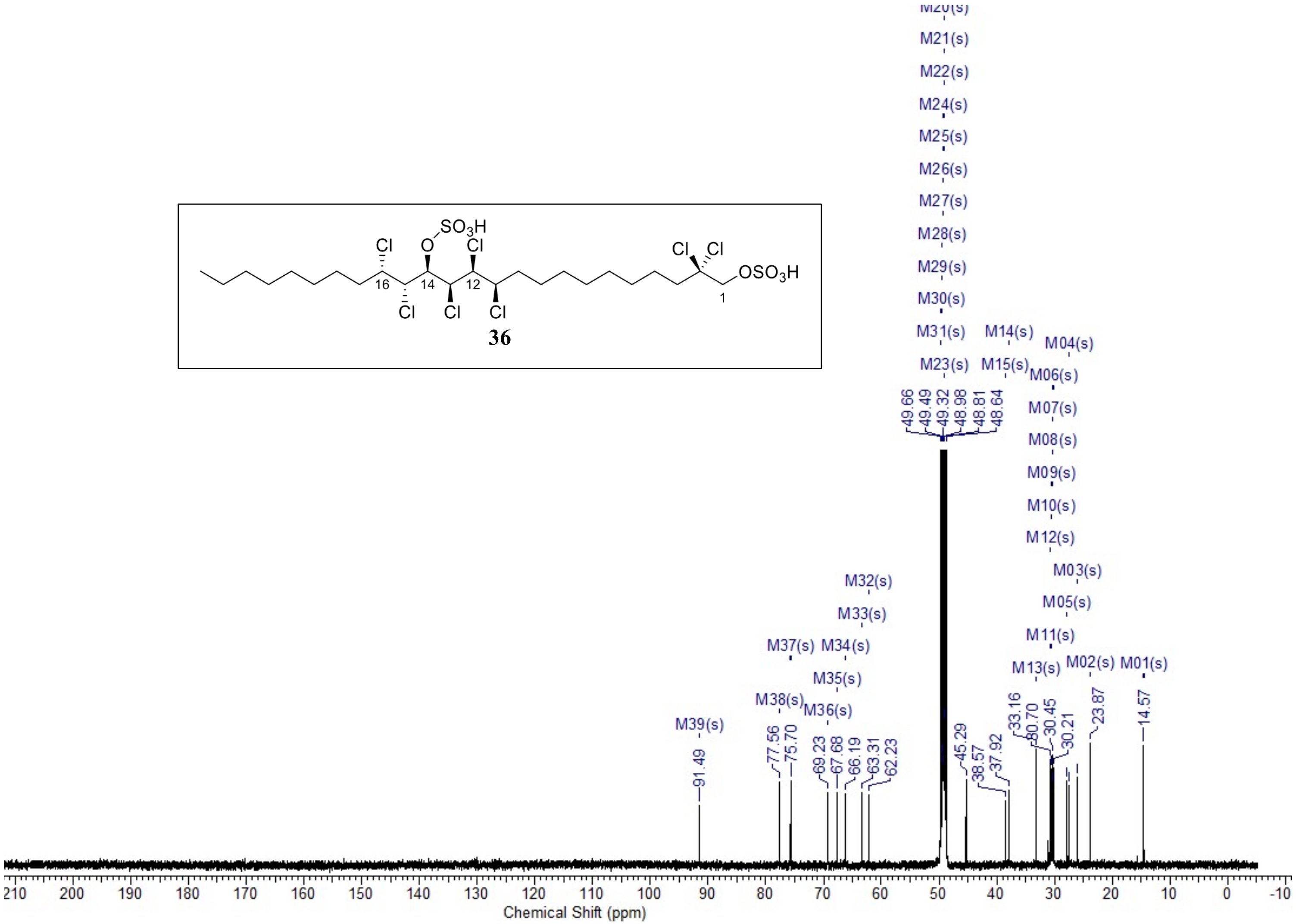
Cl
SO3H
O
Cl
Cl Cl
OSO3H
16
14
12
1
Cl
Cl
Cl
36
M20(s)
M21(s)
M22(s)
M24(s)
M25(s)
M26(s)
M27(s)
M28(s)
M29(s)
M30(s)
M31(s)
M23(s)
M14(s)
M15(s)
M04(s)
M06(s)
M07(s)
M08(s)
M09(s)
M10(s)
M12(s)
M03(s)
M05(s)
M11(s)
M13(s)
M02(s)
M01(s)
M32(s)
M33(s)
M37(s)
M34(s)
M35(s)
M38(s)
M36(s)
M39(s)
49.66
49.49
49.32
48.98
48.81
48.64
91.49
77.56
75.70
69.23
67.68
66.19
63.31
62.23
45.29
38.57
37.92
33.16
30.70
30.45
30.21
23.87
14.57
210
200
190
180
170
160
150
140
130
120
110
100
90
80
70
60
50
40
30
20
10
0
-10
Chemical Shift (ppm)

IV. X-Ray Crystallography Data

(*2S,3S,4S,5S*)-Ethyl 4,5-dichloro-2,3-dihydroxytridecanoate (15) CCDC 1470484

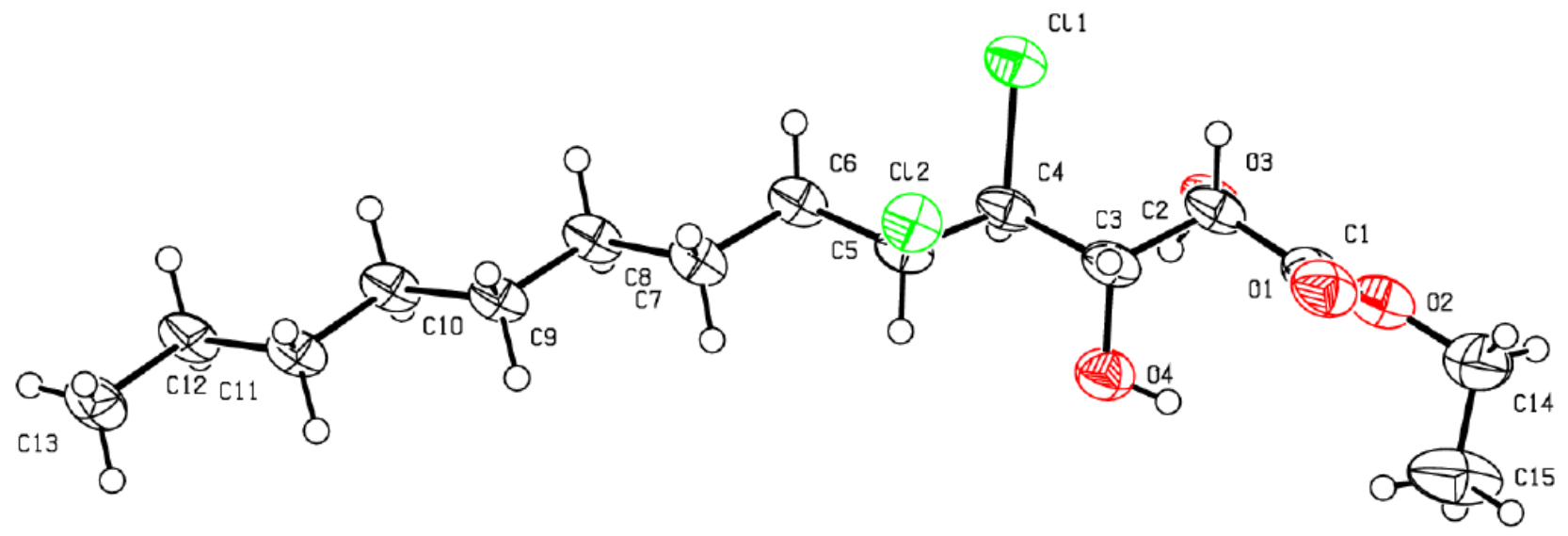

Empirical formula	$C_{15}H_{28}O_4Cl_2$
Formula weight	343.27
Crystal description	colourless block
Crystal size	0.7583 x 0.0861x 0.0217 mm
Crystal system	Orthorhombic
Space group	P 21 21 21
Unit cell dimensions	a = 5.5540(6) A alpha = 90 deg.
	b = 8.7336(10) A beta = 90 deg.
	c = 36.980(5) A gamma = 90 deg.
Volume	1793.8(4) A^3
Reflections for cell refinement	4779
Z	4
Density (calculated)	1.271 Mg/m^3
Absorption coefficient	3.359 mm^-1
F(000)	736.0
Wavelength	1.5418 A
Theta range for data collection	9.566 to 152.776 deg.
Index ranges	-6<=h<=6, -10<=k<=9,
	-45<=l<=46
Independent reflections	3611 [R(int) = 0.1037]
Data / restraints / parameters	3611/168/194 (least-squares on F^2)
Final R indices [I>2sigma(I)]	R1 = 0.1349, wR2 = 0.3369
Final R indices (all data)	R1 = 0.1504, wR2 = 0.3452
Goodness-of-fit on F^2	1.084

Absolute structure parameter	0.10(4)
Final maximum delta/sigma	0.000
Largest diff. peak and hole	1.45 and -0.75 e.A^-3

(*4R,5S,6S,7S,E*)-Ethyl-4,6,7-trichloro-5-hydroxypentadec-2-enoate (20) CCDC 1470483

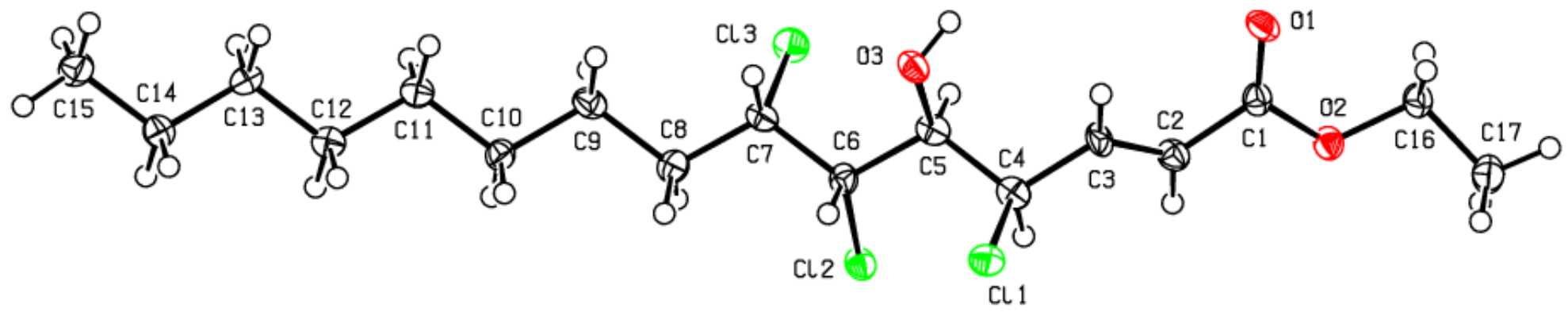

Empirical formula	$C_{17}H_{29}Cl_3O_3$
Formula weight	387.75
Crystal description	colourless block
Crystal size	0.4221 x 0.0789 x 0.0506 mm
Crystal system	Orthorhombic
Space group	P 21 21 21
Unit cell dimensions	a = 5.1865(3) A alpha = 90 deg.
	b = 9.3623(5) A beta = 90 deg.
	c = 40.6608(18) A gamma = 90 deg.
Volume	1974.38(17) A^3
Reflections for cell refinement	4779
Z	4
Density (calculated)	1.304 Mg/m^3
Absorption coefficient	4.291 mm^-1
F(000)	824
Wavelength	1.5418 A
Theta range for data collection	4.35 to 74.54 deg.
Index ranges	-4<=h<=6, -10<=k<=11,
	49<=l<=47
Independent reflections	3919 [R(int) = 0.0480]
Observed reflections	3563 [II>2\s(I)]
Absorption correction	Gaussian (Tmin = 0.3, Tmax = 1.096)
Decay correction	0%

Structure solution by	direct and difmap methods
Hydrogen atom location	geom
Hydrogen atom treatment	constr
Data / restraints / parameters	3919/0/212 (least-squares on F^2)
Final R indices [I>2sigma(I)]	R1 = 0.0638, wR2 = 0.1382
Final R indices (all data)	R1 = 0.0726, wR2 = 0.1414
Goodness-of-fit on F^2	1.218
Absolute structure parameter	0.02(3)
Final maximum delta/sigma	0.000
Largest diff. peak and hole	0.530 and -0.512 e.A^-3

V. Computational Data

All quantum chemical calculations were performed with Gaussian 09 rev. D.01.[1] Geometry optimizations were carried out with a universal solvation model (SMD) of toluene and confirmed as minima or transition structures by frequency analysis.[2] Various density functional methods were tested including hybrids with an admixture of exact exchange, employing the generalized gradient approximation (GGAs, B3LYP and wB97XD) and meta GGA (M06-2X, M11, M11-L).[3] The inclusion of density-independent atom-pairwise dispersion correction was tested with B3LYP-D3 calculations, employing a Becke-Johnson damping function at short range.[4] These results were compared with a pertubative double-hybrid funtional, B2-PLYP-D3 and explicitly correlated wavefunction theory in the form of MP2 optimizations.[5] Double, triple and quadruple zeta-valence polarized basis sets were considered in the form of Pople 6-31+G(d) and Karlsruhe def2-TZVPP and def2-QZVPP basis sets, respectively. All electronic energies in the main text are reports in kcal/mol, with distances in Å. Absolute energies in Hartree are provided in the SI. The NCI analysis was performed using nciplot to analyze the M06-2X/def2-TZVPP densities.[6]

Table S3. Results from optimizations of **39** and **40** with a double-zeta basis set: C-Cl distances and energy relative to a separated allyl cation and alkylchloride.

Entry	Level of theory	Basis set	r (C-Cl) **39**	r (C-allyl) **40**	r (C-alkyl) **40**	ΔE_o kcal/mol
1	B3YLP	6-31+G(d)	1.92	2.11	1.89	8.7
2	B3LYP-D3	6-31+G(d)	1.91	2.06	1.89	12.1
3	wB97XD	6-31+G(d)	1.87	1.93	1.87	11.8
4	MO6-2X	6-31+G(d)	1.87	1.92	1.87	13.5
5	M11	6-31+G(d)	1.87	1.91	1.87	14.4
6	M11-L	6-31+G(d)	1.87	2.57	1.82	8.5

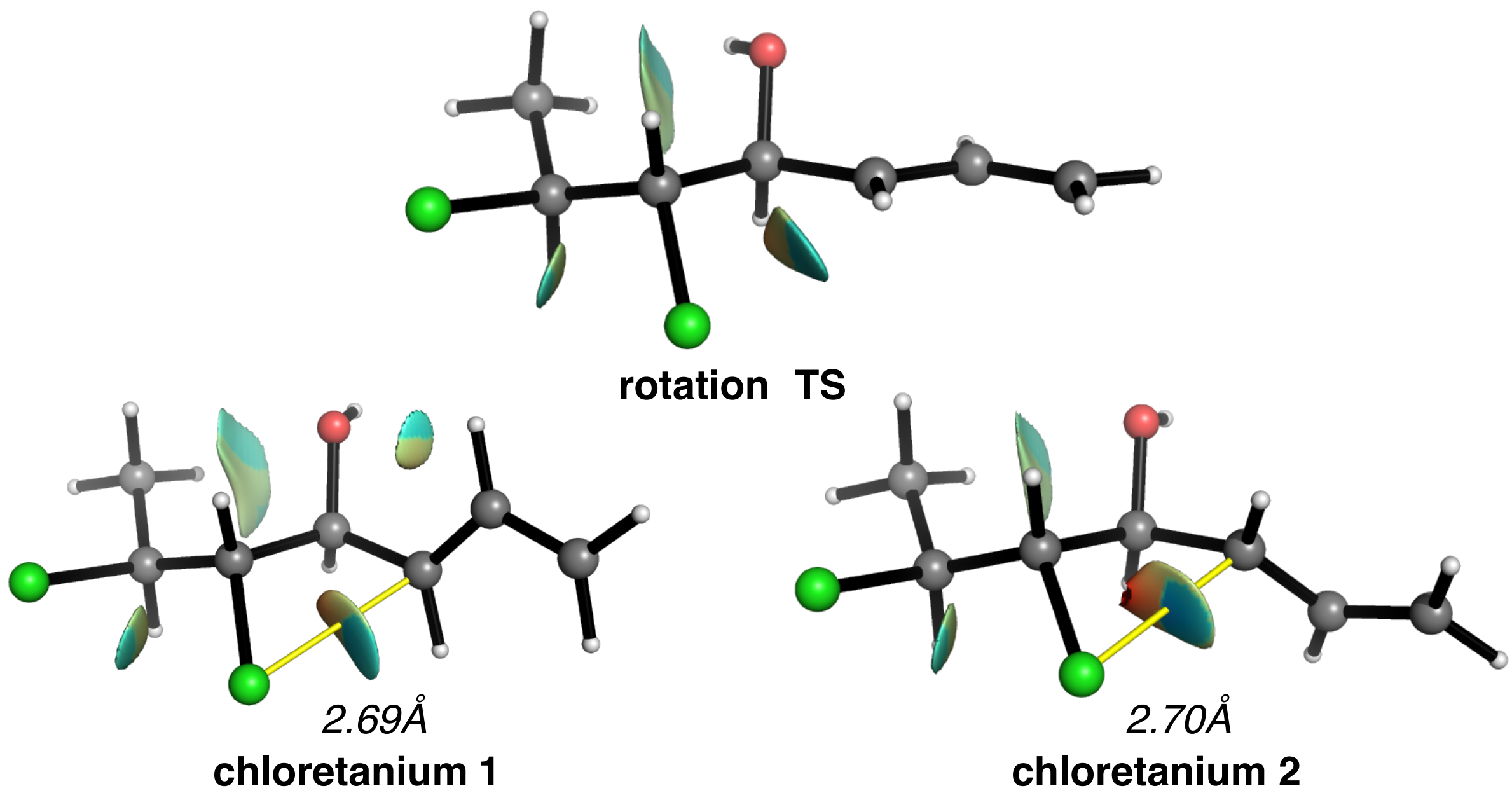

Figure S1. Computed structures and NCI (non-covalent interaction) isosurfaces for the interconversion of the epimeric chloretanium intermediates. A weakly attractive nonbonding interaction remains along the reaction coordinate between the chlorine and C13.

SMD-M06-2X/def2-TZVPP Cartesian Coordinates:

Chloretanium 1:

E: -1268.70427359 **ZPE:** 0.171497 **H:** -1268.520777 **G:** -1268.569708

C	0.33941800	-0.06675500	-0.09484800
C	-2.06913500	0.08112100	0.49070700
C	-0.78932900	0.83649900	0.48512300
H	-0.54433400	1.07957100	1.52691100
O	-0.87070600	1.96685400	-0.33278900
H	-1.00432900	2.76048500	0.19509400
C	1.72641700	0.32874400	0.40567600
H	1.82749700	0.00900900	1.44202200
C	2.04659000	1.80429300	0.26767600
H	3.07901600	1.97059200	0.56804300
H	1.41050700	2.40186200	0.92139100
H	1.91978400	2.14323100	-0.75905200
Cl	2.93114600	-0.62154000	-0.52029800
H	0.27587400	0.02431000	-1.17839300
Cl	0.02530200	-1.79130500	0.29185400
H	-2.22585300	-0.64872500	1.27620500
C	-3.06588400	0.26690800	-0.45309400
C	-4.16430400	-0.53599500	-0.36125400
H	-2.94886100	0.99922600	-1.23964400
H	-4.96845100	-0.48355100	-1.08685100
H	-4.25746000	-1.26135900	0.44023000

Chlorolanium 1:

E: -1268.69115887 **ZPE:** 0.169966 **H:** -1268.508376 **G:** -1268.560149

C	0.96192700	0.77798100	0.57109800
H	1.55718800	1.44571200	1.19326100
C	-0.44074800	0.63206800	1.16989100
H	-0.29489900	0.35573300	2.21698300
C	1.61991400	-0.58078600	0.57741300
C	-1.21134100	-0.53678400	0.49353200
Cl	0.21047600	-1.71142600	0.01728000

H	1.77343300	-0.90342200	1.60586000
H	-1.76088500	-1.12152600	1.22599400
C	-2.02087300	-0.25782400	-0.70894000
C	-3.28562300	-0.65369000	-0.76696900
C	2.81115400	-0.84036000	-0.29334000
H	2.60018800	-0.65309000	-1.34291100
H	3.17349600	-1.85753800	-0.16199700
H	3.59752800	-0.15494900	0.03122000
Cl	0.89734500	1.50837700	-1.05072400
O	-1.19933600	1.78618500	1.17116300
H	-1.27670500	2.13946400	0.27538800
H	-3.90414900	-0.41662400	-1.62240800
H	-3.74452700	-1.21226800	0.04050300
H	-1.56541200	0.29722700	-1.51874300

Chloretanium 2:

E: −1268.70576014 **ZPE:** 0.17068 **H:** −1268.522805 **G:** −1268.572542

C	0.42953500	0.00151600	-0.27845500
C	-2.02794900	0.18312300	-0.28297300
C	-0.75888000	0.86408400	0.13997900
H	-0.75069500	1.03019500	1.22019000
O	-0.76398200	2.06560700	-0.59447200
H	-1.10838900	2.78969500	-0.05978800
C	1.71690800	0.31232800	0.46603700
H	1.59804800	0.04534600	1.51552700
C	2.14115500	1.76102200	0.31999000
H	3.10590100	1.90388300	0.80231700
H	1.42056900	2.42336400	0.79981100
H	2.22895100	2.03988100	-0.72938300
Cl	3.00638700	-0.75512200	-0.16888100
H	0.57203200	0.08467600	-1.35597700
H	-2.09111700	-0.07372400	-1.33814400
Cl	-0.11126200	-1.68745200	0.04360900
C	-3.14654900	0.01708900	0.52286900
C	-4.27142400	-0.49151100	-0.04842000
H	-4.29001600	-0.75356000	-1.10047400
H	-5.17687100	-0.65458200	0.52453200
H	-3.11047300	0.27781200	1.57263800

Chlorolanium 2:

E: −1268.69060334 **ZPE:** 0.17025 **H:** −1268.507549 **G:** −1268.558791

C	0.45034200	0.79121000	-0.85162100
C	-1.39314900	-0.81435000	-0.71303800
C	1.19033400	-0.09018700	0.16581400
Cl	0.04409400	-1.61809600	0.23042100
H	-1.21691400	-1.16693200	-1.72730400
C	-2.68176700	-1.31854900	-0.14061000
H	-2.77073100	-1.10400000	0.92096800
H	-2.79322200	-2.38658200	-0.31553900
H	-3.48843300	-0.80212000	-0.66627900
H	1.10568200	0.30952800	1.17245900
C	2.55195100	-0.51386000	-0.19884700
C	3.56607500	-0.34117400	0.63923800
O	0.85175500	2.11034400	-0.68882800
H	1.71379200	2.25024900	-1.09622400
H	4.57884800	-0.58822500	0.34924200
H	3.41810000	0.06395600	1.63333200
H	2.69586600	-0.91955600	-1.19424600
C	-1.05072400	0.65650900	-0.61740900
H	-1.60327700	1.18644400	-1.39328500
Cl	-1.50937000	1.36160200	0.94530900
H	0.65757600	0.41729100	-1.86108100

TS Chloretanium 1 to 2: ($\nu = 126.95i$ cm^{-1})

E: −1268.68556086 **ZPE:** 0.169754 **H:** −1268.503863 **G:** −1268.55300

C	0.43528300	-0.14117400	-0.22611400
C	-2.09768600	-0.15028200	-0.16649000
C	-0.84623700	0.59043900	0.20527300

H	-0.84909900	0.66851800	1.30339700
O	-1.00368400	1.83907700	-0.41822800
H	-0.63575400	2.54269700	0.12545100
C	1.71074300	0.40883200	0.40994600
H	1.73180000	0.11861300	1.45936100
C	1.89664700	1.90954000	0.28067400
H	2.90199300	2.17339000	0.60205000
H	1.20935000	2.43718600	0.94597200
H	1.76090800	2.24948600	-0.74523400
Cl	3.10371800	-0.42536200	-0.34899900
H	0.51537300	-0.12586700	-1.31298600
H	-2.03633700	-1.05591100	-0.76242000
Cl	0.23031000	-1.84796800	0.28002700
C	-3.34889900	0.32224600	0.18598500
C	-4.42582600	-0.41510300	-0.20925400
H	-4.29243000	-1.31675500	-0.79719200
H	-5.43875900	-0.13280500	0.05317100
H	-3.46021300	1.22845700	0.76664300

Computational References:

[1] *Gaussian 09*, Revision **D.01**, M. J. Frisch, G. W. Trucks, H. B. Schlegel, G. E. Scuseria, M. A. Robb, J. R. Cheeseman, G. Scalmani, V. Barone, B. Mennucci, G. A. Petersson, H. Nakatsuji, M. Caricato, X. Li, H. P. Hratchian, A. F. Izmaylov, J. Bloino, G. Zheng, J. L. Sonnenberg, M. Hada, M. Ehara, K. Toyota, R. Fukuda, J. Hasegawa, M. Ishida, T. Nakajima, Y. Honda, O. Kitao, H. Nakai, T. Vreven, J. A. Montgomery, Jr., J. E. Peralta, F. Ogliaro, M. Bearpark, J. J. Heyd, E. Brothers, K. N. Kudin, V. N. Staroverov, R. Kobayashi, J. Normand, K. Raghavachari, A. Rendell, J. C. Burant, S. S. Iyengar, J. Tomasi, M. Cossi, N. Rega, J. M. Millam, M. Klene, J. E. Knox, J. B. Cross, V. Bakken, C. Adamo, J. Jaramillo, R. Gomperts, R. E. Stratmann, O. Yazyev, A. J. Austin, R. Cammi, C. Pomelli, J. W. Ochterski, R. L. Martin, K. Morokuma, V. G. Zakrzewski, G. A. Voth, P. Salvador, J. J. Dannenberg, S. Dapprich, A. D. Daniels, Ö. Farkas, J. B. Foresman, J. V. Ortiz, J. Cioslowski, and D. J. Fox, Gaussian, Inc., Wallingford CT, 2009.
[2] A. V. Marenich, C. J. Cramer, and D. G. Truhlar, *J. Phys. Chem. B* **2009***, 13*, 6378.
[3] (a) A. D. Becke, *J. Chem. Phys.* **1993**, *98*, 5648; (b) J.-D. Chai and M. Head-Gordon, *Phys. Chem. Chem. Phys*. **2008**, *10*, 6615; (c) Y. Zhao, N. E. Schultz, and D. G. Truhlar, J. Chem. Theory and Comput. **2006**, *2*, 364.; (d) R. Peverati and D. G. Truhlar, *J. Phys. Chem. Lett.* **2012**, *3*, 117.
[4] (a) S. Grimme, S. Ehrlich and L. Goerigk, *J. Comp. Chem.* **2011**, *32*, 1456.
[5] (a) L. Goerigk and S. Grimme, *J. Chem. Theory Comput.* **2011**, *7*, 291-309.; (b) M. Head-Gordon, J. A. Pople, and M. J. Frisch, *Chem. Phys. Lett.* **1988***, 153*, 503.
[6] (a) J. Contreras-Garcia, E. Johnson, S. Keinan, R. Chaudret, J-P. Piquemal, D. Beratan, W. Yang, J. *Chem. Theor. Comp.* **2011**, *7*, 625; (b) E R. Johnson, S. Keinan, P. Mori-Sanchez, J. Contreras-Garcia, A. J. Cohen, and W. Yang, *J. Am. Chem. Soc.* **2010**, *132*, 6498.
